# Randomized, Placebo-Controlled Trial of Mipomersen in Patients with Severe Hypercholesterolemia Receiving Maximally Tolerated Lipid-Lowering Therapy

**DOI:** 10.1371/journal.pone.0049006

**Published:** 2012-11-13

**Authors:** Mary P. McGowan, Jean-Claude Tardif, Richard Ceska, Lesley J. Burgess, Handrean Soran, Ioanna Gouni-Berthold, Gilbert Wagener, Scott Chasan-Taber

**Affiliations:** 1 Cardiometabolic Risk Reduction and Research Center of New England, Bedford, New Hampshire, United States of America; 2 Montreal Heart Institute, Université de Montréal, Montreal, Quebec, Canada; 3 Centre for Preventive Cardiology, General Teaching Hospital and First Medical Faculty of Charles University in Prague, Prague, Czech Republic; 4 Cardiology Unit, Stellenbosch University and Tygerberg Hospital, Parow, South Africa; 5 University Department of Medicine, Central Manchester Foundation Trust, Manchester, United Kingdom; 6 Center of Endocrinology, Diabetes and Preventive Medicine, University of Cologne, Cologne, Germany; 7 Clinical Research, Genzyme Corporation, Almere Stad Area, The Netherlands; 8 Biostatistics, Genzyme Corporation, Cambridge, Massachusetts, United States of America; University of Virginia Health System, United States of America

## Abstract

**Objectives:**

Mipomersen, an antisense oligonucleotide targeting apolipoprotein B synthesis, significantly reduces LDL-C and other atherogenic lipoproteins in familial hypercholesterolemia when added to ongoing maximally tolerated lipid-lowering therapy. Safety and efficacy of mipomersen in patients with severe hypercholesterolemia was evaluated.

**Methods and Results:**

Randomized, double-blind, placebo-controlled, multicenter trial. Patients (n  = 58) were ≥18 years with LDL-C ≥7.8 mmol/L or LDL-C ≥5.1 mmol/L plus CHD disease, on maximally tolerated lipid-lowering therapy that excluded apheresis. Weekly subcutaneous injections of mipomersen 200 mg (n  = 39) or placebo (n  = 19) were added to lipid-lowering therapy for 26 weeks. Main outcome: percent reduction in LDL-C from baseline to 2 weeks after the last dose of treatment. Mipomersen (n = 27) reduced LDL-C by 36%, from a baseline of 7.2 mmol/L, for a mean absolute reduction of 2.6 mmol/L. Conversely, mean LDL-C increased 13% in placebo (n = 18) from a baseline of 6.5 mmol/L (mipomersen vs placebo p<0.001). Mipomersen produced statistically significant (p<0.001) reductions in apolipoprotein B and lipoprotein(a), with no change in high-density lipoprotein cholesterol. Mild-to-moderate injection site reactions were the most frequently reported adverse events with mipomersen. Mild-to-moderate flu-like symptoms were reported more often with mipomersen. Alanine transaminase increase, aspartate transaminase increase, and hepatic steatosis occurred in 21%, 13% and 13% of mipomersen treated patients, respectively. Adverse events by category for the placebo and mipomersen groups respectively were: total adverse events, 16(84.2%), 39(100%); serious adverse events, 0(0%), 6(15.4%); discontinuations due to adverse events, 1(5.3%), 8(20.5%) and cardiac adverse events, 1(5.3%), 5(12.8%).

**Conclusion:**

Mipomersen significantly reduced LDL-C, apolipoprotein B, total cholesterol and non-HDL-cholesterol, and lipoprotein(a). Mounting evidence suggests it may be a potential pharmacologic option for lowering LDL-C in patients with severe hypercholesterolemia not adequately controlled using existing therapies. Future studies will explore alternative dosing schedules aimed at minimizing side effects.

**Trial Registration:**

ClinicalTrials.gov NCT00794664.

## Introduction

Familial hypercholesterolemia (FH), an autosomal dominant disorder characterized by elevated plasma levels of low-density lipoprotein cholesterol (LDL-C) is most commonly caused by a mutation in the LDL receptor gene. [1] The risk of a coronary event before age 65 is 85% in men and 50% in women with untreated heterozygous FH. [2] Although high dose statin therapy in combination with other lipid-lowering agents is effective in these patients, a small percentage have refractory severe hypercholesterolemia.[3]–[6] In small non-randomized studies LDL-apheresis reduces cardiac events and improves overall survival; however, it has limited availability, generally requires a fistula, and costs over €2500 for biweekly sessions. [5], [7] In the US, fewer than 200 of the 6000 qualified persons with severe hypercholesterolemia receive apheresis. [7] Agents capable of lowering LDL-C via novel mechanisms of action are needed.

In previous trials, weekly subcutaneous (SC) injections of mipomersen 200 mg reduced LDL-C by 25% and 28% in patients with FH when added to existing treatments. [8], [9] Mipomersen is an antisense oligonucleotide inhibitor of apolipoprotein B (Apo B) synthesis. It binds to messenger RNA encoding apolipoprotein B preventing its synthesis and secretion of Apo B containing atherogenic lipoproteins. [8], [10] This study assessed the safety and efficacy of mipomersen 200 mg per week in patients with severe hypercholesterolemia receiving maximally tolerated lipid-lowering therapy that excluded apheresis and found treatment to significantly lower atherogenic lipoproteins.

**Figure 1:**
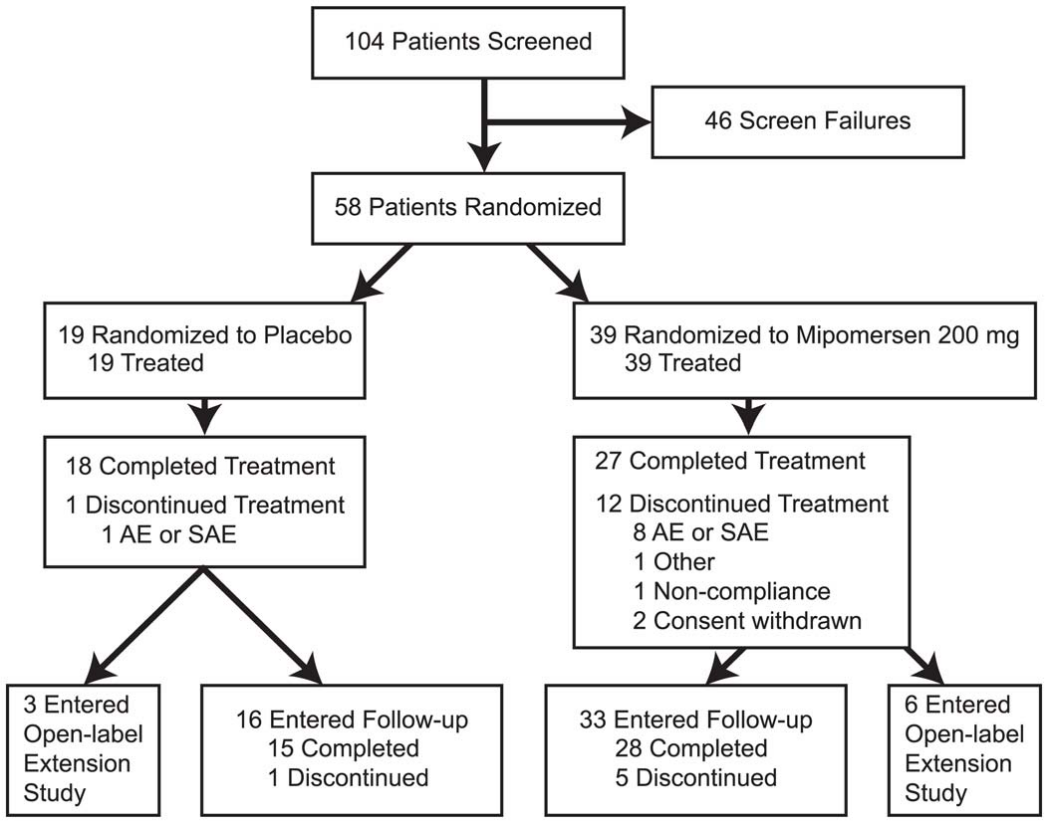
Patient Disposition – Consort Diagram.

**Table 1:** Baseline Characteristics.

	Placebo	Mipomersen	Total
	(N = 19)	(N = 39)	(N = 58)
Age (years) Mean (SD)	47·9 (13·5)	51·8 (14·3)	50·5 (14·0)
*[Min, Max]*	*[18, 66]*	*[21, 77]*	*[18, 77]*
Gender n (%)			
Male	7 (36·8)	18 (46·2)	25 (43·1)
Female	12 (63·2)	21 (53·8)	33 (56·9)
Race n (%)			
White	16 (84·2)	33 (84·6)	49 (84·5)
Black	1 (5·3)	2 (5·1)	3 (5·2)
Asian	0 (0·0)	1 (2·6)	1 (1·7)
Multiple	2 (10·5)	1 (2·6)	3 (5·2)
Other	0 (0·0)	2 (5·1)	2 (3·4)
BMI (kg/m^2^)			
Mean (SD)	29·9 (4·10)	28·4 (5·35)	28·9 (4·98)
* [Min, Max]*	*[22·8, 36·9]*	*[19·4, 39·8]*	*[19·4, 39·8]*
Waist/hip ratio			
Mean (SD)	0·94 (0·06)	0·92 (0·09)	0·93 (0·08)
* [Min, Max]*	*[0*·*8, 1*·*0]*	*[0*·*7, 1*·*1]*	*[0*·*7, 1*·*1]*
Current smoker n (%)	5 (26·3)	4 (10·3)	9 (15·5)
Alcohol use n (%)			
Current	7 (36·8)	27 (69·2)	34 (58·6)
Non-current	4 (21·1)	5 (12·8)	9 (15·5)
Never	8 (42·1)	7 (17·9)	15 (25·9)
Metabolic syndrome, n (%)^a^			
No	10 (52·6)	25 (64·1)	35 (60·3)
Yes	9 (47·4)	14 (35·9)	23 (39·7)
History of:			
Angina	5 (26·3)	11 (28·2)	16 (27·6)
Myocardial infarction	4 (21·1)	8 (20·5)	12 (20·7)
Coronary Artery Bypass Graft	6 (31·6)	12 (30·8)	18(31·0)
Percutaneous Coronary Intervention	2 (10·5)	4 (10·3)	6 (10·3)
Coronary Artery Disease without event	5 (26·3)	11 (28·2)	16 (27·6)
Peripheral Artery Disease	2 (10·5)	1 (2·6)	3 (5·2)
Abdominal Aortic Aneurysm	0 (0·0)	1 (2·6)	1 (1·7)
Carotid Disease	5 (26·3)	6 (15·4)	11 (19·0)
Coronary Heart Disease or other Atherosclerotic disease (clinical diagnosis)	15 (78·9)	31 (79·5)	46 (79·3)
Lipid Lowering Medications, n (%)	19 (100·0)	39 (100·0)	58 (100·0)
Maximal statin	8 (42·1)	16 (41·0)	24 (41·4)
Statin with ezetimibe	11 (57·9)	20 (51·3)	31 (53·4)

Abbreviations: BMI, body mass index; Max, maximum; Min, minimum; SD, standard deviation.

a Metabolic syndrome determined according to the American Heart Association and the National Heart, Lung, and Blood Institute definition.

## Methods

### Ethics Statement

This randomized, double-blind, placebo-controlled, multi-center, Phase 3 trial was undertaken between January 27, 2009 and October 14, 2010 and performed in accordance with the Declaration of Helsinki with approval by each Institutional Review Board. This multicenter study included 26 study locations; the names of Institutions and their corresponding institutional review boards/independent ethics committees are listed in the supplemental material. At screening and after adequate explanation of the aims, methods, anticipated benefits, and potential hazards of the study, all patients provided written informed consent.

**Figure 2:**
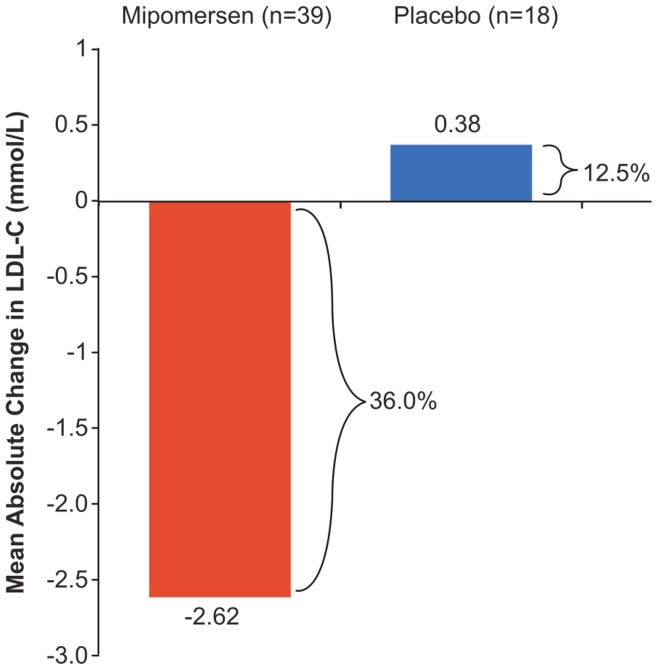
Mean Absolute Reduction in LDL-C (value closest to 2 weeks after the end of study treatment).

**Table 2:** Baseline Lipid Concentrations and Effect of Treatment on Lipid Parameters.

	Placebo (N = 18 )			Mipomersen (N = 39)			P-value^a^
Parameter	Baseline Mean (SD)	Primary Efficacy Timepoint Mean (SD)	% Change fromBaseline (95% CI)	Baseline Mean (SD)	Primary Efficacy Timepoint Mean (SD)	% Change from Baseline (95% CI)	Mipomersen vs Placebo
LDL cholesterol (mmol/L)	6·5 (2·2)	6·8 (2·6)	12·5 (−10·7, 35·8)	7·2 (1·9)	4·5 (2·1)	−35·9 (−51·3, −15·3)	<0·001
Apolipoprotein B (mmol/L)	1·8 (0·5)	1·9 (0·5)	11·4 (−6·9, 29·7)	2·0 (0·5)	1·3 (0·5)	−35·9 (−43·3, −28·4)	<0·001
Total cholesterol (mmol/L)	8·3 (2·3)	8·8 (2·6)	11·2 (−6·2, 28·5)	9·2 (2·0)	6·5 (2·1)	−28·3 (−34·9, −21·7)	<0·001
Non-HDL cholesterol (mmol/L)	7·2 (2·3)	7·7 (2·7)	14·2 (−9·6, 38·0)	7·9 (2·0)	5·1 (2·2)	−33·9 (−41·7, −26·2)	<0·001
HDL cholesterol (mmol/L)	1·1 (0·3)	1·2 (0·4)	3·2 (−5·0, 11·4)	1·3 (0·4)	1·4 (0·4)	5·8 (−1·1, 12·7)	0·650
Lipoprotein(a) (mmol/L)	0·3 (0·3)	0·3 (0·3)	−1·5 (−14·2, 11·3)	0·6 (0·7)	0·4 (0·5)	−32·7 (−43·3, −22·0)	<0·001
Triglycerides (mg/dL)	1·6 (0·6)	1·9 (0·7)	26·6 (−3·7, 56·8)	1·6 (1·0)	1·3 (0·7)	−8·6 (−21·7, 4·4)	0·034
VLDL cholesterol (mmol/L)	0·7 (0·3)	0·8 (0·3)	25·2 (−3·8, 54·1)	0·8 (0·5)	0·6 (0·3)	−9·6 (−22·4, 3·2)	0·030
Apolipoprotein A1 (mmol/L)	1·4 (0·3)	1·4 (0·3)	1·8 (−5·4, 8·9)	1·5 (0·3)	1·5 (0·3)	−3·0 (−8·2, 2·1)	0·278
LDL/HDL Ratio^b^	5·9 (3·9, 6·6)	5·8 (3·3, 7·8)	2·2 (−14·5, 17·3)	5·2 (4·1, 7·0)	3·1 (2·1, 4·3)	−41·7 (−57·4, −16·5)	<0·001

Abbreviations: CI, confidence interval; HDL, high density lipoprotein; LDL, low density lipoprotein; SD, standard deviation; VLDL, very low density lipoprotein.

a The P-values shown are for the between-group comparisons of % changes from baseline to the primary efficacy timepoint. P-values are obtained by the t-test for all parameters except LDL/HDL Ratio, where the Wilcoxon rank-sum test was used due to deviations from normality.

b Baseline and primary efficacy timepoint values are expressed as median (interquartile range); percent change is presented as median (interquartile range).

**Figure 3:**
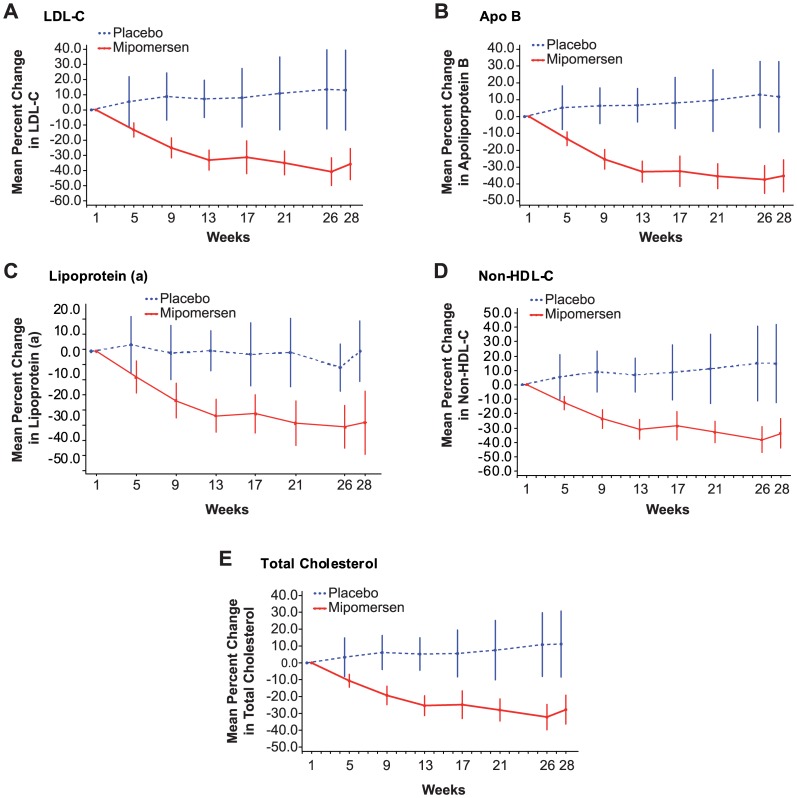
Mean Percent Change from Baseline to Week 28. Error bars indicate 95% CI. Placebo (n  = 18); mipomersen 200 mg weekly (n  = 39). (A) Low-density lipoprotein cholesterol (LDL-C) (B) Apolipoprotein B (Apo B) (C) Lipoprotein(a) (Lp(a)) (D) Non-high density lipoprotein cholesterol (non-HDL-C) (E) Total Cholesterol.

### Study Design

The protocol for this trial and supporting CONSORT checklist are available as supporting information; see Checklist S1 and Protocol S1. Adults patients with severe hypercholesterolemia defined as an LDL-C ≥5·1 mmol/L with known CHD or an LDL-C ≥7·8 mmol/L in the absence of known CHD provided written informed consent. [11] Patients were on a stable low fat diet, at a stable weight, on maximally tolerated lipid-lowering therapy, met LDL-apheresis criteria but apheresis was prohibited.[12]–[14] Exclusions included significant cardiovascular or cerebrovascular events within 24 weeks of screening, congestive heart failure, type I diabetes, poorly controlled type II diabetes, hypertension, secondary hyperlipidemia predisposition, or a history of significant renal or hepatic disease.

After 4-weeks screening, eligible patients were randomized to 26 weeks of self-administered mipomersen 200 mg per week or placebo, which consisted of 9 mg of sodium chloride, 0.004 mg of riboflavin, filled to sufficient quantity with 1 mL water for injection. The placebo was similar to active drug in appearance and administration. Permuted block randomization (blocks of size 3) was used to allocate patients to treatment in a 2∶1 ratio. There were no stratification factors used; 36 sites screened patients and 26 sites enrolled and randomized at least 1 patient. Patients were enrolled by the principal investigator at each study site after which the investigator or study coordinator called an interactive voice response system that provided blinded medication kits coded to assure that all clinical, medical, and pharmacy personnel, as well as the patient, remained blinded to treatment allocation. All clinical operations, data management, and statistical personnel also remained blinded until the database was locked. Patients underwent baseline liver and spleen magnetic resonance (MRI) or computed tomography with follow-up only in patients experiencing an ALT ≥3 X ULN on 2 consecutive results one week apart. MRI of hepatic fat content was performed by an assessor masked to treatment. [15].

Total cholesterol (TC), high-density lipoprotein cholesterol (HDL-C) and triglycerides (TG) were measured by enzymatic colorimetry. Lipoprotein(a) [Lp(a)] was measured by a standardized isoform independent assay. Rate nephelometry assessed apolipoprotein B–100 and apolipoprotein A1. [16], [17] By protocol, discontinuation was required if alanine transaminase (ALT) or aspartate aminotransferase (AST) levels were: ≥8 X ULN, ≥5 X ULN on 2 consecutive weeks, or ≥3 X ULN in conjunction with total bilirubin >1·5 X ULN or international normalized ratio >1·5.

Primary endpoint was percent change in LDL-C from baseline to 2 weeks after last dose. For study completers this was week 28. Secondary endpoints included percent change from baseline in apolipoprotein B, non-high-density lipoprotein cholesterol (non-HDL-C), and TC. Other endpoints included changes from baseline in TG, Lp(a), very low-density lipoprotein cholesterol, apolipoprotein A1, high-density lipoprotein cholesterol (HDL-C), and LDL/HDL ratio.

**Table 3:** On-Treatment Adverse Events Reported in at Least 10% of Mipomersen Patients.

	Placebo (N = 19)		Mipomersen (N = 39)	
Adverse Events	Events (#)	Patients n (%)	Events (#)	Patients n (%)
All events	69	16 (84·2)	1114	39 (100·0)
Injection site reaction	38	6 (31·6)	877	35 (89·7)
Flu-like symptoms (influenza-like illness, influenza, pyrexia, chills, myalgia, arthralgia, malaise, fatigue)	5	4 (21·1)	59	18 (46·2)
Alanine aminotransferase increased	0	0 (0)	8	8 (20·5)
Aspartate aminotransferase increased	0	0 (0)	5	5 (12·8))
Nausea	0	0 (0)	7	5 (12·8)
Hepatic steatosis	0	0 (0)	5	5 (12·8)
Nasopharyngitis	1	1 (5·3)	8	4 (10·3)
Cardiac events	1	1 (5·3)	12	5 (12·8)
Acute myocardial infarctiona, b			1	1 (2·6)
Angina pectoris^c^	1	1 (5·3)	4	2 (5·1)
Angina unstable^b^			1	1 (2·6)
Cardiac failure^b^			1	1 (2·6)
Coronary artery disease^b^			2	1 (2·6)
Prinzmetal angina^d^			2	1 (2·6)
Supraventricular extrasystoles^b^			1	1 (2.6)

a This patient also had another acute myocardial infarction occurring during post-treatment follow-up, which was fatal.

b Events not related to study drug in the opinion of the investigator.

c 3 of 4 events of angina pectoris in the mipomersen group were considered drug-related. The 4^th^ event and the event in the placebo group were considered not drug-related.

d Considered possibly drug-related.

### Statistical Analysis

Based on prior clinical experience with mipomersen the standard deviation of the percent change in LDL-C was estimated at 22%. With a 2∶1 mipomersen to placebo randomization, the study had to have at least an 80% power to detect a 20% difference between the 2 treatment groups with 45 patients using a 2-sided alpha of 5%. Efficacy comparisons employed two-sample t-test or the Wilcoxon rank sum test, as appropriate, on the intention-to-treat population and per-protocol population. Statistical significance was defined as p≤0·05 and type I error was controlled for the primary and secondary endpoints using a sequential inferential approach. All randomized patients who received at least 1 injection of study drug and had a valid baseline with at least 1 post-baseline LDL-C measurement were analyzed for efficacy. Several sensitivity analyses were performed on the primary efficacy endpoint. One analysis was run using the LDL-C value closest to week 28 for each patient, regardless of when treatment ended. In addition, a post-hoc mixed model for repeated measures analysis was conducted including the fixed, categorical effects of treatment, visit, and treatment-by-visit interaction, as well as the continuous, fixed covariate of baseline LDL-C. All randomized patients who received at least 1 injection of study drug were evaluated for safety.

**Table 4:** Laboratory Abnormalities of Interest.

	Placebo (N = 19)	Mipomersen (N = 39)
Laboratory Abnormalities^a^	n (%)	n (%)
ALT elevations		
>ULN and <2 X ULN	6 (31·6)	9 (23·1)
≥2 X ULN and <3 X ULN	1 (5·3)	9 (23·1)
≥3 X ULN and <10 X ULN	0 (0)	11 (28·2)
≥10 X ULN	0 (0)	1 (2·6)
Proteinuria (dipstick)		
≥1+	3 (15·8)	12 (30·8)
≥2+	1 (5·3)	7 (17·9)
Serum creatinine		
1.3x > baseline	4 (21·1)	8 (20·5)
Women, n = 12, 21^b^ ≥0·2 mg/dL above baseline^c^	1 (8·3)	6 (28·6)

Abbreviations: ALT, alanine aminotransferase; ULN, upper limit of normal range.

a Not all laboratory abnormalities were reported as AEs.

b The numbers after ‘n  = ’ are the total numbers of male/female patients in the placebo group and in the mipomersen group, respectively.

c Percentages are calculated out of the total number of treated male or female patients, respectively, for the particular treatment group.

### Protocol Deviations

In this study 49 patients (35 mipomersen; 14 placebo); had at least 1 protocol deviation; not all protocol deviations resulted in exclusion from analysis. Sixteen patients (2 in the placebo group and 14 in the mipomersen group) were excluded from the per-protocol population: 7 (all in the mipomersen group) due to inadequate time on study drug; 6 (2 in the placebo group and 4 in the mipomersen group) due to a large LDL-C difference between screening and baseline indicating that LDL-C levels were not stable prior to treatment; and 5 (all in the mipomersen group) due to prescribed medication changes.

The study sponsor, with critical contributions from principal investigators, designed the trial in accordance with the principles of Good Clinical Practice. The sponsor was responsible for data collection and statistical analysis; authors were involved in analysis and interpretation. The corresponding author had full access to data and was responsible for manuscript preparation. **Trial Registration: clinicaltrials.gov**–NCT00794664. http://clinicaltrials.gov/ct2/show/NCT00794664.

**Table 5:** Liver Fat Content, Fasting Levels of Alanine Aminotransferase, and Percent Change in LDL-C in Patients with Post-Baseline For-Cause Magnetic Resonance Imaging Assessments.

Patient No.^a^	Liver Fat Content Spectral Model Fat Fraction (%)			Fasting ALT Levels (U/L)			LDL-C at Primary Efficacy Timepoint (% Change from Baseline)
	Baseline	MRI 2 (Days PostLast Dose)	MRI 3 (Days PostLast Dose)	Baseline	Week 21	Week 50	
1^b^	10·5	19·9 (2)	38·2 (186)	27	147	55	−54·3
2	17·6	38·1 (43)	NA	32	108	24	−89·5
3	0·8	23·0 (21)	1·0 (387)	22	117	25	−47·8
4	5·7	31·6 (14)	15·2 (239)	37	199	42	−49·9
5	7·2	46·7 (22)	12·7 (261)	30	177^c^	85	−77·4
6	−0·9	12·1 (32)	1·9 (291)	106	268^c^	33	−49·1
7	2·9	23·3 (−42)	0 (217)	44	114	35	−52·8

Abbreviations: LDL-C, low-density lipoprotein cholesterol; MRI, magnetic resonance imaging; NA, not available.

a To preserve confidentiality, the 7 patients are indicated by sequential counter numbers.

b MRI data for this patient were spleen-corrected due to violations of the MRI protocol.

c Week 13 result shown because week 21 results were not available.

## Results

Fifty-eight patients were randomized; 45 patients completed 26 weeks from January 27, 2009 to October 14, 2010 (Figure 1). The most common reason for discontinuation was adverse events (AEs). Most patients (79·3%) had a medical history of CHD or other clinical atherosclerotic disease; 40% had metabolic syndrome. Treatment groups were similar, except for tobacco and alcohol use (Table 1).

All 58 treated patients were evaluated for safety. One placebo patient had no post-baseline LDL-C measurements and so 57 patients were evaluated in the intent-to-treat population. Of these, 41 patients (25 mipomersen; 16 placebo) were evaluated in the per-protocol population.

Despite maximally tolerated lipid-lowering therapy, with 41% of patients at maximal statin dose) the mean baseline LDL-C and other lipid levels were elevated (Table 2). Lp(a), commonly elevated in FH, was also high. [18] Niacin was a concomitant medication in 2 (5.1%) mipomersen patients, and 1 (5.3%) placebo patient. The mean percent change in LDL-C of −36% (95% CI, –51·3, −15·3) in mipomersen patients was statistically significant (p<.001) compared to the mean percent change of 12·5% (95% CI, –10·8 to 35·8) in placebo patients (Figure 2). This corresponds to a mean absolute change of −2·62 mmol/L (from 7·15 mmol/L to 4·53 mmol/L) and 0·38 mmol/L (from 6·46 mmol/L to 6·84 mmol/L) in mipomersen and placebo patients, respectively. Changes in apolipoprotein B, TC, non-HDL-C, and Lp(a) mirrored these findings (Table 2, Figure 3). Sensitivity analyses on the primary endpoint produced similar results to the primary analysis.

A >15% decrease in LDL-C was seen in 79% of mipomersen patients compared with 17% of placebo patients. Moreover, 10 (25·6%) mipomersen patients had a >50% decrease in LDL-C, 6 (15·4%) achieved a target LDL-C level of <2·6 mmol/L, and 3 (7·7%) achieved LDL-C levels <1·8 mmol/L; no placebo patients experienced LDL-C reductions of this magnitude. There was a more robust LDL-C reduction in females than males (mean reduction 44% vs. 27%) in the mipomersen group; nonetheless, the 27% reduction in men was statistically significant and clinically meaningful.

Statins, ezetimibe, and bile acid sequestrants have limited impact on Lp(a). [9], [19] Mipomersen resulted in a 33% (95% CI, −43·3, −22·0) reduction in Lp(a) compared with a 1·5% (95% CI, –14·3, 11·3) reduction with placebo (Table 2, Figure 3).

At study end, 67% of placebo patients still met LDL-apheresis criteria versus 28% of mipomersen patients. [9], [17] Among patients with CHD or other atherosclerotic diseases at baseline, 79% of placebo patients and 32% of mipomersen patients still met LDL-apheresis criteria (LDL-C ≥5.1 mmol/L). Among patients without CHD or other atherosclerotic diseases at baseline, 12% of placebo patients and 25% of mipomersen patients still met LDL-apheresis criteria (LDL-C ≥7.8 mmol/L).

All mipomersen patients experienced at least one AE (Table 3). The most common AEs were mild-to-moderate injection site reactions (ISRs) representing 79% of mipomersen AEs (pain, 59%; erythema, 56%; pruritus, 33%) generally abating within 2 to 3 days. One patient had severe injection site erythema leading to early discontinuation. Mild or moderate flu-like symptoms (FLS) occurred in 46% of mipomersen and 21% of placebo patients. The onset of FLS was generally seen early in treatment; 1 patient withdrew after 1 dose of mipomersen due to this event. Repeated administration of mipomersen did not result in worsening of local ISRs or FLS, which did not occur with every injection.

During treatment 6 mipomersen and 0 placebo patients reported a serious AE (SAE); after treatment, 5 mipomersen and 1 placebo patient reported a SAE. Two patients experienced SAEs considered drug-related (ALT increased, AST increased, and hepatic steatosis in one patient and cerebrovascular accident, angina pectoris, with Prinzmetal angina, considered possibly drug-related, in another). Nine patients (8 from the mipomersen group) withdrew due to AEs. Although 6 patients withdrew from the mipomersen group for transaminase elevation, only two patients met protocol-defined stopping rules (1 patient with ALT >8 X ULN and 1 patient with ALT ≥3X ULN with the appearance/worsening of fatigue, aches, and right upper quadrant pain or tenderness. One patient withdrew due to moderate FLS and 1 patient due to severe ISR. One patient in the placebo group experienced headache, fatigue, and restless leg syndrome resulting in early termination. On-treatment cardiac events are shown in Table 3. Of these events 2 patients had events considered drug-related or possibly drug-related–Prinzmetal angina (1 patient with 2 events) and angina pectoris (2 patients with 3 events). Two patients had non-drug related events occurring in the follow-up period: one had supraventricular tachycardia the other acute myocardial infarction.

Table 4 lists laboratory abnormalities. The incidence of serum creatinine increases >1·3 X baseline were comparable between the treatment groups. In most cases (5/8 mipomersen; 3/4 placebo), elevations in serum creatinine did not occur in the same patients experiencing proteinuria. Beta-2 microglobulin assessed tubular effects; there was no notable difference between groups. Twelve patients (31%) in the mipomersen group and no placebo-treated patients had ALT and/or AST ≥3 X ULN. No patients had a significant increase in bilirubin or other abnormalities in liver function. For-cause post-baseline MRIs in 7 of the 12 mipomersen patients revealed increased liver fat content (Table 5); 3 had steatosis. Patients with increased liver fat also demonstrated reductions in LDL-C considerably larger than the group mean (range: −44.1% to −77.4%). Six of the 7 patients had a follow-up MRI after treatment ended; liver fat content fell significantly in 5 patients.

Although anti-mipomersen antibodies (titer  = 100 to 12,800) were detected in 14/39 mipomersen-treated patients, efficacy was not impacted (mean LDL-C reduction of 34%). One patient had 2+ proteinuria at screening and was discontinued due to AEs of nausea, ALT elevation, and liver tenderness. For-cause MRI also revealed increased liver fat (patient 4, Table 5). Mipomersen had no adverse impact on muscle function, platelet count, blood sugar, or blood pressure.

## Discussion

Clinical trials demonstrate significant reductions in both cardiac events and mortality with statins in high risk populations.[20]–[22] On a background of maximally tolerated lipid-lowering therapy, mipomersen 200 mg weekly lowered LDL-C an additional 36% (absolute mean reduction of 2.61 mmol/L) in patients with severe hypercholesterolemia. Females had a greater LDL-C lowering compared with males however, both groups had statistically significant and clinically meaningful reductions. Significant reductions in apolipoprotein B, non-HDL-C, and Lp(a) also occurred.

If sustained, LDL-C reductions of this magnitude (absolute mean reduction of 2·6 mmol/L) may reduce cardiac morbidity and mortality. A meta-analysis of statin trials suggested that for each 1 mmol/L reduction in LDL-C there is a corresponding 21% to 27% reduction in major vascular events, a 19% to 28% reduction in CHD-mortality, and a 12% to 16% reduction in all-cause mortality. [22], [23] Unlike statins, mipomersen also reduced Lp(a), a known independent predictor of CHD risk, and may result in even greater cardiovascular risk reduction than predicted from the aforementioned trials. [8], [9], [18], [24], [25].

The most common AEs with a higher incidence in the mipomersen group included ISRs and FLS. While neither is life-threatening, both can influence a patient’s willingness to remain on treatment. A large, currently enrolling clinical trial (NCT01475825) is exploring lower, more frequent dosing as a means to minimize both ISR and FLS.

In addition to the liver, mipomersen localized in the kidneys. [26] An increase in proteinuria and serum creatinine was noted in some mipomersen treated patients. Although the patients experiencing an increase in creatinine were not typically the patients who developed proteinuria. Cardiac events occurred more frequently in the mipomersen group. Because several previous mipomersen studies did not note an increase in cardiac events, it is possible that this represents a chance occurrence. Larger, long-term studies are required to determine the significance of both kidney function changes and cardiac events. In this and previous studies mipomersen had no adverse effect on blood pressure. [8].

A critical long-term safety concern is the potential for hepatic steatosis due to the accumulation of TG following inhibition of Apo B synthesis. It is reassuring that persons with familial hypobetalipoproteinemia, a genetic disorder characterized by reduced Apo B synthesis and life-long depressed LDL-C, enjoy a reduced risk of cardiac disease and a concomitant increase in lifespan despite significant hepatic fat. [27], [28] Although all commonly used lipid-lowering agents can increase ALT and AST, these agents rarely cause clinically significant liver injury. [29] Following discontinuation of mipomersen, ALT levels and hepatic fat reverted toward baseline.

Study limitations include the small study size, short-term treatment period, and liver imaging applied only for cause. This small cohort may not be sufficient to adequately evaluate safety concerns. An open-label extension trial (Clinicaltrials.gov: NCT00694109; n = 141) to evaluate the long-term safety of mipomersen is ongoing. Studies with alternate mipomersen regimens are also currently underway (NCT: 01475825, 00794664, 00694109, 00770146, 00706849). It is hoped that alternative dosing schedules will minimize side effects.

Mipomersen significantly lowers LDL-C and all measured Apo B-containing lipoproteins, including Lp(a) in a population at high risk for developing cardiac disease. These results confirm the benefits and consistent safety profile seen with mipomersen in various patient populations from previous trials [8], [9], [24], [25],[30],[31]. This study supports the use of mipomersen as a potential therapeutic option for patients with severe hypercholesterolemia not adequately controlled on currently available lipid-lowering medications. Future trials with larger patient numbers will help establish the utility of mipomersen as a potential therapeutic option for the treatment of patients with severe hypercholesterolemia.

## Supporting Information

Checklist S1 **CONSORT Checklist.**
(DOC)

Protocol S1 **Trial Protocol.**
(PDF)

## References

[1] National Lipid Association Expert panel on Familial Hypercholesterolemia (2011) Familial hypercholesterolemias: Prevalence, genetics, diagnosis and screening recommendations from the National Lipid Association Expert panel on Familial Hypercholesterolemia. J Clin Lipidol 5: S9–S17. 21600530 10.1016/j.jacl.2011.03.452

[2] Civeira F, for the International Panel on Management of Familial Hypercholesterolemia (2004) Guidelines for the diagnosis and management of heterozygous familial hypercholesterolemia. Atherosclerosis 173(1): 55–68. 15177124 10.1016/j.atherosclerosis.2003.11.010

[3] Avis HJ, Visser MN, Stein EA, Wijburg FA, Trip MD, et al (2007) A systematic review and meta-analysis of statin therapy in children with familial hypercholesterolemia. Arterioscler Thromb Vasc Biol 27(8): 1803–1810. 17569881 10.1161/ATVBAHA.107.145151

[4] Rodenburg J, Vissers MN, Wiegman A, van Trotsenburg AS, van der Graaf A, et al (2007) Statin treatment in children with familial hypercholesterolemia: the younger, the better. Circulation 116(6): 664–668. 17664376 10.1161/CIRCULATIONAHA.106.671016

[5] Marais AD (2004) Familial hypercholesterolaemia. Clin Biochem Rev 25(1): 49–68. 18516203 PMC1853359

[6] Hudgins LC, Gordon BR, Parker TS, Saal SD, Levine DM, et al (2002) LDL Apheresis: an effective and safe treatment for refractory hypercholesterolemia. Cardiovasc Drug Rev 20(4): 271–280. 12481200 10.1111/j.1527-3466.2002.tb00097.x

[7] Moriarty PM, Gibson CA, Flechsenhar K (2007) Familial hypercholesterolemia and lipid apheresis. In: Davidson MH, Toth PP, Maki KC, eds. Contemporary Cardiology: Therapeutic Lipidology. Totowa, NJ: Humana Press; 267–289p.

[8] Raal FJ, Santos RD, Blom DJ, Marais AD, Charng MJ, et al (2010) Mipomersen, an apolipoprotein B synthesis inhibitor, for lowering of LDL cholesterol concentrations in patients with homozygous familial hypercholesterolaemia: a randomised, double-blind, placebo-controlled trial. Lancet 375(9719): 998–1006. 20227758 10.1016/S0140-6736(10)60284-X

[9] Stein EA, Dufour R, Gagne C, Gaudet D, East C, et al.. (2010) Abstract: 5036 A randomized, double-blind, placebo-controlled study to assess efficacy and safety of mipomersen as add-on therapy in heterozygous familial hypercholesterolemia patients with coronary artery disease. Eur Heart J 31(Abstract Suppl): 898. 10.1161/CIRCULATIONAHA.112.104125 23060426

[10] Eckstein F (2007) The versatility of oligonucleotides as potential therapeutics. Expert Opin Biol Ther 7(7): 1021–1034. 17665991 10.1517/14712598.7.7.1021

[11] National Cholesterol Education Program. (NCEP) Expert Panel on Detection, Evaluation, and Treatment of High Blood Cholesterol in Adults (Adult Treatment Panel III) (2002) Third Report of the National Cholesterol Education Program (NCEP) Expert Panel on Detection, Evaluation, and Treatment of High Blood Cholesterol in Adults (Adult Treatment Panel III) final report. Circulation. 106(25): 3143–3421. 12485966

[12] National Institute for Health and Clinical Excellence. (2008) Identification and management of familial hypercholesterolaemia. NICE clinical guideline 71. Available: http://www.nice.org.uk. Accessed 2012 Mar 20.

[13] Thompson GR (2008) HEART-UK LDL Apheresis Working Group (2008) Recommendations for the use of LDL Apheresis. Atherosclerosis 198: 247–255. 18371971 10.1016/j.atherosclerosis.2008.02.009

[14] Graham I, Atar D, Borch-Johnsen K, Boysen G, Burell G, et al (2007) European Society of Cardiology (ESC) Committee for Practice Guidelines (CPG). European guidelines on cardiovascular disease prevention in clinical practice: executive summary: Fourth Joint Task Force of the European Society of Cardiology and Other Societies on Cardiovascular Disease Prevention in Clinical Practice (Constituted by representatives of nine societies and by invited experts). Eur Heart J 28(19): 2375–2414. 17726041 10.1093/eurheartj/ehm316

[15] Yokoo T, Bydder M, Hamilton G, Middleton MS, Gamst AC, et al (2009) Nonalcoholic fatty liver disease: diagnostic and fat-grading accuracy of low-flip-angle multiecho gradient-recalled-echo MR imaging at 1.5T. Radiology 251(1): 67–76. 19221054 10.1148/radiol.2511080666 PMC2663579

[16] Marcovina SM, Albers JJ, Henderson LO, Hannon WH (1993) International Federation of Clinical Chemistry standardization project for measurements of apolipoproteins A-I and B. III. Comparability of apolipoprotein A-I values by use of international reference material. Clin Chem 39(5): 773–781. 8485867

[17] Marcovina SM, Albers JJ, Kennedy H, Mei JV, Henderson LO, et al (1994) International Federation of Clinical Chemistry standardization project for measurements of apolipoproteins A-I and B. IV. Comparability of apolipoprotein B values by use of international reference material. Clin Chem 40(4): 586–592. 8149615

[18] Nordestgaard BG, Chapman MJ, Ray K, Borén J, Andreotti F, et al (2010) European Atherosclerosis Society Consensus Panel. European Atherosclerosis Society Consensus Panel. Lipoprotein(a) as a cardiovascular risk factor: current status. Eur Heart J 31(23): 2844–2853. 20965889 10.1093/eurheartj/ehq386 PMC3295201

[19] McKenney JM, Jones PH, Bays HE, Knopp RH, Kashyap ML, et al (2007) Comparative effects on lipid levels of combination therapy with a statin and extended-release niacin or ezetimibe versus a statin alone (the COMPELL study). Atherosclerosis 192(2): 432–437. 17239888 10.1016/j.atherosclerosis.2006.11.037

[20] Cholesterol Treatment Trialists’ (CTT) Collaboration, Baigent C, Blackwell L, Emberson J, Holland LE, et al (2010) Efficacy and safety of more intensive lowering of LDL cholesterol: a meta-analysis of data from 170,000 participants in 26 randomised trials. Lancet 376(9753): 1670–1681. 21067804 10.1016/S0140-6736(10)61350-5 PMC2988224

[21] Preiss D, Sattar N (2009) Lipids, lipid modifying agents and cardiovascular risk: a review of the evidence. Clin Endocrinol (Oxf) 70(6): 815–828. 19067719 10.1111/j.1365-2265.2008.03490.x

[22] Gould AL, Davies GM, Alemao E, Yin DD, Cook JR (2007) Cholesterol reduction yields clinical benefits: meta-analysis including recent trials. Clin Ther 29(5): 778–794. 17697899 10.1016/j.clinthera.2007.05.012

[23] Baigent C, Keech A, Kearney PM, Blackwell L, Buck G, et al (2005) Cholesterol Treatment Trialists' (CTT) Collaborators. Cholesterol Treatment Trialists’ (CTT) Collaborators. Efficacy and safety of cholesterol-lowering treatment: a prospective meta-analysis of data from 90,056 participants in 14 randomised trials of statins. Lancet 366(9493): 1267–1278. 16214597 10.1016/S0140-6736(05)67394-1

[24] Akdim F, Stroes ES, Sijbrands EJ, Tribble DL, Trip MD, et al (2010) Efficacy and safety of mipomersen, an antisense inhibitor of apolipoprotein B, in hypercholesterolemic subjects receiving stable statin therapy. J Am Coll Cardiol 55(15): 1611–1618. 20378080 10.1016/j.jacc.2009.11.069

[25] Akdim F, Visser ME, Tribble DL, Baker BF, Stroes ES, et al (2010) Effect of mipomersen, an apolipoprotein B synthesis inhibitor, on low-density lipoprotein cholesterol in patients with familial hypercholesterolemia. Am J Cardiol 105(10): 1413–1419. 20451687 10.1016/j.amjcard.2010.01.003

[26] Geary RS (2009) Antisense oligonucleotide pharmacokinetics and metabolism. Expert Opin Drug Metab Toxicol 5(4): 381–391. 19379126 10.1517/17425250902877680

[27] Glueck CJ, Gartside P, Fallat RW, Sielski J, Steiner PM (1976) Longevity syndromes: Familial hypobeta and familial hyperalpha lipoproteinemia. J Lab Clin Med 88(6): 941–957. 186545

[28] Sankatsing RR, Fouchier SW, de Haan S, Hutten BA, de Groot E, et al (2005) Hepatic and cardiovascular consequences of familial hypobetalipoproteinemia. Arterioscler Thromb Vasc Biol 25(9): 1979–1984. 16002743 10.1161/01.ATV.0000176191.64314.07

[29] Bhardwaj SS, Chalasani N (2007) Lipid-lowering agents that cause drug-induced hepatotoxicity. Clin Liver Dis 11(3): 597–613. 17723922 10.1016/j.cld.2007.06.010 PMC2048990

[30] Akdim F, Tribble DL, Flaim JD, Yu R, Su J, et al (2011) Efficacy of apolipoprotein B synthesis inhibition in subjects with mild-to-moderate hyperlipidaemia. Eur Heart J 32(21): 2650–2659. 21593041 10.1093/eurheartj/ehr148

[31] Visser ME, Wagener G, Baker BF, Geary RS, Donovan JM, et al (2012) Mipomersen, an apolipoprotein B synthesis inhibitor, lowers low-density lipoprotein cholesterol in high-risk statin-intolerant patients: a randomized, double-blind, placebo-controlled trial. Eur Heart J 33(9): 1142–1149. 22507979 10.1093/eurheartj/ehs023 PMC3751967

