# Supplementary material for: Randomized, Placebo-Controlled Trial of Mipomersen in Patients with Severe Hypercholesterolemia Receiving Maximally Tolerated Lipid-Lowering Therapy
Source: PLoS One. 2012 Nov 13;7(11):e49006. doi: 10.1371/journal.pone.0049006 (PMC3496741; doi:10.1371/journal.pone.0049006)
Supplement: Protocol S1 — Trial Protocol. (PDF) [file pone.0049006.s002.pdf]

16 Appendices

16.1 Study Information

16.1.1 Protocol and Protocol Amendments

Summary of Changes, Amendment 2 2
Amendment 2 (22 May 2009).....8
Summary of Changes, Amendment 1 84
Amendment 1 (29 October 2008) 88
Original Protocol (8 October 2008) 162

SUMMARY OF CHANGES
MIPO3500108 AMENDMENT 2

Protocol Title:	A Prospective Randomized, Double-Blind, Placebo-Controlled Study to Assess the Safety and Efficacy of Mipomersen in Patients with Severe Hypercholesterolemia on a Maximally Tolerated Lipid-Lowering Regimen and who are not on Apheresis		
Protocol Number:<br>EudraCT:	MIPO3500108<br>2008-006020-53	Orig. Version Date:	FINAL: 8 October 2008
Amendment Number:	1	Version Date:	FINAL: 29 October 2008
Amendment Number:	2	Version Date:	FINAL: 22 May 2009
Author Name:	Robin Morey		
Author Signature:	Not required		

RATIONALE FOR CHANGE(S):
Protocol MIPO3500108 Amendment 1 has been modified to allow inclusion of patients with other atherosclerotic diseases (peripheral arterial disease [PAD], symptomatic carotid artery disease, or abdominal aortic aneurysm [AAA]) who meet similar low density lipoprotein cholesterol (LDL-C) entry criteria as those with coronary heart disease (CHD). In the National Cholesterol Education Program [NCEP] Adult Treatment Panel III [ATP III] guidelines, these patients are noted to have the same or higher risk for cardiovascular events as patients with clinical CHD.

In addition to inclusion of patients with CHD or other clinical atherosclerotic diseases (inclusion criterion #3), minor changes were made in other inclusion/exclusion criteria, involving duration of contraceptive use (inclusion criterion #6), anti-arrhythmic treatment (exclusion criterion #1b), active infection requiring systemic antimicrobial therapy (exclusion #1f), use of a contraceptive patch (exclusion criterion #3c), and use of anti-obesity medications (exclusion #4e). The treatment visit window was increased from ± 2 to ± 3 days. Baseline magnetic resonance imaging (MRI) or computed tomography (CT) should be collected where possible and when not contraindicated. Medications should be taken as prescribed, per Investigator judgment, even when fasting blood samples are planned to be drawn. The safety reporting information was updated to add details on Medical Events of Interest (MEOI).

In addition, minor changes have been made to the text related to safety monitoring and stopping rule guidance and restriction on lifestyle of patients to increase the clarity of the protocol without impacting study intent or conduct.

Additional rationale for all changes is provided below.

Protocol Section	Changed Text and Rationale
Cover page	Updated key sponsor contact information
Protocol Synopsis and Abbreviations	Updated to reflect overall changes in protocol
Global Change	Allowed the inclusion of patients with other clinical atherosclerotic disease (PAD, symptomatic carotid artery disease, and AAA) and LDL-C of at least 200 mg/dL (5.1 mmol/L) as this group of patients has the same or higher risk of cardiovascular events as those with clinical CHD according to NCEP ATPIII guidelines
	Clarified that approximately 75 patients may be enrolled in the study to allow minimal over-enrollment
	For safety purposes, clarified that dosing should be stopped permanently in the event of a single alanine aminotransferase (ALT) or aspartate aminotransferase (AST) laboratory result ≥ 8 x upper limit or normal (ULN) without requiring a confirmatory measure
4 Introduction	In second paragraph, added abbreviations and removed metabolic syndrome as a CHD-risk equivalent condition as this was in error:<br>▪ CHD-risk equivalent conditions include diabetes, peripheral ~~vascular~~ artery disease (PAD), abdominal aortic aneurysm (AAA), symptomatic carotid artery disease, ~~metabolic syndrome,~~ and multiple risk factors that confer a 10-year risk for CHD events > 20%.
6.3 Discussion of Study Design Including Choice of Control Group	In first paragraph, amended the text to clarify that there are 26 weeks of treatment and 24 weeks of safety follow-up as this was an error:<br>▪ This is a randomized, double-blind, placebo-controlled, parallel-group study with ~~24~~ 26 weeks of treatment and ~~6 months~~ 24 weeks of safety follow-up.
7.1 Inclusion Criteria	To criterion #2, moved the reference to LDL-C values to criterion #3 for clarity:<br>▪ Fasting ~~LDL-C ≥ 200 mg/dL (5.1 mmol/L) and~~ TG < 350 mg/dL (4.0 mmol/L) at Screening
	To criterion #3, added the reference to inclusionary LDL-C values at Screening and amended text to align with the inclusion of patients with other clinical atherosclerotic disease and high risk of cardiovascular events:<br>▪ Fasting LDL-C ≥ 200 mg/dL (5.1 mmol/L) at Screening and the ~~P~~ presence of at least 1 of the following criteria ~~for coronary disease~~:<br>a) Myocardial infarction (MI)<br>b) Percutaneous coronary intervention (PCI) or coronary artery bypass graft (CABG)<br>c) Coronary artery disease (CAD) documented by angiography or any other accepted imaging technique<br>d) ~~If 1 or more of criteria a through c are not met: a p~~ Positive exercise test (≥ 1 mm ST-depression at maximal exercise or test terminated because of angina) or a perfusion defect, e.g., thallium or single photon emission computed tomography (SPECT)<br>e) Other clinical atherosclerotic diseases: peripheral artery disease (PAD), symptomatic carotid artery disease, abdominal aortic aneurysm (AAA)<br>f) Or, if a) through ~~d~~e) are not met, fasting LDL-C ≥ 300 mg/dL (7.8 mmol/L)
	To criterion #6, added a reference to section 9.2.6.8 which details acceptable and highly effective contraceptive methods. Clarified that contraceptive use must be utilized for 6 months after the last study drug dose:<br>▪ 6. Satisfy 1 of the following (see section 9.2.6.8):<br>a) Females: Non-pregnant and non-lactating; surgically sterile, postmenopausal, or patient or partner compliant with an acceptable and highly effective contraceptive regimen for 4 weeks prior to Screening, during the treatment

Protocol Section	Changed Text and Rationale
	phase, and 24 weeks 6 months after the last study drug dose<br>b) Males: Surgically sterile or patient or partner is utilizing an acceptable and highly effective contraceptive method during the treatment phase and 24 weeks 6 months after the last study drug dose
7.2 Exclusion Criteria	To criterion #1b, clarified that anti-arrhythmic treatment should be stable at time of screening/study entry and to allow enrollment of patients in whom arrhythmia is controlled:<br>▪ Presence of a clinically significant arrhythmias, deemed to be uncontrolled at any time < 12 months from screening or if medication for an arrhythmia has been started or dose has changed < 12 months from screening. Patients with implantable pacemakers or automatic implantable cardioverter defibrillators (AICDs), or currently taking any medication for arrhythmias may be considered if deemed to be stable for the previous 12 months by the Investigator
	To criterion #1f, clarified that patients can not be treated with systemic antimicrobial therapy for active infection unless treatment is expected to be completed prior to Day 1. This is because treated acute infections such as bronchitis or a UTI should not impact the conduct of the trial:<br>▪ Active infection requiring systemic antimicrobial therapy unless treatment is expected to be completed prior to Day 1
	To criterion #3b, removed anti-obesity medications as restricted medications within 12 weeks of Screening. Added anti-obesity medications as criterion #4e to stipulate that they are exclusionary unless at a stable dose regimen for at least 12 weeks prior to Screening and if the dose and regimen are expected to remain stable until Week 28.<br>▪ b) Anti-obesity medications<br>▪ e) Anti-obesity medications
	To criterion #3c, added contraceptive patch as an exclusionary medication since it is contraindicated in this study population of patients with high risk for cardiovascular events<br>▪ dc) Oral contraceptives or contraceptive patch
8.1 Treatments Administered	Changed references of "medial thigh" to "thigh" since specification of the medial thigh for dosing is not necessary. Injections can be administered anywhere in the thigh region.
	To the last paragraph, added text related to the dosing schedule to allow limited missed doses and scheduled visits due to sickness and holidays and reduce violations that are not expected to alter safety or efficacy of a drug with the PK characteristics of mipomersen:<br>▪ Due to reasons such as sickness, up to 3 doses of medication can be administered outside the dosing windows during the course of the study. Patients can make up for a missed dose by receiving 2 doses of study drug in the week before or the week after the missed dose(s) provided that the 2 doses are given at least 3 days apart.<br>▪ Up to a total of 3 doses will be allowed to be missed during the course of the study but P patients should be strongly discouraged from missing more than 2 scheduled doses of study drug in the first 13 weeks of dosing, more than 3 scheduled doses in the 26-week treatment period, and more than 1 of the last 4 scheduled doses of study drug.<br>▪ If the Investigator or the study coordinator is aware that a patient will be missing more than the recommended number of scheduled visits during the dosing period, the patient should be excluded from the study. However due to reasons such as sickness or holidays, up to 2 scheduled visits can be missed or occur outside of the visit window during the course of the study as long as they are not at the following critical time points: Baseline, Week 13, Week 28 and Week 50.

Protocol Section	Changed Text and Rationale
9.1.1.2 Clinical Laboratory Evaluations and Therapeutic Lifestyle Changes<br>9.2.6.8 Restriction on the Lifestyle of Patients	Amended text to clarify patients will be counseled on the TLC approach throughout the course of the study and not just during the treatment period:<br>▪ The patient will also be counseled on the TLC approach (Appendix 14.1); this will include advice on diet that should be maintained throughout the ~~treatment period~~ course of the study.
9.1.2 Treatment Period Assessments<br>Table 9-1 Schedule of Study Events	Changed the treatment period visit window from ±2 to ±3 days, to reduce violations and allow for holiday weekends, etc:<br>▪ There is a visit window of ± ~~2~~ 3 days through Week 26.
9.1.2.1 Magnetic Resonance Imaging<br>Table 9-1 Schedule of Study Events	Clarified that baseline MRI or CT should be collected when possible and not contraindicated. Confirmed the conditions under which rescreened patients need not repeat the MRI for clarification.<br>▪ Where possible and when not contraindicated, baseline MRI or CT of the liver and spleen ~~will~~ should be collected for all patients prior to administering the first dose of study drug. A CT scan may be performed in lieu of both the baseline and follow-up MRI for patients in whom an MRI is contraindicated. Please note that rescreened patients need not repeat the MRI if one was acquired during the original screening period, within the previous 6 weeks, and after consultation with the Medical Monitor.
9.1.2.3 Vital Signs, Body Weight, and Anthropometric Measurements<br>9.2.6.8 Restriction on the Lifestyle of Patients<br>Table 9-1 Schedule of Study Events	Added sentence regarding continued use of medications, per Investigator judgment, for patient safety purposes:<br>▪ Any medication should be taken as prescribed, per Investigator judgment, even when fasting blood samples are planned to be drawn.
9.2.4.2.1 Reporting to Genzyme	Updated the name of the SAE reporting form to include collection of an MEOI:<br>▪ For all SAEs, a Serious Adverse Event and Medical Event of Interest Form (SAE/MEOI form) ~~SAE Report Form~~ that includes a detailed written description, copies of relevant anonymized patient records, and other documents will be sent to Genzyme Pharmacovigilance within 24 hours.
9.2.4.2.2 Medical Events of Interest	Added section on MEOI to allow collection and monitoring of noteworthy events that are serious or non-serious:<br>▪ **9.2.4.2.2. Medical Events of Interest**<br>An MEOI is a noteworthy event for the particular product or class of products that a Sponsor may wish to carefully monitor. It could be a serious or non-serious event. Note: An MEOI is not an SAE unless it meets the SAE criteria as defined in Section 9.2.2. **Events to be reported as MEOIs for mipomersen protocols:**<br>• Discontinuations due to AEs<br>• Discontinuations due to *confirmed/presumed confirmed* stopping rule criteria<br>• Incidents of patients that reach *confirmed/presumed confirmed* laboratory safety monitoring rule criteria<br>An SAE/MEOI form should be completed and a copy faxed to Genzyme Pharmacovigilance (See Study Contact List).

Protocol Section	Changed Text and Rationale
9.2.6.1 Confirmation Guidance	Clarified that the results from a laboratory retest should be available prior to the next scheduled dose of study drug.<br>▪ If the initial laboratory result is observed during the treatment period, the results from the retest ~~must~~ should be available prior to the next scheduled dose of study drug (mipomersen or placebo).
9.2.6.2 Stopping Rule Guidance	Amended the sentence regarding continued dosing to clarify that the Investigator has the discretion to continue dosing patients who do not meet stopping rules after retest:<br>▪ In general, patients who do not meet the stopping rules based upon retest may continue dosing at the discretion of the Investigator.
9.2.6.3.1 Stopping Rules for Liver Chemistry Elevations	Added text to request discussion of laboratory results that may meet stopping rule criteria with the Medical Monitor:<br>▪ In the event of confirmed or presumed confirmed laboratory results meeting the following criteria, and the event is without an alternative explanation as discussed with the Medical Monitor (e.g., concomitant therapy with anticoagulants), dosing of a patient with study drug (mipomersen or placebo) will be stopped permanently.
	Under second bullet, deleted sentence on continued dosing as it conflicts with the confirmation guidance to withhold dosing until the lab is rechecked:<br>▪ ~~Treatment with study drug [mipomersen or placebo] will continue weekly until the second consecutive weekly ALT or AST measurement is confirmed or presumed confirmed to be ≥ 5 x ULN)~~
9.2.6.3.2 Safety Monitoring Rules for Liver Chemistry Tests	Amended text to allow clinical judgment by the Investigator about when patients are considered stabilized based on weekly monitoring, to establish the minimum duration of biweekly monitoring, and to allow discussion with the Medical Monitor about patients that have ALT/AST levels that fluctuate around the value of 3 x ULN:<br>▪ Frequency of Repeat Measurements: Patients with confirmed or presumed confirmed ALT or AST levels ≥ 3 x ULN should have their liver chemistry tests (ALT, AST, alkaline phosphatase, and total bilirubin at a minimum) retested at least once weekly until levels stabilize ~~or begin to recover~~ in the judgment of the Investigator. The performance of additional laboratory tests should be discussed with the Medical Monitor. Thereafter, liver chemistry tests for these patients should be performed at least every 2 weeks for the first 6 weeks that these patients have met this safety monitoring rule, or until ALT and AST are both < 3 x ULN. If ALT or AST levels increase to ≥ 3 x ULN after reducing below 3 x ULN, the monitoring plan should be discussed with the Medical Monitor and individualized for the patient. Subsequently, liver chemistry tests for these patients should be performed at least once a month until their ALT and AST levels become ≤ 1.2 x ULN (or ≤ 1.2 x Screening value for patients who enter the study with ALT and/or AST > ULN).
9.2.6.7 Safety Monitoring Rule for Constitutional Symptoms	Amended text to allow for investigation of constitutional symptoms of potentially related adverse events:<br>▪ Patients will be instructed to report any ~~signs or~~ constitutional symptoms, such as ~~of~~ fever, ~~or flu-like symptoms~~ that may arise ~~within the first 24 hours~~ after an injection is given. ~~and t~~The Investigator should closely evaluate all potential causes of ~~the fever~~ these symptoms, including concomitant illness.
9.2.6.8 Restriction on the Lifestyle of Patients	In paragraph three, aligned text with other ongoing mipomersen clinical protocols and added text to patients to exceed the maximum consumption of alcohol by a small amount up to 2 times to reduce violations:<br>▪ Patients should be willing to limit alcohol consumption for the entire duration of the study, including follow-up: male patients to a maximum of 2 drinks (20 g) per day, and 8 drinks (80 g) per week; female patients to a maximum of 1 drink (10 g) per day, and 4 drinks (40 g) per week. Up to 2 episodes of exceeding this limit by a small amount may be allowed during the course of the study.

Protocol Section	Changed Text and Rationale
	In paragraph 4, amended text to be consistent with the changes made to inclusion #6. Clarified that contraceptive patches are exclusionary methods of contraceptives and removed stipulation of prospective discussion with the Medical Monitor:<br>▪ ~~Male patients and f~~ Female patients of childbearing potential must continue to use appropriate contraception with their partners, or refrain from sexual activity, ~~from the time of~~ for 4 weeks prior to Screening and throughout study participation until ~~24 weeks~~ 6 months after the last study drug dose ~~of mipomersen~~. Male patients must use an acceptable contraceptive method during the treatment phase and until 6 months after the last study drug dose. Oral contraceptives and contraceptive patches are contraindicated in female patients enrolled in this study, but are an acceptable method of contraception for female partners of enrolled patients. Other acceptable methods of contraception are condoms with contraceptive foam, ~~oral contraceptives that have been prospectively discussed with the Medical Monitor,~~ implantable or injectable contraceptives, ~~contraceptive patch,~~ intrauterine device, diaphragm with spermicidal gel, or sexual partner who is surgically sterilized or post-menopausal.

This protocol amendment was designed and will be conducted, recorded, and reported in compliance with the principles of Good Clinical Practice (GCP) guidelines. These guidelines are stated in U.S. federal regulations as well as “Guidance for Good Clinical Practice,” International Conference on Harmonisation of Technical Requirements for Registration of Pharmaceuticals for Human Use.

I have read and agree to abide by the requirements of this amended protocol.

Investigator Signature Date

PHASE 3 CLINICAL STUDY PROTOCOL

A Prospective Randomized, Double-Blind, Placebo-Controlled Study to Assess the Safety and Efficacy of Mipomersen in Patients with Severe Hypercholesterolemia on a Maximally Tolerated Lipid-Lowering Regimen and who are not on Apheresis

Protocol Number: MIPO3500108
EudraCT: 2008-006020-53

Protocol Amendment 2: 22 May 2009
Protocol Amendment 1: 29 October 2008
Original Protocol: 8 October 2008

**Sponsor:**	Genzyme Europe BV<br>Gooimeer 10<br>1411 DD Naarden<br>The Netherlands	Genzyme Corporation<br>500 Kendall Street<br>Cambridge, MA 02142<br>USA
**Medical Monitor/ Key Study Contacts:**	Ajay Duggal, MBChB, MRCP, DipPharmMed<br>Senior Medical Director<br>Genzyme Europe B.V.<br>Gooimeer 10<br>1411 DD Naarden<br>The Netherlands<br>e-mail:<br>Ajay.Duggal@Genzyme.com<br>tel: +31 (0)35 699 8874<br>fax: +31 (0)35 699 1402	Joanne M. Donovan, MD, PhD<br>Vice President, Clinical Research<br>Genzyme Corporation<br>USA<br>e-mail:<br>Joanne.DonovanMD@Genzyme.com
**Statistician:**	Scott Chasan-Taber, PhD<br>Senior Director, Biostatistics<br>Genzyme Corporation<br>USA	

This protocol was designed and will be conducted, recorded, and reported in compliance with the principles of Good Clinical Practice (GCP) guidelines as well as in accordance with all national, state and local laws of the appropriate regulatory authorities. These guidelines are stated in "Guidance for Good Clinical Practice," International Conference on Harmonisation of Technical Requirements for Registration of Pharmaceuticals for Human Use.

I have read and agree to abide by the requirements of this protocol.

Investigator printed name …………………………………………

Investigator Signature______________________________________Date

Signature Page for Sponsor's Representative

The following have reviewed and approved the protocol entitled, "MIPO3500108: A Prospective Randomized, Double-Blind, Placebo-Controlled Study to Assess the Safety and Efficacy of Mipomersen in Patients with Severe Hypercholesterolemia on a Maximally Tolerated Lipid-Lowering Regimen and who are not on Apheresis."

Joanne Donovan 28 May 09

Joanne M. Donovan, MD, PhD
Vice President, Clinical Research
Genzyme Corporation
USA

Date

1 SYNOPSIS

**NAME OF COMPANY**<br>Genzyme Corporation<br>500 Kendall Street<br>Cambridge, MA 02142<br>USA<br><br>Genzyme Europe BV<br>Gooimeer 10<br>1411 DD Naarden<br>The Netherlands<br><br>**NAME OF FINISHED PRODUCT**<br>mipomersen sodium injection/ISIS 301012 Injection, 200 mg/mL, 1.0 mL<br>**NAME OF ACTIVE INGREDIENT**<br>mipomersen sodium/ISIS 301012 Drug Substance	**SUMMARY TABLE**<br>Referring to Part ……<br>of the Dossier:<br>Volume:<br>Page:<br>Reference:	**FOR NATIONAL AUTHORITY USE ONLY:**
**TITLE:**<br>A Prospective Randomized, Double-Blind, Placebo-Controlled Study to Assess the Safety and Efficacy of Mipomersen in Patients with Severe Hypercholesterolemia on a Maximally Tolerated Lipid-Lowering Regimen and who are not on Apheresis		
**PROTOCOL NO.:**<br>MIPO3500108		
**INVESTIGATOR STUDY CENTERS:**<br>Approximately 50 study centers will enroll patients in this protocol.		
**OBJECTIVES:**<br>To compare the safety and efficacy of 26 weekly, subcutaneous (SC) injections of mipomersen (200 mg) against placebo in treating severely hypercholesterolemic patients who are on a maximally tolerated lipid-lowering regimen and who are not on apheresis.		
**METHODOLOGY:**<br>This is a prospective, randomized, double-blind, placebo-controlled, parallel-group, multicenter Phase 3 study to investigate the safety and efficacy of mipomersen to treat severe hypercholesterolemia. Approximately 51 severely hypercholesterolemic adult patients will be randomized, who are on a maximally tolerated lipid-lowering regimen and not on apheresis, with either 1) low density lipoprotein cholesterol (LDL-C) ≥ 200 mg/dL (5.1 mmol/L) for patients with coronary heart disease (CHD) or other forms of clinical atherosclerotic disease, or 2) LDL-C ≥ 300 mg/dL (7.8 mmol/L). This represents a patient population that is considered to have the same or higher risk for cardiovascular events as patients in whom LDL-C apheresis is indicated in the United States of America (USA). Twenty-four (24) additional patients may be randomized to the study, for a total of approximately 75 patients, to provide up to 90% power for efficacy and to provide further safety information.<br><br>Patients will be randomized in a 2:1 ratio to receive mipomersen or placebo, respectively. Mipomersen or a matching volume of placebo will be administered weekly via SC injection for 26 doses.<br><br>Following the treatment period and Week 28 evaluation, patients may elect to continue in an open-label extension study, if one is available, pending approval by the Ethics Committee and the appropriate regulatory authority. Patients who choose not to participate or are ineligible for the open-label extension study will continue post-treatment follow-up in the current protocol.<br><br>The study will be conducted in compliance with International Conference on Harmonisation (ICH), Good Clinical Practice (GCP) guidelines as well as in accordance with all national, state and local laws		

**NAME OF COMPANY**<br>Genzyme Corporation<br>500 Kendall Street<br>Cambridge, MA 02142<br>USA<br><br>Genzyme Europe BV<br>Gooimeer 10<br>1411 DD Naarden<br>The Netherlands<br><br>**NAME OF FINISHED PRODUCT**<br>mipomersen sodium injection/ISIS 301012 Injection, 200 mg/mL, 1.0 mL<br>**NAME OF ACTIVE INGREDIENT**<br>mipomersen sodium/ISIS 301012 Drug Substance	**SUMMARY TABLE**<br>Referring to Part ……<br>of the Dossier:<br>Volume:<br>Page:<br>Reference:	**FOR NATIONAL AUTHORITY USE ONLY:**

of the appropriate regulatory authorities. The duration of the study, including completing patient enrollment, is expected to be approximately 18 months. Each patient will participate in a ≤ 4-week screening period and a 26-week treatment period. All patients, including those who discontinue prematurely and have received ≥ 1 dose of study drug, should be followed for safety for 24 weeks after their last dose of study drug as part of the post-treatment evaluation period. The end of study is defined as the last patient's last visit.

SAFETY MONITORING RULES:

An independent data monitoring committee (DMC) will provide an ongoing, expert, independent review of safety data to assure that the risks to study patients are minimized.

If any of the stopping criteria described in this protocol are met and are confirmed, the patient will be permanently discontinued from further treatment with study drug (mipomersen or placebo), evaluated fully in consultation with the Medical Monitor, and will be entered into the post-treatment evaluation portion of the study. In general, patients who do not meet the stopping rules based upon retest may continue dosing at the discretion of the Investigator. However, the Investigator and the Medical Monitor should confer as to whether additional close monitoring of the patient is appropriate.

NUMBER OF PATIENTS:
Between 51 and approximately 75 patients will be randomized into this study.

INCLUSION/EXCLUSION CRITERIA:
Inclusion Criteria:

Patients must fulfill all of the following criteria:

1. Age ≥ 18 years
2. Fasting triglycerides (TG) < 350 mg/dL (4.0 mmol/L) at Screening
3. Fasting LDL-C ≥ 200 mg/dL (5.1 mmol/L) at Screening and the presence of at least 1 of the following criteria:
   a) Myocardial infarction (MI)
   b) Percutaneous coronary intervention (PCI) or coronary artery bypass graft (CABG)
   c) Coronary artery disease (CAD) documented by angiography or any other accepted imaging technique
   d) Positive exercise test (≥ 1 mm ST-depression at maximal exercise or test terminated because of angina) or a perfusion defect, e.g., thallium or single photon

NAME OF COMPANY	SUMMARY TABLE	FOR NATIONAL AUTHORITY USE ONLY:
Genzyme Corporation<br>500 Kendall Street<br>Cambridge, MA 02142<br>USA<br><br>Genzyme Europe BV<br>Gooimeer 10<br>1411 DD Naarden<br>The Netherlands<br><br>**NAME OF FINISHED PRODUCT**<br>mipomersen sodium injection/ISIS 301012 Injection, 200 mg/mL, 1.0 mL<br>**NAME OF ACTIVE INGREDIENT**<br>mipomersen sodium/ISIS 301012 Drug Substance	Referring to Part ……<br>of the Dossier:<br>Volume:<br>Page:<br>Reference:	

emission computed tomography (SPECT)

e) Other clinical atherosclerotic diseases: peripheral artery disease (PAD), symptomatic carotid artery disease, abdominal aortic aneurysm (AAA)

f) Or, if a) through e) are not met, fasting LDL-C ≥ 300 mg/dL (7.8 mmol/L)

4. On a stable, maximally tolerated lipid-lowering regimen and expected to remain on it through Week 28 (must satisfy all criteria):

a) A statin at a maximally tolerated dose per Investigator judgment, for at least 8 weeks prior to Screening.

b) A stable low-fat diet (e.g., National Cholesterol Education Program [NCEP]-Adult Treatment Panel [ATP] III therapeutic lowering cholesterol [TLC] or equivalent) beginning at least 12 weeks prior to the first dose of study drug

c) A medication from an additional class of hypolipidemic agents, per Investigator's judgment (e.g., bile acid sequestrants, niacin/nicotinic acid, fibrates) for at least 8 weeks prior to Screening.

5. Body mass index (BMI) ≤ 40 kg/m^2 with weight stable (± 4 kg) for > 6 weeks prior to Screening per patient report.

6. Satisfy 1 of the following (see Section 9.2.6.8):

a) Females: Non-pregnant and non-lactating; surgically sterile, postmenopausal, or patient or partner compliant with an acceptable and highly effective contraceptive regimen for 4 weeks prior to Screening, during the treatment phase, and 6 months after the last study drug dose

b) Males: Surgically sterile or patient or partner is utilizing an acceptable and highly effective contraceptive method during the treatment phase and 6 months after the last study drug dose

7. Given informed consent

Exclusion Criteria:

1. Any of the following diagnoses, conditions, or prior treatments:

a) MI, PCI, CABG, cerebrovascular accident (CVA), unstable angina or acute coronary syndrome within 24 weeks of Screening

b) Presence of a clinically significant arrhythmia deemed to be uncontrolled at any time < 12 months from screening or if medication for an arrhythmia has been started or dose has changed < 12 months from screening. Patients with

NAME OF COMPANY<br>Genzyme Corporation<br>500 Kendall Street<br>Cambridge, MA 02142<br>USA<br><br>Genzyme Europe BV<br>Gooimeer 10<br>1411 DD Naarden<br>The Netherlands<br><br>**NAME OF FINISHED PRODUCT**<br>mipomersen sodium injection/ISIS 301012 Injection, 200 mg/mL, 1.0 mL<br>**NAME OF ACTIVE INGREDIENT**<br>mipomersen sodium/ISIS 301012 Drug Substance	**SUMMARY TABLE**<br>Referring to Part ……<br>of the Dossier:<br>Volume:<br>Page:<br>Reference:	**FOR NATIONAL AUTHORITY USE ONLY:**

implantable pacemakers or automatic implantable cardioverter defibrillators (AICDs) may be considered if deemed to be stable for the previous 12 months by the Investigator

c) Type 1 diabetes mellitus

d) New York Heart Association (NYHA) functional classification III or IV heart failure

e) Hypertension, systolic blood pressure (BP) ≥ 160 mmHg, or diastolic BP ≥ 95 mmHg at Screening (despite antihypertensive medication/therapy)

f) Active infection requiring systemic antimicrobial therapy unless treatment is expected to be completed prior to Day 1

g) Positive test for human immunodeficiency virus (HIV) or hepatitis B or C at Screening

h) Any uncontrolled condition that may predispose the patient to secondary hyperlipidemia such as uncontrolled hypothyroidism.

i) Malignancy within 5 years, except for basal or squamous cell carcinoma of the skin that has been adequately treated

j) Clinically significant hepatic or renal disease or Gilbert's syndrome

k) Apheresis within 3 months prior to Screening or expected to start apheresis during the treatment phase

2. The following laboratory values at Screening:

a) Serum creatine phosphokinase (CPK) ≥ 3 x upper limit of normal (ULN)

b) Alanine aminotransferase (ALT) levels > 1.5 x ULN

c) Serum creatinine > 0.1 mg/dL (> 8.8 µmol/L) above ULN for women, or > 0.2 mg/dL (> 17.7 µmol/L) above ULN for men

d) Proteinuria (> 1+ on dipstick, confirmed on retest, with further confirmation by quantitative total urine protein > 1.0 g/24 hr)

e) Total Bilirubin > 1.0 x ULN

f) Glycosylated hemoglobin A (HbA_{1C}) > 8.0%

3. Use of the following medications within 12 weeks of Screening:

a) Medications that may affect lipids except those allowed per the protocol, including but not limited to Cholestin™ (also known as red yeast rice, or monascus purpureus extract)

<table>
<tr>
<td>NAME OF COMPANY
Genzyme Corporation
500 Kendall Street
Cambridge, MA 02142
USA

Genzyme Europe BV
Gooimeer 10
1411 DD Naarden
The Netherlands

NAME OF FINISHED PRODUCT
mipomersen sodium injection/ISIS 301012 Injection, 200 mg/mL, 1.0 mL
NAME OF ACTIVE INGREDIENT
mipomersen sodium/ISIS 301012 Drug Substance</td>
<td>SUMMARY TABLE
Referring to Part ……
of the Dossier:
Volume:
Page:
Reference:</td>
<td>FOR NATIONAL AUTHORITY USE ONLY:</td>
</tr>
<tr>
<td colspan="3">
b) Chronic systemic corticosteroids or anabolic agents except for replacement therapy
c) Oral contraceptives or contraceptive patch
4. Use of the following medications unless a stable dose regimen was used for at least 12 weeks prior to Screening and the dose and regimen are expected to remain stable until Week 28:
a) Oral anticoagulants (e.g., warfarin)
b) Hormone replacement therapy
c) Diabetes medications including but not limited to sulfonylureas, metformin and glitazones, with the exception of changes of ± 10 units of insulin.
d) Antiviral therapy for herpes simplex virus (HSV)
e) Anti-obesity medications
5. Treatment with another investigational drug, biological agent, or device within 4 weeks of Screening or 5 half-lives of study agent, whichever is longer
6. Recent history of, or current, drug or alcohol abuse, or unwillingness to limit alcohol consumption for the entire duration of the study, including follow-up
7. Any disorders that would limit study participation or unwillingness to comply with study procedures, including follow-up, as specified by this protocol, or unwillingness to cooperate fully with the Investigator
8. Have any other medical conditions that, in the opinion of the Investigator, would make the patient unsuitable for enrollment, or could interfere with the patient participating in or completing the study
</td>
</tr>
<tr>
<td colspan="3">
DOSE/ROUTE/REGIMEN:
Patients will be randomized in a 2:1 ratio (i.e., mipomersen: placebo)

<u>Mipomersen</u>: 1-mL injections of 200 mg of mipomersen (ISIS 301012) will be administered weekly for 26 weeks.

Injections will be administered as SC injections into the outer area of the upper arm, thigh region, or abdomen. The first 3 doses must be supervised by a health professional, including study center personnel or home health nurse. Thereafter, patients will be given the option to self-administer study drug injections unsupervised with the exception of doses on clinic visit days.

A specific administration schedule will be used in this study in order to gain some understanding about the patient's preference of injection site and the reason for the choice. For the first 3 injections, the
</td>
</tr>
</table>

NAME OF COMPANY<br>Genzyme Corporation<br>500 Kendall Street<br>Cambridge, MA 02142<br>USA<br><br>Genzyme Europe BV<br>Gooimeer 10<br>1411 DD Naarden<br>The Netherlands<br><br>**NAME OF FINISHED PRODUCT**<br>mipomersen sodium injection/ISIS 301012 Injection, 200 mg/mL, 1.0 mL<br>**NAME OF ACTIVE INGREDIENT**<br>mipomersen sodium/ISIS 301012 Drug Substance	**SUMMARY TABLE**<br>Referring to Part ……<br>of the Dossier:<br>Volume:<br>Page:<br>Reference:	**FOR NATIONAL AUTHORITY USE ONLY:**
injection site will be specified for all patients. The first injection will be in the outer area of the upper arm, the second injection will be in the thigh region, and the third injection will be in the abdomen. Based on the patient's experience from the first 3 doses, each will be allowed to choose the injection site for their remaining injections.		
**REFERENCE TREATMENT:**<br>Adult hypercholesterolemic patients receiving either mipomersen or placebo as part of the study protocol will be on a maximally tolerated lipid lowering regimen.<br><br>Placebo: 1-mL weekly injections of placebo (i.e., vehicle consisting of 9 mg of sodium chloride, 0.004 mg of riboflavin, filled to (QS) 1 mL with water for injection) for 26 weeks.<br><br>Injections will be administered using the same instructions as provided for mipomersen.		
**CRITERIA FOR EVALUATION:**<br>Safety:<br><br>Safety will be evaluated in terms of all treatment-emergent adverse events (AEs) and serious adverse events (SAEs), physical examination findings, vital signs parameters and clinical laboratory parameters. AEs will be categorized using a standardized coding dictionary (e.g., Medical Dictionary for Regulatory Activities [MedDRA]). Clinical laboratory tests including chemistry, hematology with differential, and urinalysis will be tabulated.<br><br>Efficacy:<br><br>Efficacy will be assessed by measuring LDL-C, apolipoprotein (apo) B, and lipoprotein (a) (Lp[a]). The incremental effects of mipomersen on total cholesterol, TG, non-high density lipoprotein cholesterol (non-HDL-C), very low density lipoprotein cholesterol (VLDL-C), apo A-1, and lipoprotein subclasses may also be evaluated.		
**STATISTICAL METHODS:**<br>Primary Efficacy Endpoint<br><br>The primary efficacy endpoint is the percent change in LDL-C from Baseline to Week 28/Early Termination (ET represents the observation closest to 2 weeks after last dose among patients who early terminate study medication dosing).<br><br>Secondary Efficacy Endpoints<br><br>Secondary efficacy endpoints include the percent change in apoB, and Lp(a) from Baseline to		

NAME OF COMPANY<br>Genzyme Corporation<br>500 Kendall Street<br>Cambridge, MA 02142<br>USA<br><br>Genzyme Europe BV<br>Gooimeer 10<br>1411 DD Naarden<br>The Netherlands<br><br>**NAME OF FINISHED PRODUCT**<br>mipomersen sodium injection/ISIS 301012 Injection, 200 mg/mL, 1.0 mL<br>**NAME OF ACTIVE INGREDIENT**<br>mipomersen sodium/ISIS 301012 Drug Substance	**SUMMARY TABLE**<br>Referring to Part ……<br>of the Dossier:<br>Volume:<br>Page:<br>Reference:	**FOR NATIONAL AUTHORITY USE ONLY:**

Week 28/ET and the percent of patients with LDL-C below LDL-C entry criteria at Week 28/ET (200 mg/dL [5.1 mmol/L] for patients with coronary heart disease (CHD) or other clinical atherosclerotic disease, or 300 mg/dL [7.8 mmol/L]). The incremental effects of mipomersen on total cholesterol, TG, non-HDL-C, VLDL-C, apo A-1, and lipoprotein subclasses may also be evaluated.

Statistical Analyses

Efficacy Analyses:

The primary efficacy analysis will be performed on the Full Analysis Set, which represents the practically-feasible intent-to-treat (ITT) population as delineated in ICH Guideline E9, and will be comprised of all patients who receive at least 1 injection and have at least 1 post-Baseline LDL-C measurement.

Percent change from Baseline to Week 28/ET in LDL-C, apo B, and Lp(a) will be compared between treatment groups using a 2-sample t-test (if data departs substantially from normality, a Wilcoxon rank sum test will be used). The analysis will take place after all patients have completed Week 28 and the database has been locked. The percent of patients with LDL-C below LDL-C entry criteria at Week 28/ET will be compared between treatment groups using Fisher's exact test.

Safety Analyses:

The safety analyses will be performed on the Safety Set defined as all patients who receive at least 1 injection. Treatment duration and amount of study drug received will be summarized by treatment group, as will concomitant medications and treatments. Treatment-emergent AEs will be summarized by body system, preferred term, severity and relationship to the study procedures or treatments. Additionally listings of SAEs and AEs leading to discontinuation will be generated. Clinical laboratory evaluations, vital signs, electrocardiogram (ECG), and physical examination findings will be tabulated by treatment group. In addition, the number of patients who experience abnormalities in clinical laboratory evaluations will be summarized by treatment group.

Plasma concentrations and elimination half-life of mipomersen will be summarized over time by individual patient and by treatment group

Power and Sample Size

Based upon prior clinical trial experience with mipomersen, it is estimated that the standard deviation of the percent change in LDL-C is approximately 22%. With 45 randomized patients (15 patients in the control group and 30 patients in the mipomersen-treated group), there would be 80% power to detect a 20 percentage point difference between the 2 groups. Enrollment will be conducted such that at least 51 patients are randomized to allow for potential exclusions from an analysis set.

2 TABLE OF CONTENTS

1 SYNOPSIS 3
2 TABLE OF CONTENTS 10
3 ABBREVIATIONS AND TERMS 14
4 INTRODUCTION 17
4.1 ApoB-100 as a Therapeutic Target for Hypercholesterolemia 18
4.2 Mipomersen 19
4.2.1 Mechanism of Action 19
4.2.2 Chemistry 19
4.3 Summary of Benefits and Risks 20
5 STUDY OBJECTIVES 21
6 INVESTIGATIONAL PLAN 22
6.1 Study Design 22
6.2 Endpoints 24
6.2.1 Efficacy Endpoints 24
6.2.1.1 Primary Efficacy Endpoint 24
6.2.1.2 Secondary Efficacy Endpoints 24
6.2.2 Safety Endpoints 24
6.3 Discussion of Study Design Including Choice of Control Group 24
7 PATIENT POPULATION AND SELECTION 26
7.1 Inclusion Criteria 26
7.2 Exclusion Criteria 27
7.3 Patient Withdrawal 28
8 TREATMENTS 30
8.1 Treatments Administered 30
8.2 Investigational Product 31
8.2.1 Packaging and Labeling 31
8.2.2 Study Drug Preparation 31
8.2.3 Drug Storage 31
8.3 Prior and Concomitant Medications 31
8.4 Method of Assigning Patients to Treatment 32
8.5 Dose Selection 32
8.6 Blinding and Randomization 32
8.6.1 Unblinding 33
9 EFFICACY AND SAFETY VARIABLES 34
9.1 Efficacy and Safety Measurements Assessed and Study Flowchart 34
9.1.1 Screening Assessments 39
9.1.1.1 Medical History, Physical Examinations, 12-Lead Electrocardiogram 39
9.1.1.2 Clinical Laboratory Evaluations and Therapeutic Lifestyle Changes 39
9.1.2 Treatment Period Assessments 40
9.1.2.1 Magnetic Resonance Imaging and Computed Tomography 41
9.1.2.2 Clinical Laboratory Evaluations 41

9.1.2.3 Demographics, Vital Signs, Body Weight, and Anthropometric Measurements 44
9.1.2.4 Pharmacokinetic Trough Levels 44
9.1.2.5 Electrocardiogram 44
9.1.2.6 Physical Examination 45
9.1.2.7 Concomitant Medications 45
9.1.2.8 Injection Site Reaction Assessment 45
9.1.3 Post-Treatment Evaluation Period Assessments 46
9.2 Adverse Events 46
9.2.1 Definition of Adverse Events 46
9.2.2 Definition of Serious Adverse Events 46
9.2.3 Evaluation of Adverse Events and Serious Adverse Events 47
9.2.3.1 Severity of Adverse Events 47
9.2.3.2 Action Taken/Medication or Therapy Given 48
9.2.3.3 Outcome of Adverse Events 48
9.2.3.4 Relationship to Study Treatment 49
9.2.3.5 Clinical Laboratory Tests 49
9.2.3.6 Dosing Errors 50
9.2.4 Adverse Experience and Serious Adverse Experience Reporting 50
9.2.4.1 Adverse Experience Reporting 50
9.2.4.2 Serious Adverse Event Reporting 50
9.2.5 Pregnancy Reporting 52
9.2.6 Stopping Rules and Safety Monitoring 52
9.2.6.1 Confirmation Guidance 53
9.2.6.2 Stopping Rule Guidance 53
9.2.6.3 Liver Chemistry Rules 53
9.2.6.4 Safety Monitoring Rules for Renal Function Test Results 55
9.2.6.5 Safety Monitoring Rule for Platelet Count Results 55
9.2.6.6 Safety Monitoring Rules for Coagulation Parameters 56
9.2.6.7 Safety Monitoring Rule for Constitutional Symptoms 56
9.2.6.8 Restriction on the Lifestyle of Patients 56
9.2.7 Summary of Risks 57
10 DATA COLLECTION, QUALITY ASSURANCE, AND MANAGEMENT 57
10.1 Recording of Data 57
10.2 Data Quality Assurance 57
10.3 Data Management 57
11 STATISTICAL METHODS AND PLANNED ANALYSES 59
11.1 Power and Determination of Sample Size 59
11.2 Demographics and Baseline Characteristics 60

11.3 Patient Accountability ... 60
11.4 Study Treatment Usage and Compliance ... 60
11.5 Efficacy ... 60
11.5.1 Primary Efficacy Endpoint ... 60
11.5.2 Secondary Efficacy Endpoints ... 60
11.5.3 Efficacy Analysis ... 60
11.6 Safety ... 61
11.6.1 Safety Endpoints ... 61
11.6.2 Safety Analysis ... 61
11.7 Statistical Analysis Plan ... 61
12 SPECIAL REQUIREMENTS AND PROCEDURES ... 62
12.1 Institutional and Ethical Review ... 62
12.2 Changes to the Conduct of the Study or Protocol ... 62
12.3 Investigator's Responsibilities ... 62
12.3.1 Patient Informed Consent ... 62
12.3.2 Electronic Case Report Forms ... 63
12.3.3 Record Retention ... 63
12.3.4 Patient Discontinuation ... 64
12.3.5 Study or Site Termination ... 64
12.3.6 Monitoring ... 64
12.3.7 Materials Control ... 65
12.3.7.1 Receipt of Clinical Supplies ... 65
12.3.7.2 Disposition of Unused Clinical Supplies ... 65
12.3.8 Warnings, Precautions, Contraindications ... 65
12.3.9 Clinical Study Report ... 65
12.3.10 Disclosure of Data ... 65
13 REFERENCES ... 67
14 APPENDICES ... 69
14.1 NCEP – ATP III Therapeutic Lifestyle Changes ... 70
14.2 Lipoprotein Testing ... 71
14.3 Pregnancy Notification Forms ... 72

LIST OF IN-TEXT TABLES

Table 9-1 Schedule of Study Events ... 35
Table 9-2 List of Screening Clinical Laboratory Evaluations ... 40
Table 9-3 List of Treatment Period Clinical Laboratory Evaluations ... 43
Table 9-4 Contact List for Serious Adverse Event Reporting ... 51
Table 14-1 Nutrient Composition of the TLC Diet ... 70
Table 14-2 Lipoprotein Testing ... 71

LIST OF IN-TEXT FIGURES

Figure 4-1 Design of Chimeric 2´-MOE Phosphorothioate Oligonucleotides (MOE-Gapmer) 20

Figure 6-1 Study Design 24

3 ABBREVIATIONS AND TERMS

AAA	Abdominal aortic aneurysm
AE	Adverse event
AICD	Automatic implantable cardioverter defibrillator
ALT	Alanine aminotransferase (SGPT)
ANA	Antinuclear antibody
apo	Apolipoprotein
aPTT	Activated Partial Thromboplastin Time
ASO	Antisense oligonucleotide
AST	Aspartate aminotransferase (SGOT)
ATP	Adult Treatment Panel
BMI	Body Mass Index
BP	Blood pressure
BUN	Blood urea nitrogen
C3	Complement
CABG	Coronary artery bypass graft
CAD	Coronary artery disease
CHD	Coronary heart disease
C_{min}	Minimum plasma concentration
CMV	Cytomegalovirus
CPK	Creatine phosphokinase
eCRF	Electronic Case Report Form
CS	Clinically significant
CT	Computed tomography
CTTC	Cholesterol Treatment Trialists' Collaboration
CVA	Cerebrovascular accident
DMC	Data Monitoring Committee
DNA	Deoxyribonucleic acid
EBV	Epstein-Barr Virus
ECG	Electrocardiogram
EDC	Electronic data capture
ESR	Erythrocyte sedimentation rate
ET	Early termination
GCP	Good Clinical Practice
HAV	hepatitis A virus

HBsAg	Hepatitis B surface antigen
hCG	Human chorionic gonadotropin
HCV	hepatitis C virus
HDL-C	High density lipoprotein cholesterol
HEENT	Head, ears, eyes, nose, throat
HIV	Human immunodeficiency virus
HbA_{1c}	Glycosylated hemoglobin A
HMG-CoA	3-hydroxy-3-methylglutaryl coenzyme A
hsCRP	High-sensitivity C-reactive protein
HSV	Herpes simplex virus
ICH	International Conference on Harmonisation
IEC	Independent Ethics Committee
IgG	Immunoglobulin G
IgM	Immunoglobulin M
INR	International normalized ratio (for anticoagulant monitoring)
IRB	Institutional Review Board
ISR	Injection site reaction
ITT	Intent-to-treat
IVRS	Interactive voice response system
LDL	Low density lipoprotein
LDL-C	Low density lipoprotein cholesterol
LFT	Liver function test
Lp(a)	Lipoprotein a
MCV	Mean corpuscular volume
MedDRA	Medical Dictionary for Regulatory Activities
MEOI	Medical Event Of Interest
MI	Myocardial ~~I~~infarction
MOE	O-(2-Methoxyethyl)-D-Ribose
MRI	Magnetic resonance imaging
mRNA	Messenger Ribonucleic Acid
NCEP	National Cholesterol Education Program
NCS	Not Clinically Significant
NEPTUNE II	National Cholesterol Education (NCEP) Program Evaluation Project Utilizing Novel E-Technology II
NYHA	New York Heart Association
PAD	Peripheral artery disease

P/C	Urine protein to creatinine ratio
PK	Pharmacokinetic(s)
PT	Prothrombin Time
PCI	Percutaneous coronary intervention
PTT	Partial thromboplastin time
QA	Quality Assurance
QRS	Part of ECG wave representing ventricular depolarization
QS	A sufficient quantity to make
RR	Inter-beat interval
SAE	Serious Adverse Event
SC	Subcutaneous
SPECT	Single photon emission computed tomography
Study Drug	mipomersen or placebo
T_4	Thyroxine
TG	Triglycerides
TLC	Therapeutic Lifestyle Changes
TSH	Thyroid Stimulating Hormone
UAA	Translation stop codon
ULN	Upper Limit of Normal
USA	United States of America
VLDL	Very low density lipoprotein
VLDL-C	Very low density lipoprotein cholesterol
WBC	White blood cells
WHR	Waist-to-hip ratio

4 INTRODUCTION

Hypercholesterolemia is a common condition that in its untreated form is categorized by a broad range of elevated low density lipoprotein cholesterol (LDL-C) concentrations. Its etiology is mixed and although environmental contributors such as diet play a role, there is a strong genetic component especially in those patients who have LDL-C concentrations in the higher ranges. Monogenic forms of hypercholesterolemia include the different varieties of familial hypercholesterolemia, but there are also thought to be many polygenic contributors.

Elevated LDL-C is a major risk factor for coronary heart disease (CHD) and in the last years, results of observational and interventional studies in primary and secondary prevention indicate a continuous positive relationship between coronary artery disease (CAD) risk and blood cholesterol concentrations (National Cholesterol Education Program [NCEP] Adult Treatment Panel III [ATP III]) (Grundy, 2004, *Circulation*; NCEP, 2002, *Circulation)*. The strong association between LDL-C concentrations and CHD risk is attributed to the central role of LDL-C in the development and progression of atherosclerotic lesions, the underlying cause of CHD. Low density lipoprotein is a major source of lesion cholesterol and stimulates inflammatory processes involved in lesion development (Davis, 2001, *Arterioscler Thromb Vasc Bio.*; Hulthe, 2002, *Arterioscler Thromb Vasc Biol*; Skalen, 2002, *Nature*). In addition reducing LDL-C is associated with improved outcomes in many studies. A meta-analysis of many randomized controlled outcome studies indicated that a 1 mmol/L reduction in LDL-C resulted in a 23% reduction in vascular events across a range of end of treatment LDL-C concentrations (Cholesterol Treatment Trialists' Collaboration [CTTC], 2005, *Lancet*). Because of the increased risk of elevated LDL-C plasma concentrations, LDL-C reduction has been the principal goal of CHD prevention strategies (Knopp, 1999, *N Engl J Med*; Gotto, 2002, *Am J Med*). The goals for lipid-lowering therapy were established by the NCEP and were updated most recently in 2004 (Grundy, 2004, *Circulation*). The NCEP guidelines define 3 major categories of risk for clinical events and provide risk-based LDL-C targets. Those at highest risk are patients with CHD or CHD-risk equivalent conditions and should be treated to LDL-C targets < 100 mg/dL (<2.6 mmol/L) or lower (< 70 mg/dL [< 1.8 mmol/L]). CHD-risk equivalent conditions include diabetes, peripheral artery disease (PAD), abdominal aortic aneurysm (AAA), symptomatic carotid artery disease, and multiple risk factors that confer a 10-year risk for CHD events > 20%.

There are currently 5 classes of approved therapeutic agents for hyperlipidemia, all of which are administered orally. These include the bile-acid sequestrants, fibrates, niacin, statins, and cholesterol absorption inhibitors. The choice of therapy is individualized to accommodate the needs of the patient although the statins are the overwhelming therapy of choice due to the extent of LDL-C reductions achieved and the wealth of evidence of benefit. The statins

are competitive inhibitors of 3-hydroxy-3-methylglutaryl coenzyme A (HMG-CoA) reductase, the rate-limiting step in cholesterol biosynthesis. Administration of these drugs also leads to increased hepatic low density lipoprotein (LDL) receptor expression and enhanced cholesterol clearance. In addition, the statins appear to have pleiotropic effects, including anti-inflammatory, anti thrombotic, and anti-proliferative properties that may prevent plaque growth and rupture. Despite the availability and clinical effectiveness of these agents, recent data (Sueta, 2003, *J Cardiac Failure*) indicate that a significant proportion of patients do not attain target LDL-C values and thus that cardiovascular mortality rates remain high, especially in high-risk patients. Studies such as the NCEP Program Evaluation Project Utilizing Novel E-Technology II (NEPTUNE II) show that a large number of high-risk patients are not sufficiently treated. Of patients classified as very high risk, only 18% were at the optional therapeutic LDL-C goal of < 70 mg/dL (< 1.8 mmol/L) with 60% achieving concentrations < 100 mg/dL (2.6 mmol/L) (Davidson, 2005, *Am J Cardiol*). In individuals with CHD or CHD equivalent who did not meet their targets, average LDL-C concentrations were 125 mg/dL (3.2 mmol/L). More stringent LDL-C lowering goals, coupled with a clearer understanding of the molecular basis of various types of dyslipidemia, underscore the need for the development of new therapeutics.

4.1 ApoB-100 as a Therapeutic Target for Hypercholesterolemia

Apolipoproteins (apo) are a family of structural proteins present on the surface of lipoproteins that are important for the regulation of lipid transport and metabolism. They were named in an arbitrary alphabetical order and include apoA, B, C and E. ApoB exists in the plasma in 2 main isoforms, apoB-48 and apoB-100. In man, the apoB-48 is synthesized exclusively by the gut while apoB-100 is synthesized by the liver (Powell, 1987, *Cell*). The intestinal (B-48) and hepatic (B-100) forms of apoB are produced from a single gene and a single messenger ribonucleic acid (mRNA) transcript. This results from an organ-specific apoB mRNA editing enzyme (apobec-1), which generates a stop codon within the mRNA, changing CAA at position 2153 base pairs to a translation stop codon (UAA) (Chen, 1987, *Science*). Translation of the edited transcript yields a truncated protein product similar in composition to the N-terminal half of apoB 100. From structural studies, it is thought that apoB-48 represents the amino terminal 47% of apoB 100.

ApoB-100 is the major apolipoprotein of very low density lipoprotein (VLDL), intermediate-density lipoprotein, and LDL, comprising approximately 30%, 60%, and 95% of the protein in these lipoproteins, respectively. ApoB-100 is essential for the assembly and secretion of VLDL from the liver. Lipids such as triglycerides (TG) and cholesterol are packaged with apoB-100 and other phospholipids into VLDL, which in turn is secreted into the plasma, where additional apolipoproteins are added. Thus, inhibition of apoB-100 would be expected

to impair VLDL synthesis and result in lower concentrations of LDL-C. Indeed, individuals who are unable to translate full length apoB-100 have plasma concentrations of apoB and LDL-C that are 25% to 50% of normal (Sankatsing, 2005, *Arterioscler Thromb Vasc Biol*).

4.2 Mipomersen

4.2.1 Mechanism of Action

Antisense oligonucleotides (ASO) are designed to be complementary to a segment of a target mRNA. ASO binding to mRNA is dependent on Watson and Crick base-pairing rules and is, therefore, highly specific for the target sequence. Binding of the ASO to its cognate mRNA results in RNase H-mediated degradation of the cognate mRNA thus inhibiting mRNA translation into protein. First generation oligonucleotides are nearly identical to endogenous deoxyribonucleic acid (DNA), differing only in the replacement of sulfur for 1 of the non-bridging oxygens in each of the phosphate linkages of DNA to form phosphorothioate linkages. Phosphorothioate linkages are more resistant than phosphodiester linkages to nuclease degradation and thus make the ASOs more resistant to metabolism by nucleases. Second generation ASOs have the same phosphorothioate linkages, but in addition, the nucleotides are modified at the 2' position of the ribose with 2'-O-(2-methoxyethyl)-D-ribose (2'-MOE). The MOE modification (1) increases affinity towards the cognate mRNA, (2) increases resistance to exonucleases and endonucleases (thereby increasing tissue half-life), and (3) ameliorates some of the high-dose toxicities associated with first generation ASOs. Increased affinity results in potency that is increased compared to first-generation ASOs.

4.2.2 Chemistry

Mipomersen is an antisense drug targeted to human apoB-100, the principal apolipoprotein of atherogenic LDL and its metabolic precursor, VLDL. Mipomersen is complementary to the coding region of the mRNA for apoB-100, binding by Watson and Crick base pairing. The hybridization (binding) of mipomersen to the cognate mRNA results in RNase H-mediated degradation of the cognate mRNA thus inhibiting translation of the apoB-100 protein.

Mipomersen is the nonadecasodium salt of a 20-base (20-mer) phosphorothioate oligonucleotide. Each of the 19 internucleotide linkages is a 3´-O to 5´-O phosphorothioate diester. Ten of the 20 sugar residues are 2-deoxy-D-ribose, the remaining 10 are 2'-MOE. The 2´-MOE modification improves binding affinity for the target mRNA while increasing stability against nuclease-mediated degradation relative to 2´-deoxyribonucleosides. However, since the 2´ MOE modification reduces RNase H activity, a chimeric oligonucleotide strategy was employed in which 2´-deoxyribonucleosides that support RNase H activity are flanked by nuclease resistant 2´-MOE ribonucleosides. These modifications are commonly known as second-generation ASO, and the chimeric design of mipomersen improves its pharmacologic profile (relative to 2´ deoxyribonucleosides) while preserving

RNase H degradation of the target mRNA. Mipomersen is also referred to as a 5-10-5 MOE gapmer where the term gap refers to the ten 2′ deoxyribonucleosides that are necessary to support enzymatic cleavage of the cognate mRNA.

The sequence of mipomersen is shown (note that all cytosine residues are methylated at the 5-position).

Figure 4-1 Design of Chimeric 2′-MOE Phosphorothioate Oligonucleotides (MOE-Gapmer)

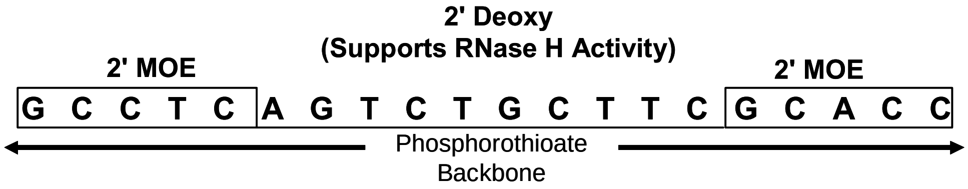

4.3 Summary of Benefits and Risks

Please refer to the Investigator's Brochure for additional information on clinical and nonclinical studies, and any known potential risks and benefits to humans.

5 STUDY OBJECTIVES

The objective of this study is to compare the safety and efficacy of 26 weekly, subcutaneous (SC) injections of mipomersen (200 mg) against placebo in treating severely hypercholesterolemic patients who are on a maximally tolerated lipid-lowering regimen and who are not on apheresis.

6 INVESTIGATIONAL PLAN

6.1 Study Design

This is a prospective, randomized, double-blind, placebo-controlled, parallel-group, multicenter Phase 3 study to investigate the safety and efficacy of mipomersen to treat severe hypercholesterolemia. Approximately 51 severely hypercholesterolemic adult patients will be randomized who are on a maximally tolerated lipid-lowering regimen and not on apheresis, with LDL-C of either 1) at least 200 mg/dL (5.1 mmol/L) for patients with CHD or other clinical atherosclerotic disease, or 2) at least 300 mg/dL (7.8 mmol/L). Patients with other clinical atherosclerotic disease (PAD, symptomatic carotid artery disease, and AAA) are considered to have the same or greater risk for cardiovascular events as patients with CHD according to NCEP ATPIII guidelines (NCEP, 2002, *Circulation*). Therefore, the patient population included in this study is considered to have a risk of CHD that is the same or greater than that of the patients in whom LDL-C apheresis is indicated in the United States of America (USA). Twenty-four (24) additional patients may be randomized to the study, for a total of approximately 75 patients, to provide up to 90% power for efficacy and to provide further safety information.

Patients will be randomized in a 2:1 ratio to receive mipomersen or placebo, respectively. Mipomersen or a matching volume of placebo will be administered weekly via SC injection for 26 doses.

Following the treatment period and Week 28 evaluation, patients may elect to continue in an open-label extension study, if one is available, pending approval by the Ethics Committee and the appropriate regulatory authority. Patients who choose not to participate or are ineligible for the open-label extension study will continue post-treatment follow-up in the current protocol.

The study will be conducted in compliance with International Conference on Harmonisation (ICH), Good Clinical Practice (GCP) guidelines as well as in accordance with all national, state and local laws of the appropriate regulatory authorities. The duration of the study, including completing patient enrollment, is expected to be approximately 18 months. Each patient will participate in a ≤ 4-week screening period and a 26-week treatment period. All patients, including those who discontinue prematurely and have received ≥ 1 dose of study drug, should be followed for safety for 24 weeks after their last dose of study drug as part of the post-treatment evaluation period (Figure 6-1).

An independent data monitoring committee (DMC) will provide an ongoing, expert, independent review of safety data to assure that the risks to study patients are minimized. This ongoing review will include pre-specified review of safety data during the conduct of

the study as defined by the DMC Charter. Based on these data, the DMC may recommend changes in study conduct to Genzyme. As part of the conduct of this study, Genzyme will periodically evaluate the nature, frequency and severity of AEs that have been identified as potential risks associated with the use of mipomersen or other new observations.

The end of study is defined as the last patient's last visit.

Figure 6-1 Study Design

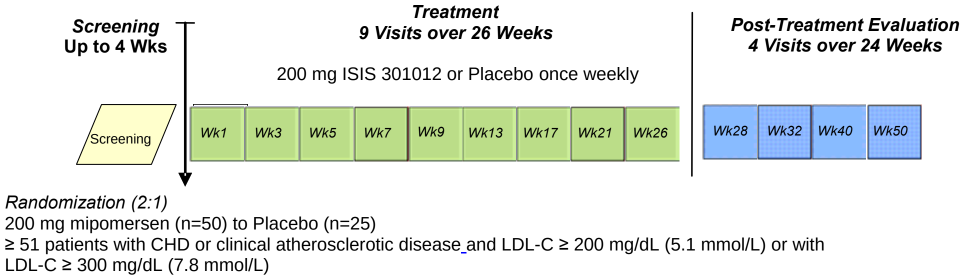

6.2 Endpoints

6.2.1 Efficacy Endpoints

6.2.1.1 Primary Efficacy Endpoint

The primary efficacy endpoint is the percent change in LDL-C from Baseline to Week 28/Early Termination (ET represents the observation closest to 2 weeks after last dose among patients who terminate study medication dosing early).

6.2.1.2 Secondary Efficacy Endpoints

Secondary efficacy endpoints include the percent change in apoB, and lipoprotein a (Lp[a]) from Baseline to Week 28/ET and the percent of patients with LDL-C below LDL-C entry criteria at Week 28/ET (200 mg/dL [5.1 mmol/L] for patients with coronary heart disease [CHD] or other clinical atherosclerotic disease, or 300 mg/dL [7.8 mmol/L]). The incremental effects of mipomersen on total cholesterol, TG, non-high density lipoprotein cholesterol (non-HDL-C), very low density lipoprotein cholesterol (VLDL-C), apo A-1, and lipoprotein subclasses may also be evaluated.

6.2.2 Safety Endpoints

Safety will be determined using the incidence of treatment-emergent adverse events (AEs), clinical laboratory evaluations, vital signs, electrocardiograms (ECGs), and physical examination findings. Adverse events will be categorized using the Medical Dictionary for Regulatory Activities (MedDRA). Plasma concentrations of mipomersen will be evaluated.

6.3 Discussion of Study Design Including Choice of Control Group

This is a randomized, double-blind, placebo-controlled, parallel-group study with 26 weeks of treatment and 24 weeks of safety follow-up. This treatment duration and follow-up has been chosen as it is roughly 5 times the terminal elimination half-life of mipomersen, so that

the efficacy endpoint can be evaluated when drug tissue levels are expected to be at > 90% of steady-state values and the safety assessments carried out when plasma trough levels are close to 0.

It is considered appropriate to treat the control group with placebo as these patients will also be receiving a maximally tolerated lipid-lowering regimen including statin (the gold standard of care), diet, and a medication from a second class of hyperlipidemic agents.

Based on nonclinical and clinical findings discussed in the Investigator's Brochure, close monitoring of safety laboratory parameters has been stipulated within the protocol particularly focusing on liver function tests (LFTs; also including imaging of the liver and spleen by magnetic resonance imaging [MRI]) or computed tomography (CT), and clotting parameters. The protocol also specifies under what conditions the implementation of clear stopping rules are to be employed to protect patients from unnecessary exposure to study medication. The LFT stopping rules are adapted from the draft guidance for industry, "Drug-Induced Liver Injury: Premarketing Clinical Evaluation," issued by the U.S. Department of Health and Human Services, Food and Drug Administration, October 2007.

Injection site reactions (ISRs) will be closely monitored. Inflammatory markers will be measured regularly.

Finally, the overall safety of the study will be monitored by an independent DMC.

7 PATIENT POPULATION AND SELECTION

Approximately 50 study centers will enroll approximately 75 patients in this protocol.

7.1 Inclusion Criteria

Patients must fulfill all of the following criteria:

1. Age ≥ 18 years
2. Fasting TG < 350 mg/dL (4.0 mmol/L) at Screening
3. Fasting LDL-C ≥ 200 mg/dL (5.1 mmol/L) at Screening and the presence of at least 1 of the following criteria:
   a) Myocardial infarction (MI)
   b) Percutaneous coronary intervention (PCI) or coronary artery bypass graft (CABG)
   c) CAD documented by angiography or any other accepted imaging technique
   d) Positive exercise test (≥ 1 mm ST-depression at maximal exercise or test terminated because of angina) or a perfusion defect, e.g., thallium or single photon emission computed tomography (SPECT)
   e) Other clinical atherosclerotic diseases: PAD, symptomatic carotid artery disease, AAA
   f) Or, if a) through e) are not met, fasting LDL-C ≥ 300 mg/dL (7.8 mmol/L)
4. On a stable, maximally tolerated lipid-lowering regimen and expected to remain on it through Week 28 (must satisfy all criteria):
   a) A statin at a maximally tolerated dose per Investigator judgment, for at least 8 weeks prior to Screening
   b) A stable low-fat diet (e.g., NCEP-ATP III therapeutic lowering cholesterol [TLC] or equivalent) beginning at least 12 weeks prior to the first dose of study drug
   c) A medication from an additional class of hypolipidemic agents, per Investigator's judgment (e.g., bile acid sequestrants, niacin/nicotinic acid, fibrates) for at least 8 weeks prior to Screening
5. Body mass index (BMI) ≤ 40 kg/m^2 with weight stable (± 4 kg) for > 6 weeks prior to Screening per patient report.
6. Satisfy 1 of the following (see Section 9.2.6.8):
   a) Females: Non-pregnant and non-lactating; surgically sterile, postmenopausal, or patient or partner compliant with an acceptable and highly effective contraceptive regimen for 4 weeks prior to Screening, during the treatment phase, and 6 months after the last study drug dose
   b) Males: Surgically sterile or patient or partner is utilizing an acceptable and highly effective contraceptive method during the treatment phase and 6 months after the last study drug dose

7. Given informed consent

7.2 Exclusion Criteria

Patients meeting any of the following criteria will be excluded from the study:

1. Any of the following diagnoses, conditions, or prior treatments:
    a) MI, PCI, CABG, cerebrovascular accident (CVA), unstable angina or acute coronary syndrome within 24 weeks of Screening
    b) Presence of a clinically significant arrhythmia deemed to be uncontrolled at any time < 12 months from screening or if medication for an arrhythmia has been started or dose has changed < 12 months from screening. Patients with implantable pacemakers or automatic implantable cardioverter defibrillators (AICDs) may be considered if deemed to be stable for the previous 12 months by the Investigator
    c) Type 1 diabetes mellitus
    d) New York Heart Association (NYHA) functional classification III or IV heart failure
    e) Hypertension, systolic blood pressure (BP) ≥ 160 mmHg, or diastolic BP ≥ 95 mmHg at Screening (despite antihypertensive medication/therapy)
    f) Active infection requiring systemic antimicrobial therapy unless treatment is expected to be completed prior to Day 1
    g) Positive test for human immunodeficiency virus (HIV) or hepatitis B or C at Screening
    h) Any uncontrolled condition that may predispose the patient to secondary hyperlipidemia such as uncontrolled hypothyroidism.
    i) Malignancy within 5 years, except for basal or squamous cell carcinoma of the skin that has been adequately treated
    j) Clinically significant hepatic or renal disease or Gilbert’s syndrome
    k) Apheresis within 3 months prior to Screening or expected to start apheresis during the treatment phase
2. The following laboratory values at Screening:
    a) Serum creatine phosphokinase (CPK) ≥ 3 x upper limit of normal (ULN)
    b) Alanine aminotransferase (ALT) levels > 1.5 x ULN
    c) Serum creatinine > 0.1 mg/dL (> 8.8 µmol/L) above ULN for women, or > 0.2 mg/dL (> 17.7 µmol/L) above ULN for men
    d) Proteinuria (> 1+ on dipstick, confirmed on retest, with further confirmation by quantitative total urine protein > 1.0 g/24 hr)
    e) Total Bilirubin > 1.0 x ULN
    f) Glycosylated hemoglobin A (HbA_{1C}) > 8.0%
3. Use of the following medications within 12 weeks of Screening:

a) Medications that may affect lipids except those allowed per the protocol, including but not limited to Cholestin™ (also known as red yeast rice, or monascus purpureus extract)

b) Chronic systemic corticosteroids or anabolic agents except for replacement therapy

c) Oral contraceptives or contraceptive patch

4. Use of the following medications unless a stable dose regimen was used for at least 12 weeks prior to Screening and the dose and regimen are expected to remain stable until Week 28:

   a) Oral anticoagulants (e.g., warfarin)

   b) Hormone replacement therapy

   c) Diabetes medications including but not limited to sulfonylureas, metformin and glitazones, with the exception of changes of ± 10 units of insulin.

   d) Antiviral therapy for herpes simplex virus (HSV)

   e) Anti-obesity medications

5. Treatment with another investigational drug, biological agent, or device within 4 weeks of Screening or 5 half-lives of study agent, whichever is longer

6. Recent history of, or current, drug or alcohol abuse, or unwillingness to limit alcohol consumption for the entire duration of the study, including follow-up

7. Any disorders that would limit study participation or unwillingness to comply with study procedures, including follow-up, as specified by this protocol, or unwillingness to cooperate fully with the Investigator

8. Have any other medical conditions that, in the opinion of the Investigator, would make the patient unsuitable for enrollment, or could interfere with the patient participating in or completing the study

7.3 Patient Withdrawal

Patients have the right to withdraw from the study at any time and for any reason without prejudice to their ongoing or future medical care.

Any patient who withdraws consent to participate in the study will be removed from further treatment and/or study observation immediately upon the date of request.

Should a patient (or a legally acceptable representative) request or decide to withdraw from the study, all efforts will be made to complete and report the observations as thoroughly as possible up to the date of withdrawal, and an Early Termination visit should be performed. All information should be reported on the applicable electronic Case Report Forms (eCRFs).

Patients who request to stop study drug or have been withdrawn from study treatment at the request of the Investigator or Sponsor before completion of the protocol-specified treatment period, and who have received at least 1 dose of study drug and have not revoked their consent to participate in the study, will be strongly encouraged to continue the post-treatment evaluation period beginning with the Week 28 visit assessments. This visit should be scheduled within 2 weeks of their last dose of study drug. For those patients who withdraw from the study prior to receiving any dose of study drug, no further follow up is necessary.

Patients who request to withdraw from the study during the post-treatment evaluation period should be encouraged to undergo clinical laboratory and safety evaluations including, but not limited to, hematology and chemistry panels, urinalysis, pregnancy test (for women of childbearing potential), vital signs collection and a physical examination prior to leaving the study (if it has been longer than 4 weeks since these procedures were last performed).

The Investigator must record the reason for withdrawal on the Study Termination eCRF.

If the patient withdraws due to an AE, the Investigator should arrange for the patient to have follow-up visits until the AE has resolved or stabilized.

Reasons for removal from study drug or observation may include:

- Withdrawal of consent
- Administrative decision by the Investigator or Sponsor
- Pregnancy (report on Pregnancy Notification Forms)
- Ineligibility
- Significant protocol deviation
- Patient non-compliance
- The patient experiences an AE that is considered intolerable by the patient or Investigator (includes disease progression; report on AE eCRF)

8 TREATMENTS

8.1 Treatments Administered

Patients will be randomized in a 2:1 ratio (i.e., mipomersen:placebo)

Mipomersen: 1-mL weekly injections of 200 mg of mipomersen will be administered for 26 weeks.

Placebo: 1-mL weekly injections of placebo (i.e., vehicle consisting of 9 mg of sodium chloride, 0.004 mg of riboflavin, filled to (QS) 1 mL with water for injection) for 26 weeks.

Injections will be administered as SC injections into the outer area of the upper arm, the thigh region, or the abdomen. The first 3 doses must be supervised by a health professional, including study center personnel or home health nurse. Thereafter, patients will be given the option to self-administer study drug injections unsupervised with the exception of doses on clinic visit days.

A specific administration schedule will be used in this study in order to gain some understanding about the patient's preference of injection site and the reason for the choice. For the first 3 injections, the injection site will be specified for all patients. The first injection will be in the outer area of the upper arm, the second injection will be in the thigh region, and the third injection will be in the abdomen. Based on the patient's experience from the first 3 doses, each will be allowed to choose the injection site for their remaining injections. The chosen site and the reason for the choice, (e.g., tolerability, convenience, physician recommendation, aesthetic, no reason, etc.) will be recorded on the eCRF. The patient may choose a different injection site at any time during the remainder of the treatment period. The new injection site and the reason for the change should be recorded on the eCRF. The patient's opinion on injection site location will also be collected at the conclusion of the treatment period.

The dose schedule may be adjusted in the event a patient is unable to come to the clinic due to vacations or holidays as follows:

- Due to reasons such as sickness, up to 3 doses of medication can be administered outside the dosing windows during the course of the study. Patients can make up for a missed dose by receiving 2 doses of study drug in the week before or the week after the missed dose(s) provided that the 2 doses are given at least 3 days apart.
- Up to a total of 3 doses will be allowed to be missed during the course of the study but patients should be strongly discouraged from missing more than 2 scheduled doses of study drug in the first 13 weeks of dosing, more than 3 scheduled doses in the 26-week treatment period, and more than 1 of the last 4 scheduled doses of study drug.
- If the Investigator or the study coordinator is aware that a patient will be missing more than the recommended number of scheduled visits during the dosing period, the patient

should be excluded from the study. However due to reasons such as sickness or holidays, up to 2 scheduled visits can be missed or occur outside of the visit window during the course of the study as long as they are not at the following critical time points: Week 1, Week 13, Week 28 and Week 50.

Dosing details must be recorded in the patient's source documents and the eCRF.

8.2 Investigational Product

Mipomersen is supplied as 200 mg/mL of mipomersen (ISIS 301012), with 1 mL of solution per vial.

Placebo is the vehicle, consisting of 9 mg of sodium chloride, 0.004 mg of riboflavin, QS to 1 mL with water for injection.

8.2.1 Packaging and Labeling

The study drug described in Section 8.2 is contained in 2-mL stoppered glass vials that will be provided to the study center by the Sponsor or designee. Mipomersen active pharmaceutical ingredient is manufactured by Isis Pharmaceuticals, Inc., Carlsbad, California, USA.

The Sponsor or designee will provide the Investigator with packaged study drug labeled in accordance with specific country regulatory requirements.

8.2.2 Study Drug Preparation

The study drug must be brought to room temperature prior to administration. Using aseptic technique, withdraw 1 mL from the vial of mipomersen (ISIS 301012) or placebo. Refer to the Dosing Instructions in the Investigational Product Handling Manual for additional details.

8.2.3 Drug Storage

The study drug must be stored securely at 2° to 8° Celsius and be protected from light in a limited access area.

8.3 Prior and Concomitant Medications

Reasonable efforts will be made to determine all medications and treatments (pharmacological and non-pharmacological) received by the patient. Prior medications or treatments are defined as any medication or therapy taken by the patient within 1 month prior to Screening. In addition, an attempt will be made to collect data on any lipid-lowering medications taken within 1 year prior to Screening. A medication or treatment is considered concomitant if it is taken at any time after the Screening visit up to and including the day of the final study evaluation. Data on medications will include: name, dose, route, regimen, start date, stop date, and indication. Data on treatments will include: treatment, start date, stop date, and indication. At each study visit, the patient will be asked about any additional

medications or treatments or any changes in regimen or dosages since the last visit. Indications for any new medications or treatments during the study period will be recorded as an AE.

All concomitant lipid-lowering drugs are to remain constant from Screening through Week 28 of the study. Investigators may prescribe concomitant medications or treatments deemed necessary to provide adequate supportive care, with the exception of those listed in Section 7.2 as exclusions.

All concomitant medications taken during the study must be recorded in the source documentation and in the eCRF.

8.4 Method of Assigning Patients to Treatment

Using an Interactive Voice Response System (IVRS), patients will be randomized to mipomersen or placebo in a 2:1 ratio, prior to study drug administration. Patient enrollment will be conducted such that at least 51 patients are randomized with LDL-C of 1) at least 200 mg/dL (5.1 mmol/L) for patients with CHD or other clinical atherosclerotic disease, or 2) at least 300 mg/dL (7.8 mmol/L). This represents a patient population that is considered to have the same risk or higher risk for cardiovascular events as patients in whom LDL-C apheresis is indicated in the USA. Approximately 24 more patients may be randomized to provide additional efficacy and safety information. The Sponsor's Quality Assurance (QA) & Compliance department or designee will hold a copy of the randomization schedule, generated by the Sponsor (or designee). All patients, Investigators, study staff, and the Sponsor will be blinded to the treatment assignment.

8.5 Dose Selection

The 200 mg/week dose was selected for this study because results from Phase 1 and Phase 2 clinical studies showed a satisfactory safety profile with a significant pharmacodynamic effect at this dose (see Investigator's Brochure). Due to the long terminal elimination half-life of mipomersen, the treatment duration is 26 weeks so that the efficacy endpoint can be evaluated when drug tissue levels are expected to be at > 90% of steady-state values.

The safety and tolerability of the proposed dosing regimen is also supported by nonclinical chronic toxicology studies (see Investigator's Brochure).

8.6 Blinding and Randomization

Patients, monitors, study center personnel, and the Sponsor will remain blinded to the patient's treatment assignment throughout the study. In addition, in order to ensure maintenance of the study blind, lipid data will be not be available to the patients, Investigators, study staff, or the Sponsor until the study has been unblinded, as knowledge of

these data could unblind these individuals to the treatment assignment and could influence patient assessment.

8.6.1 Unblinding

In the event of an emergency where the identity of the study drug must be known by the Investigator to provide appropriate medical treatment, the Investigator will be allowed to unblind using the IVRS. However, prior to this, the Investigator should make every effort to discuss the situation with the Medical Monitor.

In the event that an unblinding occurs, the Investigator will notify the Sponsor or designee of the unblinding within 24 hours of its occurrence. The treatment assignment should only be unblinded in the case of a serious adverse event (SAE; see Section 9.2.2), and when knowledge of the treatment assignment will impact the clinical management of the patient. Every reasonable attempt should be made to complete the End-of-Study (study termination) evaluation procedures prior to unblinding as knowledge of the treatment assignment could influence patient assessment.

9 EFFICACY AND SAFETY VARIABLES

9.1 Efficacy and Safety Measurements Assessed and Study Flowchart

The study for an individual patient will consist of the following periods:

- Up to a 4-week screening period
- A 26-week treatment period during which study drug will be administered (consisting of a SC dose once a week), and
- A 24-week post-treatment evaluation period

Table 9-1 summarizes the schedule of study events at each visit for patients enrolled.

Table 9-1 Schedule of Study Events

Study Period	Screen-ing	Treatment									Post-Treatment Evaluation			
Study Week	≤ 4 weeks	Wk 1	Wk 3	Wk 5	Wk 7	Wk 9	Wk 13	Wk 17	Wk 21	Wk 26	Wk 28	Wk 32	Wk 40	Wk 50
Study Day	-28 to -1	1	15	29	43	57	85	113	141	176	190	218	274	344
Visit[h]	-1	Visit 1	Visit 2	Visit 3	Visit 4	Visit 5	Visit 6	Visit 7	Visit 8	Visit 9	PTx Visit 1	PTx Visit 2	PTx Visit 3	PTx Visit 4
Visit Window		± 3 days									± 7 days			
Written Informed Consent	X													
Medical History	X													
Demographics and Baseline Characteristics	X	X												
Inclusion / Exclusion Criteria	X													
Physical Examination[a]	X			X		X	X				X			X
Abdominal & Hip Circumference	X										X			
Liver & Spleen MRI or CT		X[b]												
Vital Signs (+ body weight and height at Screening)	X	X	X	X	X	X	X	X	X	X	X	X	X	X
Urinalysis[c]	X[g]	X[d]	X	X	X	X	X[d]	X	X	X	X[d]	X	X	X
Pregnancy Test[e]	X	X		X		X	X	X	X	X		X	X	X
ISR Evaluation		X	X	X	X	X	X	X	X	X	X			
Weekly Study Drug[f] Administration		X	X	X	X	X	X	X	X	X				

Table 9-1 Schedule of Study Events (Continued)

Study Period	Screen -ing	Treatment									Post-Treatment Evaluation			
Study Week	≤ 4 weeks	Wk 1	Wk 3	Wk 5	Wk 7	Wk 9	Wk 13	Wk 17	Wk 21	Wk 26	Wk 28	Wk 32	Wk 40	Wk 50
Study Day	-28 to -1	1	15	29	43	57	85	113	141	176	190	218	274	344
Visit[h]	-1	Visit 1	Visit 2	Visit 3	Visit 4	Visit 5	Visit 6	Visit 7	Visit 8	Visit 9	PTx Visit 1	PTx Visit 2	PTx Visit 3	PTx Visit 4
Visit Window		± 3 days									± 7 days			
12-Lead ECG	X						X				X			X
AE Assessment	X	X	X	X	X	X	X	X	X	X	X	X	X	X
Concomitant Medications/Therapies	X	X	X	X	X	X	X	X	X	X	X	X	X	X

[a] Full physical examination at Screening must include height measurement. Physical examinations, including general appearance, skin, head, ears, eyes, nose, throat (HEENT), lymph nodes, heart, lungs, abdomen, extremities/joints, neurological, and mental status will be performed at Screening and during the treatment period. Additionally, the patients will be monitored for lymphadenopathy and splenomegaly.

[b] Where possible and when not contraindicated, baseline magnetic resonance imaging (MRI) or computed tomography (CT) should be done prior to administering the first dose of the study drug. Please note that rescreened patients need not repeat the MRI if one was acquired during the original screening period, within the previous 6 weeks, and after consultation with the Medical Monitor. Follow-up MRI or CT will be obtained if clinically indicated (i.e., for alanine aminotransferase [ALT] elevations ≥ 3 x upper limit of normal [ULN]) during the course of the study. Please refer to the Site Reference Binder for MRI and CT procedures and settings.

[c] Sample collected prior to study drug administration during treatment period

[d] Urinalysis on these days includes total protein, microalbumin, creatinine, urine protein to creatinine ratio (P/C ratio), and β2-microglobulin

[e] For females of childbearing potential only.

[f] The first 3 doses must be supervised by a health professional, including study center personnel or home health nurse. Thereafter, patients will be given the option to self-administer study drug injections unsupervised.

[g] At Screening, if urine dipstick is positive (> 1+) for microalbuminuria, then a 24-hour sample will be obtained to assess protein excretion. If total protein > 1g/24 hr then the patient will be excluded.

ISR = injection site reaction
ECG = electrocardiogram
AE=adverse event

Table 9-1 Schedule of Study Events (Continued)

Study Period	Screen-ing	Treatment									Post-Treatment Evaluation			
Study Week	≤ 4 weeks	Wk 1	Wk 3	Wk 5	Wk 7	Wk 9	Wk 13	Wk 17	Wk 21	Wk 26	Wk 28	Wk 32	Wk 40	Wk 50
Study Day	-28 to -1	1	15	29	43	57	85	113	141	176	190	218	274	344
Visit[h]	-1	Visit 1	Visit 2	Visit 3	Visit 4	Visit 5	Visit 6	Visit 7	Visit 8	Visit 9	PTx Visit 1	PTx Visit 2	PTx Visit 3	PTx Visit 4
Visit Window		± 3 days									± 7 days			
Chemistry Panel (+ CPK)	X	X	X	X	X	X	X	X	X	X	X	X	X	X
Full Serum Lipid Panel	X	X		X		X	X	X	X	X	X	X	X	X
HbA_{1C}[k]	X						X				X			X
Hematology (Complete Blood Count + Diff.)	X	X	X	X	X	X	X	X	X	X	X	X	X	X
Coagulation (aPTT, PT)		X					X				X			
Hepatitis B & C, HIV	X													
Thyroid function (T_4, TSH)	X													
Trough PK - ISIS 301012[j]		X	X	X	X	X	X	X	X	X	X	X	X	X
ISIS 301012 Antibodies[i]		X									X			X
IgG		X					X				X			
Cardiovascular Risk Markers (hsCRP and LDL subclass concentrations)	X	X					X			X	X			
Complement (C3)		X					X				X			

Table 9-1 Schedule of Study Events (Continued)

Study Period	Screen-ing	Treatment									Post-Treatment Evaluation			
Study Week	≤ 4 weeks	Wk 1	Wk 3	Wk 5	Wk 7	Wk 9	Wk 13	Wk 17	Wk 21	Wk 26	Wk 28	Wk 32	Wk 40	Wk 50
Study Day	-28 to -1	1	15	29	43	57	85	113	141	176	190	218	274	344
Visit[h]	-1	Visit 1	Visit 2	Visit 3	Visit 4	Visit 5	Visit 6	Visit 7	Visit 8	Visit 9	PTx Visit 1	PTx Visit 2	PTx Visit 3	PTx Visit 4
Visit Window		± 3 days[c]									± 7 days			
Erythrocyte Sedimentation Rate (ESR)		X					X				X			
Inflammatory Markers[l]		X					X				X			

h All visits are conducted after an overnight fast for > 10 hours (only water and medications per Investigator judgment permitted). Note: all labs must be performed prior to injection of study drug.

i Sample collected prior to study drug administration during treatment period

j The outpatient visits at Week 13 and Week 26 must be scheduled on a dosing day, and must occur 7 days after the previous dose. Blood samples for pharmacokinetic (PK) trough levels MUST be drawn prior to mipomersen administration at all outpatient visits during the treatment period.

k At Screening for all patients and diabetic patients only thereafter.

l In addition to the pre-specified inflammatory markers (i.e., immunoglobulin G [IgG], white blood cell [WBC] differentials, complement [C3], erythrocyte sedimentation rate [ESR], and high-sensitivity C-reactive protein [hsCRP]), a serum sample specifically designated for the measurement of inflammatory parameters will be collected pretreatment, during treatment (Week 13) and at the end of treatment (Week 28) and appropriately stored for subsequent analysis, as needed. This will allow for the measurement of other inflammatory markers should significant changes in the primary markers be noted or for further characterization of inflammation based on emerging markers.

Note: The laboratory test, hsCRP, is a marker of inflammation and, as such, may be elevated due to any condition that causes general inflammation including but not limited to recent or ongoing illness, viral, bacterial or fungal infection, tissue injury, trauma, bone fractures or exacerbation of chronic inflammatory conditions. Since this test is sensitive to these other conditions, samples for hsCRP evaluations should not be drawn from patients with acute inflammatory conditions or with chronic inflammatory conditions that have been acutely exacerbated until such conditions are clinically stable. In any event, tests should not be postponed for more than 4 weeks. Reasons for such postponing tests should be documented.

Note: All items on this page of the study schedule require blood draws (collected prior to study drug administration during the treatment period). CPK=Creatine phosphokinase, aPTT=activated partial thromboplastin time, PT=prothrombin time, T_4=thyroxine, TSH=thyroid stimulating hormone, HbA_{1c}=Glycosylated hemoglobin A, HIV=human immunodeficiency virus, LDL=low density lipoprotein.

9.1.1 Screening Assessments

The initial Screening visit will take place within a maximum of 4 weeks before the first dose of study drug is administered (on Day 1). Once written informed consent is obtained, a patient's eligibility for entry into the study will be assessed according to the inclusion and exclusion criteria.

9.1.1.1 Medical History, Physical Examinations, 12-Lead Electrocardiogram

Patients will give a full medical history, and undergo a full physical examination, including anthropometric and vital signs measurements and monitoring for lymphadenopathy and splenomegaly (Refer to Section 9.1.2.6). A 12-lead ECG tracing will be obtained.

9.1.1.2 Clinical Laboratory Evaluations and Therapeutic Lifestyle Changes

Blood and urine samples will be collected for clinical laboratory evaluations according to the schedule in Table 9-1 (see Table 9-2 for a complete list of analytes). The patient will also be counseled on the TLC approach (Appendix 14.1); this will include advice on diet that should be maintained throughout the course of the study. Lipoprotein testing information is in Appendix 14.2.

Table 9-2 List of Screening Clinical Laboratory Evaluations

**Screening Assessments**			
**Clinical Chemistry**	**Hematology**	**Urinalysis**	**Other**
Sodium<br>Potassium<br>Chloride<br>Bicarbonate<br>Total protein<br>Albumin<br>Total globulin<br>Calcium<br>Phosphorus<br>Glucose<br>Blood urea nitrogen (BUN)<br>Creatinine<br>Uric Acid<br>LDH<br>Total, Direct, and Indirect Bilirubin<br>Alkaline phosphatase<br>AST (SGOT)<br>ALT (SGPT)<br>CPK	Red blood cells<br>Hemoglobin<br>Hematocrit<br>MCV<br>Platelets<br>White blood cells (WBC)<br>WBC Differential (% and absolute)<br>• Neutrophils<br>• Eosinophils<br>• Basophils<br>• Lymphocytes<br>• Monocytes	Specific gravity<br>pH<br>Protein<br>Glucose<br>Ketones<br>Bilirubin<br>Blood<br>Red blood cells<br>WBC<br>Epithelial cells<br>Bacteria<br>Casts<br>Crystals	Thyroid Function<br>T_4<br>TSH<br><br>Hepatitis<br>Hepatitis B surface antigen<br>Hepatitis C antibody<br><br>HIV<br>HIV antibody<br><br>Pregnancy<br>hCG<br><br>Cardiovascular Risk Markers<br>hsCRP<br>LDL-subclass concentrations
		**Full Lipid Panel**	**Metabolic**
		ApoB<br>ApoA-1<br>Total cholesterol<br>LDL-C<br>HDL-C<br>VLDL-C<br>TG<br>Lp(a)	HbA_{1c}

BUN=blood urea nitrogen, LDH=low density lipoprotein, AST (SGOT)=aspartate aminotransferase, ALT (SGPT)=alanine aminotransferase, CPK=creatine phosphokinase, MCV=mean corpuscular volume, WBC=white blood cells, apo=apoliprotein, LDL-C=low density lipoprotein cholesterol, HDL-C=high density lipoprotein cholesterol, VLDL-C= very high density lipoprotein cholesterol, TG=triglycerides, Lp(a)=lipoprotein (a), T_4=thyroxine, TSH=thyroid-stimulating hormone, HIV=human immunodeficiency virus, hCG=human chorionic gonadotropin, hsCRP=high-sensitivity C-reactive protein, HbA_{1c}=glycosylated hemoglobin A.

9.1.2 Treatment Period Assessments

The study for an individual patient will consist of the following periods:

- Up to a 4-week screening period
- A 26-week treatment period during which study drug will be administered (consisting of a SC dose once a week), and

- A 24-week post-treatment evaluation period

There is a visit window of ± 3 days through Week 26. In the event that a visit does not occur on the scheduled date, all subsequent visits should be calculated based on the time elapsed since Day 1, rather than from the date of the last visit. The outpatient visits at Week 13 and Week 26 must be scheduled on a dosing day, and must occur 7 days after the previous dose.

9.1.2.1 Magnetic Resonance Imaging and Computed Tomography

Where possible and when not contraindicated, baseline MRI or CT of the liver and spleen will be collected for all patients prior to administering the first dose of study drug. A CT scan may be performed in lieu of both the baseline and follow-up MRI for patients in whom an MRI is contraindicated. Please note that rescreened patients need not repeat the MRI if one was acquired during the original screening period, within the previous 6 weeks, and after consultation with the Medical Monitor. The images will be stored for future analysis if required. Follow-up MRI (or CT) will be obtained if clinically indicated (i.e., for ALT elevations ≥ 3 x ULN) during the course of the study. Please refer to the Site Reference Binder for MRI (or CT) procedures and settings.

9.1.2.2 Clinical Laboratory Evaluations

The following clinical laboratory tests will be assessed by a central laboratory at the time points specified in Table 9-1.

A blood sample for baseline full serum lipid panel, hematology with differential, chemistry, cardiovascular risk markers (high-sensitivity C-reactive protein [hsCRP] and LDL subclass concentrations), immunoglobulin G (IgG), ISIS 301012 antibodies, erythrocyte sedimentation rate (ESR), complement (C3), and coagulation parameters (activated partial thromboplastin time [aPTT] and prothrombin time [PT]) will be drawn prior to study drug administration according to the schedule in Table 9-1. In addition to the pre-specified inflammatory markers (i.e., IgG, white blood cell (WBC) differentials, C3, ESR, and hsCRP), a serum sample specifically designated for the measurement of inflammatory parameters will be collected pretreatment, during treatment (Week 13) and at the end of treatment (Week 28) and appropriately stored for subsequent analysis, as needed. This will allow for the measurement of other inflammatory markers should significant changes in the primary markers be noted or for further characterization of inflammation based on emerging markers. Urinalysis will be performed at each visit during the treatment period. Pregnancy tests for women of childbearing potential will be performed monthly during study participation. Table 9-3 details clinical laboratory evaluations for the treatment period; Table 9-1 lists the visits at which samples will be drawn.

A central laboratory will provide collection supplies, arrange collection, and perform analysis of clinical laboratory evaluations indicated in Table 9-2 and Table 9-3. Using the central laboratory for unscheduled clinical laboratory evaluations and redraws is recommended. Procedures for the handling and shipment of all central laboratory samples will be included in the laboratory manual. Specimens will be appropriately processed by the central laboratory facility and laboratory reports will be made available to the Investigator in a timely manner to ensure appropriate clinical review.

The Investigator is responsible for reviewing and signing all laboratory reports. The clinical significance of each value outside of reference range will be assessed and documented as either not clinically significant (NCS) or clinically significant (CS). All CS values require a comment and the out-of-range value or the overlying diagnosis (or value if the diagnosis is unknown) must be captured as an AE.

Clinical significance is defined as any finding that results in an alteration in medical care (for definition of clinical significance, please refer to Section 9.2.3.5). The Investigator will continue to monitor the patient until the parameter returns to its baseline status or until agreement is reached between the Investigator and Sponsor.

Clinical laboratory reports will serve as source documents.

Table 9-3 List of Treatment Period Clinical Laboratory Evaluations

**Clinical Safety Assessments (Minimum Requirements)**		**Efficacy Assessments**	**Other Assessments**
**Clinical Chemistry**	**Urinalysis**	**Full Lipid Panel**	**Metabolic**
Sodium<br>Potassium<br>Chloride<br>Bicarbonate<br>Total protein<br>Albumin<br>Total globulin<br>Calcium<br>Phosphorus<br>Glucose<br>BUN<br>Creatinine<br>Uric Acid<br>LDH<br>Total, Direct, and Indirect Bilirubin<br>Alkaline phosphatase<br>AST (SGOT)<br>ALT (SGPT)<br>CPK	Specific gravity<br>pH<br>Protein<br>Glucose<br>Ketones<br>Bilirubin<br>Blood<br>Red blood cells<br>WBC<br>Epithelial cells<br>Bacteria<br>Casts<br>Crystals<br>Creatinine*<br>Total Protein*<br>Protein to creatinine (P/C) Ratio*<br>Microalbumin*<br>β2-microglobulin*	ApoB<br>ApoA-1<br>Total cholesterol<br>LDL-C<br>HDL-C<br>VLDL-C<br>TG<br>Lp(a)	HbA_{1c}<br>**Inflammatory Markers†**<br>To be determined
**Coagulation**	**Hematology**	**Cardiovascular Risk Markers**	**Immune Function**
aPTT<br>PT<br>INR<br>**Complement**<br>C3	Red blood cells<br>Hemoglobin<br>Hematocrit<br>MCV<br>Platelets<br>White blood cells<br>WBC Differential (% and absolute)<br>• Neutrophils<br>• Eosinophils<br>• Basophils<br>• Lymphocytes<br>• Monocytes	hsCRP<br>LDL subclass concentrations	IgG<br>ISIS 301012 antibodies<br>ESR<br>**Pharmacokinetic**<br>Trough ISIS 301012 level<br>**Pregnancy**<br>hCG[a]

*At select outpatient visits (Weeks 1, 13, and 28)
[a] For women of childbearing potential; see Table 9-1 for schedule of visits at which a pregnancy test is performed.
†In addition to the pre-specified inflammatory markers (i.e., IgG, WBC Differential, C3, ESR, and hsCRP), a serum sample specifically designated for the measurement of inflammatory parameters will be collected pretreatment, during treatment (Week 13) and at the end of treatment (Week 28) and appropriately stored for subsequent analysis, as needed.

9.1.2.3 Demographics, Vital Signs, Body Weight, and Anthropometric Measurements

Demographic data, height, and weight will be recorded at the Screening visit.

Vital signs (BP, heart rate, respiratory rate, and temperature) will be measured at the time points specified in Table 9-1. Semi-supine systolic and diastolic BP should always be measured on the same arm (preferentially on the left arm) with the patient being in a semi-supine position for at least 5 minutes. Any clinically significant abnormal findings will be recorded on the AE page of the eCRF. Any medication should be taken as prescribed, per Investigator judgment, even when fasting blood samples are planned to be drawn.

If a CS deterioration is noted, the changes will be documented as AEs on the eCRF. Clinical significance is defined as any finding that results in an alteration in medical care. The Investigator will continue to monitor the patient until the parameter returns to its baseline status or until agreement is reached between the Investigator and Sponsor.

Height and body weight should be measured without shoes. Body weight should be measured with the patient fully dressed in everyday clothes, but overcoats should be removed before weighing.

Waist circumference should be measured with a stretchless tape and patient in a standing position after normal expiration, midway between the caudal part of the lateral costal arch and the iliac crest. Hip circumference should be measured at the symphysis trochanter level. Calculations to be performed are BMI and waist-to-hip ratio (WHR).

9.1.2.4 Pharmacokinetic Trough Levels

At each outpatient visit to the study center, including Day 1, a blood sample will be drawn prior to study drug administration for plasma PK trough levels. Blood samples for PK trough levels MUST be drawn prior to study drug administration at all outpatient visits during the treatment period.

9.1.2.5 Electrocardiogram

The ECG will be recorded after the patient has been resting in a supine position for at least 5 minutes. Six limb leads, as specified by Einthoven (I, II and III) and Goldberger (aVR, aVL and aVF) and 6 precordial leads (V1-V6) according to Wilson will be used. Printouts of the recordings will be done with 25 mm/s time resolution at an amplification of 10 mm/mV. Electrocardiograms will be performed at Screening, and at Weeks 13, 28, and 50.

The ECG tracing should contain at least 4 QRS complexes (in order to obtain 3 RR intervals). Each ECG tracing should include the following information: identification of each lead, study number, study day number, patient number, paper speed, voltage calibration, and date and time of recording. After identification of each lead, the Investigator should evaluate, sign, and date the printout. The Investigator should state whether the ECG is normal or abnormal; if abnormal, whether the ECG tracing is clinically significant.

9.1.2.6 Physical Examination

A complete physical examination will be conducted at the time points specified in Table 9-1. The examination will include an assessment of the patient general appearance; skin; head, ears, eyes, nose throat (HEENT); examinations of lymph nodes; heart; lungs; abdomen; extremities/joints; and neurological and mental status. Additionally, the patients will be specifically monitored for lymphadenopathy and splenomegaly and any findings will be recorded. Whenever possible, the same physician should perform the examination at each study visit.

If a clinically significant deterioration is noted, the change will be documented as an AE on the eCRF. Clinical significance is defined as any finding that results in an alteration in medical care. The Investigator will continue to monitor the patient until the parameter returns to its baseline status or until agreement is reached between the Investigator and Sponsor.

9.1.2.7 Concomitant Medications

Concomitant medications are to be assessed at all visits per Table 9-1. Reasonable efforts will be made to determine all treatments (pharmacological and non-pharmacological) received by the patient. A therapy is considered concomitant if it is taken at any time after the Screening visit up to and including the day of the final study evaluation. Data on pharmacological and non-pharmacological treatments will be captured on separate eCRF pages, but both to include: name, dose, route, regimen, start date, stop date, and indication. At each study visit, the patient will be asked about any additional treatments or any changes in regimen or dosages since the last visit. Indications for any new medications or therapies during the study period will be recorded as an AE.

9.1.2.8 Injection Site Reaction Assessment

At each outpatient visit during the treatment period, an ISR assessment will be made for the current and previous injection sites. ISR assessments are recorded starting after the first injection is given.

9.1.3 Post-Treatment Evaluation Period Assessments

There is a visit window of ± 7 days throughout the post-treatment evaluation period. During this period, MRI or CT (if clinically indicated), clinical laboratory evaluations, vital signs, body weight, anthropometric measurements, plasma PK trough levels, ECG, and a full physical examination will be performed according to the schedule in Table 9-1, and as described in Section 9.1.2. Adverse events and changes in concomitant medication use will continue to be reported at each visit.

9.2 Adverse Events

9.2.1 Definition of Adverse Events

An AE is defined as any undesirable physical, psychological or behavioral effect experienced by a patient during their participation in an investigational study, in conjunction with the use of the drug, whether or not product-related. This includes any untoward signs or symptoms experienced by the patient from the time of signing the informed consent until completion of the study.

Adverse events may include, but are not limited to:

- Subjective or objective symptoms spontaneously offered by the patient and/or observed by the Investigator or medical staff
- Laboratory abnormalities of clinical significance

Disease signs, and symptoms and/or laboratory abnormalities already existing prior to the use of the product are not considered AEs after treatment unless they recur after the patient has recovered from the pre-existing condition or, in the opinion of the Investigator, they represent a clinically significant exacerbation in intensity or frequency.

9.2.2 Definition of Serious Adverse Events

A SAE is any AE that results in any of the following outcomes:

- Death
- Life-threatening experience
- Required or prolonged inpatient hospitalization
- Persistent or significant disability/incapacity
- Congenital Anomaly
- Important medical events that may jeopardize the patient or subject and may require medical or surgical intervention to prevent one of the outcomes listed above.

Death: The patient died following an AE.

Life-threatening experience: Any AE that places the patient, in the view of the reporter, at immediate risk of death from the AE as it occurred, i.e., does not include an AE that had it occurred in a more severe form, might have caused death.

Requires inpatient hospitalization, or prolongation of existing hospitalization: The AE resulted in an initial inpatient hospitalization or prolonged an existing hospitalization of the patient. For this protocol, hospitalization is defined as an admission to a medical facility of greater than 24 hours.

Persistent or significant disability/incapacity: An AE that results in a substantial disruption of a person's ability to conduct normal life functions.

Congenital anomaly: The exposure of the patient to the drug during pregnancy that is judged to have resulted in the congenital anomaly/birth defect.

Important medical events: AEs that may not result in death, be life-threatening, or require hospitalization may be considered SAEs when, based upon appropriate medical judgment, they may jeopardize the patient and may require medical or surgical intervention to prevent one of the outcomes listed above.

Planned hospital admissions or surgical procedures for an illness or disease that existed before the administration of the study treatment or before the patient was enrolled in the study, will not be captured as SAEs unless they occur at a time other than the planned date.

Serious adverse events will be followed until resolution or until such time agreed upon by the Sponsor or designee and the Investigator. Adverse events and concomitant medications will be collected from the time of informed consent through study completion (Week 50 or Early Termination).

9.2.3 Evaluation of Adverse Events and Serious Adverse Events

9.2.3.1 Severity of Adverse Events

The Investigator will assess the severity of the (S)AE using the following categories: Mild, Moderate, and Severe. This assessment is subjective, and the Investigator should use medical judgment to compare the reported (S)AE to similar events observed in clinical practice.

Guidelines for severity assessment are listed below:

Mild: Symptom(s) barely noticeable to the patient or do(es) not make the patient uncomfortable. The (S)AE does not influence performance or functioning. Prescription drugs are not ordinarily needed for relief of symptom(s).

Moderate: Symptom(s) of a sufficient severity to make the patient uncomfortable. Performance of daily activities is influenced. Treatment of symptom(s) may be needed.

Severe: Symptom(s) of a sufficient severity to cause the patient severe discomfort. Severity may cause cessation of treatment with the study drug. Treatment for symptom(s) may be given.

Severity is not equivalent to seriousness.

9.2.3.2 Action Taken/Medication or Therapy Given

The Investigator will be required to provide any action taken with regards to an (S)AE. The action taken is determined as follows:

No change: No change in product dosing was implemented. This includes cases in which the product was discontinued prior to the occurrence of the (S)AE.

Temporarily Discontinued: The drug was discontinued temporarily and will probably be restarted at a later time point. The Investigator will provide a stop and restart date.

Permanently Discontinued: The drug was discontinued permanently. The Investigator will provide a stop date.

9.2.3.3 Outcome of Adverse Events

The Investigator will be required to provide information regarding the patient outcome of an (S)AE to the study drug.

The patient outcome is determined as follows:

Recovered: The (S)AE has resolved and the patient does not have any residual symptoms.

Recovered With Sequelae: The (S)AE has resolved, however the patient has some residual symptoms. The Investigator will provide information to specify the sequelae.

Not Yet Recovered: The patient has not yet recovered from the (S)AE. These cases will be followed-up until the report is classified in one of the other categories or follow-up is no longer required as determined by the Investigator. The Investigator must follow patients with (S)AEs until their condition resolves or stabilizes. Certain (S)AEs may not resolve. Serious adverse events will be followed until resolution or until such time agreed upon by the Sponsor or designee and the Investigator.

Ongoing at the time of death: The (S)AE was ongoing at the time of death of the patient and was not directly related to the patient's death.

Fatal: The patient died from the (S)AE. The patient's date of death is the date of the (S)AE cessation.

9.2.3.4 Relationship to Study Treatment

The Investigator will assess the relationship between the (S)AE and the study treatment according to the following definitions:

Unrelated: There was no relationship of the (S)AE to the use of the study drug. This may include, but is not limited, to the (S)AE being an expected outcome of a previously existing or concurrent disease, concomitant medication or procedure the patient experienced during their treatment period. For reporting purposes, Unrelated will be considered Not Product Related.

Remote/Unlikely: (Serious) Adverse experiences which are judged probably not related to the drug. For reporting purposes, Remote/Unlikely will be considered Not Product Related.

Possible: There was no clear relationship of the (S)AE to the use of the study drug; however, one cannot definitively conclude that there was no relationship. For reporting purposes, Possible will be considered Product Related.

Probable: While a clear relationship to the drug cannot be established, the experience is associated with an expected (S)AE or there is no other medical condition or intervention which would explain the occurrence of such an experience. For reporting purposes, Probable will be considered Product Related.

Definite: The relationship of the use of the drug to the experience is considered definitively established. For reporting purposes, Definite will be considered Product Related.

9.2.3.5 Clinical Laboratory Tests

The Investigator will assess all laboratory results outside the normal range as CS or NCS. Clinical significance is defined as any variation that has medical relevance resulting in an alteration in medical care. Whenever possible, the underlying diagnosis should be listed in lieu of the abnormal laboratory values. Laboratory abnormalities deemed NCS by the Investigator should not be reported as (S)AEs. Similarly, laboratory abnormalities reported as (S)AEs by the Investigator should not be deemed NCS on the laboratory sheet.

9.2.3.6 Dosing Errors

Dosing details should be captured on the Dosing eCRF. In addition, if the patient takes a dose of study drug that significantly exceeds protocol specifications, the "Dose/dosing rate exceeded protocol specifications" box should also be checked. If the patient is symptomatic, the "Associated with AE(s)? - Yes" box should be checked. If the event does not meet serious criteria, the symptom(s) should be documented on the non-serious AE eCRF and the corresponding AE numbers listed on the Dosing eCRF; if the event meets serious criteria, an SAE form should be completed and the event reported as a SAE instead. The site should also document the SAE on the AE eCRF and enter the corresponding AE numbers on the Dosing eCRF. Do not capture the event on the non-serious AE eCRF or SAE form if the patient is not symptomatic.

9.2.4 Adverse Experience and Serious Adverse Experience Reporting

9.2.4.1 Adverse Experience Reporting

Adverse events, including SAEs, will be reported from the time the patient signs the informed consent form until study completion. All AEs, including SAEs, experienced by the patient will be noted on the eCRF. A full description including the nature, date, time of onset and resolution, determination of seriousness, intensity, corrective treatment, outcome, and relationship to study drug will be recorded. Changes in vital signs, laboratory results and other safety assessments from Baseline will be recorded if they are deemed clinically significant. When possible, a diagnosis should be recorded as an AE, rather than the symptoms related to that diagnosis. A medical or surgical procedure is not an AE; rather the condition leading to the procedure should be recorded as the AE. All SAEs experienced by the patient will be recorded on a SAE report form and reported to Genzyme.

The Investigator must follow patients with AEs until their condition resolves or stabilizes. Certain AEs may not resolve. Serious adverse events will be followed until resolution or until such time agreed upon by Genzyme or its designee and the Investigator. Adverse events and concomitant medications will be collected from the time of informed consent through study completion (Week 50 or Early Termination).

9.2.4.2 Serious Adverse Event Reporting

9.2.4.2.1 Reporting to Genzyme

All SAEs must be reported by to the Genzyme Pharmacovigilance Department within 24 hours of the Investigator's first knowledge of the event (Table 9-4). SAE communication is directed to:

Table 9-4 Contact List for Serious Adverse Event Reporting

United States and Rest of World (excluding Europe)	Europe
**Genzyme US Pharmacovigilance**<br><br>FAX: 1 617 761 8506<br><br>Email:<br>pharmacovigilancesafety@genzyme.com<br><br>Phone:<br>Night (6 pm EST- 8 am EST), Weekends and Holidays<br>1 800 745 4447 (US toll free number)<br>1 617 768 9000 (outside US)	**Genzyme Europe BV Pharmacovigilance**<br><br>FAX: +31 (0) 35 694 87 56<br><br>Email:<br>EUPharmacovigilance@genzyme.com<br><br>Phone:<br>7days/24hours<br>+31 (0) 35 699 12 99

For all SAEs, a Serious Adverse Event and Medical Event of Interest Form (SAE/MEOI form) that includes a detailed written description, copies of relevant anonymized patient records, and other documents will be sent to Genzyme Pharmacovigilance within 24 hours. Follow-up SAE reports will be forwarded as soon as the information is available.

Additionally, the Institutional Review Board (IRB)/Independent Ethics Committee (IEC) must also be notified in writing of any SAEs according to the applicable regulations. Genzyme will report SAEs to appropriate regulatory agencies, as required.

After the study is completed, if the Investigator or study staff becomes aware of a related SAE that occurs within 30 days of study completion, this event and any known details must be reported to Genzyme Pharmacovigilance.

9.2.4.2.2 Medical Events of Interest

An MEOI is a noteworthy event for the particular product or class of products that a Sponsor may wish to carefully monitor. It could be a serious or non-serious event. Note: An MEOI is not an SAE unless it meets the SAE criteria as defined in Section 9.2.2. **Events to be reported as MEOIs for mipomersen protocols**:

- Discontinuations due to AEs
- Discontinuations due to *confirmed/presumed confirmed* stopping rule criteria
- Incidents of patients that reach *confirmed/presumed confirmed* laboratory safety monitoring rule criteria

An SAE/MEOI form should be completed and a copy faxed to Genzyme Pharmacovigilance (see Study Contact List).

9.2.5 Pregnancy Reporting

If a patient becomes pregnant at anytime during the conduct of this study, she must not receive any additional study drug and must be discontinued from the study.

The patient must be followed until the outcome of the pregnancy is known (i.e., delivery, elective termination, or spontaneous abortion). If the pregnancy results in birth of a child, additional follow-up information may be requested.

In case of paternal exposure anytime after receiving study treatment, the patient may continue the study. However, the patient's pregnant partner must be followed until the outcome of the pregnancy is known (i.e., delivery, elective termination, or spontaneous abortion). If the pregnancy results in the birth of a child, additional follow-up information may be requested.

The Investigator must notify Genzyme Pharmacovigilance within 24 hours of first learning of the occurrence of pregnancy, using the appropriate Pregnancy Notification Form(s) (Appendix 14.3, Forms A and/or B), providing as much information as possible. For reporting initial notification of a female study patient please provide Pregnancy Notification Form A. If study patient is male, please complete and provide both Pregnancy Notification Forms A and B. The Investigator must notify Genzyme about reported complications within 24 hours using the same procedure. Outcome of pregnancy, once known by the Investigator, must also be reported to Genzyme within 24 hours using the Pregnancy Outcome Form C and faxing it to Genzyme. For reporting additional information about the pregnancy use Pregnancy Notification Form(s) A and/or B and indicate "follow-up" on the form.

Communications regarding pregnancies should be directed to the contacts listed in Table 9-4.

Please note that pregnancy in and of itself is neither an AE nor a SAE. Pregnancy should not be entered into the eCRF as an AE unless the Investigator suspects an interaction between the study treatment and contraceptive method used. For female patients, pregnancy will be documented as the reason for study discontinuation.

9.2.6 Stopping Rules and Safety Monitoring

In addition to the standard monitoring of clinical safety parameters, the following guidelines are provided for the monitoring of selected parameters chosen based on preclinical and

clinical observations (see Section 8, "Guidance for the Investigator," in the Investigator's Brochure).

9.2.6.1 Confirmation Guidance

At any time during the study (treatment or post-treatment periods), initial clinical laboratory results meeting the stopping rules or safety monitoring criteria presented below must be confirmed by performing measurements (ideally in the same laboratory that performed the initial measurement) on new specimens except for ALT or AST levels ≥ 8 x ULN. All new specimen collections should take place as soon as possible (ideally within 3 days of the initial collection) and, in any event, no longer than 5 days from the collection that yielded the initial observation. If the initial laboratory result is observed during the treatment period, the results from the retest should be available prior to the next scheduled dose of study drug (mipomersen or placebo).

If a new specimen is not, or cannot be, collected ≤ 7 days of the specimen collection that produced the initial laboratory observation and/or the result from the test of the new specimen is not available prior to the scheduled administration of the next dose, the initial laboratory result is presumed confirmed.

9.2.6.2 Stopping Rule Guidance

If any of the stopping criteria described below are met and are confirmed, the patient will be permanently discontinued from further treatment with study drug (mipomersen or placebo), evaluated fully as outlined below and in consultation with the Medical Monitor, and will be entered into the post-treatment evaluation portion of the study. In general, patients who do not meet the stopping rules based upon retest may continue dosing at the discretion of the Investigator. However, the Investigator and the Medical Monitor should confer as to whether additional close monitoring of the patient is appropriate.

9.2.6.3 Liver Chemistry Rules

The following rules are adapted from the draft guidance for industry, "Drug-Induced Liver Injury: Premarketing Clinical Evaluation," issued by the U.S. Department of Health and Human Services, Food and Drug Administration, October 2007.

9.2.6.3.1 Stopping Rules for Liver Chemistry Elevations

In the event of single laboratory result meeting the following criteria, dosing of a patient with study drug (mipomersen or placebo) will be stopped permanently:

- ALT or aspartate aminotransferase (AST) ≥ 8 x ULN

In the event of confirmed or presumed confirmed laboratory results meeting the following criteria, and the event is without an alternative explanation as discussed with the Medical Monitor (e.g., concomitant therapy with anticoagulants), dosing of a patient with study drug (mipomersen or placebo) will be stopped permanently:

- ALT or AST ≥ 5 x ULN at 2 consecutive weekly measurements (not less than 7 days) at least 1 of which is confirmed by retest. (Weekly measurement of liver chemistry tests will be instituted following the first ALT or AST ≥ 5 x ULN finding.)
- ALT or AST ≥ 3 x ULN and total bilirubin > 1.5 x ULN or international normalized ratio (INR) > 1.5

In the event of confirmed or presumed confirmed laboratory results for either of the following criteria, continued dosing of a patient with study drug (mipomersen or placebo) will be discussed with the Medical Monitor:

- ALT or AST ≥ 3 x ULN with the appearance or worsening of symptoms felt by the Investigator to be potentially related to hepatic inflammation, such as fatigue, nausea, vomiting, right upper quadrant pain or tenderness, fever, rash or eosinophilia

9.2.6.3.2 Safety Monitoring Rules for Liver Chemistry Tests

In the event of an ALT or AST measurement that is ≥ 3 x ULN at any time during the study (treatment or post-treatment period), the initial measurement(s) should be confirmed as described above. Similarly, confirmatory measurements should also be performed if ALT or AST levels increase to ≥ 5 x ULN (following the initial meeting of each of those criteria).

Frequency of Repeat Measurements: Patients with confirmed or presumed confirmed ALT or AST levels ≥ 3 x ULN should have their liver chemistry tests (ALT, AST, alkaline phosphatase, and total bilirubin at a minimum) retested at least once weekly until levels stabilize in the judgment of the Investigator. The performance of additional laboratory tests should be discussed with the Medical Monitor. Thereafter, liver chemistry tests for these patients should be performed at least every 2 weeks for the first 6 weeks that these patients have met this safety monitoring rule, or until ALT and AST are both < 3 x ULN. If ALT or AST levels increase to ≥ 3 x ULN after reducing below 3 x ULN, the monitoring plan should be discussed with the Medical Monitor and individualized for the patient. Subsequently, liver chemistry tests for these patients should be performed at least once a month until their ALT and AST levels become ≤ 1.2 x ULN (or ≤ 1.2 x Screening value for patients who enter the study with ALT and/or AST > ULN).

Further Investigation into Liver Chemistry Elevations: For patients with confirmed or presumed confirmed ALT or AST levels ≥ 3 x ULN, the following evaluations should be performed:

- A detailed history of symptoms and prior and concurrent diseases.
- A history for concomitant drug use (including nonprescription medications, herbal and dietary supplement preparations), alcohol use, recreational drug use, and special diets.
- A history for exposure to environmental chemical agents and travel.
- Serology for viral hepatitis (hepatitis A virus [HAV] immunoglobulin M [IgM], hepatitis B surface antigen [HBsAg], hepatitis C virus [HCV] antibody, cytomegalovirus (CMV) IgM, and Epstein-Barr Virus [EBV] antibody panel)
- Serum albumin, PT or INR, and partial thromboplastin time (PTT)
- Serology for autoimmune hepatitis (e.g., antinuclear antibody [ANA]).
- Liver MRI (chemical shift imaging) or CT

Additional liver evaluations, including gastroenterology/hepatology consults, may be performed at the discretion of the Investigator, in consultation with the Medical Monitor. Repetition of the above evaluations should be considered if a patient’s ALT and/or AST levels reach ≥ 5 x ULN.

9.2.6.4 Safety Monitoring Rules for Renal Function Test Results

In the event of confirmed or presumed confirmed laboratory results for either of the following criteria, continued dosing of a patient with study drug (mipomersen or placebo) will be discussed with the Medical Monitor:

- Serum creatinine increase ≥ 0.2 or 0.3 mg/dL (≥ 17.7 or 26.5 µmol/L) above Baseline, for women and men, respectively
- Proteinuria, dipstick ≥ 2 + (confirmed by dipstick re-measurement and then further confirmed by a quantitative total urine protein measurement of > 1.0 g/24 hr)

The follow-up schedule for any events meeting either of these criteria will be determined by the Investigator in consultation with the Medical Monitor.

9.2.6.5 Safety Monitoring Rule for Platelet Count Results

In the event of a confirmed or presumed confirmed platelet count less than 75,000/mm^3, continued dosing of a patient with study drug (mipomersen or placebo) will be discussed with the Medical Monitor. The follow-up schedule for any events meeting this criterion will be determined by the Investigator in consultation with the Medical Monitor.

9.2.6.6 Safety Monitoring Rules for Coagulation Parameters

In the event that a patient has a confirmed or presumed confirmed finding for either of the following 2 criteria and the event is without an alternative explanation (e.g., concomitant therapy with anticoagulants), the Investigator should discuss the follow-up of the event with the Medical Monitor.

- aPTT > 75 seconds
- PT > 20 seconds or INR > 1.5

9.2.6.7 Safety Monitoring Rule for Constitutional Symptoms

Patients will be instructed to report any constitutional symptoms, such as fever, that may arise after an injection is given. The Investigator should closely evaluate all potential causes of these symptoms, including concomitant illness.

9.2.6.8 Restriction on the Lifestyle of Patients

Patients will be counseled to follow the TLC approach (see Appendix 14.1), or similar approach depending on local guidelines, throughout the course of the study.

Patients will be required to fast overnight for > 10 hours (only water permitted) before a blood sample is taken for a full serum lipid panel on all visit days. Any medication should be taken as prescribed, per Investigator judgment, even when fasting blood samples are planned to be drawn.

Patients should be willing to limit alcohol consumption for the entire duration of the study, including follow-up: male patients to a maximum of 2 drinks (20 g) per day, and 8 drinks (80 g) per week; female patients to a maximum of 1 drink (10 g) per day, and 4 drinks (40 g) per week. Up to 2 episodes of exceeding this limit by a small amount may be allowed during the course of the study.

Female patients of childbearing potential must continue to use appropriate and highly effective contraception with their partners, or refrain from sexual activity, for 4 weeks prior to Screening and throughout study participation until 6 months after the last study drug dose. Male patients must use an acceptable and highly effective contraceptive method during the treatment phase and until 6 months after the last study drug dose. Oral contraceptives and contraceptive patches are contraindicated in female patients enrolled in this study, but are an acceptable method of contraception for female partners of enrolled patients. Other acceptable methods of contraception are condoms with contraceptive foam, implantable or injectable contraceptives, intrauterine device, diaphragm with spermicidal gel, or sexual partner who is surgically sterilized or postmenopausal.

9.2.7 Summary of Risks

Please refer to the Investigator's Brochure for information regarding the risks and benefits of mipomersen.

10 DATA COLLECTION, QUALITY ASSURANCE, AND MANAGEMENT

10.1 Recording of Data

Original documents, data, and records being: hospital records, clinical and office charts, laboratory notes/reports, memoranda, patients' evaluation checklists, pharmacy dispensing records, recorded data from automated instruments, copies of transcriptions certified after verification as being accurate copies, source document worksheets (i.e., for study visits and IVRS), and X-rays are considered source documents. Medical histories and narrative statements relating to the patient's progress (i.e., source documents) will be maintained during the trial and for a period of 15 years after completion of the study. The Investigator must provide direct access to the source documents to the Sponsor or designee.

All data captured for this study are to be recorded in the patient's notes first and then entered in the eCRF.

Clinical data will be entered using electronic data capture (EDC) technology from a reputable vendor. All data captured electronically will be provided on a CD-ROM at the end of the study in PDF format.

All required data will be recorded in the eCRF. All missing data will be explained. Any changes made to the data after initial entry will be captured in an electronic audit trail.

10.2 Data Quality Assurance

The eCRFs will be reviewed manually at the study site for completeness by a Clinical Monitor from the Sponsor or designee, and returned to the Sponsor or designee, for data management and analysis. If necessary, the study site will be contacted for corrections and/or clarifications. All data will be entered into a study database for analysis and reporting. Any data captured electronically will be electronically transferred to the database. Upon completion of data entry, the database will receive a QA check to ensure acceptable accuracy and completeness.

10.3 Data Management

The Sponsor or designee will be responsible for:

- Database creation and validation
- eCRF review and data validation

Prior to finalizing and locking the database, all decisions concerning the inclusion or exclusion of data from the analysis for each patient will be determined by appropriate clinical and statistical personnel. Any and all exclusions related to either safety or efficacy will be documented in patient listings.

11 STATISTICAL METHODS AND PLANNED ANALYSES

The Sponsor or designee will be responsible for the production of the following items:

1. Data listings and summary tables
2. Statistical analysis
3. Combined clinical and statistical study report

All eCRF data, as well as any outcomes derived from the data, will be summarized in detailed data listings. Patient data listings will be presented for all patients enrolled into the study.

Descriptive summary statistics including n, mean, median, standard deviation, interquartile range (25th percentile, 75th percentile), and range (minimum, maximum) for continuous variables, and counts and percentages for categorical variables will be used to summarize most data. Where appropriate, p-values will be reported. All statistical tests will be conducted using 2-sided tests with 5% Type I error rates unless otherwise stated.

Prior to locking the database and unblinding the study, all decisions concerning exclusion of patients from analysis sets will be made by appropriate clinical and statistical personnel. The Safety Set will include all randomized patients who received at least 1 injection. The Full Analysis Set, which represents the practically-feasible intent-to-treat (ITT) population as delineated in ICH Guideline E9, will include the subset of the Safety Set with at least 1 post-Baseline LDL-C measure. The Per-Protocol Set will include the subset of the Full Analysis Set with no significant protocol deviations that would be expected to effect efficacy assessments. All efficacy parameters will be assessed on the Per-Protocol Set and Full Analysis Set (primary analysis population). All safety assessments will be performed on the Safety Set.

11.1 Power and Determination of Sample Size

Based upon prior clinical trial experience with mipomersen, it is estimated that the standard deviation of the percent change in LDL-C is approximately 22%. With 45 randomized patients (15 in the control group and 30 patients in the mipomersen-treated group), there would be 80% power to detect a 20 percentage point difference between the 2 groups. Enrollment will be conducted such that at least 51 patients are randomized to allow for potential exclusions from an analysis set. Should enrollment continue such approximately 75 patients are randomized, this will yield as much as 90% power to detect a 20 percentage point difference between the 2 groups.

11.2 Demographics and Baseline Characteristics

Demographic and baseline characteristics will be summarized using descriptive statistics by treatment group.

11.3 Patient Accountability

Patient randomization will be summarized by study site and treatment group. The patient disposition will be summarized.

All patients enrolled will be included in a summary of patient accountability for this study. The frequency and percentages of patients enrolled, presenting at each visit, discontinuing before study completion (including reason for discontinuation), and completing the study will be presented.

11.4 Study Treatment Usage and Compliance

Treatment duration and amount of study drug received will be summarized by treatment group.

11.5 Efficacy

11.5.1 Primary Efficacy Endpoint

The primary efficacy endpoint is the percent change in LDL-C from Baseline to Week 28/ET (ET represents the observation closest to 2 weeks after last dose among patients who early terminate study medication dosing).

11.5.2 Secondary Efficacy Endpoints

Secondary efficacy endpoints include the percent change in apoB and Lp(a) from Baseline to Week 28/ET and the percent of patients with LDL-C below LDL-C entry criteria at Week 28/ET (200 mg/dL [5.1 mmol/L] for patients with coronary heart disease [CHD] or other atherosclerotic disease, or 300 mg/dL [7.8 mmol/L]). The incremental effects of mipomersen on total cholesterol, TG, non-HDL-C, VLDL-C, apo A-1, and lipoprotein subclasses may also be evaluated.

11.5.3 Efficacy Analysis

Percent change from Baseline to Week 28/ET in LDL-C, apo B, total cholesterol, HDL-C, non-HDL-C, TG, and Lp(a) will be compared between treatment groups using a 2-sample t-test (if data departs substantially from normality; a Wilcoxon rank sum test will be used). The percent of patients with LDL-C below LDL-C entry criteria at Week 28/ET will be compared between treatment groups using Fisher's exact test. The analysis will take place after all patients have completed Week 28 and this database has been locked.

The Baseline is the value on Day 1, prior to the first dose of study drug. For patients without a Day 1 value (e.g., missing or inadequate sample), the last observation prior to the first dose of study drug will be used as Baseline.

11.6 Safety

11.6.1 Safety Endpoints

Safety will be determined using the incidence of treatment-emergent AEs, clinical laboratory evaluations, vital signs, ECGs, and physical examination findings. AEs will be categorized using MedDRA. Plasma concentrations of mipomersen will be evaluated.

11.6.2 Safety Analysis

The safety analyses will be performed on the Safety Set defined as all patients who receive at least 1 injection. Treatment duration and amount of study drug received will be summarized by treatment group, as will concomitant medications and treatments.

Treatment-emergent AEs will be summarized by body system, preferred term, severity, and relationship to the study procedures/treatments. If a patient has more than 1 occurrence of the same AE, he/she will be counted only once within that preferred term in the summary tables. The most severe occurrence of an AE, as well as the most extreme relationship of the AE to the study procedures or treatment, will be indicated in cases of multiple occurrences of the same AE. All AEs will be presented in a listing. Additionally listings of SAEs and AEs leading to discontinuation will be generated.

Clinical laboratory evaluations, vital signs, ECGs, and physical examination findings will be tabulated by treatment group. In addition, the number of patients who experience abnormalities in clinical laboratory evaluations will be summarized by treatment group.

Trough plasma concentrations of mipomersen (C_{min}) will be summarized over time by individual patient and by treatment group. Elimination half-life of mipomersen will be estimated for patients who enter the post-treatment follow-up period. Exploratory analyses will include but may not be limited to comparisons of trough plasma concentration of mipomersen over time within individual patients and within each treatment group.

11.7 Statistical Analysis Plan

A formal statistical analysis plan will be developed and finalized prior to database lock for the primary efficacy endpoint (e.g., all patients have completed Week 28).

12 SPECIAL REQUIREMENTS AND PROCEDURES

12.1 Institutional and Ethical Review

This protocol was designed and will be conducted, recorded, and reported in compliance with the principles of GCP guidelines as well as in accordance with all national, state and local laws of the appropriate regulatory authorities. These guidelines are stated in "Guidance for Good Clinical Practice," ICH Technical Requirements for Registration of Pharmaceuticals for Human Use.

A copy of the protocol, proposed informed consent form, other written patient information, and any proposed advertising material must be submitted to the IRB/IEC for written approval. A copy of the written approval of the protocol and informed consent form must be received by the Sponsor or designee before recruitment of patients into the study and shipment of study drug.

The Investigator must submit and, where necessary, obtain approval from the IRB/IEC for all subsequent protocol amendments and changes to the informed consent document. The Investigator should notify the IRB/IEC of deviations from the protocol or SAEs occurring at the study center and other AE reports received from the Sponsor or designee in accordance with local procedures.

The Investigator will be responsible for obtaining annual IRB/IEC approval/renewal throughout the duration of the study. Copies of the Investigator's reports and the IRB's/IEC's continuance of approval must be sent to the Sponsor or designee.

12.2 Changes to the Conduct of the Study or Protocol

No change in the study procedures shall be effected without the mutual agreement of the Investigator and Sponsor. All changes must be documented by signed protocol amendments. If changes to the design of the study are made, the amendment must be submitted to and approved by the IRB/IEC, signed by the Investigator, and returned to the Sponsor for submission to the appropriate regulatory authorities (e.g., US Food and Drug Administration [FDA], European national regulatory agencies, etc.).

12.3 Investigator's Responsibilities

12.3.1 Patient Informed Consent

The written informed consent document should be prepared in the language(s) of the potential patient population, based on an English version provided by the Sponsor or designee.

Before a patient's participation in the trial, the Investigator is responsible for obtaining written informed consent from the patient after adequate explanation of the aims, methods, anticipated benefits, and potential hazards of the study and before any protocol-specific screening procedures or any study drugs are administered.

The acquisition of informed consent and the patient's agreement or refusal of his/her notification of the primary care physician should be documented in the patient's medical records, and the informed consent form should be signed and personally dated by the patient and by the person who conducted the informed consent discussion (not necessarily an Investigator). The original signed informed consent form should be retained in accordance with institutional policy, and a copy of the signed consent form should be provided to the patient or legally acceptable representative.

12.3.2 Electronic Case Report Forms

All data will be obtained using EDC. Refer to Section 10.1 for details regarding recording of data.

Copies of pertinent records in connection with the study, including patient charts, laboratory data, etc., will be made available to Genzyme on request with due precaution towards protecting the privacy of the patient.

12.3.3 Record Retention

Essential documents should be retained until at least 2 years after the last approval of a marketing application in an ICH region and until there are no pending or contemplated marketing applications in an ICH region or at least 2 years have elapsed since the formal discontinuation of clinical development of the investigational product. Patient files and other source data must be kept for the maximum period of time permitted by your institution, but not less than 15 years. Should Investigators be unable to continue maintenance of patient files for the full 15 years, Genzyme will assist in this regard.

These documents should be retained for a longer period, however, if required by the applicable regulatory requirements or by an agreement with the Sponsor. It is the responsibility of the Sponsor to inform the Investigator/institution as to when these documents no longer need to be retained.

Essential documents are those documents, which individually and collectively, permit evaluation of the conduct of a trial and the quality of the data produced. These documents serve to demonstrate the compliance of the Investigator, Sponsor or designee, and monitor with the standards of GCP and with all applicable regulatory requirements.

Any or all of the documents should be available for audit by the Sponsor's or designee's auditor and inspection by the regulatory authority(-ies).

12.3.4 Patient Discontinuation

If a patient decides to discontinue participation in the study, he or she should be contacted in order to obtain information about the reason(s) for discontinuation and collection of any potential AEs. Whenever possible the patient should return to the clinic for the Week 28 assessments. The Investigator will document the eCRF describing the reason for discontinuation.

12.3.5 Study or Site Termination

The Sponsor reserves the right to terminate the study, according to the study contract. For example, the study may be terminated and dosing stopped at any time if the Medical Monitor is of the opinion that proceeding with the study will compromise the safety of the patients in the study. The Investigator should notify the regulatory authority and IRB/IEC in writing of the trial's completion or early termination and provide a copy of the notification to the Sponsor or designee.

12.3.6 Monitoring

The Sponsor representative or designee and regulatory authority inspectors are responsible for contacting and visiting the Investigator for the purpose of inspecting the facilities and, upon request, inspecting the various records of the trial (for example, eCRFs and other pertinent data) provided that patient confidentiality is respected.

The Sponsor representative or designee is responsible for inspecting the eCRFs at regular intervals throughout the study to verify adherence to the protocol; completeness, accuracy, and consistency of the data; and adherence to local regulations on the conduct of clinical research. The monitor should have access to patient medical records and other study-related records needed to verify the entries on the eCRFs.

The Investigator agrees to cooperate with the monitor to ensure that any problems detected in the course of these monitoring visits, including delays in completing eCRFs, are resolved.

In accordance with ICH GCP and the Sponsor's audit plans, this study may be selected for audit by representatives from the Sponsor's Clinical and QA Department (or designees). Inspection of study center facilities (e.g., pharmacy, drug storage areas, laboratories) and review of study-related records will occur to evaluate the trial conduct and compliance with the protocol, ICH GCP, and applicable regulatory requirements.

12.3.7 Materials Control

12.3.7.1 Receipt of Clinical Supplies

The study staff is required to document the receipt of study drug supplies.

12.3.7.2 Disposition of Unused Clinical Supplies

The study staff is required to document the dispensing, accountability, and return of study drug supplies. The study center must return all used and unused study drug as per Sponsor or designee instructions. Note that unused study drug should be maintained in refrigerated storage (2° to 8°C) until returned. Refer to the Investigational Product Handling Manual for additional details.

12.3.8 Warnings, Precautions, Contraindications

For specific information concerning warnings, precautions, and contraindications, the Investigator is asked to refer to the appropriate section of the Investigator's Brochure. Because of the possibility of AEs, a fully equipped emergency cart, or equivalent supplies and equipment, and personnel competent in recognizing and treating adverse reactions of all types should be immediately available.

Dose-dependent, transient, and reversible prolongations of aPTT have been observed with phosphorothioate oligonucleotides. Therefore, mipomersen should be used with caution in patients receiving warfarin, heparin or fractionated heparin products.

12.3.9 Clinical Study Report

If deemed appropriate by the Sponsor, the Coordinating Investigator shall be designated to sign the completed clinical study report at the end of this study.

The signatory Coordinating Investigator shall be identified by the Sponsor upon the completion of the study, based upon factors including, but not limited to, prior clinical research experience and publications, patient enrollment and level of involvement in the study.

12.3.10 Disclosure of Data

All information obtained during the conduct of this study will be regarded as confidential, and written permission from the Sponsor is required prior to disclosing any information relative to this study. Manuscripts prepared for publication will be in accordance with the policy established and presented to the Investigator previously by the Sponsor. Submission to the Sponsor for review and comment prior to submission to the publisher will be required. This requirement should not be construed as a means of restricting publication, but is intended solely to assure concurrence regarding data, evaluations, and conclusions and to

provide an opportunity to share with the Investigator any new and/or unpublished information of which he/she may be unaware.

13 REFERENCES

Chen SH, Habib G, Yang CY, et al. Apolipoprotein B-48 is the product of a messenger RNA with organ-specific in-frame stop codon. *Science*. 1987;238(4825):363–366.

Cholesterol Treatment Trialists' Collaborators [CTTC]. Efficacy and safety of cholesterol-lowering treatment: prospective meta-analysis of data from 90,056 participants in 14 randomised trials of statins. *Lancet.* 2005;366:1267-1278.

Davidson MH, Maki KC, Pearson TA, et al. Results of the National Cholesterol Education Program (NCEP) Evaluation Project Utilizing Novel E-Technology (NEPTUNE) II survey and implications for treatment under the recent NCEP Writing Group Recommendations. *Am J Cardiol.* 2005;96(4):556-563.

Davis RA, Hui TY. 2000 George Lyman Duff Memorial Lecture. Atherosclerosis is a liver disease of the heart. *Arterioscler Thromb Vasc Biol*. 2001;21:887-898.

Gotto AM Jr. Management of dyslipidemia. *Am J Med*. 2002;112(8A):10S-18S.

Grundy SM, Cleeman JI, Merz CN, et al. Implications of recent clinical trials for the National Cholesterol Education Program Adult Treatment Panel III guidelines. *Circulation*. 2004;110:227-239.

Hulthe J, Fagerberg B. Circulating oxidized LDL is associated with subclinical atherosclerosis development and inflammatory cytokines (AIR study). *Arterioscler Thromb Vasc Biol*. 2002;22(7):1162-1167.

Knopp RH. Drug treatment of lipid disorders. *N Engl J Med*. 1999;341(7):498-511.

Third report of the National Cholesterol Education Program (NCEP) expert panel on detection, evaluation, and treatment of high blood cholesterol in adults (Adult Treatment Panel III) Final Report. *Circulation*. 2002;106(25):3143-3421.

Powell LM, Wallis SC, Pease RJ, Edwards YH, Knott TJ, Scott J. A novel form of tissue-specific RNA processing produces apolipoprotein-B48 in intestine. *Cell*. 1987;50(6):831–840.

Sankatsing RR, Fouchier SW, de Haan S, et al. Hepatic and cardiovascular consequences of familial hypobetalipoproteinemia. *Arterioscler Thromb Vasc Biol.* 2005;25:1979-1984.

Skalen K, Gustafsson M, Rydberg EK, et al. Subendothelial retention of atherogenic lipoproteins in early atherosclerosis. *Nature*. 2002;417(6890):750-754.

Sueta CA, Massing MW, Chowdhury M, Biggs DP, Simpson RJ Jr. Undertreatment of hyperlipidemia in patients with coronary artery disease and heart failure. *J Card Failure*. 2003;9(1):36-41.

14 APPENDICES

14.1 NCEP – ATP III Therapeutic Lifestyle Changes

- Reduced intakes of saturated fats (< 7% of total calories) and cholesterol (< 200 mg per day)
- Therapeutic options for enhancing LDL lowering such as plant stanols/sterols (2 g/day) and increased viscous (soluble) fiber (10 to 25 g/day)
- Weight reduction
- Increased physical activity

Table 14-1 Nutrient Composition of the TLC Diet

Nutrient	Recommended Intake
Saturated fat*	Less than 7% of total calories
Polyunsaturated fat	Up to 10% of total calories
Monounsaturated fat	Up to 20% of total calories
Carbohydrate†	50% to 60% of total calories
Fiber	20 to 30 g/day
Protein	Approximately 15% of total calories
Cholesterol	Less than 200 mg/day
Total calories (energy)‡	Balance energy intake and expenditure to maintain desirable body weight/prevent weight gain

*Trans fatty acids are another LDL-raising fat that should be kept at a low intake.
†Carbohydrate should be derived predominantly from foods rich in complex carbohydrates including grains, especially whole grains, fruits, and vegetables.
‡Daily energy expenditure should include at least moderate physical activity (contributing approximately 200 kcal per day)

14.2 Lipoprotein Testing

Lipoprotein testing will be performed in a clinical laboratory which holds a current certification of traceability to the national reference system for total cholesterol. In addition, the analytical systems and reagents used to measure total cholesterol and HDL-C must be certified by the Cholesterol Reference Method Laboratory Network (CRMLN) as having documented traceability to the national reference system for total cholesterol, and the designated comparison method for HDL-C, respectively.

Table 14-2 Lipoprotein Testing

Lipoprotein	Analysis Method
Total Cholesterol	Enzymatic colorimetry
LDL-C	Friedewald Calculation; ultracentrifugation for TG > 400
HDL-C	Dextran sulfate precipitation followed by enzymatic colorimetry
Triglycerides	Enzymatic colorimetry
Apo B-100	Rate Nephelometry
Apo A-1	Rate Nephelometry
Lp(a)	Isoform Independent assay standardized to Northwest Lipid Research Clinic

14.3 Pregnancy Notification Forms

Genzyme Coporation
500 Kendall Street
Cambridge, MA 02142

Genzyme Europe BV
Gooimeer 10
1411 DD Naarden
Netherlands

Pregnancy Notification Form A

Maternal Exposure

Report Type: ☐ Initial ☐ Follow-up

Genzyme Use Only:
Case ID: ____
Report Type: ☐ Prospective ☐ Retrospective

Source of fetal exposure: ☐ Maternal ☐ Paternal (please also complete Pregnancy Notification Form B)

I. Physician Information:

Name	Address	Phone	Fax	Office Contact
Primary Care Provider				
OB				

II. Maternal Information:

Is the patient participating in a Genzyme Clinical Trial?

☐ Yes Protocol Number: ____ Subject's Study Number/Initials: ____/____

☐ No Patient's Initials: ____

Date of Birth: ___/___/___ dd / mmm / yyyy

Age at the time of Conception: ____

Country of Birth: ____

Country of Residence: ____

Occupation: ____

Race: ☐ Not provided ☐ Caucasian ☐ Black ☐ Asian ☐ Native American ☐ Hispanic ☐ Other____

Highest Education Level: ____ Height: ____ ☐cm ☐inches Weight: ____ ☐kg ☐lb

III. Product Exposure:

Genzyme Product: ____

Date of First Exposure: ___/___/___ dd / mmm / yyyy

Date of Last Exposure: ___/___/___ dd / mmm / yyyy

Trimester Exposure: ☐ 1st ☐ 2nd ☐ 3rd

Gestational Age at Time of Drug Exposure in Weeks: ____

Dose, Route & Frequency: ____

IV. Patient's History:

Patient's Previous Obstetric History: Grava:____ Para:____ Spontaneous Abortions:____ Other: ____

Were live births full-term? ☐ Yes ☐ No ____
If no, please describe.

Does the patient have a history of subfertility? ☐ No ☐ Yes____
If yes, please describe.

Patient's Relevant Medical and Social History (include information on known risk factors or conditions that may affect the outcome of the pregnancy e.g. alcohol, smoking or other substance consumption (include start and stop dates as appropriate), hypertension, eclampsia, diabetes, seizure disorder, thyroid disorder, asthma, allergic disease, heart disease, depression or other psychiatric disorders, sexually transmitted disorders, hepatitis, AIDs, environmental or occupational exposure that may pose a risk factor, or medical complications in a prior pregnancy): ____

Patient's Family History (includes hereditary diseases, congenital abnormalities, psychomotor retardation, and other familial disorders, both maternal and paternal): ____

Consanguinity between parents: ____
Specify degree.

Does the patient have a history of fetal or neonatal abnormalities? ☐ No ☐ Yes ____
If yes, please describe.

V. 1 June 2006

Please fax form to Genzyme Pharmacovigilance Department US at 617-761-8506 or EU at +31 35 694 8756.

Genzyme Coporation
500 Kendall Street
Cambridge, MA 02142

Genzyme Europe BV
Gooimeer 10
1411 DD Naarden
Netherlands

Pregnancy Notification Form A

Report Type: ☐ Initial ☐ Follow-up

Genzyme Use Only:
Case ID: ________
Report Type: ☐ Prospective ☐ Retrospective

V. Present Pregnancy: Number of Fetuses: ________

Date Pregnancy was Confirmed: ____/____/____ (dd / mmm / yyyy) Method by which Pregnancy was Confirmed: ________

Date of Last Menstrual Period: ____/____/____ (dd / mmm / yyyy) Expected Delivery Date: ____/____/____ (dd / mmm / yyyy) Is Pregnancy in-vitro? ☐ Yes ☐ No

Was the patient treated for infertility? ☐ No ☐ Yes ________
If yes, please describe.

Prenatal Testing	Date	Result	Reason	Comment<br>Please use section VII if needed
Ultrasound				
Alpha-fetoprotein				
Amniocentesis				
Chorionic villus sampling				
Serology (rubella, toxoplasmosis)				
Other				

VI. Concomitant Medication: Record medication(s) within the 30 days prior to last menstrual period.

Medication	Dose	Route	Duration	Indication

VII. Additional Information: Significant medical complications thus far in the pregnancy. Specifiy week of gestation that the complication occurred, treatment and outcomes. Attach additional information if required.

VIII. Reporter Information: (If different than section I.)
Name, Addresss, Telephone Number and Fax:

IX. Person Completing This Form

Signature: ________ Print Name: ________ Date: ________

V. 1 June 2006 **Please fax form to Genzyme Pharmacovigilance Department US at 617-761-8506 or EU at +31 35 694 8756.**

Page 2 of 2

Genzyme Coporation
500 Kendall Street
Cambridge, MA 02142

Genzyme Europe BV
Gooimeer 10
1411 DD Naarden
Netherlands

Pregnancy Notification Form B

Paternal Exposure

Report Type: ☐ Initial ☐ Follow-up

Genzyme Use Only:
Case ID:__________
Report Type: ☐ Prospective ☐ Retrospective

I. Paternal Information: Is the patient participating in a Genzyme Clinical Trial?

☐ Yes Protocol Number: __________ Subject's Study Number/Initials: _____/_____

☐ No Patient's intials: (last, first, middle)__________

Date of Birth: ___/___/___ dd / mmm / yyyy
Country of Birth: ________
Country of Residence: ________
Occupation: ________

Race: ☐ Not provided ☐ Caucasian ☐ Black ☐ Asian ☐ Native American ☐ Hispanic ☐ Other________

II. Product Exposure: Genzyme Product: __________

Date of First Exposure: ___/___/___ dd / mmm / yyyy
Date of Last Exposure: ___/___/___ dd / mmm / yyyy
Trimester Exposure: ☐ 1st ☐ 2nd ☐ 3rd
Gestational Age at Time of Drug Exposure in Weeks: ________
Dose, Route & Frequency: ________

III. Patient's History:

Patient's Relevant Medical and Social History (include alcohol, smoking or other substance consumption (include start and stop dates as appropriate), sexually transmitted disorders, hepatitis, AIDs, environmental or occupational exposure, etc.):

IV. Concomitant Medication: Record medication(s) within the 30 days prior to last menstrual period.

Medication	Dose	Route	Duration	Indication

V. Additional Information:

VI. Reporter Information:

Name, Addresss, Telephone Number and Fax:

VII. Person Completing This Form

Signature:__________ Print Name:__________ Date:________

V. 1 June 2006

Please fax form to Genzyme Pharmacovigilance Department US at 617-761-8506 or EU at +31 35 694 8756.

Page 1 of 1

Genzyme Coporation
500 Kendall Street
Cambridge, MA 02142

Genzyme Europe BV
Gooimeer 10
1411 DD Naarden
Netherlands

Pregnancy Outcome Form C

Report Type: ☐ Initial ☐ Follow-up

Genzyme Use Only:
Case ID: ____________
Report Type: ☐ Prospective ☐ Retrospective

I. Pregnancy Outcome: (If multiple birth, please photocopy and complete a form for each infant.)

Patient's intials: ________

☐ Live Birth: Date of Birth: ____/____/_____ (dd / mmm / yyyy) Weight: ______ ☐kg ☐lb Length: ______ ☐cm ☐inches Gestational age at delivery: ________

Type of live birth: ☐ Full term > 35 weeks gestation ☐ Preterm ≤ 35 weeks gestation ☐ Low birth weight <2500 grams

Method of Delivery: ☐ Vaginal ☐ Cesarean ____________ *Please provide indication*

Sex: ☐ Male ☐ Female

Apgar score: 1 Minute ________ 5 Minute ________

Did the newborn experience any complications during or after birth? ☐ No ☐ Yes ____________

Was there fetal dysmaturity? ☐ No ☐ Yes ____________ *If yes, please describe.*

Result of the newborns physical exam: ☐ Within Normal Limits ☐ Abnormal ____________ *Please describe major and minor congenital abnormalities* ☐ Unknown

☐ Non-Live Birth:

Date: ____/____/_____ (dd / mmm / yyyy) ☐ Elective termination ☐ Spontaneous Abortion (≤ 20 weeks) ☐ Stillborn (≥ 20 weeks)

Gestational age at termination: ________

Results of physical examination (gender, external anomalies) and pathology, if applicable: ____________

II. Obstetric Information:

☐ Yes ☐ No Complications during pregnancy, specify: ____________

☐ Yes ☐ No Complications during labor/delivery, specify: ____________

Medications administered to the mother during delivery: ____________

III. Additional Information: Describe any additional information regarding the outcome of this pregnancy:

IV. Pediatrician Contact:

Pediatrician Name	Address	Phone	Fax	Office Contact

V. Reporter Information: (If different than section IV.)
Name, Addresss, Telephone Number and Fax:

VI. Person Completing This Form

Signature: ____________ Print Name: ____________ Date: ________

V. 1 June 2006

Please fax form to Genzyme Pharmacovigilance Department US at 617-761-8506 or EU at +31 35 694 8756.

Page 1 of 1

SUMMARY OF CHANGES
MIPO3500108 AMENDMENT 1

Protocol Title:	A Prospective Randomized, Double-Blind, Placebo-Controlled Study to Assess the Safety and Efficacy of Mipomersen in Patients with Severe Hypercholesterolemia on a Maximally Tolerated Lipid-Lowering Regimen and who are not on Apheresis		
Protocol Number:<br>EudraCT:	MIPO3500108<br>2008-006020-53	Orig. Version Date:	FINAL: 8 October 2008
Amendment Number:	1	Version Date:	FINAL: 29 October 2008
Author Name:	Marisa Greenfield		
Author Signature:	Not required		

RATIONALE FOR CHANGE(S):
Original protocol MIPO3500108 has been modified to align with the Sponsor's overall development plan, simplifying the study design to include a single LDL-C inclusion criterion and to minimize the number of secondary endpoints. A single LDL-C entry criterion for patients with CAD will eliminate the risk of not enrolling sufficient numbers above 200 mg/dL (5.1 mmol/L). This change also aligns MIPO3500108 with other ISIS 301012 protocols.

- All secondary endpoints but apoB and Lp(a) have been removed.
- The percentage of patients achieving LDL-C below 200 mg/dL will be reported.
- The specified strata of LDL-C between 190 and 199 mg/dL have been removed. The lower level of LDL cut-off will now be ≥ 200 mg/dL.
- The efficacy analysis on the patients with LDL-C ≥ 200 mg/dL (5.1 mmol/L) has been removed, since this is no longer a subpopulation of interest

In addition, changes have been made to:

- Add the option for patients to participate an open-label extension study (if one is available) at Week 28, in which patients may either extend dosing or just continue follow-up
- Clarify that inclusion of patients on fibrates is allowed (if dose is stable)
- Exclude use of oral contraceptives, since these are medically contraindicated in patients with CHD

No patients have been or will be enrolled under the original protocol.

Finally, minor changes have been made to the text to increase the clarity of the protocol without impacting study intent or conduct.

Protocol Section/Page	Changed Text
Synopsis, Methodology/Page 3<br>Section 6.1 Study Design/Page 22	Approximately 7551 severely hypercholesterolemic adult patients will be randomized, who are on a maximally tolerated lipid-lowering regimen and not on apheresis, with low density lipoprotein cholesterol (LDL-C) ≥ 190 mg/dL (4.9 mmol/L), and who are not on apheresis will be randomized into this study. Patient enrollment will be conducted such that at least 51 patients are randomized with LDL-C of at least 200 mg/dL (5.1 mmol/L) for patients with coronary heart disease (CHD), or at least 300 mg/dL (7.8 mmol/L) for patients without CHD, representing. This represents a patient population in which LDL-C apheresis is indicated in the United States of America (USA).
Synopsis, Methodology/Page 3<br>Section 6.1 Study Design/Page 22	Additionally, approximately 24 patients with LDL-C of at least 190 mg/dL (4.9 mmol/L) will be randomized to provide additional exposure among a slightly broader patient populationAs many as 24 additional patients may be randomized to the study, for a total of 75 patients, to provide up to 90% power for efficacy and to provide further safety information.
Added new paragraph<br>Synopsis, Methodology/Page 3<br>Section 6.1 Study Design/Page 22	Following the treatment period and Week 28 evaluation, patients may elect to continue in an open-label extension study, if one is available, pending approval by the Ethics Committee and the appropriate regulatory authority. Patients who choose not to participate or are ineligible for the open-label extension study will continue post-treatment follow-up in the current protocol.
Synopsis, Number of Patients/ Page 4	ApproximatelyBetween 51 and 75 patients will be randomized into this study.
Synopsis, Inclusion/Exclusion Criteria, Inclusion Criterion 2/ Page 4<br>Section 7.1 Inclusion Criteria, Inclusion Criterion 2/Page 25	Fasting LDL-C ≥ 190200 mg/dL (4.95.1 mmol/L) and triglycerides (TG) < 350 mg/dL (4.0 mmol/L) at Screening
Synopsis, Inclusion/Exclusion Criteria, Inclusion Criterion 3e/ Page 4<br>Section 7.1 Inclusion Criteria, Inclusion Criterion 3e/Page 25	Or, if a) through d) are not met, LDL-C >≥ 300 mg/dL (7.8 mmol/L)
Synopsis, Inclusion/Exclusion Criteria, Exclusion Criterion 3d/ Page 6<br>Section 7.2 Exclusion Criteria, Exclusion Criterion 3d/Page 27	Oral contraceptives unless prospectively discussed with the Medical Monitor
Synopsis, Inclusion/Exclusion Criteria, Exclusion Criterion 3e/Page 6<br>Section 7.2 Exclusion Criteria, Exclusion Criterion 3e/Page 27	Fibrates unless prospectively discussed with the Medical Monitor

Protocol Section	Changed Text
Synopsis, Criteria for Evaluation Page 8	Efficacy will be assessed by measuring LDL-C, apolipoprotein (apo) B, total cholesterol, TG, high density lipoprotein cholesterol (HDL-C), non-HDL-C, and lipoprotein (a) (Lp[a]). The incremental effects of mipomersen on total cholesterol, TG, non-high density lipoprotein cholesterol (non-HDL-C), very low density lipoprotein cholesterol (VLDL-C), apo A-1, and lipoprotein subclasses may also be exploredevaluated.
Synopsis, Statistical Methods/ Page 8<br>Section 6.2.1.2 Secondary Efficacy Endpoints/Page 23<br>Section 11.5.2 Secondary Efficacy Endpoints/Page 52	Secondary efficacy endpoints include the percent change in apoB, total cholesterol, non-HDL-C, TG, HDL-C, and Lp(a) from Baseline to Week 28/ET and the percent of patients with LDL-C below LDL-C entry criteria at Week 28/ET (200 mg/dL [5.1 mmol/L] for patients with coronary heart disease [CHD], or 300 mg/dL [7.8 mmol/L] for patients without CHD). The incremental effects of mipomersen on total cholesterol, TG, non-HDL-C, VLDL-C, apo A-1, and lipoprotein subclasses may also be evaluated.
Synopsis, Statistical Methods/ Page 9<br>Section 11 Statistical Methods and Planned Analyses/Page 57	In addition, the primary efficacy analysis will be performed on the subset of patients with LDL-C of at least 200 mg/dL (5.1 mmol/L), for patients with CHD, or at least 300 mg/dL (7.8 mmol/L), for patients without CHD, representing a patient population in which LDL-C apheresis is indicated in the USA.
Synopsis, Statistical Methods/ Page 9	Percent change from Baseline to Week 28/ET in LDL-C, apo B, total cholesterol, HDL-C, non-HDL-C, TG, and Lp(a) will be compared between treatment groups using a 2-sample t-test (if data departs substantially from normality, a Wilcoxon rank sum test will be used).
Added new sentence<br>Synopsis, Statistical Methods/ Page 9<br>Section 11.5.3 Efficacy Analysis/Page 57	The percent of patients with LDL-C below LDL-C entry criteria at Week 28/ET will be compared between treatment groups using Fisher's exact test.
Synopsis, Statistical Methods/ Page 9<br>Section 11.1 Power and Determination of Sample Size/Page 57	Enrollment will be conducted such that at least 51 patients are randomized (to allow for potential exclusions from an analysis set) with LDL-C of at least 200 mg/dL (5.1 mmol/L), for patients with CHD, or at least 300 mg/dL (7.8 mmol/L), for patients without CHD, representing a patient population in which LDL-C apheresis is indicated in the USA.
Synopsis, Statistical Methods/Page 9<br>Section 6.3 Discussion of Study Design Including Choice of Control Group /Page 24<br>Section 11.1 Power and Determination of Sample Size/Page 58	Additionally, approximately 24 patients with LDL-C of at least 190 mg/dL (4.9 mmol/L) will be randomized to provide additional exposure among a slightly broader patient population. Therefore, approximately 75 patients will be randomized.
Section 4 Introduction/Page 17	Elevated LDL-C is a major risk factor for coronary heart disease (CHD) and in the last years, results of observational and interventional studies in primary and secondary prevention indicate a continuous positive relationship between coronary artery disease (CAD) risk and blood cholesterol concentrations (National Cholesterol Education Program [NCEP] Adult Treatment Panel III [ATP III]) (Grundy, 2004, *Circulation*; NCEP, 2002, *Circulation*; see Appendix 14.3).

Protocol Section	Changed Text
Section 6.3 Discussion of Study Design Including Choice of Control Group/Page 24	~~Patient randomization will be stratified based on LDL-C concentration at Screening, in order to represent a patient population in which LDL-C apheresis is indicated in the USA.~~
Section 7 Patient Population and Selection/Page 25	Approximately 50 study centers will enroll ~~approximately~~up to 75 patients in this protocol.
Section 8.4 Method of Assigning Patients to Treatment/Page 31	~~Additionally, approximately~~Up to 24 more patients ~~with LDL-C of at least 190 mg/dL (4.9 mmol/L) will~~may be randomized to provide additional ~~exposure among a slightly broader patient population~~efficacy and safety information.
Added new sentence<br>Section 11.1 Power and Determination of Sample Size/ Page 58	Should enrollment continue such that as many as 75 patients are randomized, this will yield as much as 90% power to detect a 20 percentage point difference between the 2 groups.
Deleted last sentence<br>Section 11.5.3 Efficacy Analysis/ Page 59	~~The incremental effects of mipomersen on VLDL-C, apo A-1, and lipoprotein subclasses may also be explored.~~
Section 11.7 Statistical Analysis Plan/Page 59	A formal statistical analysis plan will be developed and finalized prior to database lock for the primary efficacy endpoint ~~after~~(e.g., all patients have completed Week ~~28.~~28).
Appendix 14.3 NCEP - ATP III Guidelines for Determining >20% 10-Year Risk for CHD/Page 69	Deleted entire appendix

This protocol amendment was designed and will be conducted, recorded, and reported in compliance with the principles of Good Clinical Practice (GCP) guidelines. These guidelines are stated in U.S. federal regulations as well as "Guidance for Good Clinical Practice," International Conference on Harmonisation of Technical Requirements for Registration of Pharmaceuticals for Human Use.

I have read and agree to abide by the requirements of this amended protocol.

Investigator Signature Date

PHASE 3 CLINICAL STUDY PROTOCOL

A Prospective Randomized, Double-Blind, Placebo-Controlled Study to Assess the Safety and Efficacy of Mipomersen in Patients with Severe Hypercholesterolemia on a Maximally Tolerated Lipid-Lowering Regimen and who are not on Apheresis

Protocol Number: MIPO3500108
EudraCT: 2008-006020-53
Protocol Amendment 1: 29 October 2008
Original Protocol: 8 October 2008

Sponsor:

Genzyme Europe BV
Gooimeer 10
1411 DD Naarden
The Netherlands

Genzyme Corporation
500 Kendall Street
Cambridge, MA 02142
USA

Medical Monitor/ Key Study Contacts:

Ajay Duggal, MBChB, MRCP, DipPharmMed
Senior Medical Director
Genzyme Europe Research
310 Cambridge Science Park
Milton Road
Cambridge, UK
e-mail:
Ajay.Duggal@Genzyme.com
tel: +44 1223 394 050
fax: +44 1223 394191

Joanne M. Donovan, MD, PhD
Vice President, Clinical Research
Genzyme Corporation
USA
e-mail:
Joanne.DonovanMD@Genzyme.com

Statistician:

Scott Chasan-Taber, PhD
Senior Director, Biostatistics
Genzyme Corporation
USA

This protocol was designed and will be conducted, recorded, and reported in compliance with the principles of Good Clinical Practice (GCP) guidelines as well as in accordance with all national, state and local laws of the appropriate regulatory authorities. These guidelines are stated in “Guidance for Good Clinical Practice,” International Conference on Harmonisation of Technical Requirements for Registration of Pharmaceuticals for Human Use.

I have read and agree to abide by the requirements of this protocol.

Investigator printed name …………………………………………

Investigator Signature_______________________________________Date

Signature Page for Sponsor's Representative

The following have reviewed and approved the protocol entitled, "MIPO3500108: A Prospective Randomized, Double-Blind, Placebo-Controlled Study to Assess the Safety and Efficacy of Mipomersen in Patients with Severe Hypercholesterolemia on a Maximally Tolerated Lipid-Lowering Regimen and who are not on Apheresis."

Joanne M Donovan	29 October 08
Joanne M. Donovan, MD, PhD<br>Vice President, Clinical Research<br>Genzyme Corporation<br>USA	Date

1 SYNOPSIS

NAME OF COMPANY	SUMMARY TABLE	FOR NATIONAL AUTHORITY USE ONLY:
Genzyme Corporation<br>500 Kendall Street<br>Cambridge, MA 02142<br>USA<br><br>Genzyme Europe BV<br>Gooimeer 10<br>1411 DD Naarden<br>The Netherlands<br><br>**NAME OF FINISHED PRODUCT**<br>mipomersen sodium injection/ISIS 301012 Injection, 200 mg/mL, 1.0 mL<br>**NAME OF ACTIVE INGREDIENT**<br>mipomersen sodium/ISIS 301012 Drug Substance	Referring to Part ……<br>of the Dossier:<br>Volume:<br>Page:<br>Reference:	

TITLE:
A Prospective Randomized, Double-Blind, Placebo-Controlled Study to Assess the Safety and Efficacy of Mipomersen in Patients with Severe Hypercholesterolemia on a Maximally Tolerated Lipid-Lowering Regimen and who are not on Apheresis

PROTOCOL NO.:
MIPO3500108

INVESTIGATOR STUDY CENTERS:
Approximately 50 study centers will enroll patients in this protocol.

OBJECTIVES:
To compare the safety and efficacy of 26 weekly, subcutaneous (SC) injections of mipomersen (200 mg) against placebo in treating severely hypercholesterolemic patients who are on a maximally tolerated lipid-lowering regimen and who are not on apheresis.

METHODOLOGY:
This is a prospective, randomized, double-blind, placebo-controlled, parallel-group, multicenter Phase 3 study to investigate the safety and efficacy of mipomersen to treat severe hypercholesterolemia. Approximately 51 severely hypercholesterolemic adult patients will be randomized, who are on a maximally tolerated lipid-lowering regimen and not on apheresis, with low density lipoprotein cholesterol (LDL-C) $\geq$ 200 mg/dL (5.1 mmol/L) for patients with coronary heart disease (CHD), or at least 300 mg/dL (7.8 mmol/L) for patients without CHD. This represents a patient population in which LDL-C apheresis is indicated in the United States of America (USA). As many as 24 additional patients may be randomized to the study, for a total of 75 patients, to provide up to 90% power for efficacy and to provide further safety information.

Patients will be randomized in a 2:1 ratio to receive mipomersen or placebo, respectively. Mipomersen or a matching volume of placebo will be administered weekly via SC injection for 26 doses.

Following the treatment period and Week 28 evaluation, patients may elect to continue in an open-label extension study, if one is available, pending approval by the Ethics Committee and the appropriate regulatory authority. Patients who choose not to participate or are ineligible for the open-label extension study will continue post-treatment follow-up in the current protocol.

The study will be conducted in compliance with International Conference on Harmonisation (ICH), Good Clinical Practice (GCP) guidelines as well as in accordance with all national, state and local laws of the appropriate regulatory authorities. The duration of the study, including completing patient

NAME OF COMPANY	SUMMARY TABLE	FOR NATIONAL AUTHORITY USE ONLY:
Genzyme Corporation<br>500 Kendall Street<br>Cambridge, MA 02142<br>USA<br><br>Genzyme Europe BV<br>Gooimeer 10<br>1411 DD Naarden<br>The Netherlands<br><br>**NAME OF FINISHED PRODUCT**<br>mipomersen sodium injection/ISIS 301012 Injection, 200 mg/mL, 1.0 mL<br>**NAME OF ACTIVE INGREDIENT**<br>mipomersen sodium/ISIS 301012 Drug Substance	Referring to Part ……<br>of the Dossier:<br>Volume:<br>Page:<br>Reference:	

enrollment, is expected to be approximately 18 months. Each patient will participate in a ≤ 4-week screening period and a 26-week treatment period. All patients, including those who discontinue prematurely and have received ≥ 1 dose of study drug, should be followed for safety for 24 weeks after their last dose of study drug as part of the post-treatment evaluation period. The end of study is defined as the last patient's last visit.

SAFETY MONITORING RULES:

An independent data monitoring committee (DMC) will provide an ongoing, expert, independent review of safety data to assure that the risks to study patients are minimized.

If any of the stopping criteria described in this protocol are met and are confirmed, the patient will be permanently discontinued from further treatment with study drug (mipomersen or placebo), evaluated fully in consultation with the Medical Monitor, and will be entered into the post-treatment evaluation portion of the study. In general, patients who do not meet the stopping rules based upon retest may continue dosing. However, the Investigator and the Medical Monitor should confer as to whether additional close monitoring of the patient is appropriate.

NUMBER OF PATIENTS:
Between 51 and 75 patients will be randomized into this study.

INCLUSION/EXCLUSION CRITERIA:
Inclusion Criteria:

Patients must fulfill all of the following criteria:

1. Age ≥ 18 years
2. Fasting LDL-C ≥ 200 mg/dL (5.1 mmol/L) and triglycerides (TG) < 350 mg/dL (4.0 mmol/L) at Screening
3. Presence of at least 1 of the following criteria for coronary disease:
   a) Myocardial infarction (MI)
   b) Percutaneous coronary intervention (PCI) or coronary artery bypass graft (CABG)
   c) Coronary artery disease (CAD) documented by angiography or any other accepted imaging technique
   d) If 1 or more of criteria a through c are not met: a positive exercise test (≥ 1 mm ST-depression at maximal exercise or test terminated because of angina) or a perfusion defect, e.g., thallium or single photon emission computed tomography

NAME OF COMPANY<br>Genzyme Corporation<br>500 Kendall Street<br>Cambridge, MA 02142<br>USA<br><br>Genzyme Europe BV<br>Gooimeer 10<br>1411 DD Naarden<br>The Netherlands<br><br>**NAME OF FINISHED PRODUCT**<br>mipomersen sodium injection/ISIS 301012 Injection, 200 mg/mL, 1.0 mL<br>**NAME OF ACTIVE INGREDIENT**<br>mipomersen sodium/ISIS 301012 Drug Substance	**SUMMARY TABLE**<br>Referring to Part ……<br>of the Dossier:<br>Volume:<br>Page:<br>Reference:	**FOR NATIONAL AUTHORITY USE ONLY:**

(SPECT)

e) Or, if a) through d) are not met, LDL-C ≥ 300 mg/dL (7.8 mmol/L)

4. On a stable, maximally tolerated lipid-lowering regimen and expected to remain on it through Week 28 (must satisfy all criteria):

   a) A statin at a maximally tolerated dose per Investigator judgment, for at least 8 weeks prior to Screening.

   b) A stable low-fat diet (e.g., National Cholesterol Education Program [NCEP]-Adult Treatment Panel [ATP] III therapeutic lowering cholesterol [TLC] or equivalent) beginning at least 12 weeks prior to the first dose of study drug

   c) A medication from an additional class of hypolipidemic agents, per Investigator’s judgment (e.g., bile acid sequestrants, niacin/nicotinic acid, fibrates) for at least 8 weeks prior to Screening.

5. Body mass index (BMI) ≤ 40 kg/m^2 with weight stable (± 4 kg) for > 6 weeks prior to Screening per patient report.

6. Satisfy 1 of the following:

   a) Females: Non-pregnant and non-lactating; surgically sterile, postmenopausal, or patient or partner compliant with an acceptable contraceptive regimen for 4 weeks prior to Screening, during the treatment phase, and 24 weeks after the last study drug dose

   b) Males: Surgically sterile or patient or partner is utilizing an acceptable contraceptive method during the treatment phase and 24 weeks after the last study drug dose

7. Given informed consent

Exclusion Criteria:

1. Any of the following diagnoses, conditions, or prior treatments:

   a) MI, PCI, CABG, cerebrovascular accident (CVA), unstable angina or acute coronary syndrome within 24 weeks of Screening

   b) Presence of clinically significant arrhythmias, implantable pacemakers or automatic implantable cardioverter defibrillators (AICDs), or currently taking any medication for arrhythmias

   c) Type 1 diabetes mellitus

NAME OF COMPANY<br>Genzyme Corporation<br>500 Kendall Street<br>Cambridge, MA 02142<br>USA<br><br>Genzyme Europe BV<br>Gooimeer 10<br>1411 DD Naarden<br>The Netherlands<br><br>**NAME OF FINISHED PRODUCT**<br>mipomersen sodium injection/ISIS 301012 Injection, 200 mg/mL, 1.0 mL<br>**NAME OF ACTIVE INGREDIENT**<br>mipomersen sodium/ISIS 301012 Drug Substance	**SUMMARY TABLE**<br>Referring to Part ……<br>of the Dossier:<br>Volume:<br>Page:<br>Reference:	**FOR NATIONAL AUTHORITY USE ONLY:**

d) New York Heart Association (NYHA) functional classification III or IV heart failure

e) Hypertension, systolic blood pressure (BP) ≥ 160 mmHg, or diastolic BP ≥ 95 mmHg at Screening (despite antihypertensive medication/therapy)

f) Active infection requiring systemic antimicrobial therapy

g) Positive test for human immunodeficiency virus (HIV) or hepatitis B or C at Screening

h) Any uncontrolled condition that may predispose the patient to secondary hyperlipidemia such as uncontrolled hypothyroidism.

i) Malignancy within 5 years, except for basal or squamous cell carcinoma of the skin that has been adequately treated

j) Clinically significant hepatic or renal disease or Gilbert's syndrome

k) Apheresis within 3 months prior to Screening or expected to start apheresis during the treatment phase

2. The following laboratory values at Screening:

   a) Serum creatine phosphokinase (CPK) ≥ 3 x upper limit of normal (ULN)

   b) Alanine aminotransferase (ALT) levels > 1.5 x ULN

   c) Serum creatinine > 0.1 mg/dL (> 8.8 µmol/L) above ULN for women, or > 0.2 mg/dL (> 17.7 µmol/L) above ULN for men

   d) Proteinuria (> 1+ on dipstick, confirmed on retest, with further confirmation by quantitative total urine protein > 1.0 g/24 hr)

   e) Total Bilirubin > 1.0 x ULN

   f) Glycosylated hemoglobin A (HbA_{1C}) > 8.0%

3. Use of the following medications within 12 weeks of Screening:

   a) Medications that may affect lipids except those allowed per the protocol, including but not limited to Cholestin™ (also known as red yeast rice, or monascus purpureus extract)

   b) Anti-obesity medications

   c) Chronic systemic corticosteroids or anabolic agents except for replacement therapy

   d) Oral contraceptives

4. Use of the following medications unless a stable dose regimen was used for at least

**NAME OF COMPANY**<br>Genzyme Corporation<br>500 Kendall Street<br>Cambridge, MA 02142<br>USA<br><br>Genzyme Europe BV<br>Gooimeer 10<br>1411 DD Naarden<br>The Netherlands<br><br>**NAME OF FINISHED PRODUCT**<br>mipomersen sodium injection/ISIS 301012 Injection, 200 mg/mL, 1.0 mL<br>**NAME OF ACTIVE INGREDIENT**<br>mipomersen sodium/ISIS 301012 Drug Substance	**SUMMARY TABLE**<br>Referring to Part ……<br>of the Dossier:<br>Volume:<br>Page:<br>Reference:	**FOR NATIONAL AUTHORITY USE ONLY:**

12 weeks prior to Screening and the dose and regimen are expected to remain stable until Week 28:

a) Oral anticoagulants (e.g., warfarin)

b) Hormone replacement therapy

c) Diabetes medications including but not limited to sulfonylureas, metformin and glitazones, with the exception of changes of ± 10 units of insulin.

d) Antiviral therapy for herpes simplex virus (HSV)

5. Treatment with another investigational drug, biological agent, or device within 4 weeks of Screening or 5 half-lives of study agent, whichever is longer
6. Recent history of, or current, drug or alcohol abuse, or unwillingness to limit alcohol consumption for the entire duration of the study, including follow-up
7. Any disorders that would limit study participation or unwillingness to comply with study procedures, including follow-up, as specified by this protocol, or unwillingness to cooperate fully with the Investigator
8. Have any other medical conditions that, in the opinion of the Investigator, would make the patient unsuitable for enrollment, or could interfere with the patient participating in or completing the study

DOSE/ROUTE/REGIMEN:
Patients will be randomized in a 2:1 ratio (i.e., mipomersen: placebo)

Mipomersen: 1-mL injections of 200 mg of mipomersen (ISIS 301012) will be administered weekly for 26 weeks.

Injections will be administered as SC injections into the outer area of the upper arm, medial thigh region, or abdomen. The first 3 doses must be supervised by a health professional, including study center personnel or home health nurse. Thereafter, patients will be given the option to self-administer study drug injections unsupervised with the exception of doses on clinic visit days.

A specific administration schedule will be used in this study in order to gain some understanding about the patient's preference of injection site and the reason for the choice. For the first 3 injections, the injection site will be specified for all patients. The first injection will be in the outer area of the upper arm, the second injection will be in the medial thigh region, and the third injection will be in the abdomen. Based on the patient's experience from the first 3 doses, each will be allowed to choose the injection site for their remaining injections.

**NAME OF COMPANY**<br>Genzyme Corporation<br>500 Kendall Street<br>Cambridge, MA 02142<br>USA<br><br>Genzyme Europe BV<br>Gooimeer 10<br>1411 DD Naarden<br>The Netherlands<br><br>**NAME OF FINISHED PRODUCT**<br>mipomersen sodium injection/ISIS 301012<br>Injection, 200 mg/mL, 1.0 mL<br>**NAME OF ACTIVE INGREDIENT**<br>mipomersen sodium/ISIS 301012 Drug Substance	**SUMMARY TABLE**<br>Referring to Part ……<br>of the Dossier:<br>Volume:<br>Page:<br>Reference:	**FOR NATIONAL AUTHORITY USE ONLY:**
**REFERENCE TREATMENT:**<br>Adult hypercholesterolemic patients receiving either mipomersen or placebo as part of the study protocol will be on a maximally tolerated lipid lowering regimen.<br><br>Placebo: 1-mL weekly injections of placebo (i.e., vehicle consisting of 9 mg of sodium chloride, 0.004 mg of riboflavin, filled to (QS) 1 mL with water for injection) for 26 weeks.<br><br>Injections will be administered using the same instructions as provided for mipomersen.		
**CRITERIA FOR EVALUATION:**<br>Safety:<br><br>Safety will be evaluated in terms of all treatment-emergent adverse events (AEs) and serious adverse events (SAEs), physical examination findings, vital signs parameters and clinical laboratory parameters. AEs will be categorized using a standardized coding dictionary (e.g., Medical Dictionary for Regulatory Activities [MedDRA]). Clinical laboratory tests including chemistry, hematology with differential, and urinalysis will be tabulated.<br><br>Efficacy:<br><br>Efficacy will be assessed by measuring LDL-C, apolipoprotein (apo) B, and lipoprotein (a) (Lp[a]). The incremental effects of mipomersen on total cholesterol, TG, non-high density lipoprotein cholesterol (non-HDL-C), very low density lipoprotein cholesterol (VLDL-C), apo A-1, and lipoprotein subclasses may also be evaluated.		
**STATISTICAL METHODS:**<br>Primary Efficacy Endpoint<br><br>The primary efficacy endpoint is the percent change in LDL-C from Baseline to Week 28/Early Termination (ET represents the observation closest to 2 weeks after last dose among patients who early terminate study medication dosing).<br><br>Secondary Efficacy Endpoints<br><br>Secondary efficacy endpoints include the percent change in apoB, and Lp(a) from Baseline to Week 28/ET and the percent of patients with LDL-C below LDL-C entry criteria at Week 28/ET (200 mg/dL (5.1 mmol/L) for patients with coronary heart disease (CHD), or 300 mg/dL (7.8 mmol/L) for patients without CHD). The incremental effects of mipomersen on total cholesterol, TG, non-HDL-C, VLDL-C, apo A-1, and lipoprotein subclasses may also be evaluatedd.<br><br>Statistical Analyses		

NAME OF COMPANY<br>Genzyme Corporation<br>500 Kendall Street<br>Cambridge, MA 02142<br>USA<br><br>Genzyme Europe BV<br>Gooimeer 10<br>1411 DD Naarden<br>The Netherlands<br><br>**NAME OF FINISHED PRODUCT**<br>mipomersen sodium injection/ISIS 301012 Injection, 200 mg/mL, 1.0 mL<br>**NAME OF ACTIVE INGREDIENT**<br>mipomersen sodium/ISIS 301012 Drug Substance	**SUMMARY TABLE**<br>Referring to Part ……<br>of the Dossier:<br>Volume:<br>Page:<br>Reference:	**FOR NATIONAL AUTHORITY USE ONLY:**

Efficacy Analyses:

The primary efficacy analysis will be performed on the Full Analysis Set, which represents the practically-feasible intent-to-treat (ITT) population as delineated in ICH Guideline E9, and will be comprised of all patients who receive at least 1 injection and have at least 1 post-Baseline LDL-C measurement.

Percent change from Baseline to Week 28/ET in LDL-C, apo B, and Lp(a) will be compared between treatment groups using a 2-sample t-test (if data departs substantially from normality, a Wilcoxon rank sum test will be used). The analysis will take place after all patients have completed Week 28 and the database has been locked. The percent of patients with LDL-C below LDL-C entry criteria at Week 28/ET will be compared between treatment groups using Fisher's exact test.

Safety Analyses:

The safety analyses will be performed on the Safety Set defined as all patients who receive at least 1 injection. Treatment duration and amount of study drug received will be summarized by treatment group, as will concomitant medications and treatments. Treatment-emergent AEs will be summarized by body system, preferred term, severity and relationship to the study procedures or treatments. Additionally listings of SAEs and AEs leading to discontinuation will be generated. Clinical laboratory evaluations, vital signs, electrocardiogram (ECG), and physical examination findings will be tabulated by treatment group. In addition, the number of patients who experience abnormalities in clinical laboratory evaluations will be summarized by treatment group.

Plasma concentrations and elimination half-life of mipomersen will be summarized over time by individual patient and by treatment group

Power and Sample Size

Based upon prior clinical trial experience with mipomersen, it is estimated that the standard deviation of the percent change in LDL-C is approximately 22%. With 45 randomized patients (15 patients in the control group and 30 patients in the mipomersen-treated group), there would be 80% power to detect a 20 percentage point difference between the 2 groups. Enrollment will be conducted such that at least 51 patients are randomized to allow for potential exclusions from an analysis set.

2 TABLE OF CONTENTS

1 SYNOPSIS ... 3
2 TABLE OF CONTENTS ... 10
3 ABBREVIATIONS AND TERMS ... 14
4 INTRODUCTION ... 17
4.1 ApoB-100 as a Therapeutic Target for Hypercholesterolemia ... 18
4.2 Mipomersen ... 19
4.2.1 Mechanism of Action ... 19
4.2.2 Chemistry ... 19
4.3 Summary of Benefits and Risks ... 20
5 STUDY OBJECTIVES ... 21
6 INVESTIGATIONAL PLAN ... 22
6.1 Study Design ... 22
6.2 Endpoints ... 24
6.2.1 Efficacy Endpoints ... 24
6.2.1.1 Primary Efficacy Endpoint ... 24
6.2.1.2 Secondary Efficacy Endpoints ... 24
6.2.2 Safety Endpoints ... 24
6.3 Discussion of Study Design Including Choice of Control Group ... 24
7 PATIENT POPULATION AND SELECTION ... 26
7.1 Inclusion Criteria ... 26
7.2 Exclusion Criteria ... 27
7.3 Patient Withdrawal ... 28
8 TREATMENTS ... 30
8.1 Treatments Administered ... 30
8.2 Investigational Product ... 31
8.2.1 Packaging and Labeling ... 31
8.2.2 Study Drug Preparation ... 31
8.2.3 Drug Storage ... 31
8.3 Prior and Concomitant Medications ... 31
8.4 Method of Assigning Patients to Treatment ... 32
8.5 Dose Selection ... 32
8.6 Blinding and Randomization ... 32
8.6.1 Unblinding ... 33
9 EFFICACY AND SAFETY VARIABLES ... 34
9.1 Efficacy and Safety Measurements Assessed and Study Flowchart ... 34
9.1.1 Screening Assessments ... 39
9.1.1.1 Medical History, Physical Examinations, 12-Lead Electrocardiogram ... 39
9.1.1.2 Clinical Laboratory Evaluations and Therapeutic Lifestyle Changes ... 39
9.1.2 Treatment Period Assessments ... 40
9.1.2.1 Magnetic Resonance Imaging and Computed Tomography ... 41
9.1.2.2 Clinical Laboratory Evaluations ... 41

9.1.2.3 Demographics, Vital Signs, Body Weight, and Anthropometric Measurements ... 44
9.1.2.4 Pharmacokinetic Trough Levels ... 44
9.1.2.5 Electrocardiogram ... 44
9.1.2.6 Physical Examination ... 45
9.1.2.7 Concomitant Medications ... 45
9.1.2.8 Injection Site Reaction Assessment ... 45
9.1.3 Post-Treatment Evaluation Period Assessments ... 45
9.2 Adverse Events ... 46
9.2.1 Definition of Adverse Events ... 46
9.2.2 Definition of Serious Adverse Events ... 46
9.2.3 Evaluation of Adverse Events and Serious Adverse Events ... 47
9.2.3.1 Severity of Adverse Events ... 47
9.2.3.2 Action Taken/Medication or Therapy Given ... 48
9.2.3.3 Outcome of Adverse Events ... 48
9.2.3.4 Relationship to Study Treatment ... 49
9.2.3.5 Clinical Laboratory Tests ... 49
9.2.3.6 Dosing Errors ... 49
9.2.4 Adverse Experience and Serious Adverse Experience Reporting ... 50
9.2.4.1 Adverse Experience Reporting ... 50
9.2.4.2 Serious Adverse Event Reporting ... 50
9.2.5 Pregnancy Reporting ... 51
9.2.6 Stopping Rules and Safety Monitoring ... 52
9.2.6.1 Confirmation Guidance ... 52
9.2.6.2 Stopping Rule Guidance ... 53
9.2.6.3 Liver Chemistry Rules ... 53
9.2.6.4 Safety Monitoring Rules for Renal Function Test Results ... 55
9.2.6.5 Safety Monitoring Rule for Platelet Count Results ... 55
9.2.6.6 Safety Monitoring Rules for Coagulation Parameters ... 55
9.2.6.7 Safety Monitoring Rule for Constitutional Symptoms ... 55
9.2.6.8 Restriction on the Lifestyle of Patients ... 55
9.2.7 Summary of Risks ... 56
10 DATA COLLECTION, QUALITY ASSURANCE, AND MANAGEMENT ... 56
10.1 Recording of Data ... 56
10.2 Data Quality Assurance ... 56
10.3 Data Management ... 57
11 STATISTICAL METHODS AND PLANNED ANALYSES ... 58
11.1 Power and Determination of Sample Size ... 58
11.2 Demographics and Baseline Characteristics ... 59

11.3 Patient Accountability ... 59
11.4 Study Treatment Usage and Compliance ... 59
11.5 Efficacy ... 59
11.5.1 Primary Efficacy Endpoint ... 59
11.5.2 Secondary Efficacy Endpoints ... 59
11.5.3 Efficacy Analysis ... 59
11.6 Safety ... 60
11.6.1 Safety Endpoints ... 60
11.6.2 Safety Analysis ... 60
11.7 Statistical Analysis Plan ... 60
12 SPECIAL REQUIREMENTS AND PROCEDURES ... 61
12.1 Institutional and Ethical Review ... 61
12.2 Changes to the Conduct of the Study or Protocol ... 61
12.3 Investigator's Responsibilities ... 61
12.3.1 Patient Informed Consent ... 61
12.3.2 Electronic Case Report Forms ... 62
12.3.3 Record Retention ... 62
12.3.4 Patient Discontinuation ... 63
12.3.5 Study or Site Termination ... 63
12.3.6 Monitoring ... 63
12.3.7 Materials Control ... 63
12.3.7.1 Receipt of Clinical Supplies ... 63
12.3.7.2 Disposition of Unused Clinical Supplies ... 64
12.3.8 Warnings, Precautions, Contraindications ... 64
12.3.9 Clinical Study Report ... 64
12.3.10 Disclosure of Data ... 64
13 REFERENCES ... 65
14 APPENDICES ... 67
14.1 NCEP – ATP III Therapeutic Lifestyle Changes ... 68
14.2 Lipoprotein Testing ... 69
14.3 Pregnancy Notification Forms ... 70

LIST OF IN-TEXT TABLES

Table 9-1 Schedule of Study Events ... 35
Table 9-2 List of Screening Clinical Laboratory Evaluations ... 40
Table 9-3 List of Treatment Period Clinical Laboratory Evaluations ... 43
Table 9-4 Contact List for Serious Adverse Event Reporting ... 51
Table 14-1 Nutrient Composition of the TLC Diet ... 68
Table 14-2 Lipoprotein Testing ... 69

LIST OF IN-TEXT FIGURES

Figure 4-1 Design of Chimeric 2´-MOE Phosphorothioate Oligonucleotides (MOE-Gapmer) ... 20

Figure 6-1 Study Design ... 24

3 ABBREVIATIONS AND TERMS

AE	Adverse event
AICD	Automatic implantable cardioverter defibrillator
ALT	Alanine aminotransferase (SGPT)
ANA	Antinuclear antibody
apo	Apolipoprotein
aPTT	Activated Partial Thromboplastin Time
ASO	Antisense oligonucleotide
AST	Aspartate aminotransferase (SGOT)
ATP	Adult Treatment Panel
BMI	Body Mass Index
BP	Blood pressure
BUN	Blood urea nitrogen
C3	Complement
CABG	Coronary artery bypass graft
CAD	Coronary artery disease
CHD	Coronary heart disease
C_{min}	Minimum plasma concentration
CMV	Cytomegalovirus
CPK	Creatine phosphokinase
eCRF	Electronic Case Report Form
CS	Clinically significant
CT	Computed tomography
CTTC	Cholesterol Treatment Trialists' Collaboration
CVA	Cerebrovascular accident
DMC	Data Monitoring Committee
DNA	Deoxyribonucleic acid
EBV	Epstein-Barr Virus
ECG	Electrocardiogram
EDC	Electronic data capture
ESR	Erythrocyte sedimentation rate
ET	Early termination
GCP	Good Clinical Practice
HAV	hepatitis A virus
HBsAg	Hepatitis B surface antigen

hCG	Human chorionic gonadotropin
HCV	hepatitis C virus
HDL-C	High density lipoprotein cholesterol
HEENT	Head, ears, eyes, nose, throat
HIV	Human immunodeficiency virus
HbA_{1c}	Glycosylated hemoglobin A
HMG-CoA	3-hydroxy-3-methylglutaryl coenzyme A
hsCRP	High-sensitivity C-reactive protein
HSV	Herpes simplex virus
ICH	International Conference on Harmonisation
IEC	Independent Ethics Committee
IgG	Immunoglobulin G
IgM	Immunoglobulin M
INR	International normalized ratio (for anticoagulant monitoring)
IRB	Institutional Review Board
ISR	Injection site reaction
ITT	Intent-to-treat
IVRS	Interactive voice response system
LDL	Low density lipoprotein
LDL-C	Low density lipoprotein cholesterol
LFT	Liver function test
Lp(a)	Lipoprotein a
MCV	Mean corpuscular volume
MedDRA	Medical Dictionary for Regulatory Activities
MI	Myocardial Infarction
MOE	O-(2-Methoxyethyl)-D-Ribose
MRI	Magnetic resonance imaging
mRNA	Messenger Ribonucleic Acid
NCEP	National Cholesterol Education Program
NCS	Not Clinically Significant
NEPTUNE II	National Cholesterol Education (NCEP) Program Evaluation Project Utilizing Novel E-Technology II
NYHA	New York Heart Association
P/C	Urine protein to creatinine ratio
PK	Pharmacokinetic(s)
PT	Prothrombin Time

PCI	Percutaneous coronary intervention
PTT	Partial thromboplastin time
QA	Quality Assurance
QRS	Part of ECG wave representing ventricular depolarization
QS	A sufficient quantity to make
RR	Inter-beat interval
SAE	Serious Adverse Event
SC	Subcutaneous
SPECT	Single photon emission computed tomography
Study Drug	mipomersen or placebo
T_4	Thyroxine
TG	Triglycerides
TLC	Therapeutic Lifestyle Changes
TSH	Thyroid Stimulating Hormone
UAA	Translation stop codon
ULN	Upper Limit of Normal
USA	United States of America
VLDL	Very low density lipoprotein
VLDL-C	Very low density lipoprotein cholesterol
WBC	White blood cells
WHR	Waist-to-hip ratio

4 INTRODUCTION

Hypercholesterolemia is a common condition that in its untreated form is categorized by a broad range of elevated low density lipoprotein cholesterol (LDL-C) concentrations. Its etiology is mixed and although environmental contributors such as diet play a role, there is a strong genetic component especially in those patients who have LDL-C concentrations in the higher ranges. Monogenic forms of hypercholesterolemia include the different varieties of familial hypercholesterolemia, but there are also thought to be many polygenic contributors.

Elevated LDL-C is a major risk factor for coronary heart disease (CHD) and in the last years, results of observational and interventional studies in primary and secondary prevention indicate a continuous positive relationship between coronary artery disease (CAD) risk and blood cholesterol concentrations (National Cholesterol Education Program [NCEP] Adult Treatment Panel III [ATP III]) (Grundy, 2004, *Circulation*; NCEP, 2002, *Circulation)*. The strong association between LDL-C concentrations and CHD risk is attributed to the central role of LDL-C in the development and progression of atherosclerotic lesions, the underlying cause of CHD. Low density lipoprotein is a major source of lesion cholesterol and stimulates inflammatory processes involved in lesion development (Davis, 2001, *Arterioscler Thromb Vasc Bio.*; Hulthe, 2002, *Arterioscler Thromb Vasc Biol*; Skalen, 2002, *Nature*). In addition reducing LDL-C is associated with improved outcomes in many studies. A meta-analysis of many randomized controlled outcome studies indicated that a 1 mmol/L reduction in LDL-C resulted in a 23% reduction in vascular events across a range of end of treatment LDL-C concentrations (Cholesterol Treatment Trialists' Collaboration [CTTC], 2005, *Lancet*). Because of the increased risk of elevated LDL-C plasma concentrations, LDL-C reduction has been the principal goal of CHD prevention strategies (Knopp, 1999, *N Engl J Med*; Gotto, 2002, *Am J Med*). The goals for lipid-lowering therapy were established by the NCEP and were updated most recently in 2004 (Grundy, 2004, *Circulation*). The NCEP guidelines define 3 major categories of risk for clinical events and provide risk-based LDL-C targets. Those at highest risk are patients with CHD or CHD-risk equivalent conditions and should be treated to LDL-C targets < 100 mg/dL (<2.6 mmol/L) or lower (< 70 mg/dL [< 1.8 mmol/L]). CHD-risk equivalent conditions include diabetes, peripheral vascular disease, abdominal aortic aneurysm, symptomatic carotid artery disease, metabolic syndrome, and multiple risk factors that confer a 10-year risk for CHD events > 20%.

There are currently 5 classes of approved therapeutic agents for hyperlipidemia, all of which are administered orally. These include the bile-acid sequestrants, fibrates, niacin, statins, and cholesterol absorption inhibitors. The choice of therapy is individualized to accommodate the needs of the patient although the statins are the overwhelming therapy of choice due to the extent of LDL-C reductions achieved and the wealth of evidence of benefit. The statins

are competitive inhibitors of 3-hydroxy-3-methylglutaryl coenzyme A (HMG-CoA) reductase, the rate-limiting step in cholesterol biosynthesis. Administration of these drugs also leads to increased hepatic low density lipoprotein (LDL) receptor expression and enhanced cholesterol clearance. In addition, the statins appear to have pleiotropic effects, including anti-inflammatory, anti thrombotic, and anti-proliferative properties that may prevent plaque growth and rupture. Despite the availability and clinical effectiveness of these agents, recent data (Sueta, 2003, *J Cardiac Failure*) indicate that a significant proportion of patients do not attain target LDL-C values and thus that cardiovascular mortality rates remain high, especially in high-risk patients. Studies such as the NCEP Program Evaluation Project Utilizing Novel E-Technology II (NEPTUNE II) show that a large number of high-risk patients are not sufficiently treated. Of patients classified as very high risk, only 18% were at the optional therapeutic LDL-C goal of < 70 mg/dL (< 1.8 mmol/L) with 60% achieving concentrations < 100 mg/dL (2.6 mmol/L) (Davidson, 2005, *Am J Cardiol*). In individuals with CHD or CHD equivalent who did not meet their targets, average LDL-C concentrations were 125 mg/dL (3.2 mmol/L). More stringent LDL-C lowering goals, coupled with a clearer understanding of the molecular basis of various types of dyslipidemia, underscore the need for the development of new therapeutics.

4.1 ApoB-100 as a Therapeutic Target for Hypercholesterolemia

Apolipoproteins (apo) are a family of structural proteins present on the surface of lipoproteins that are important for the regulation of lipid transport and metabolism. They were named in an arbitrary alphabetical order and include apoA, B, C and E. ApoB exists in the plasma in 2 main isoforms, apoB-48 and apoB-100. In man, the apoB-48 is synthesized exclusively by the gut while apoB-100 is synthesized by the liver (Powell, 1987, *Cell*). The intestinal (B-48) and hepatic (B-100) forms of apoB are produced from a single gene and a single messenger ribonucleic acid (mRNA) transcript. This results from an organ-specific apoB mRNA editing enzyme (apobec-1), which generates a stop codon within the mRNA, changing CAA at position 2153 base pairs to a translation stop codon (UAA) (Chen, 1987, *Science*). Translation of the edited transcript yields a truncated protein product similar in composition to the N-terminal half of apoB 100. From structural studies, it is thought that apoB-48 represents the amino terminal 47% of apoB 100.

ApoB-100 is the major apolipoprotein of very low density lipoprotein (VLDL), intermediate-density lipoprotein, and LDL, comprising approximately 30%, 60%, and 95% of the protein in these lipoproteins, respectively. ApoB-100 is essential for the assembly and secretion of VLDL from the liver. Lipids such as triglycerides (TG) and cholesterol are packaged with apoB-100 and other phospholipids into VLDL, which in turn is secreted into the plasma, where additional apolipoproteins are added. Thus, inhibition of apoB-100 would be expected

to impair VLDL synthesis and result in lower concentrations of LDL-C. Indeed, individuals who are unable to translate full length apoB-100 have plasma concentrations of apoB and LDL-C that are 25% to 50% of normal (Sankatsing, 2005, *Arterioscler Thromb Vasc Biol*).

4.2 Mipomersen

4.2.1 Mechanism of Action

Antisense oligonucleotides (ASO) are designed to be complementary to a segment of a target mRNA. ASO binding to mRNA is dependent on Watson and Crick base-pairing rules and is, therefore, highly specific for the target sequence. Binding of the ASO to its cognate mRNA results in RNase H-mediated degradation of the cognate mRNA thus inhibiting mRNA translation into protein. First generation oligonucleotides are nearly identical to endogenous deoxyribonucleic acid (DNA), differing only in the replacement of sulfur for 1 of the non-bridging oxygens in each of the phosphate linkages of DNA to form phosphorothioate linkages. Phosphorothioate linkages are more resistant than phosphodiester linkages to nuclease degradation and thus make the ASOs more resistant to metabolism by nucleases. Second generation ASOs have the same phosphorothioate linkages, but in addition, the nucleotides are modified at the 2' position of the ribose with 2'-O-(2-methoxyethyl)-D-ribose (2'-MOE). The MOE modification (1) increases affinity towards the cognate mRNA, (2) increases resistance to exonucleases and endonucleases (thereby increasing tissue half-life), and (3) ameliorates some of the high-dose toxicities associated with first generation ASOs. Increased affinity results in potency that is increased compared to first-generation ASOs.

4.2.2 Chemistry

Mipomersen is an antisense drug targeted to human apoB-100, the principal apolipoprotein of atherogenic LDL and its metabolic precursor, VLDL. Mipomersen is complementary to the coding region of the mRNA for apoB-100, binding by Watson and Crick base pairing. The hybridization (binding) of mipomersen to the cognate mRNA results in RNase H-mediated degradation of the cognate mRNA thus inhibiting translation of the apoB-100 protein.

Mipomersen is the nonadecasodium salt of a 20-base (20-mer) phosphorothioate oligonucleotide. Each of the 19 internucleotide linkages is a 3′-O to 5′-O phosphorothioate diester. Ten of the 20 sugar residues are 2-deoxy-D-ribose, the remaining 10 are 2'-MOE. The 2′-MOE modification improves binding affinity for the target mRNA while increasing stability against nuclease-mediated degradation relative to 2′-deoxyribonucleosides. However, since the 2′ MOE modification reduces RNase H activity, a chimeric oligonucleotide strategy was employed in which 2′-deoxyribonucleosides that support RNase H activity are flanked by nuclease resistant 2′-MOE ribonucleosides. These modifications are commonly known as second-generation ASO, and the chimeric design of mipomersen improves its pharmacologic profile (relative to 2′ deoxyribonucleosides) while preserving

RNase H degradation of the target mRNA. Mipomersen is also referred to as a 5-10-5 MOE gapmer where the term gap refers to the ten 2′ deoxyribonucleosides that are necessary to support enzymatic cleavage of the cognate mRNA.

The sequence of mipomersen is shown (note that all cytosine residues are methylated at the 5-position).

Figure 4-1 Design of Chimeric 2′-MOE Phosphorothioate Oligonucleotides (MOE-Gapmer)

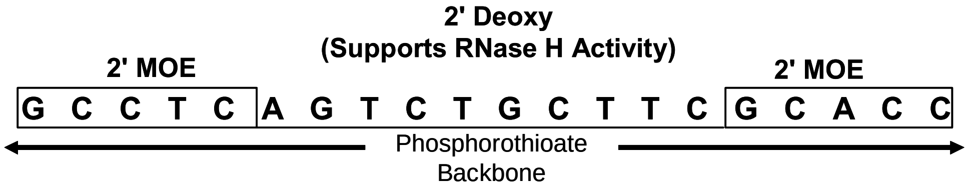

4.3 Summary of Benefits and Risks

Please refer to the Investigator's Brochure for additional information on clinical and nonclinical studies, and any known potential risks and benefits to humans.

5 STUDY OBJECTIVES

The objective of this study is to compare the safety and efficacy of 26 weekly, subcutaneous (SC) injections of mipomersen (200 mg) against placebo in treating severely hypercholesterolemic patients who are on a maximally tolerated lipid-lowering regimen and who are not on apheresis.

6 INVESTIGATIONAL PLAN

6.1 Study Design

This is a prospective, randomized, double-blind, placebo-controlled, parallel-group, multicenter Phase 3 study to investigate the safety and efficacy of mipomersen to treat severe hypercholesterolemia. Approximately 51 severely hypercholesterolemic adult patients will be randomized who are on a maximally tolerated lipid-lowering regimen and not on apheresis, with low density lipoprotein cholesterol (LDL-C) of at least 200 mg/dL (5.1 mmol/L) for patients with coronary heart disease (CHD), or at least 300 mg/dL (7.8 mmol/L), for patients without CHD. This represents a patient population in which LDL-C apheresis is indicated in the United States of America (USA). As many as 24 additional patients may be randomized to the study, for a total of 75 patients, to provide up to 90% power for efficacy and to provide further safety information.

Patients will be randomized in a 2:1 ratio to receive mipomersen or placebo, respectively. Mipomersen or a matching volume of placebo will be administered weekly via SC injection for 26 doses.

Following the treatment period and Week 28 evaluation, patients may elect to continue in an open-label extension study, if one is available, pending approval by the Ethics Committee and the appropriate regulatory authority. Patients who choose not to participate or are ineligible for the open-label extension study will continue post-treatment follow-up in the current protocol.

The study will be conducted in compliance with International Conference on Harmonisation (ICH), Good Clinical Practice (GCP) guidelines as well as in accordance with all national, state and local laws of the appropriate regulatory authorities. The duration of the study, including completing patient enrollment, is expected to be approximately 18 months. Each patient will participate in a ≤ 4-week screening period and a 26-week treatment period. All patients, including those who discontinue prematurely and have received ≥ 1 dose of study drug, should be followed for safety for 24 weeks after their last dose of study drug as part of the post-treatment evaluation period (Figure 6-1).

An independent data monitoring committee (DMC) will provide an ongoing, expert, independent review of safety data to assure that the risks to study patients are minimized. This ongoing review will include pre-specified review of safety data during the conduct of the study as defined by the DMC Charter. Based on these data, the DMC may recommend changes in study conduct to Genzyme. As part of the conduct of this study, Genzyme will periodically evaluate the nature, frequency and severity of AEs that have been identified as potential risks associated with the use of mipomersen or other new observations.

The end of study is defined as the last patient's last visit.

Figure 6-1 Study Design

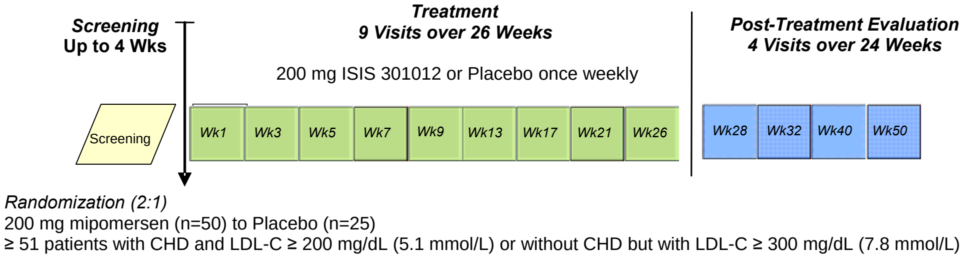

6.2 Endpoints

6.2.1 Efficacy Endpoints

6.2.1.1 Primary Efficacy Endpoint

The primary efficacy endpoint is the percent change in LDL-C from Baseline to Week 28/Early Termination (ET represents the observation closest to 2 weeks after last dose among patients who terminate study medication dosing early).

6.2.1.2 Secondary Efficacy Endpoints

Secondary efficacy endpoints include the percent change in apoB, and lipoprotein a (Lp[a]) from Baseline to Week 28/ET and the percent of patients with LDL-C below LDL-C entry criteria at Week 28/ET (200 mg/dL [5.1 mmol/L] for patients with coronary heart disease [CHD], or 300 mg/dL [7.8 mmol/L] for patients without CHD). The incremental effects of mipomersen on total cholesterol, TG, non-high density lipoprotein cholesterol (non-HDL-C), very low density lipoprotein cholesterol (VLDL-C), apo A-1, and lipoprotein subclasses may also be evaluated.

6.2.2 Safety Endpoints

Safety will be determined using the incidence of treatment-emergent adverse events (AEs), clinical laboratory evaluations, vital signs, electrocardiograms (ECGs), and physical examination findings. Adverse events will be categorized using the Medical Dictionary for Regulatory Activities (MedDRA). Plasma concentrations of mipomersen will be evaluated.

6.3 Discussion of Study Design Including Choice of Control Group

This is a randomized, double-blind, placebo-controlled, parallel-group study with 24 weeks of treatment and 6 months of safety follow-up. This treatment duration and follow-up has been chosen as it is roughly 5 times the terminal elimination half-life of mipomersen, so that

the efficacy endpoint can be evaluated when drug tissue levels are expected to be at > 90% of steady-state values and the safety assessments carried out when plasma trough levels are close to 0.

It is considered appropriate to treat the control group with placebo as these patients will also be receiving a maximally tolerated lipid-lowering regimen including statin (the gold standard of care), diet, and a medication from a second class of hyperlipidemic agents.

Based on nonclinical and clinical findings discussed in the Investigator's Brochure, close monitoring of safety laboratory parameters has been stipulated within the protocol particularly focusing on liver function tests (LFTs; also including imaging of the liver and spleen by magnetic resonance imaging [MRI]) or computed tomography (CT), and clotting parameters. The protocol also specifies under what conditions the implementation of clear stopping rules are to be employed to protect patients from unnecessary exposure to study medication. The LFT stopping rules are adapted from the draft guidance for industry, "Drug-Induced Liver Injury: Premarketing Clinical Evaluation," issued by the U.S. Department of Health and Human Services, Food and Drug Administration, October 2007.

Injection site reactions (ISRs) will be closely monitored. Inflammatory markers will be measured regularly.

Finally, the overall safety of the study will be monitored by an independent DMC.

7 PATIENT POPULATION AND SELECTION

Approximately 50 study centers will enroll up to 75 patients in this protocol.

7.1 Inclusion Criteria

Patients must fulfill all of the following criteria:

1. Age ≥ 18 years
2. Fasting LDL-C ≥ 200 mg/dL (5.1 mmol/L) and TG < 350 mg/dL (4.0 mmol/L) at Screening
3. Presence of at least 1 of the following criteria for coronary disease:
   a) Myocardial infarction (MI)
   b) Percutaneous coronary intervention (PCI) or coronary artery bypass graft (CABG)
   c) CAD documented by angiography or any other accepted imaging technique
   d) If 1 or more of criteria a) through c) are not met: a positive exercise test (≥ 1 mm ST-depression at maximal exercise or test terminated because of angina) or a perfusion defect, e.g., thallium or single photon emission computed tomography (SPECT)
   e) Or, if a) through d) are not met, LDL-C ≥ 300 mg/dL (7.8 mmol/L)
4. On a stable, maximally tolerated lipid-lowering regimen and expected to remain on it through Week 28 (must satisfy all criteria):
   a) A statin at a maximally tolerated dose per Investigator judgment, for at least 8 weeks prior to Screening
   b) A stable low-fat diet (e.g., NCEP-ATP III therapeutic lowering cholesterol [TLC] or equivalent) beginning at least 12 weeks prior to the first dose of study drug
   c) A medication from an additional class of hypolipidemic agents, per Investigator's judgment (e.g., bile acid sequestrants, niacin/nicotinic acid, fibrates) for at least 8 weeks prior to Screening
5. Body mass index (BMI) ≤ 40 kg/m^2 with weight stable (± 4 kg) for > 6 weeks prior to Screening per patient report.
6. Satisfy 1 of the following:
   a) Females: Non-pregnant and non-lactating; surgically sterile, postmenopausal, or patient or partner compliant with an acceptable contraceptive regimen for 4 weeks prior to Screening, during the treatment phase, and 24 weeks after the last study drug dose
   b) Males: Surgically sterile or patient or partner is utilizing an acceptable contraceptive method during the treatment phase and 24 weeks after the last study drug dose
7. Given informed consent

7.2 Exclusion Criteria

Patients meeting any of the following criteria will be excluded from the study:

1. Any of the following diagnoses, conditions, or prior treatments:
    a) MI, PCI, CABG, cerebrovascular accident (CVA), unstable angina or acute coronary syndrome within 24 weeks of Screening
    b) Presence of clinically significant arrhythmias, implantable pacemakers or automatic implantable cardioverter defibrillators (AICDs), or currently taking any medication for arrhythmias
    c) Type 1 diabetes mellitus
    d) New York Heart Association (NYHA) functional classification III or IV heart failure
    e) Hypertension, systolic blood pressure (BP) ≥ 160 mmHg, or diastolic BP ≥ 95 mmHg at Screening (despite antihypertensive medication/therapy)
    f) Active infection requiring systemic antimicrobial therapy
    g) Positive test for human immunodeficiency virus (HIV) or hepatitis B or C at Screening
    h) Any uncontrolled condition that may predispose the patient to secondary hyperlipidemia such as uncontrolled hypothyroidism.
    i) Malignancy within 5 years, except for basal or squamous cell carcinoma of the skin that has been adequately treated
    j) Clinically significant hepatic or renal disease or Gilbert's syndrome
    k) Apheresis within 3 months prior to Screening or expected to start apheresis during the treatment phase
2. The following laboratory values at Screening:
    a) Serum creatine phosphokinase (CPK) ≥ 3 x upper limit of normal (ULN)
    b) Alanine aminotransferase (ALT) levels > 1.5 x ULN
    c) Serum creatinine > 0.1 mg/dL (> 8.8 µmol/L) above ULN for women, or > 0.2 mg/dL (> 17.7 µmol/L) above ULN for men
    d) Proteinuria (> 1+ on dipstick, confirmed on retest, with further confirmation by quantitative total urine protein > 1.0 g/24 hr)
    e) Total Bilirubin > 1.0 x ULN
    f) Glycosylated hemoglobin A (HbA_{1C}) > 8.0%
3. Use of the following medications within 12 weeks of Screening:
    a) Medications that may affect lipids except those allowed per the protocol, including but not limited to Cholestin™ (also known as red yeast rice, or monascus purpureus extract)
    b) Anti-obesity medications

c) Chronic systemic corticosteroids or anabolic agents except for replacement therapy
d) Oral contraceptives

4. Use of the following medications unless a stable dose regimen was used for at least 12 weeks prior to Screening and the dose and regimen are expected to remain stable until Week 28:
   a) Oral anticoagulants (e.g., warfarin)
   b) Hormone replacement therapy
   c) Diabetes medications including but not limited to sulfonylureas, metformin and glitazones, with the exception of changes of ± 10 units of insulin.
   d) Antiviral therapy for herpes simplex virus (HSV)
5. Treatment with another investigational drug, biological agent, or device within 4 weeks of Screening or 5 half-lives of study agent, whichever is longer
6. Recent history of, or current, drug or alcohol abuse, or unwillingness to limit alcohol consumption for the entire duration of the study, including follow-up
7. Any disorders that would limit study participation or unwillingness to comply with study procedures, including follow-up, as specified by this protocol, or unwillingness to cooperate fully with the Investigator
8. Have any other medical conditions that, in the opinion of the Investigator, would make the patient unsuitable for enrollment, or could interfere with the patient participating in or completing the study

7.3 Patient Withdrawal

Patients have the right to withdraw from the study at any time and for any reason without prejudice to their ongoing or future medical care.

Any patient who withdraws consent to participate in the study will be removed from further treatment and/or study observation immediately upon the date of request.

Should a patient (or a legally acceptable representative) request or decide to withdraw from the study, all efforts will be made to complete and report the observations as thoroughly as possible up to the date of withdrawal, and an Early Termination visit should be performed. All information should be reported on the applicable electronic Case Report Forms (eCRFs).

Patients who request to stop study drug or have been withdrawn from study treatment at the request of the Investigator or Sponsor before completion of the protocol-specified treatment period, and who have received at least 1 dose of study drug and have not revoked their consent to participate in the study, will be strongly encouraged to continue the post-treatment evaluation period beginning with the Week 28 visit assessments. This visit should be

scheduled within 2 weeks of their last dose of study drug. For those patients who withdraw from the study prior to receiving any dose of study drug, no further follow up is necessary.

Patients who request to withdraw from the study during the post-treatment evaluation period should be encouraged to undergo clinical laboratory and safety evaluations including, but not limited to, hematology and chemistry panels, urinalysis, pregnancy test (for women of childbearing potential), vital signs collection and a physical examination prior to leaving the study (if it has been longer than 4 weeks since these procedures were last performed).

The Investigator must record the reason for withdrawal on the Study Termination eCRF.

If the patient withdraws due to an AE, the Investigator should arrange for the patient to have follow-up visits until the AE has resolved or stabilized.

Reasons for removal from study drug or observation may include:

- Withdrawal of consent
- Administrative decision by the Investigator or Sponsor
- Pregnancy (report on Pregnancy Notification Forms)
- Ineligibility
- Significant protocol deviation
- Patient non-compliance
- The patient experiences an AE that is considered intolerable by the patient or Investigator (includes disease progression; report on AE eCRF)

8 TREATMENTS

8.1 Treatments Administered

Patients will be randomized in a 2:1 ratio (i.e., mipomersen:placebo)

Mipomersen: 1-mL weekly injections of 200 mg of mipomersen will be administered for 26 weeks.

Placebo: 1-mL weekly injections of placebo (i.e., vehicle consisting of 9 mg of sodium chloride, 0.004 mg of riboflavin, filled to (QS) 1 mL with water for injection) for 26 weeks.

Injections will be administered as SC injections into the outer area of the upper arm, the medial thigh region, or the abdomen. The first 3 doses must be supervised by a health professional, including study center personnel or home health nurse. Thereafter, patients will be given the option to self-administer study drug injections unsupervised with the exception of doses on clinic visit days.

A specific administration schedule will be used in this study in order to gain some understanding about the patient's preference of injection site and the reason for the choice. For the first 3 injections, the injection site will be specified for all patients. The first injection will be in the outer area of the upper arm, the second injection will be in the medial thigh region, and the third injection will be in the abdomen. Based on the patient's experience from the first 3 doses, each will be allowed to choose the injection site for their remaining injections. The chosen site and the reason for the choice, (e.g., tolerability, convenience, physician recommendation, aesthetic, no reason, etc.) will be recorded on the eCRF. The patient may choose a different injection site at any time during the remainder of the treatment period. The new injection site and the reason for the change should be recorded on the eCRF. The patient's opinion on injection site location will also be collected at the conclusion of the treatment period.

The dose schedule may be adjusted in the event a patient is unable to come to the clinic due to vacations or holidays as follows:

- Patients can make up for a missed dose by receiving 2 doses of study drug in the week before or the week after the missed dose(s) provided that the 2 doses are given at least 3 days apart.
- Patients should be strongly discouraged from missing more than 2 scheduled doses of study drug in the first 13 weeks of dosing, more than 3 scheduled doses in the 26-week treatment period, and more than 1 of the last 4 scheduled doses of study drug.
- If the Investigator or the study coordinator is aware that a patient will be missing more than the recommended number of scheduled visits during the dosing period, the patient should be excluded from the study.

Dosing details must be recorded in the patient's source documents and the eCRF.

8.2 Investigational Product

Mipomersen is supplied as 200 mg/mL of mipomersen (ISIS 301012), with 1 mL of solution per vial.

Placebo is the vehicle, consisting of 9 mg of sodium chloride, 0.004 mg of riboflavin, QS to 1 mL with water for injection.

8.2.1 Packaging and Labeling

The study drug described in Section 8.2 is contained in 2-mL stoppered glass vials that will be provided to the study center by the Sponsor or designee. Mipomersen active pharmaceutical ingredient is manufactured by Isis Pharmaceuticals, Inc., Carlsbad, California, USA.

The Sponsor or designee will provide the Investigator with packaged study drug labeled in accordance with specific country regulatory requirements.

8.2.2 Study Drug Preparation

The study drug must be brought to room temperature prior to administration. Using aseptic technique, withdraw 1 mL from the vial of mipomersen (ISIS 301012) or placebo. Refer to the Dosing Instructions in the Investigational Product Handling Manual for additional details.

8.2.3 Drug Storage

The study drug must be stored securely at 2° to 8° Celsius and be protected from light in a limited access area.

8.3 Prior and Concomitant Medications

Reasonable efforts will be made to determine all medications and treatments (pharmacological and non-pharmacological) received by the patient. Prior medications or treatments are defined as any medication or therapy taken by the patient within 1 month prior to Screening. In addition, an attempt will be made to collect data on any lipid-lowering medications taken within 1 year prior to Screening. A medication or treatment is considered concomitant if it is taken at any time after the Screening visit up to and including the day of the final study evaluation. Data on medications will include: name, dose, route, regimen, start date, stop date, and indication. Data on treatments will include: treatment, start date, stop date, and indication. At each study visit, the patient will be asked about any additional medications or treatments or any changes in regimen or dosages since the last visit. Indications for any new medications or treatments during the study period will be recorded as an AE.

All concomitant lipid-lowering drugs are to remain constant from Screening through Week 28 of the study. Investigators may prescribe concomitant medications or treatments deemed necessary to provide adequate supportive care, with the exception of those listed in Section 7.2 as exclusions.

All concomitant medications taken during the study must be recorded in the source documentation and in the eCRF.

8.4 Method of Assigning Patients to Treatment

Using an Interactive Voice Response System (IVRS), patients will be randomized to mipomersen or placebo in a 2:1 ratio, prior to study drug administration. Patient enrollment will be conducted such that at least 51 patients are randomized with LDL-C of at least 200 mg/dL (5.1 mmol/L) for patients with CHD, or at least 300 mg/dL (7.8 mmol/L) for patients without CHD, representing a patient population in which LDL-C apheresis is indicated in the USA. Up to 24 more patients may be randomized to provide additional efficacy and safety information. The Sponsor's Quality Assurance (QA) & Compliance department or designee will hold a copy of the randomization schedule, generated by the Sponsor (or designee). All patients, Investigators, study staff, and the Sponsor will be blinded to the treatment assignment.

8.5 Dose Selection

The 200 mg/week dose was selected for this study because results from Phase 1 and Phase 2 clinical studies showed a satisfactory safety profile with a significant pharmacodynamic effect at this dose (see Investigator's Brochure). Due to the long terminal elimination half-life of mipomersen, the treatment duration is 26 weeks so that the efficacy endpoint can be evaluated when drug tissue levels are expected to be at > 90% of steady-state values.

The safety and tolerability of the proposed dosing regimen is also supported by nonclinical chronic toxicology studies (see Investigator's Brochure).

8.6 Blinding and Randomization

Patients, monitors, study center personnel, and the Sponsor will remain blinded to the patient's treatment assignment throughout the study. In addition, in order to ensure maintenance of the study blind, lipid data will be not be available to the patients, Investigators, study staff, or the Sponsor until the study has been unblinded, as knowledge of these data could unblind these individuals to the treatment assignment and could influence patient assessment.

8.6.1 Unblinding

In the event of an emergency where the identity of the study drug must be known by the Investigator to provide appropriate medical treatment, the Investigator will be allowed to unblind using the IVRS. However, prior to this, the Investigator should make every effort to discuss the situation with the Medical Monitor.

In the event that an unblinding occurs, the Investigator will notify the Sponsor or designee of the unblinding within 24 hours of its occurrence. The treatment assignment should only be unblinded in the case of a serious adverse event (SAE; see Section 9.2.2), and when knowledge of the treatment assignment will impact the clinical management of the patient. Every reasonable attempt should be made to complete the End-of-Study (study termination) evaluation procedures prior to unblinding as knowledge of the treatment assignment could influence patient assessment.

9 EFFICACY AND SAFETY VARIABLES

9.1 Efficacy and Safety Measurements Assessed and Study Flowchart

The study for an individual patient will consist of the following periods:

- Up to a 4-week screening period
- A 26-week treatment period during which study drug will be administered (consisting of a SC dose once a week), and
- A 24-week post-treatment evaluation period

Table 9-1 summarizes the schedule of study events at each visit for patients enrolled.

Table 9-1 Schedule of Study Events

Study Period	Screen -ing	Treatment									Post-Treatment Evaluation			
Study Week	≤ 4 weeks	Wk 1	Wk 3	Wk 5	Wk 7	Wk 9	Wk 13	Wk 17	Wk 21	Wk 26	Wk 28	Wk 32	Wk 40	Wk 50
Study Day	-28 to -1	1	15	29	43	57	85	113	141	176	190	218	274	344
Visit[h]	-1	Visit 1	Visit 2	Visit 3	Visit 4	Visit 5	Visit 6	Visit 7	Visit 8	Visit 9	PTx Visit 1	PTx Visit 2	PTx Visit 3	PTx Visit 4
Visit Window		± 2 days									± 7 days			
Written Informed Consent	X													
Medical History	X													
Demographics and Baseline Characteristics	X	X												
Inclusion / Exclusion Criteria	X													
Physical Examination[a]	X			X		X	X				X			X
Abdominal & Hip Circumference	X										X			
Liver & Spleen MRI or CT		X[b]												
Vital Signs (+ body weight and height at Screening)	X	X	X	X	X	X	X	X	X	X	X	X	X	X
Urinalysis[c]	X[g]	X[d]	X	X	X	X	X[d]	X	X	X	X[d]	X	X	X
Pregnancy Test[e]	X	X		X		X	X	X	X	X		X	X	X
ISR Evaluation		X	X	X	X	X	X	X	X	X	X			
Weekly Study Drug[f] Administration		X	X	X	X	X	X	X	X	X				

Table 9-1 Schedule of Study Events (Continued)

Study Period	Screen-ing	Treatment									Post-Treatment Evaluation			
Study Week	≤ 4 weeks	Wk 1	Wk 3	Wk 5	Wk 7	Wk 9	Wk 13	Wk 17	Wk 21	Wk 26	Wk 28	Wk 32	Wk 40	Wk 50
Study Day	-28 to -1	1	15	29	43	57	85	113	141	176	190	218	274	344
Visit[h]	-1	Visit 1	Visit 2	Visit 3	Visit 4	Visit 5	Visit 6	Visit 7	Visit 8	Visit 9	PTx Visit 1	PTx Visit 2	PTx Visit 3	PTx Visit 4
Visit Window		± 2 days									± 7 days			
12-Lead ECG	X						X				X			X
AE Assessment	X	X	X	X	X	X	X	X	X	X	X	X	X	X
Concomitant Medications/Therapies	X	X	X	X	X	X	X	X	X	X	X	X	X	X

[a] Full physical examination at Screening must include height measurement. Physical examinations, including general appearance, skin, head, ears, eyes, nose, throat (HEENT), lymph nodes, heart, lungs, abdomen, extremities/joints, neurological, and mental status will be performed at Screening and during the treatment period. Additionally, the patients will be monitored for lymphadenopathy and splenomegaly.

[b] Baseline magnetic resonance imaging (MRI) or computed tomography (CT) should be done prior to administering the first dose of the study drug. Follow-up MRI or CT will be obtained if clinically indicated (i.e., for alanine aminotransferase [ALT] elevations ≥ 3 x upper limit of normal [ULN]) during the course of the study. Please refer to the Site Reference Binder for MRI and CT procedures and settings.

[c] Sample collected prior to study drug administration during treatment period

[d] Urinalysis on these days includes total protein, microalbumin, creatinine, urine protein to creatinine ratio (P/C ratio), and β2-microglobulin

[e] For females of childbearing potential only.

[f] The first 3 doses must be supervised by a health professional, including study center personnel or home health nurse. Thereafter, patients will be given the option to self-administer study drug injections unsupervised.

[g] At Screening, if urine dipstick is positive (> 1+) for microalbuminuria, then a 24-hour sample will be obtained to assess protein excretion. If total protein > 1g/24 hr then the patient will be excluded.

ISR = injection site reaction
ECG = electrocardiogram
AE=adverse event

Table 9-1 Schedule of Study Events (Continued)

Study Period	Screen-ing	Treatment									Post-Treatment Evaluation			
Study Week	≤ 4 weeks	Wk 1	Wk 3	Wk 5	Wk 7	Wk 9	Wk 13	Wk 17	Wk 21	Wk 26	Wk 28	Wk 32	Wk 40	Wk 50
Study Day	-28 to -1	1	15	29	43	57	85	113	141	176	190	218	274	344
Visit[h]	-1	Visit 1	Visit 2	Visit 3	Visit 4	Visit 5	Visit 6	Visit |7	Visit 8	Visit 9	PTx Visit 1	PTx Visit 2	PTx Visit 3	PTx Visit 4
Visit Window		± 2 days									± 7 days			
Chemistry Panel (+ CPK)	X	X	X	X	X	X	X	X	X	X	X	X	X	X
Full Serum Lipid Panel	X	X		X		X	X	X	X	X	X	X	X	X
HbA_{1C}[k]	X						X				X			X
Hematology (Complete Blood Count + Diff.)	X	X	X	X	X	X	X	X	X	X	X	X	X	X
Coagulation (aPTT, PT)		X					X				X			
Hepatitis B & C, HIV	X													
Thyroid function (T_4, TSH)	X													
Trough PK - ISIS 301012[j]		X	X	X	X	X	X	X	X	X	X	X	X	X
ISIS 301012 Antibodies[i]		X									X			X
IgG		X					X				X			
Cardiovascular Risk Markers (hsCRP and LDL subclass concentrations)	X	X					X			X	X			
Complement (C3)		X					X				X			

Table 9-1 Schedule of Study Events (Continued)

Study Period	Screen-ing	Treatment									Post-Treatment Evaluation			
Study Week	≤ 4 weeks	Wk 1	Wk 3	Wk 5	Wk 7	Wk 9	Wk 13	Wk 17	Wk 21	Wk 26	Wk 28	Wk 32	Wk 40	Wk 50
Study Day	-28 to -1	1	15	29	43	57	85	113	141	176	190	218	274	344
Visit[h]	-1	Visit 1	Visit 2	Visit 3	Visit 4	Visit 5	Visit 6	Visit 7	Visit 8	Visit 9	PTx Visit 1	PTx Visit 2	PTx Visit 3	PTx Visit 4
Visit Window		± 2 days[c]									± 7 days			
Erythrocyte Sedimentation Rate (ESR)		X					X				X			
Inflammatory Markers[l]		X					X				X			

[h] All visits are conducted after an overnight fast for > 10 hours (only water permitted). Note: all labs must be performed prior to injection of study drug.
[i] Sample collected prior to study drug administration during treatment period
[j] The outpatient visits at Week 13 and Week 26 must be scheduled on a dosing day, and must occur 7 days after the previous dose. Blood samples for pharmacokinetic (PK) trough levels MUST be drawn prior to mipomersen administration at all outpatient visits during the treatment period.
[k] At Screening for all patients and diabetic patients only thereafter.
[l] In addition to the pre-specified inflammatory markers (i.e., immunoglobulin G [IgG], white blood cell [WBC] differentials, complement [C3], erythrocyte sedimentation rate [ESR], and high-sensitivity C-reactive protein [hsCRP]), a serum sample specifically designated for the measurement of inflammatory parameters will be collected pretreatment, during treatment (Week 13) and at the end of treatment (Week 28) and appropriately stored for subsequent analysis, as needed. This will allow for the measurement of other inflammatory markers should significant changes in the primary markers be noted or for further characterization of inflammation based on emerging markers.

Note: The laboratory test, hsCRP, is a marker of inflammation and, as such, may be elevated due to any condition that causes general inflammation including but not limited to recent or ongoing illness, viral, bacterial or fungal infection, tissue injury, trauma, bone fractures or exacerbation of chronic inflammatory conditions. Since this test is sensitive to these other conditions, samples for hsCRP evaluations should not be drawn from patients with acute inflammatory conditions or with chronic inflammatory conditions that have been acutely exacerbated until such conditions are clinically stable. In any event, tests should not be postponed for more than 4 weeks. Reasons for such postponing tests should be documented.

Note: All items on this page of the study schedule require blood draws (collected prior to study drug administration during the treatment period). CPK=Creatine phosphokinase, aPTT=activated partial thromboplastin time, PT=prothrombin time, T_4=thyroxine, TSH=thyroid stimulating hormone, HbA_{1c}=Glycosylated hemoglobin A, HIV=human immunodeficiency virus, LDL=low density lipoprotein.

9.1.1 Screening Assessments

The initial Screening visit will take place within a maximum of 4 weeks before the first dose of study drug is administered (on Day 1). Once written informed consent is obtained, a patient's eligibility for entry into the study will be assessed according to the inclusion and exclusion criteria.

9.1.1.1 Medical History, Physical Examinations, 12-Lead Electrocardiogram

Patients will give a full medical history, and undergo a full physical examination, including anthropometric and vital signs measurements and monitoring for lymphadenopathy and splenomegaly (Refer to Section 9.1.2.6). A 12-lead ECG tracing will be obtained.

9.1.1.2 Clinical Laboratory Evaluations and Therapeutic Lifestyle Changes

Blood and urine samples will be collected for clinical laboratory evaluations according to the schedule in Table 9-1 (see Table 9-2 for a complete list of analytes). The patient will also be counseled on the TLC approach (Appendix 14.1); this will include advice on diet that should be maintained throughout the treatment period. Lipoprotein testing information is in Appendix 14.2.

Table 9-2 List of Screening Clinical Laboratory Evaluations

<table>
<tr><th colspan="4">Screening Assessments</th></tr>
<tr><th>Clinical Chemistry</th><th>Hematology</th><th>Urinalysis</th><th>Other</th></tr>
<tr>
<td rowspan="3">Sodium
Potassium
Chloride
Bicarbonate
Total protein
Albumin
Total globulin
Calcium
Phosphorus
Glucose
Blood urea nitrogen (BUN)
Creatinine
Uric Acid
LDH
Total, Direct, and Indirect Bilirubin
Alkaline phosphatase
AST (SGOT)
ALT (SGPT)
CPK</td>
<td rowspan="3">Red blood cells
Hemoglobin
Hematocrit
MCV
Platelets
White blood cells (WBC)
WBC Differential (% and absolute)
• Neutrophils
• Eosinophils
• Basophils
• Lymphocytes
• Monocytes</td>
<td>Specific gravity
pH
Protein
Glucose
Ketones
Bilirubin
Blood
Red blood cells
WBC
Epithelial cells
Bacteria
Casts
Crystals</td>
<td><u>Thyroid Function</u>
T_4
TSH

<u>Hepatitis</u>
Hepatitis B surface antigen
Hepatitis C antibody

<u>HIV</u>
HIV antibody

<u>Pregnancy</u>
hCG

<u>Cardiovascular Risk Markers</u>
hsCRP
LDL-subclass concentrations</td>
</tr>
<tr><th>Full Lipid Panel</th><th>Metabolic</th></tr>
<tr><td>ApoB
ApoA-1
Total cholesterol
LDL-C
HDL-C
VLDL-C
TG
Lp(a)</td><td>HbA_{1c}</td></tr>
</table>

BUN=blood urea nitrogen, LDH=low density lipoprotein, AST (SGOT)=aspartate aminotransferase, ALT (SGPT)=alanine aminotransferase, CPK=creatine phosphokinase, MCV=mean corpuscular volume, WBC=white blood cells, apo=apoliprotein, LDL-C=low density lipoprotein cholesterol, HDL-C=high density lipoprotein cholesterol, VLDL-C= very high density lipoprotein cholesterol, TG=triglycerides, Lp(a)=lipoprotein (a), T_4=thyroxine, TSH=thyroid-stimulating hormone, HIV=human immunodeficiency virus, hCG=human chorionic gonadotropin, hsCRP=high-sensitivity C-reactive protein, HbA_{1c}=glycosylated hemoglobin A.

9.1.2 Treatment Period Assessments

The study for an individual patient will consist of the following periods:

- Up to a 4-week screening period
- A 26-week treatment period during which study drug will be administered (consisting of a SC dose once a week), and

- A 24-week post-treatment evaluation period

There is a visit window of ± 2 days through Week 26. In the event that a visit does not occur on the scheduled date, all subsequent visits should be calculated based on the time elapsed since Day 1, rather than from the date of the last visit. The outpatient visits at Week 13 and Week 26 must be scheduled on a dosing day, and must occur 7 days after the previous dose.

9.1.2.1 Magnetic Resonance Imaging and Computed Tomography

Baseline MRI or CT of the liver and spleen will be collected for all patients prior to administering the first dose of study drug. A CT scan may be performed in lieu of both the baseline and follow-up MRI for patients in whom an MRI is contraindicated. The images will be stored for future analysis if required. Follow-up MRI (or CT) will be obtained if clinically indicated (i.e., for ALT elevations ≥ 3 x ULN) during the course of the study. Please refer to the Site Reference Binder for MRI (or CT) procedures and settings.

9.1.2.2 Clinical Laboratory Evaluations

The following clinical laboratory tests will be assessed by a central laboratory at the time points specified in Table 9-1.

A blood sample for baseline full serum lipid panel, hematology with differential, chemistry, cardiovascular risk markers (high-sensitivity C-reactive protein [hsCRP] and LDL subclass concentrations), immunoglobulin G (IgG), ISIS 301012 antibodies, erythrocyte sedimentation rate (ESR), complement (C3), and coagulation parameters (activated partial thromboplastin time [aPTT] and prothrombin time [PT]) will be drawn prior to study drug administration according to the schedule in Table 9-1. In addition to the pre-specified inflammatory markers (i.e., IgG, white blood cell (WBC) differentials, C3, ESR, and hsCRP), a serum sample specifically designated for the measurement of inflammatory parameters will be collected pretreatment, during treatment (Week 13) and at the end of treatment (Week 28) and appropriately stored for subsequent analysis, as needed. This will allow for the measurement of other inflammatory markers should significant changes in the primary markers be noted or for further characterization of inflammation based on emerging markers. Urinalysis will be performed at each visit during the treatment period. Pregnancy tests for women of childbearing potential will be performed monthly during study participation. Table 9-3 details clinical laboratory evaluations for the treatment period; Table 9-1 lists the visits at which samples will be drawn.

A central laboratory will provide collection supplies, arrange collection, and perform analysis of clinical laboratory evaluations indicated in Table 9-2 and Table 9-3. Using the central laboratory for unscheduled clinical laboratory evaluations and redraws is

recommended. Procedures for the handling and shipment of all central laboratory samples will be included in the laboratory manual. Specimens will be appropriately processed by the central laboratory facility and laboratory reports will be made available to the Investigator in a timely manner to ensure appropriate clinical review.

The Investigator is responsible for reviewing and signing all laboratory reports. The clinical significance of each value outside of reference range will be assessed and documented as either not clinically significant (NCS) or clinically significant (CS). All CS values require a comment and the out-of-range value or the overlying diagnosis (or value if the diagnosis is unknown) must be captured as an AE.

Clinical significance is defined as any finding that results in an alteration in medical care (for definition of clinical significance, please refer to Section 9.2.3.5). The Investigator will continue to monitor the patient until the parameter returns to its baseline status or until agreement is reached between the Investigator and Sponsor.

Clinical laboratory reports will serve as source documents.

Table 9-3 List of Treatment Period Clinical Laboratory Evaluations

Clinical Safety Assessments (Minimum Requirements)		Efficacy Assessments	Other Assessments
**Clinical Chemistry**	**Urinalysis**	**Full Lipid Panel**	**Metabolic**
Sodium<br>Potassium<br>Chloride<br>Bicarbonate<br>Total protein<br>Albumin<br>Total globulin<br>Calcium<br>Phosphorus<br>Glucose<br>BUN<br>Creatinine<br>Uric Acid<br>LDH<br>Total, Direct, and Indirect Bilirubin<br>Alkaline phosphatase<br>AST (SGOT)<br>ALT (SGPT)<br>CPK	Specific gravity<br>pH<br>Protein<br>Glucose<br>Ketones<br>Bilirubin<br>Blood<br>Red blood cells<br>WBC<br>Epithelial cells<br>Bacteria<br>Casts<br>Crystals<br>Creatinine*<br>Total Protein*<br>Protein to creatinine (P/C) Ratio*<br>Microalbumin*<br>β2-microglobulin*	ApoB<br>ApoA-1<br>Total cholesterol<br>LDL-C<br>HDL-C<br>VLDL-C<br>TG<br>Lp(a)	HbA$_{1c}$<br>**Inflammatory Markers†**<br>To be determined
**Coagulation**	**Hematology**	**Cardiovascular Risk Markers**	**Immune Function**
aPTT<br>PT<br>INR<br>**Complement**<br>C3	Red blood cells<br>Hemoglobin<br>Hematocrit<br>MCV<br>Platelets<br>White blood cells<br>WBC Differential (% and absolute)<br>• Neutrophils<br>• Eosinophils<br>• Basophils<br>• Lymphocytes<br>• Monocytes	hsCRP<br>LDL subclass concentrations	IgG<br>ISIS 301012 antibodies<br>ESR<br>**Pharmacokinetic**<br>Trough ISIS 301012 level<br>**Pregnancy**<br>hCG[a]

*At select outpatient visits (Weeks 1, 13, and 28)
[a] For women of childbearing potential; see Table 9-1 for schedule of visits at which a pregnancy test is performed.
†In addition to the pre-specified inflammatory markers (i.e., IgG, WBC Differential, C3, ESR, and hsCRP), a serum sample specifically designated for the measurement of inflammatory parameters will be collected pretreatment, during treatment (Week 13) and at the end of treatment (Week 28) and appropriately stored for subsequent analysis, as needed.

9.1.2.3 Demographics, Vital Signs, Body Weight, and Anthropometric Measurements

Demographic data, height, and weight will be recorded at the Screening visit.

Vital signs (BP, heart rate, respiratory rate, and temperature) will be measured at the time points specified in Table 9-1. Semi-supine systolic and diastolic BP should always be measured on the same arm (preferentially on the left arm) with the patient being in a semi-supine position for at least 5 minutes. Any clinically significant abnormal findings will be recorded on the AE page of the eCRF.

If a CS deterioration is noted, the changes will be documented as AEs on the eCRF. Clinical significance is defined as any finding that results in an alteration in medical care. The Investigator will continue to monitor the patient until the parameter returns to its baseline status or until agreement is reached between the Investigator and Sponsor.

Height and body weight should be measured without shoes. Body weight should be measured with the patient fully dressed in everyday clothes, but overcoats should be removed before weighing.

Waist circumference should be measured with a stretchless tape and patient in a standing position after normal expiration, midway between the caudal part of the lateral costal arch and the iliac crest. Hip circumference should be measured at the symphysis trochanter level. Calculations to be performed are BMI and waist-to-hip ratio (WHR).

9.1.2.4 Pharmacokinetic Trough Levels

At each outpatient visit to the study center, including Day 1, a blood sample will be drawn prior to study drug administration for plasma PK trough levels. Blood samples for PK trough levels MUST be drawn prior to study drug administration at all outpatient visits during the treatment period.

9.1.2.5 Electrocardiogram

The ECG will be recorded after the patient has been resting in a supine position for at least 5 minutes. Six limb leads, as specified by Einthoven (I, II and III) and Goldberger (aVR, aVL and aVF) and 6 precordial leads (V1-V6) according to Wilson will be used. Printouts of the recordings will be done with 25 mm/s time resolution at an amplification of 10 mm/mV. Electrocardiograms will be performed at Screening, and at Weeks 13, 28, and 50.

The ECG tracing should contain at least 4 QRS complexes (in order to obtain 3 RR intervals). Each ECG tracing should include the following information: identification of each lead, study number, study day number, patient number, paper speed, voltage

calibration, and date and time of recording. After identification of each lead, the Investigator should evaluate, sign, and date the printout. The Investigator should state whether the ECG is normal or abnormal; if abnormal, whether the ECG tracing is clinically significant.

9.1.2.6 Physical Examination

A complete physical examination will be conducted at the time points specified in Table 9-1. The examination will include an assessment of the patient general appearance; skin; head, ears, eyes, nose throat (HEENT); examinations of lymph nodes; heart; lungs; abdomen; extremities/joints; and neurological and mental status. Additionally, the patients will be specifically monitored for lymphadenopathy and splenomegaly and any findings will be recorded. Whenever possible, the same physician should perform the examination at each study visit.

If a clinically significant deterioration is noted, the change will be documented as an AE on the eCRF. Clinical significance is defined as any finding that results in an alteration in medical care. The Investigator will continue to monitor the patient until the parameter returns to its baseline status or until agreement is reached between the Investigator and Sponsor.

9.1.2.7 Concomitant Medications

Concomitant medications are to be assessed at all visits per Table 9-1. Reasonable efforts will be made to determine all treatments (pharmacological and non-pharmacological) received by the patient. A therapy is considered concomitant if it is taken at any time after the Screening visit up to and including the day of the final study evaluation. Data on pharmacological and non-pharmacological treatments will be captured on separate eCRF pages, but both to include: name, dose, route, regimen, start date, stop date, and indication. At each study visit, the patient will be asked about any additional treatments or any changes in regimen or dosages since the last visit. Indications for any new medications or therapies during the study period will be recorded as an AE.

9.1.2.8 Injection Site Reaction Assessment

At each outpatient visit during the treatment period, an ISR assessment will be made for the current and previous injection sites. ISR assessments are recorded starting after the first injection is given.

9.1.3 Post-Treatment Evaluation Period Assessments

There is a visit window of ± 7 days throughout the post-treatment evaluation period. During this period, MRI or CT (if clinically indicated), clinical laboratory evaluations, vital signs, body weight, anthropometric measurements, plasma PK trough levels, ECG, and a full

physical examination will be performed according to the schedule in Table 9-1, and as described in Section 9.1.2. Adverse events and changes in concomitant medication use will continue to be reported at each visit.

9.2 Adverse Events

9.2.1 Definition of Adverse Events

An AE is defined as any undesirable physical, psychological or behavioral effect experienced by a patient during their participation in an investigational study, in conjunction with the use of the drug, whether or not product-related. This includes any untoward signs or symptoms experienced by the patient from the time of signing the informed consent until completion of the study.

Adverse events may include, but are not limited to:

- Subjective or objective symptoms spontaneously offered by the patient and/or observed by the Investigator or medical staff
- Laboratory abnormalities of clinical significance

Disease signs, and symptoms and/or laboratory abnormalities already existing prior to the use of the product are not considered AEs after treatment unless they recur after the patient has recovered from the pre-existing condition or, in the opinion of the Investigator, they represent a clinically significant exacerbation in intensity or frequency.

9.2.2 Definition of Serious Adverse Events

A SAE is any AE that results in any of the following outcomes:

- Death
- Life-threatening experience
- Required or prolonged inpatient hospitalization
- Persistent or significant disability/incapacity
- Congenital Anomaly
- Important medical events that may jeopardize the patient or subject and may require medical or surgical intervention to prevent one of the outcomes listed above.

Death: The patient died following an AE.

Life-threatening experience: Any AE that places the patient, in the view of the reporter, at immediate risk of death from the AE as it occurred, i.e., does not include an AE that had it occurred in a more severe form, might have caused death.

Requires inpatient hospitalization, or prolongation of existing hospitalization: The AE resulted in an initial inpatient hospitalization or prolonged an existing hospitalization of the patient. For this protocol, hospitalization is defined as an admission to a medical facility of greater than 24 hours.

Persistent or significant disability/incapacity: An AE that results in a substantial disruption of a person's ability to conduct normal life functions.

Congenital anomaly: The exposure of the patient to the drug during pregnancy that is judged to have resulted in the congenital anomaly/birth defect.

Important medical events: AEs that may not result in death, be life-threatening, or require hospitalization may be considered SAEs when, based upon appropriate medical judgment, they may jeopardize the patient and may require medical or surgical intervention to prevent one of the outcomes listed above.

Planned hospital admissions or surgical procedures for an illness or disease that existed before the administration of the study treatment or before the patient was enrolled in the study, will not be captured as SAEs unless they occur at a time other than the planned date.

Serious adverse events will be followed until resolution or until such time agreed upon by the Sponsor or designee and the Investigator. Adverse events and concomitant medications will be collected from the time of informed consent through study completion (Week 50 or Early Termination).

9.2.3 Evaluation of Adverse Events and Serious Adverse Events

9.2.3.1 Severity of Adverse Events

The Investigator will assess the severity of the (S)AE using the following categories: Mild, Moderate, and Severe. This assessment is subjective, and the Investigator should use medical judgment to compare the reported (S)AE to similar events observed in clinical practice.

Guidelines for severity assessment are listed below:

Mild: Symptom(s) barely noticeable to the patient or do(es) not make the patient uncomfortable. The (S)AE does not influence performance or functioning. Prescription drugs are not ordinarily needed for relief of symptom(s).

Moderate: Symptom(s) of a sufficient severity to make the patient uncomfortable. Performance of daily activities is influenced. Treatment of symptom(s) may be needed.

Severe: Symptom(s) of a sufficient severity to cause the patient severe discomfort. Severity may cause cessation of treatment with the study drug. Treatment for symptom(s) may be given.

Severity is not equivalent to seriousness.

9.2.3.2 Action Taken/Medication or Therapy Given

The Investigator will be required to provide any action taken with regards to an (S)AE. The action taken is determined as follows:

No change: No change in product dosing was implemented. This includes cases in which the product was discontinued prior to the occurrence of the (S)AE.

Temporarily Discontinued: The drug was discontinued temporarily and will probably be restarted at a later time point. The Investigator will provide a stop and restart date.

Permanently Discontinued: The drug was discontinued permanently. The Investigator will provide a stop date.

9.2.3.3 Outcome of Adverse Events

The Investigator will be required to provide information regarding the patient outcome of an (S)AE to the study drug.

The patient outcome is determined as follows:

Recovered: The (S)AE has resolved and the patient does not have any residual symptoms.

Recovered With Sequelae: The (S)AE has resolved, however the patient has some residual symptoms. The Investigator will provide information to specify the sequelae.

Not Yet Recovered: The patient has not yet recovered from the (S)AE. These cases will be followed-up until the report is classified in one of the other categories or follow-up is no longer required as determined by the Investigator. The Investigator must follow patients with (S)AEs until their condition resolves or stabilizes. Certain (S)AEs may not resolve. Serious adverse events will be followed until resolution or until such time agreed upon by the Sponsor or designee and the Investigator.

Ongoing at the time of death: The (S)AE was ongoing at the time of death of the patient and was not directly related to the patient's death.

Fatal: The patient died from the (S)AE. The patient's date of death is the date of the (S)AE cessation.

9.2.3.4 Relationship to Study Treatment

The Investigator will assess the relationship between the (S)AE and the study treatment according to the following definitions:

Unrelated: There was no relationship of the (S)AE to the use of the study drug. This may include, but is not limited, to the (S)AE being an expected outcome of a previously existing or concurrent disease, concomitant medication or procedure the patient experienced during their treatment period. For reporting purposes, Unrelated will be considered Not Product Related.

Remote/Unlikely: (Serious) Adverse experiences which are judged probably not related to the drug. For reporting purposes, Remote/Unlikely will be considered Not Product Related.

Possible: There was no clear relationship of the (S)AE to the use of the study drug; however, one cannot definitively conclude that there was no relationship. For reporting purposes, Possible will be considered Product Related.

Probable: While a clear relationship to the drug cannot be established, the experience is associated with an expected (S)AE or there is no other medical condition or intervention which would explain the occurrence of such an experience. For reporting purposes, Probable will be considered Product Related.

Definite: The relationship of the use of the drug to the experience is considered definitively established. For reporting purposes, Definite will be considered Product Related.

9.2.3.5 Clinical Laboratory Tests

The Investigator will assess all laboratory results outside the normal range as CS or NCS. Clinical significance is defined as any variation that has medical relevance resulting in an alteration in medical care. Whenever possible, the underlying diagnosis should be listed in lieu of the abnormal laboratory values. Laboratory abnormalities deemed NCS by the Investigator should not be reported as (S)AEs. Similarly, laboratory abnormalities reported as (S)AEs by the Investigator should not be deemed NCS on the laboratory sheet.

9.2.3.6 Dosing Errors

Dosing details should be captured on the Dosing eCRF. In addition, if the patient takes a dose of study drug that significantly exceeds protocol specifications, the “Dose/dosing rate exceeded protocol specifications” box should also be checked. If the patient is symptomatic, the “Associated with AE(s)? - Yes” box should be checked. If the event does not meet serious criteria, the symptom(s) should be documented on the non-serious AE eCRF and the corresponding AE numbers listed on the Dosing eCRF; if the event meets serious criteria, an SAE form should be completed and the event reported as a SAE instead. The site should

also document the SAE on the AE eCRF and enter the corresponding AE numbers on the Dosing eCRF. Do not capture the event on the non-serious AE eCRF or SAE form if the patient is not symptomatic.

9.2.4 Adverse Experience and Serious Adverse Experience Reporting

9.2.4.1 Adverse Experience Reporting

Adverse events, including SAEs, will be reported from the time the patient signs the informed consent form until study completion. All AEs, including SAEs, experienced by the patient will be noted on the eCRF. A full description including the nature, date, time of onset and resolution, determination of seriousness, intensity, corrective treatment, outcome, and relationship to study drug will be recorded. Changes in vital signs, laboratory results and other safety assessments from Baseline will be recorded if they are deemed clinically significant. When possible, a diagnosis should be recorded as an AE, rather than the symptoms related to that diagnosis. A medical or surgical procedure is not an AE; rather the condition leading to the procedure should be recorded as the AE. All SAEs experienced by the patient will be recorded on a SAE report form and reported to Genzyme.

The Investigator must follow patients with AEs until their condition resolves or stabilizes. Certain AEs may not resolve. Serious adverse events will be followed until resolution or until such time agreed upon by Genzyme or its designee and the Investigator. Adverse events and concomitant medications will be collected from the time of informed consent through study completion (Week 50 or Early Termination).

9.2.4.2 Serious Adverse Event Reporting

9.2.4.2.1 Reporting to Genzyme

All SAEs must be reported by to the Genzyme Pharmacovigilance Department within 24 hours of the Investigator's first knowledge of the event (Table 9-4). SAE communication is directed to:

Table 9-4 Contact List for Serious Adverse Event Reporting

United States and Rest of World (excluding Europe)	Europe
**Genzyme US Pharmacovigilance**<br><br>FAX: 1 617 761 8506<br><br>Email:<br>pharmacovigilancesafety@genzyme.com<br><br>Phone:<br>Night (6 pm EST- 8 am EST), Weekends and Holidays<br>1 800 745 4447 (US toll free number)<br>1 617 768 9000 (outside US)	**Genzyme Europe BV Pharmacovigilance**<br><br>FAX: +31 (0) 35 694 87 56<br><br>Email:<br>EUPharmacovigilance@genzyme.com<br><br>Phone:<br>7days/24hours<br>+31 (0) 35 699 12 99

For all SAEs, a SAE Report Form that includes a detailed written description, copies of relevant anonymized patient records, and other documents will be sent to Genzyme Pharmacovigilance within 24 hours. Follow-up SAE reports will be forwarded as soon as the information is available.

Additionally, the Institutional Review Board (IRB)/Independent Ethics Committee (IEC) must also be notified in writing of any SAEs according to the applicable regulations. Genzyme will report SAEs to appropriate regulatory agencies, as required.

After the study is completed, if the Investigator or study staff becomes aware of a related SAE that occurs within 30 days of study completion, this event and any known details must be reported to Genzyme Pharmacovigilance.

9.2.5 Pregnancy Reporting

If a patient becomes pregnant at anytime during the conduct of this study, she must not receive any additional study drug and must be discontinued from the study.

The patient must be followed until the outcome of the pregnancy is known (i.e., delivery, elective termination, or spontaneous abortion). If the pregnancy results in birth of a child, additional follow-up information may be requested.

In case of paternal exposure anytime after receiving study treatment, the patient may continue the study. However, the patient’s pregnant partner must be followed until the outcome of the pregnancy is known (i.e., delivery, elective termination, or spontaneous

abortion). If the pregnancy results in the birth of a child, additional follow-up information may be requested.

The Investigator must notify Genzyme Pharmacovigilance within 24 hours of first learning of the occurrence of pregnancy, using the appropriate Pregnancy Notification Form(s) (Appendix 14.3, Forms A and/or B), providing as much information as possible. For reporting initial notification of a female study patient please provide Pregnancy Notification Form A. If study patient is male, please complete and provide both Pregnancy Notification Forms A and B. The Investigator must notify Genzyme about reported complications within 24 hours using the same procedure. Outcome of pregnancy, once known by the Investigator, must also be reported to Genzyme within 24 hours using the Pregnancy Outcome Form C and faxing it to Genzyme. For reporting additional information about the pregnancy use Pregnancy Notification Form(s) A and/or B and indicate “follow-up” on the form.

Communications regarding pregnancies should be directed to the contacts listed in Table 9-4.

Please note that pregnancy in and of itself is neither an AE nor a SAE. Pregnancy should not be entered into the eCRF as an AE unless the Investigator suspects an interaction between the study treatment and contraceptive method used. For female patients, pregnancy will be documented as the reason for study discontinuation.

9.2.6 Stopping Rules and Safety Monitoring

In addition to the standard monitoring of clinical safety parameters, the following guidelines are provided for the monitoring of selected parameters chosen based on preclinical and clinical observations (see Section 8, “Guidance for the Investigator,” in the Investigator’s Brochure).

9.2.6.1 Confirmation Guidance

At any time during the study (treatment or post-treatment periods), initial clinical laboratory results meeting the stopping rules or safety monitoring criteria presented below must be confirmed by performing measurements (ideally in the same laboratory that performed the initial measurement) on new specimens. All new specimen collections should take place as soon as possible (ideally within 3 days of the initial collection) and, in any event, no longer than 5 days from the collection that yielded the initial observation. If the initial laboratory result is observed during the treatment period, the results from the retest must be available prior to the next scheduled dose of study drug (mipomersen or placebo).

If a new specimen is not, or cannot be, collected ≤ 7 days of the specimen collection that produced the initial laboratory observation and/or the result from the test of the new

specimen is not available prior to the scheduled administration of the next dose, the initial laboratory result is presumed confirmed.

9.2.6.2 Stopping Rule Guidance

If any of the stopping criteria described below are met and are confirmed, the patient will be permanently discontinued from further treatment with study drug (mipomersen or placebo), evaluated fully as outlined below and in consultation with the Medical Monitor, and will be entered into the post-treatment evaluation portion of the study. In general, patients who do not meet the stopping rules based upon retest may continue dosing. However, the Investigator and the Medical Monitor should confer as to whether additional close monitoring of the patient is appropriate.

9.2.6.3 Liver Chemistry Rules

The following rules are adapted from the draft guidance for industry, "Drug-Induced Liver Injury: Premarketing Clinical Evaluation," issued by the U.S. Department of Health and Human Services, Food and Drug Administration, October 2007.

9.2.6.3.1 Stopping Rules for Liver Chemistry Elevations

In the event of confirmed or presumed confirmed laboratory results meeting the following criteria, and the event is without an alternative explanation (e.g., concomitant therapy with anticoagulants), dosing of a patient with study drug (mipomersen or placebo) will be stopped permanently:

- ALT or aspartate aminotransferase (AST) ≥ 8 x ULN
- ALT or AST ≥ 5 x ULN at 2 consecutive weekly measurements (not less than 7 days) at least 1 of which is confirmed by retest. (Weekly measurement of liver chemistry tests will be instituted following the first ALT or AST ≥ 5 x ULN finding. Treatment with study drug [mipomersen or placebo] will continue weekly until the second consecutive weekly ALT or AST measurement is confirmed or presumed confirmed to be ≥ 5 x ULN).
- ALT or AST ≥ 3 x ULN and total bilirubin > 1.5 x ULN or international normalized ratio (INR) > 1.5

In the event of confirmed or presumed confirmed laboratory results for either of the following criteria, continued dosing of a patient with study drug (mipomersen or placebo) will be discussed with the Medical Monitor:

- ALT or AST ≥ 3 x ULN with the appearance or worsening of symptoms felt by the Investigator to be potentially related to hepatic inflammation, such as fatigue, nausea, vomiting, right upper quadrant pain or tenderness, fever, rash or eosinophilia

9.2.6.3.2 Safety Monitoring Rules for Liver Chemistry Tests

In the event of an ALT or AST measurement that is ≥ 3 x ULN at any time during the study (treatment or post-treatment period), the initial measurement(s) should be confirmed as described above. Similarly, confirmatory measurements should also be performed if ALT or AST levels increase to ≥ 5 x ULN and 8 x ULN (following the initial meeting of each of those criteria).

Frequency of Repeat Measurements: Patients with confirmed or presumed confirmed ALT or AST levels ≥ 3 x ULN should have their liver chemistry tests (ALT, AST, alkaline phosphatase, and total bilirubin at a minimum) retested at least once weekly until levels stabilize or begin to recover. The performance of additional laboratory tests should be discussed with the Medical Monitor. Thereafter, liver chemistry tests for these patients should be performed at least every 2 weeks until ALT and AST are both < 3 x ULN. Subsequently, liver chemistry tests for these patients should be performed at least once a month until their ALT and AST levels become ≤ 1.2 x ULN (or ≤ 1.2 x Screening value for patients who enter the study with ALT and/or AST > ULN).

Further Investigation into Liver Chemistry Elevations: For patients with confirmed or presumed confirmed ALT or AST levels ≥ 3 x ULN, the following evaluations should be performed:

- A detailed history of symptoms and prior and concurrent diseases.
- A history for concomitant drug use (including nonprescription medications, herbal and dietary supplement preparations), alcohol use, recreational drug use, and special diets.
- A history for exposure to environmental chemical agents and travel.
- Serology for viral hepatitis (hepatitis A virus [HAV] immunoglobulin M [IgM], hepatitis B surface antigen [HBsAg], hepatitis C virus [HCV] antibody, cytomegalovirus (CMV) IgM, and Epstein-Barr Virus [EBV] antibody panel)
- Serum albumin, PT or INR, and partial thromboplastin time (PTT)
- Serology for autoimmune hepatitis (e.g., antinuclear antibody [ANA]).
- Liver MRI (chemical shift imaging) or CT

Additional liver evaluations, including gastroenterology/hepatology consults, may be performed at the discretion of the Investigator, in consultation with the Medical Monitor. Repetition of the above evaluations should be considered if a patient's ALT and/or AST levels reach ≥ 5 x ULN.

9.2.6.4 Safety Monitoring Rules for Renal Function Test Results

In the event of confirmed or presumed confirmed laboratory results for either of the following criteria, continued dosing of a patient with study drug (mipomersen or placebo) will be discussed with the Medical Monitor:

- Serum creatinine increase ≥ 0.2 or 0.3 mg/dL (≥ 17.7 or 26.5 µmol/L) above Baseline, for women and men, respectively
- Proteinuria, dipstick ≥ 2 + (confirmed by dipstick re-measurement and then further confirmed by a quantitative total urine protein measurement of > 1.0 g/24 hr)

The follow-up schedule for any events meeting either of these criteria will be determined by the Investigator in consultation with the Medical Monitor.

9.2.6.5 Safety Monitoring Rule for Platelet Count Results

In the event of a confirmed or presumed confirmed platelet count less than 75,000/mm^3, continued dosing of a patient with study drug (mipomersen or placebo) will be discussed with the Medical Monitor. The follow-up schedule for any events meeting this criterion will be determined by the Investigator in consultation with the Medical Monitor.

9.2.6.6 Safety Monitoring Rules for Coagulation Parameters

In the event that a patient has a confirmed or presumed confirmed finding for either of the following 2 criteria and the event is without an alternative explanation (e.g., concomitant therapy with anticoagulants), the Investigator should discuss the follow-up of the event with the Medical Monitor.

- aPTT > 75 seconds
- PT > 20 seconds or INR > 1.5

9.2.6.7 Safety Monitoring Rule for Constitutional Symptoms

Patients will be instructed to report any signs or symptoms of fever or flu-like symptoms that may arise within the first 24 hours after an injection is given and the Investigator should closely evaluate all potential causes of the fever, including concomitant illness.

9.2.6.8 Restriction on the Lifestyle of Patients

Patients will be counseled to follow the TLC approach (see Appendix 14.1), or similar approach depending on local guidelines, throughout the treatment period.

Patients will be required to fast overnight for > 10 hours (only water permitted) before a blood sample is taken for a full serum lipid panel on all visit days.

Patients should be willing to limit alcohol consumption for the entire duration of the study: male patients to a maximum of 2 drinks (20 g) per day, and 8 drinks (80 g) per week; female patients to a maximum of 1 drink (10 g) per day, and 4 drinks (40 g) per week.

Male patients and female patients of childbearing potential must continue to use appropriate contraception with their partners, or refrain from sexual activity, from the time of Screening and throughout study participation until 24 weeks after the last dose of mipomersen. Acceptable methods of contraception are condoms with contraceptive foam, oral contraceptives that have been prospectively discussed with the Medical Monitor, implantable or injectable contraceptives, contraceptive patch, intrauterine device, diaphragm with spermicidal gel, or sexual partner who is surgically sterilized or postmenopausal.

9.2.7 Summary of Risks

Please refer to the Investigator's Brochure for information regarding the risks and benefits of mipomersen.

10 DATA COLLECTION, QUALITY ASSURANCE, AND MANAGEMENT

10.1 Recording of Data

Original documents, data, and records being: hospital records, clinical and office charts, laboratory notes/reports, memoranda, patients' evaluation checklists, pharmacy dispensing records, recorded data from automated instruments, copies of transcriptions certified after verification as being accurate copies, source document worksheets (i.e., for study visits and IVRS), and X-rays are considered source documents. Medical histories and narrative statements relating to the patient's progress (i.e., source documents) will be maintained during the trial and for a period of 15 years after completion of the study. The Investigator must provide direct access to the source documents to the Sponsor or designee.

All data captured for this study are to be recorded in the patient's notes first and then entered in the eCRF.

Clinical data will be entered using electronic data capture (EDC) technology from a reputable vendor. All data captured electronically will be provided on a CD-ROM at the end of the study in PDF format.

All required data will be recorded in the eCRF. All missing data will be explained. Any changes made to the data after initial entry will be captured in an electronic audit trail.

10.2 Data Quality Assurance

The eCRFs will be reviewed manually at the study site for completeness by a Clinical Monitor from the Sponsor or designee, and returned to the Sponsor or designee, for data

management and analysis. If necessary, the study site will be contacted for corrections and/or clarifications. All data will be entered into a study database for analysis and reporting. Any data captured electronically will be electronically transferred to the database. Upon completion of data entry, the database will receive a QA check to ensure acceptable accuracy and completeness.

10.3 Data Management

The Sponsor or designee will be responsible for:

- Database creation and validation
- eCRF review and data validation

Prior to finalizing and locking the database, all decisions concerning the inclusion or exclusion of data from the analysis for each patient will be determined by appropriate clinical and statistical personnel. Any and all exclusions related to either safety or efficacy will be documented in patient listings.

11 STATISTICAL METHODS AND PLANNED ANALYSES

The Sponsor or designee will be responsible for the production of the following items:

1. Data listings and summary tables
2. Statistical analysis
3. Combined clinical and statistical study report

All eCRF data, as well as any outcomes derived from the data, will be summarized in detailed data listings. Patient data listings will be presented for all patients enrolled into the study.

Descriptive summary statistics including n, mean, median, standard deviation, interquartile range (25th percentile, 75th percentile), and range (minimum, maximum) for continuous variables, and counts and percentages for categorical variables will be used to summarize most data. Where appropriate, p-values will be reported. All statistical tests will be conducted using 2-sided tests with 5% Type I error rates unless otherwise stated.

Prior to locking the database and unblinding the study, all decisions concerning exclusion of patients from analysis sets will be made by appropriate clinical and statistical personnel. The Safety Set will include all randomized patients who received at least 1 injection. The Full Analysis Set, which represents the practically-feasible intent-to-treat (ITT) population as delineated in ICH Guideline E9, will include the subset of the Safety Set with at least 1 post-Baseline LDL-C measure. The Per-Protocol Set will include the subset of the Full Analysis Set with no significant protocol deviations that would be expected to effect efficacy assessments. All efficacy parameters will be assessed on the Per-Protocol Set and Full Analysis Set (primary analysis population). All safety assessments will be performed on the Safety Set.

11.1 Power and Determination of Sample Size

Based upon prior clinical trial experience with mipomersen, it is estimated that the standard deviation of the percent change in LDL-C is approximately 22%. With 45 randomized patients (15 in the control group and 30 patients in the mipomersen-treated group), there would be 80% power to detect a 20 percentage point difference between the 2 groups. Enrollment will be conducted such that at least 51 patients are randomized to allow for potential exclusions from an analysis set. Should enrollment continue such that as many as 75 patients are randomized, this will yield as much as 90% power to detect a 20 percentage point difference between the 2 groups.

11.2 Demographics and Baseline Characteristics

Demographic and baseline characteristics will be summarized using descriptive statistics by treatment group.

11.3 Patient Accountability

Patient randomization will be summarized by study site and treatment group. The patient disposition will be summarized.

All patients enrolled will be included in a summary of patient accountability for this study. The frequency and percentages of patients enrolled, presenting at each visit, discontinuing before study completion (including reason for discontinuation), and completing the study will be presented.

11.4 Study Treatment Usage and Compliance

Treatment duration and amount of study drug received will be summarized by treatment group.

11.5 Efficacy

11.5.1 Primary Efficacy Endpoint

The primary efficacy endpoint is the percent change in LDL-C from Baseline to Week 28/ET (ET represents the observation closest to 2 weeks after last dose among patients who early terminate study medication dosing).

11.5.2 Secondary Efficacy Endpoints

Secondary efficacy endpoints include the percent change in apoB and Lp(a) from Baseline to Week 28/ET and the percent of patients with LDL-C below LDL-C entry criteria at Week 28/ET (200 mg/dL [5.1 mmol/L] for patients with coronary heart disease [CHD], or 300 mg/dL [7.8 mmol/L] for patients without CHD). The incremental effects of mipomersen on total cholesterol, TG, non-HDL-C, VLDL-C, apo A-1, and lipoprotein subclasses may also be evaluated.

11.5.3 Efficacy Analysis

Percent change from Baseline to Week 28/ET in LDL-C, apo B, total cholesterol, HDL-C, non-HDL-C, TG, and Lp(a) will be compared between treatment groups using a 2-sample t-test (if data departs substantially from normality; a Wilcoxon rank sum test will be used). The percent of patients with LDL-C below LDL-C entry criteria at Week 28/ET will be compared between treatment groups using Fisher's exact test. The analysis will take place after all patients have completed Week 28 and this database has been locked.

The Baseline is the value on Day 1, prior to the first dose of study drug. For patients without a Day 1 value (e.g., missing or inadequate sample), the last observation prior to the first dose of study drug will be used as Baseline.

11.6 Safety

11.6.1 Safety Endpoints

Safety will be determined using the incidence of treatment-emergent AEs, clinical laboratory evaluations, vital signs, ECGs, and physical examination findings. AEs will be categorized using MedDRA. Plasma concentrations of mipomersen will be evaluated.

11.6.2 Safety Analysis

The safety analyses will be performed on the Safety Set defined as all patients who receive at least 1 injection. Treatment duration and amount of study drug received will be summarized by treatment group, as will concomitant medications and treatments.

Treatment-emergent AEs will be summarized by body system, preferred term, severity, and relationship to the study procedures/treatments. If a patient has more than 1 occurrence of the same AE, he/she will be counted only once within that preferred term in the summary tables. The most severe occurrence of an AE, as well as the most extreme relationship of the AE to the study procedures or treatment, will be indicated in cases of multiple occurrences of the same AE. All AEs will be presented in a listing. Additionally listings of SAEs and AEs leading to discontinuation will be generated.

Clinical laboratory evaluations, vital signs, ECGs, and physical examination findings will be tabulated by treatment group. In addition, the number of patients who experience abnormalities in clinical laboratory evaluations will be summarized by treatment group.

Trough plasma concentrations of mipomersen (C_{min}) will be summarized over time by individual patient and by treatment group. Elimination half-life of mipomersen will be estimated for patients who enter the post-treatment follow-up period. Exploratory analyses will include but may not be limited to comparisons of trough plasma concentration of mipomersen over time within individual patients and within each treatment group.

11.7 Statistical Analysis Plan

A formal statistical analysis plan will be developed and finalized prior to database lock for the primary efficacy endpoint (e.g., all patients have completed Week 28).

12 SPECIAL REQUIREMENTS AND PROCEDURES

12.1 Institutional and Ethical Review

This protocol was designed and will be conducted, recorded, and reported in compliance with the principles of GCP guidelines as well as in accordance with all national, state and local laws of the appropriate regulatory authorities. These guidelines are stated in "Guidance for Good Clinical Practice," ICH Technical Requirements for Registration of Pharmaceuticals for Human Use.

A copy of the protocol, proposed informed consent form, other written patient information, and any proposed advertising material must be submitted to the IRB/IEC for written approval. A copy of the written approval of the protocol and informed consent form must be received by the Sponsor or designee before recruitment of patients into the study and shipment of study drug.

The Investigator must submit and, where necessary, obtain approval from the IRB/IEC for all subsequent protocol amendments and changes to the informed consent document. The Investigator should notify the IRB/IEC of deviations from the protocol or SAEs occurring at the study center and other AE reports received from the Sponsor or designee in accordance with local procedures.

The Investigator will be responsible for obtaining annual IRB/IEC approval/renewal throughout the duration of the study. Copies of the Investigator's reports and the IRB's/IEC's continuance of approval must be sent to the Sponsor or designee.

12.2 Changes to the Conduct of the Study or Protocol

No change in the study procedures shall be effected without the mutual agreement of the Investigator and Sponsor. All changes must be documented by signed protocol amendments. If changes to the design of the study are made, the amendment must be submitted to and approved by the IRB/IEC, signed by the Investigator, and returned to the Sponsor for submission to the appropriate regulatory authorities (e.g., US Food and Drug Administration [FDA], European national regulatory agencies, etc.).

12.3 Investigator's Responsibilities

12.3.1 Patient Informed Consent

The written informed consent document should be prepared in the language(s) of the potential patient population, based on an English version provided by the Sponsor or designee.

Before a patient's participation in the trial, the Investigator is responsible for obtaining written informed consent from the patient after adequate explanation of the aims, methods,

anticipated benefits, and potential hazards of the study and before any protocol-specific screening procedures or any study drugs are administered.

The acquisition of informed consent and the patient's agreement or refusal of his/her notification of the primary care physician should be documented in the patient's medical records, and the informed consent form should be signed and personally dated by the patient and by the person who conducted the informed consent discussion (not necessarily an Investigator). The original signed informed consent form should be retained in accordance with institutional policy, and a copy of the signed consent form should be provided to the patient or legally acceptable representative.

12.3.2 Electronic Case Report Forms

All data will be obtained using EDC. Refer to Section 10.1 for details regarding recording of data.

Copies of pertinent records in connection with the study, including patient charts, laboratory data, etc., will be made available to Genzyme on request with due precaution towards protecting the privacy of the patient.

12.3.3 Record Retention

Essential documents should be retained until at least 2 years after the last approval of a marketing application in an ICH region and until there are no pending or contemplated marketing applications in an ICH region or at least 2 years have elapsed since the formal discontinuation of clinical development of the investigational product. Patient files and other source data must be kept for the maximum period of time permitted by your institution, but not less than 15 years. Should Investigators be unable to continue maintenance of patient files for the full 15 years, Genzyme will assist in this regard.

These documents should be retained for a longer period, however, if required by the applicable regulatory requirements or by an agreement with the Sponsor. It is the responsibility of the Sponsor to inform the Investigator/institution as to when these documents no longer need to be retained.

Essential documents are those documents, which individually and collectively, permit evaluation of the conduct of a trial and the quality of the data produced. These documents serve to demonstrate the compliance of the Investigator, Sponsor or designee, and monitor with the standards of GCP and with all applicable regulatory requirements.

Any or all of the documents should be available for audit by the Sponsor's or designee's auditor and inspection by the regulatory authority(-ies).

12.3.4 Patient Discontinuation

If a patient decides to discontinue participation in the study, he or she should be contacted in order to obtain information about the reason(s) for discontinuation and collection of any potential AEs. Whenever possible the patient should return to the clinic for the Week 28 assessments. The Investigator will document the eCRF describing the reason for discontinuation.

12.3.5 Study or Site Termination

The Sponsor reserves the right to terminate the study, according to the study contract. For example, the study may be terminated and dosing stopped at any time if the Medical Monitor is of the opinion that proceeding with the study will compromise the safety of the patients in the study. The Investigator should notify the regulatory authority and IRB/IEC in writing of the trial's completion or early termination and provide a copy of the notification to the Sponsor or designee.

12.3.6 Monitoring

The Sponsor representative or designee and regulatory authority inspectors are responsible for contacting and visiting the Investigator for the purpose of inspecting the facilities and, upon request, inspecting the various records of the trial (for example, eCRFs and other pertinent data) provided that patient confidentiality is respected.

The Sponsor representative or designee is responsible for inspecting the eCRFs at regular intervals throughout the study to verify adherence to the protocol; completeness, accuracy, and consistency of the data; and adherence to local regulations on the conduct of clinical research. The monitor should have access to patient medical records and other study-related records needed to verify the entries on the eCRFs.

The Investigator agrees to cooperate with the monitor to ensure that any problems detected in the course of these monitoring visits, including delays in completing eCRFs, are resolved.

In accordance with ICH GCP and the Sponsor's audit plans, this study may be selected for audit by representatives from the Sponsor's Clinical and QA Department (or designees). Inspection of study center facilities (e.g., pharmacy, drug storage areas, laboratories) and review of study-related records will occur to evaluate the trial conduct and compliance with the protocol, ICH GCP, and applicable regulatory requirements.

12.3.7 Materials Control

12.3.7.1 Receipt of Clinical Supplies

The study staff is required to document the receipt of study drug supplies.

12.3.7.2 Disposition of Unused Clinical Supplies

The study staff is required to document the dispensing, accountability, and return of study drug supplies. The study center must return all used and unused study drug as per Sponsor or designee instructions. Note that unused study drug should be maintained in refrigerated storage (2° to 8°C) until returned. Refer to the Investigational Product Handling Manual for additional details.

12.3.8 Warnings, Precautions, Contraindications

For specific information concerning warnings, precautions, and contraindications, the Investigator is asked to refer to the appropriate section of the Investigator’s Brochure. Because of the possibility of AEs, a fully equipped emergency cart, or equivalent supplies and equipment, and personnel competent in recognizing and treating adverse reactions of all types should be immediately available.

Dose-dependent, transient, and reversible prolongations of aPTT have been observed with phosphorothioate oligonucleotides. Therefore, mipomersen should be used with caution in patients receiving warfarin, heparin or fractionated heparin products.

12.3.9 Clinical Study Report

If deemed appropriate by the Sponsor, the Coordinating Investigator shall be designated to sign the completed clinical study report at the end of this study.

The signatory Coordinating Investigator shall be identified by the Sponsor upon the completion of the study, based upon factors including, but not limited to, prior clinical research experience and publications, patient enrollment and level of involvement in the study.

12.3.10 Disclosure of Data

All information obtained during the conduct of this study will be regarded as confidential, and written permission from the Sponsor is required prior to disclosing any information relative to this study. Manuscripts prepared for publication will be in accordance with the policy established and presented to the Investigator previously by the Sponsor. Submission to the Sponsor for review and comment prior to submission to the publisher will be required. This requirement should not be construed as a means of restricting publication, but is intended solely to assure concurrence regarding data, evaluations, and conclusions and to provide an opportunity to share with the Investigator any new and/or unpublished information of which he/she may be unaware.

13 REFERENCES

Chen SH, Habib G, Yang CY, et al. Apolipoprotein B-48 is the product of a messenger RNA with organ-specific in-frame stop codon. *Science*. 1987;238(4825):363–366.

Cholesterol Treatment Trialists' Collaborators [CTTC]. Efficacy and safety of cholesterol-lowering treatment: prospective meta-analysis of data from 90,056 participants in 14 randomised trials of statins. *Lancet.* 2005;366:1267-1278.

Davidson MH, Maki KC, Pearson TA, et al. Results of the National Cholesterol Education Program (NCEP) Evaluation Project Utilizing Novel E-Technology (NEPTUNE) II survey and implications for treatment under the recent NCEP Writing Group Recommendations. *Am J Cardiol.* 2005;96(4):556-563.

Davis RA, Hui TY. 2000 George Lyman Duff Memorial Lecture. Atherosclerosis is a liver disease of the heart. *Arterioscler Thromb Vasc Biol*. 2001;21:887-898.

Gotto AM Jr. Management of dyslipidemia. *Am J Med*. 2002;112(8A):10S-18S.

Grundy SM, Cleeman JI, Merz CN, et al. Implications of recent clinical trials for the National Cholesterol Education Program Adult Treatment Panel III guidelines. *Circulation*. 2004;110:227-239.

Hulthe J, Fagerberg B. Circulating oxidized LDL is associated with subclinical atherosclerosis development and inflammatory cytokines (AIR study). *Arterioscler Thromb Vasc Biol*. 2002;22(7):1162-1167.

Knopp RH. Drug treatment of lipid disorders. *N Engl J Med*. 1999;341(7):498-511.

Third report of the National Cholesterol Education Program (NCEP) expert panel on detection, evaluation, and treatment of high blood cholesterol in adults (Adult Treatment Panel III) Final Report. *Circulation*. 2002;106(25):3143-3421.

Powell LM, Wallis SC, Pease RJ, Edwards YH, Knott TJ, Scott J. A novel form of tissue-specific RNA processing produces apolipoprotein-B48 in intestine. *Cell*. 1987;50(6):831–840.

Sankatsing RR, Fouchier SW, de Haan S, et al. Hepatic and cardiovascular consequences of familial hypobetalipoproteinemia. *Arterioscler Thromb Vasc Biol.* 2005;25:1979-1984.

Skalen K, Gustafsson M, Rydberg EK, et al. Subendothelial retention of atherogenic lipoproteins in early atherosclerosis. *Nature*. 2002;417(6890):750-754.

Sueta CA, Massing MW, Chowdhury M, Biggs DP, Simpson RJ Jr. Undertreatment of hyperlipidemia in patients with coronary artery disease and heart failure. *J Card Failure*. 2003;9(1):36-41.

14 APPENDICES

14.1 NCEP – ATP III Therapeutic Lifestyle Changes

- Reduced intakes of saturated fats (< 7% of total calories) and cholesterol (< 200 mg per day)
- Therapeutic options for enhancing LDL lowering such as plant stanols/sterols (2 g/day) and increased viscous (soluble) fiber (10 to 25 g/day)
- Weight reduction
- Increased physical activity

Table 14-1 Nutrient Composition of the TLC Diet

Nutrient	Recommended Intake
Saturated fat*	Less than 7% of total calories
Polyunsaturated fat	Up to 10% of total calories
Monounsaturated fat	Up to 20% of total calories
Carbohydrate†	50% to 60% of total calories
Fiber	20 to 30 g/day
Protein	Approximately 15% of total calories
Cholesterol	Less than 200 mg/day
Total calories (energy)‡	Balance energy intake and expenditure to maintain desirable body weight/prevent weight gain

*Trans fatty acids are another LDL-raising fat that should be kept at a low intake.
†Carbohydrate should be derived predominantly from foods rich in complex carbohydrates including grains, especially whole grains, fruits, and vegetables.
‡Daily energy expenditure should include at least moderate physical activity (contributing approximately 200 kcal per day)

14.2 Lipoprotein Testing

Lipoprotein testing will be performed in a clinical laboratory which holds a current certification of traceability to the national reference system for total cholesterol. In addition, the analytical systems and reagents used to measure total cholesterol and HDL-C must be certified by the Cholesterol Reference Method Laboratory Network (CRMLN) as having documented traceability to the national reference system for total cholesterol, and the designated comparison method for HDL-C, respectively.

Table 14-2 Lipoprotein Testing

Lipoprotein	Analysis Method
Total Cholesterol	Enzymatic colorimetry
LDL-C	Friedewald Calculation; ultracentrifugation for TG > 400
HDL-C	Dextran sulfate precipitation followed by enzymatic colorimetry
Triglycerides	Enzymatic colorimetry
Apo B-100	Rate Nephelometry
Apo A-1	Rate Nephelometry
Lp(a)	Isoform Independent assay standardized to Northwest Lipid Research Clinic

14.3 Pregnancy Notification Forms

Genzyme Coporation
500 Kendall Street
Cambridge, MA 02142

Genzyme Europe BV
Gooimeer 10
1411 DD Naarden
Netherlands

Pregnancy Notification Form A

Maternal Exposure

Report Type: ☐ Initial ☐ Follow-up

Source of fetal exposure: ☐ Maternal ☐ Paternal (please also complete Pregnancy Notification Form B)

Genzyme Use Only:
Case ID: ______
Report Type: ☐ Prospective ☐ Retrospective

I. Physician Information:

Name	Address	Phone	Fax	Office Contact
Primary Care Provider				
OB				

II. Maternal Information:
Is the patient participating in a Genzyme Clinical Trial?

☐ Yes Protocol Number: ______ Subject's Study Number/Initials: ____/____

☐ No Patient's Initials: ______

Date of Birth: ___/___/___ (dd / mmm / yyyy)
Age at the time of Conception: ______
Country of Birth: ______
Country of Residence: ______
Occupation: ______

Race: ☐ Not provided ☐ Caucasian ☐ Black ☐ Asian ☐ Native American ☐ Hispanic ☐ Other______

Highest Education Level: ______ Height: ______ ☐ cm ☐ inches Weight: ______ ☐ kg ☐ lb

III. Product Exposure:
Genzyme Product: ______

Date of First Exposure: ___/___/___ (dd / mmm / yyyy)
Date of Last Exposure: ___/___/___ (dd / mmm / yyyy)
Trimester Exposure: ☐ 1st ☐ 2nd ☐ 3rd
Gestational Age at Time of Drug Exposure in Weeks: ______
Dose, Route & Frequency: ______

IV. Patient's History:
Patient's Previous Obstetric History: Grava:____ Para:____ Spontaneous Abortions:_____ Other: ______

Were live births full-term? ☐ Yes ☐ No ______
If no, please describe.

Does the patient have a history of subfertility? ☐ No ☐ Yes______
If yes, please describe.

Patient's Relevant Medical and Social History (include information on known risk factors or conditions that may affect the outcome of the pregnancy e.g. alcohol, smoking or other substance consumption (include start and stop dates as appropriate), hypertension, eclampsia, diabetes, seizure disorder, thyroid disorder, asthma, allergic disease, heart disease, depression or other psychiatric disorders, sexually transmitted disorders, hepatitis, AIDs, environmental or occupational exposure that may pose a risk factor, or medical complications in a prior pregnancy): ______

Patient's Family History (includes hereditary diseases, congenital abnormalities, psychomotor retardation, and other familial disorders, both maternal and paternal): ______

Consanguinity between parents: ______
Specify degree.

Does the patient have a history of fetal or neonatal abnormalities? ☐ No ☐ Yes ______
If yes, please describe.

V. 1 June 2006

Please fax form to Genzyme Pharmacovigilance Department US at 617-761-8506 or EU at +31 35 694 8756.

Genzyme Coporation
500 Kendall Street
Cambridge, MA 02142

Genzyme Europe BV
Gooimeer 10
1411 DD Naarden
Netherlands

Pregnancy Notification Form A

Report Type: ☐ Initial ☐ Follow-up

Genzyme Use Only:
Case ID: ____________
Report Type: ☐ Prospective ☐ Retrospective

V. Present Pregnancy: Number of Fetuses: ________

Date Pregnancy was Confirmed: ____/____/____ (dd / mmm / yyyy) Method by which Pregnancy was Confirmed: ____________________

Date of Last Menstrual Period: ____/____/____ (dd / mmm / yyyy) Expected Delivery Date: ____/____/____ (dd / mmm / yyyy) Is Pregnancy in-vitro? ☐ Yes ☐ No

Was the patient treated for infertility? ☐ No ☐ Yes ____________________
If yes, please describe.

Prenatal Testing	Date	Result	Reason	Comment<br>Please use section VII if needed
Ultrasound				
Alpha-fetoprotein				
Amniocentesis				
Chorionic villus sampling				
Serology (rubella, toxoplasmosis)				
Other				

VI. Concomitant Medication: Record medication(s) within the 30 days prior to last menstrual period.

Medication	Dose	Route	Duration	Indication

VII. Additional Information: Significant medical complications thus far in the pregnancy. Specifiy week of gestation that the complication occurred, treatment and outcomes. Attach additional information if required.

VIII. Reporter Information: (If different than section I.)
Name, Addresss, Telephone Number and Fax:

IX. Person Completing This Form

Signature: ____________________ Print Name: ____________________ Date: ________

V. 1 June 2006 **Please fax form to Genzyme Pharmacovigilance Department US at 617-761-8506 or EU at +31 35 694 8756.**

Page 2 of 2

Genzyme Coporation
500 Kendall Street
Cambridge, MA 02142

Genzyme Europe BV
Gooimeer 10
1411 DD Naarden
Netherlands

Pregnancy Notification Form B

Paternal Exposure

Report Type: ☐ Initial ☐ Follow-up

Genzyme Use Only:
Case ID:________
Report Type: ☐ Prospective ☐ Retrospective

I. Paternal Information: Is the patient participating in a Genzyme Clinical Trial?

☐ Yes Protocol Number: __________ Subject's Study Number/Initials: _____/_____

☐ No Patient's intials: (last, first, middle)__________

Date of Birth: ____/____/_____ dd / mmm / yyyy

Country of Birth: __________

Country of Residence: __________

Occupation: __________

Race: ☐ Not provided ☐ Caucasian ☐ Black ☐ Asian ☐ Native American ☐ Hispanic ☐ Other__________

II. Product Exposure: Genzyme Product: __________

Date of First Exposure: ____/____/_____ dd / mmm / yyyy

Date of Last Exposure: ____/____/_____ dd / mmm / yyyy

Trimester Exposure: ☐ 1st ☐ 2nd ☐ 3rd

Gestational Age at Time of Drug Exposure in Weeks: __________

Dose, Route & Frequency: __________

III. Patient's History:

Patient's Relevant Medical and Social History (include alcohol, smoking or other substance consumption (include start and stop dates as appropriate), sexually transmitted disorders, hepatitis, AIDs, environmental or occupational exposure, etc.):

____

____

____

____

IV. Concomitant Medication: Record medication(s) within the 30 days prior to last menstrual period.

Medication	Dose	Route	Duration	Indication

V. Additional Information:

VI. Reporter Information:

Name, Addresss, Telephone Number and Fax:

VII. Person Completing This Form

Signature:__________ Print Name:__________ Date:__________

V. 1 June 2006

Please fax form to Genzyme Pharmacovigilance Department US at 617-761-8506 or EU at +31 35 694 8756.

Page 1 of 1

Genzyme Coporation
500 Kendall Street
Cambridge, MA 02142

Genzyme Europe BV
Gooimeer 10
1411 DD Naarden
Netherlands

Pregnancy Outcome Form C

Report Type: ☐ Initial ☐ Follow-up

Genzyme Use Only:
Case ID: ______
Report Type: ☐ Prospective ☐ Retrospective

I. Pregnancy Outcome: (If multiple birth, please photocopy and complete a form for each infant.)

Patient's intials: ______

☐ Live Birth: Date of Birth: ____/____/_____ (dd / mmm / yyyy) Weight: ______ ☐ kg ☐ lb Length: ______ ☐ cm ☐ inches Gestational age at delivery: ______

Type of live birth: ☐ Full term > 35 weeks gestation ☐ Preterm ≤ 35 weeks gestation ☐ Low birth weight <2500 grams

Method of Delivery: ☐ Vaginal ☐ Cesarean ______ *Please provide indication*

Sex: ☐ Male ☐ Female

Apgar score: 1 Minute ______ 5 Minute ______

Did the newborn experience any complications during or after birth? ☐ No ☐ Yes ______

Was there fetal dysmaturity? ☐ No ☐ Yes ______ *If yes, please describe.*

Result of the newborns physical exam: ☐ Within Normal Limits ☐ Abnormal ______ *Please describe major and minor congenital abnormalities* ☐ Unknown

☐ Non-Live Birth:

Date: ____/____/_____ (dd / mmm / yyyy) ☐ Elective termination ☐ Spontaneous Abortion (≤ 20 weeks) ☐ Stillborn (≥ 20 weeks)

Gestational age at termination: ______

Results of physical examination (gender, external anomalies) and pathology, if applicable: ______

II. Obstetric Information:

☐ Yes ☐ No Complications during pregnancy, specify: ______

☐ Yes ☐ No Complications during labor/delivery, specify: ______

Medications administered to the mother during delivery: ______

III. Additional Information: Describe any additional information regarding the outcome of this pregnancy:

IV. Pediatrician Contact:

Pediatrician Name	Address	Phone	Fax	Office Contact

V. Reporter Information: (If different than section IV.)
Name, Addresss, Telephone Number and Fax:

VI. Person Completing This Form

Signature: ______ Print Name: ______ Date: ______

V. 1 June 2006

Please fax form to Genzyme Pharmacovigilance Department US at 617-761-8506 or EU at +31 35 694 8756.

Page 1 of 1

PHASE 3 CLINICAL STUDY PROTOCOL

A Prospective Randomized, Double-Blind, Placebo-Controlled Study to Assess the Safety and Efficacy of Mipomersen in Patients with Severe Hypercholesterolemia on a Maximally Tolerated Lipid-Lowering Regimen and who are not on Apheresis

Protocol Number: MIPO3500108
EudraCT: 2008-006020-53
Final: 8 October 2008

**Sponsor:**	Genzyme Europe BV<br>Gooimeer 10<br>1411 DD Naarden<br>The Netherlands	Genzyme Corporation<br>500 Kendall Street<br>Cambridge, MA 02142<br>USA
**Medical Monitor/ Key Study Contacts:**	Ajay Duggal, MBChB, MRCP, DipPharmMed<br>Senior Medical Director<br>Genzyme Europe Research<br>310 Cambridge Science Park<br>Milton Road<br>Cambridge, UK<br>e-mail:<br>Ajay.Duggal@Genzyme.com<br>tel: +44 1223 394 050<br>fax: +44 1223 394191	Joanne M. Donovan, MD, PhD<br>Vice President, Clinical Research<br>Genzyme Corporation<br>USA<br>e-mail:<br>Joanne.DonovanMD@Genzyme.com
**Statistician:**	Scott Chasan-Taber, PhD<br>Senior Director, Biostatistics<br>Genzyme Corporation<br>USA	

This protocol was designed and will be conducted, recorded, and reported in compliance with the principles of Good Clinical Practice (GCP) guidelines as well as in accordance with all national, state and local laws of the appropriate regulatory authorities. These guidelines are stated in "Guidance for Good Clinical Practice," International Conference on Harmonisation of Technical Requirements for Registration of Pharmaceuticals for Human Use.

I have read and agree to abide by the requirements of this protocol.

Investigator printed name …………………………………………

Investigator Signature____Date

Signature Page for Sponsor's Representative

The following have reviewed and approved the protocol entitled, "MIPO3500108: A Prospective Randomized, Double-Blind, Placebo-Controlled Study to Assess the Safety and Efficacy of Mipomersen in Patients with Severe Hypercholesterolemia on a Maximally Tolerated Lipid-Lowering Regimen and who are not on Apheresis."

09 Oct 08

Joanne M. Donovan, MD, PhD
Vice President, Clinical Research
Genzyme Corporation
USA

Date

1 SYNOPSIS

NAME OF COMPANY	SUMMARY TABLE	FOR NATIONAL AUTHORITY USE ONLY:
Genzyme Corporation<br>500 Kendall Street<br>Cambridge, MA 02142<br>USA<br><br>Genzyme Europe BV<br>Gooimeer 10<br>1411 DD Naarden<br>The Netherlands<br><br>**NAME OF FINISHED PRODUCT**<br>mipomersen sodium injection/ISIS 301012 Injection, 200 mg/mL, 1.0 mL<br>**NAME OF ACTIVE INGREDIENT**<br>mipomersen sodium/ISIS 301012 Drug Substance	Referring to Part ……<br>of the Dossier:<br>Volume:<br>Page:<br>Reference:	

TITLE:
A Prospective Randomized, Double-Blind, Placebo-Controlled Study to Assess the Safety and Efficacy of Mipomersen in Patients with Severe Hypercholesterolemia on a Maximally Tolerated Lipid-Lowering Regimen and who are not on Apheresis

PROTOCOL NO.:
MIPO3500108

INVESTIGATOR STUDY CENTERS:
Approximately 50 study centers will enroll patients in this protocol.

OBJECTIVES:
To compare the safety and efficacy of 26 weekly, subcutaneous (SC) injections of mipomersen (200 mg) against placebo in treating severely hypercholesterolemic patients who are on a maximally tolerated lipid-lowering regimen and who are not on apheresis.

METHODOLOGY:
This is a prospective, randomized, double-blind, placebo-controlled, parallel-group, multicenter Phase 3 study to investigate the safety and efficacy of mipomersen to treat severe hypercholesterolemia. Approximately 75 severely hypercholesterolemic adult patients who are on a maximally tolerated lipid-lowering regimen with low density lipoprotein cholesterol (LDL-C) ≥ 190 mg/dL (4.9 mmol/L), and who are not on apheresis will be randomized into this study. Patient enrollment will be conducted such that at least 51 patients are randomized with LDL-C of at least 200 mg/dL (5.1 mmol/L) for patients with coronary heart disease (CHD), or at least 300 mg/dL (7.8 mmol/L) for patients without CHD, representing a patient population in which LDL-C apheresis is indicated in the United States of America (USA). Additionally, approximately 24 patients with LDL-C of at least 190 mg/dL (4.9 mmol/L) will be randomized to provide additional exposure among a slightly broader patient population.

Patients will be randomized in a 2:1 ratio to receive mipomersen or placebo, respectively. Mipomersen or a matching volume of placebo will be administered weekly via SC injection for 26 doses.

The study will be conducted in compliance with International Conference on Harmonisation (ICH), Good Clinical Practice (GCP) guidelines as well as in accordance with all national, state and local laws of the appropriate regulatory authorities. The duration of the study, including completing patient enrollment, is expected to be approximately 18 months. Each patient will participate in a ≤ 4-week screening period and a 26-week treatment period. All patients, including those who discontinue prematurely and have received ≥ 1 dose of study drug, should be followed for safety for 24 weeks after

NAME OF COMPANY<br>Genzyme Corporation<br>500 Kendall Street<br>Cambridge, MA 02142<br>USA<br><br>Genzyme Europe BV<br>Gooimeer 10<br>1411 DD Naarden<br>The Netherlands<br><br>**NAME OF FINISHED PRODUCT**<br>mipomersen sodium injection/ISIS 301012 Injection, 200 mg/mL, 1.0 mL<br>**NAME OF ACTIVE INGREDIENT**<br>mipomersen sodium/ISIS 301012 Drug Substance	**SUMMARY TABLE**<br>Referring to Part ……<br>of the Dossier:<br>Volume:<br>Page:<br>Reference:	**FOR NATIONAL AUTHORITY USE ONLY:**

their last dose of study drug as part of the post-treatment evaluation period. The end of study is defined as the last patient's last visit.

SAFETY MONITORING RULES:

An independent data monitoring committee (DMC) will provide an ongoing, expert, independent review of safety data to assure that the risks to study patients are minimized.

If any of the stopping criteria described in this protocol are met and are confirmed, the patient will be permanently discontinued from further treatment with study drug (mipomersen or placebo), evaluated fully in consultation with the Medical Monitor, and will be entered into the post-treatment evaluation portion of the study. In general, patients who do not meet the stopping rules based upon retest may continue dosing. However, the Investigator and the Medical Monitor should confer as to whether additional close monitoring of the patient is appropriate.

NUMBER OF PATIENTS:
Approximately 75 patients will be randomized into this study.

INCLUSION/EXCLUSION CRITERIA:
Inclusion Criteria:

Patients must fulfill all of the following criteria:

1. Age ≥ 18 years
2. Fasting LDL-C ≥ 190 mg/dL (4.9 mmol/L) and triglycerides (TG) < 350 mg/dL (4.0 mmol/L) at Screening
3. Presence of at least 1 of the following criteria for coronary disease:
   a) Myocardial infarction (MI)
   b) Percutaneous coronary intervention (PCI) or coronary artery bypass graft (CABG)
   c) Coronary artery disease (CAD) documented by angiography or any other accepted imaging technique
   d) If 1 or more of criteria a through c are not met: a positive exercise test (≥ 1 mm ST-depression at maximal exercise or test terminated because of angina) or a perfusion defect, e.g., thallium or single photon emission computed tomography (SPECT)
   e) Or, if a) through d) are not met, LDL-C > 300 mg/dL (7.8 mmol/L)

**NAME OF COMPANY**<br>Genzyme Corporation<br>500 Kendall Street<br>Cambridge, MA 02142<br>USA<br>Genzyme Europe BV<br>Gooimeer 10<br>1411 DD Naarden<br>The Netherlands<br>**NAME OF FINISHED PRODUCT**<br>mipomersen sodium injection/ISIS 301012 Injection, 200 mg/mL, 1.0 mL<br>**NAME OF ACTIVE INGREDIENT**<br>mipomersen sodium/ISIS 301012 Drug Substance	**SUMMARY TABLE**<br>Referring to Part ……<br>of the Dossier:<br>Volume:<br>Page:<br>Reference:	**FOR NATIONAL AUTHORITY USE ONLY:**

4. On a stable, maximally tolerated lipid-lowering regimen and expected to remain on it through Week 28 (must satisfy all criteria):
   a) A statin at a maximally tolerated dose per Investigator judgment, for at least 8 weeks prior to Screening.
   b) A stable low-fat diet (e.g., National Cholesterol Education Program [NCEP]-Adult Treatment Panel [ATP] III therapeutic lowering cholesterol [TLC] or equivalent) beginning at least 12 weeks prior to the first dose of study drug
   c) A medication from an additional class of hypolipidemic agents, per Investigator's judgment (e.g., bile acid sequestrants, niacin/nicotinic acid, fibrates) for at least 8 weeks prior to Screening.
5. Body mass index (BMI) ≤ 40 kg/m^2 with weight stable (± 4 kg) for > 6 weeks prior to Screening per patient report.
6. Satisfy 1 of the following:
   a) Females: Non-pregnant and non-lactating; surgically sterile, postmenopausal, or patient or partner compliant with an acceptable contraceptive regimen for 4 weeks prior to Screening, during the treatment phase, and 24 weeks after the last study drug dose
   b) Males: Surgically sterile or patient or partner is utilizing an acceptable contraceptive method during the treatment phase and 24 weeks after the last study drug dose
7. Given informed consent

Exclusion Criteria:

1. Any of the following diagnoses, conditions, or prior treatments:
   a) MI, PCI, CABG, cerebrovascular accident (CVA), unstable angina or acute coronary syndrome within 24 weeks of Screening
   b) Presence of clinically significant arrhythmias, implantable pacemakers or automatic implantable cardioverter defibrillators (AICDs), or currently taking any medication for arrhythmias
   c) Type 1 diabetes mellitus
   d) New York Heart Association (NYHA) functional classification III or IV heart failure
   e) Hypertension, systolic blood pressure (BP) ≥ 160 mmHg, or diastolic BP

NAME OF COMPANY<br>Genzyme Corporation<br>500 Kendall Street<br>Cambridge, MA 02142<br>USA<br><br>Genzyme Europe BV<br>Gooimeer 10<br>1411 DD Naarden<br>The Netherlands<br><br>**NAME OF FINISHED PRODUCT**<br>mipomersen sodium injection/ISIS 301012<br>Injection, 200 mg/mL, 1.0 mL<br>**NAME OF ACTIVE INGREDIENT**<br>mipomersen sodium/ISIS 301012 Drug Substance	**SUMMARY TABLE**<br>Referring to Part ……<br>of the Dossier:<br>Volume:<br>Page:<br>Reference:	**FOR NATIONAL AUTHORITY USE ONLY:**

≥ 95 mmHg at Screening (despite antihypertensive medication/therapy)

f) Active infection requiring systemic antimicrobial therapy

g) Positive test for human immunodeficiency virus (HIV) or hepatitis B or C at Screening

h) Any uncontrolled condition that may predispose the patient to secondary hyperlipidemia such as uncontrolled hypothyroidism.

i) Malignancy within 5 years, except for basal or squamous cell carcinoma of the skin that has been adequately treated

j) Clinically significant hepatic or renal disease or Gilbert's syndrome

k) Apheresis within 3 months prior to Screening or expected to start apheresis during the treatment phase

2. The following laboratory values at Screening:

a) Serum creatine phosphokinase (CPK) ≥ 3 x upper limit of normal (ULN)

b) Alanine aminotransferase (ALT) levels > 1.5 x ULN

c) Serum creatinine > 0.1 mg/dL (> 8.8 µmol/L) above ULN for women, or > 0.2 mg/dL (> 17.7 µmol/L) above ULN for men

d) Proteinuria (> 1+ on dipstick, confirmed on retest, with further confirmation by quantitative total urine protein > 1.0 g/24 hr)

e) Total Bilirubin > 1.0 x ULN

f) Glycosylated hemoglobin A (HbA_{1C}) > 8.0%

3. Use of the following medications within 12 weeks of Screening:

a) Medications that may affect lipids except those allowed per the protocol, including but not limited to Cholestin™ (also known as red yeast rice, or monascus purpureus extract)

b) Anti-obesity medications

c) Chronic systemic corticosteroids or anabolic agents except for replacement therapy

d) Oral contraceptives unless prospectively discussed with the Medical Monitor

e) Fibrates unless prospectively discussed with the Medical Monitor

4. Use of the following medications unless a stable dose regimen was used for at least 12 weeks prior to Screening and the dose and regimen are expected to remain stable

NAME OF COMPANY<br>Genzyme Corporation<br>500 Kendall Street<br>Cambridge, MA 02142<br>USA<br>Genzyme Europe BV<br>Gooimeer 10<br>1411 DD Naarden<br>The Netherlands<br>**NAME OF FINISHED PRODUCT**<br>mipomersen sodium injection/ISIS 301012 Injection, 200 mg/mL, 1.0 mL<br>**NAME OF ACTIVE INGREDIENT**<br>mipomersen sodium/ISIS 301012 Drug Substance	**SUMMARY TABLE**<br>Referring to Part ……<br>of the Dossier:<br>Volume:<br>Page:<br>Reference:	**FOR NATIONAL AUTHORITY USE ONLY:**

until Week 28:

a) Oral anticoagulants (e.g., warfarin)

b) Hormone replacement therapy

c) Diabetes medications including but not limited to sulfonylureas, metformin and glitazones, with the exception of changes of ± 10 units of insulin.

d) Antiviral therapy for herpes simplex virus (HSV)

5. Treatment with another investigational drug, biological agent, or device within 4 weeks of Screening or 5 half-lives of study agent, whichever is longer
6. Recent history of, or current, drug or alcohol abuse, or unwillingness to limit alcohol consumption for the entire duration of the study, including follow-up
7. Any disorders that would limit study participation or unwillingness to comply with study procedures, including follow-up, as specified by this protocol, or unwillingness to cooperate fully with the Investigator
8. Have any other medical conditions that, in the opinion of the Investigator, would make the patient unsuitable for enrollment, or could interfere with the patient participating in or completing the study

DOSE/ROUTE/REGIMEN:
Patients will be randomized in a 2:1 ratio (i.e., mipomersen: placebo)

Mipomersen: 1-mL injections of 200 mg of mipomersen (ISIS 301012) will be administered weekly for 26 weeks.

Injections will be administered as SC injections into the outer area of the upper arm, medial thigh region, or abdomen. The first 3 doses must be supervised by a health professional, including study center personnel or home health nurse. Thereafter, patients will be given the option to self-administer study drug injections unsupervised with the exception of doses on clinic visit days.

A specific administration schedule will be used in this study in order to gain some understanding about the patient's preference of injection site and the reason for the choice. For the first 3 injections, the injection site will be specified for all patients. The first injection will be in the outer area of the upper arm, the second injection will be in the medial thigh region, and the third injection will be in the abdomen. Based on the patient's experience from the first 3 doses, each will be allowed to choose the injection site for their remaining injections.

REFERENCE TREATMENT:

<table>
<tr><td>NAME OF COMPANY
Genzyme Corporation
500 Kendall Street
Cambridge, MA 02142
USA

Genzyme Europe BV
Gooimeer 10
1411 DD Naarden
The Netherlands

NAME OF FINISHED PRODUCT
mipomersen sodium injection/ISIS 301012
Injection, 200 mg/mL, 1.0 mL
NAME OF ACTIVE INGREDIENT
mipomersen sodium/ISIS 301012 Drug Substance</td><td>SUMMARY TABLE
Referring to Part ……
of the Dossier:
Volume:
Page:
Reference:</td><td>FOR NATIONAL AUTHORITY USE ONLY:</td></tr>
<tr><td colspan="3">Adult hypercholesterolemic patients receiving either mipomersen or placebo as part of the study protocol will be on a maximally tolerated lipid lowering regimen.

<u>Placebo</u>: 1-mL weekly injections of placebo (i.e., vehicle consisting of 9 mg of sodium chloride, 0.004 mg of riboflavin, filled to (QS) 1 mL with water for injection) for 26 weeks.

Injections will be administered using the same instructions as provided for mipomersen.</td></tr>
<tr><td colspan="3">CRITERIA FOR EVALUATION:
Safety:

Safety will be evaluated in terms of all treatment-emergent adverse events (AEs) and serious adverse events (SAEs), physical examination findings, vital signs parameters and clinical laboratory parameters. AEs will be categorized using a standardized coding dictionary (e.g., Medical Dictionary for Regulatory Activities [MedDRA]). Clinical laboratory tests including chemistry, hematology with differential, and urinalysis will be tabulated.

Efficacy:

Efficacy will be assessed by measuring LDL-C, apolipoprotein (apo) B, total cholesterol, TG, high density lipoprotein cholesterol (HDL-C), non-HDL-C, and lipoprotein (a) (Lp[a]). The incremental effects of mipomersen on very low density lipoprotein cholesterol (VLDL-C), apo A-1, and lipoprotein subclasses may also be explored.</td></tr>
<tr><td colspan="3">STATISTICAL METHODS:
Primary Efficacy Endpoint

The primary efficacy endpoint is the percent change in LDL-C from Baseline to Week 28/Early Termination (ET represents the observation closest to 2 weeks after last dose among patients who early terminate study medication dosing).

Secondary Efficacy Endpoints

Secondary efficacy endpoints include the percent change in apoB, total cholesterol, non-HDL-C, TG, HDL-C, and Lp(a) from Baseline to Week 28/ET.

Statistical Analyses

Efficacy Analyses:

The primary efficacy analysis will be performed on the Full Analysis Set, which represents the practically-feasible intent-to-treat (ITT) population as delineated in ICH Guideline E9, and will be</td></tr>
</table>

NAME OF COMPANY<br>Genzyme Corporation<br>500 Kendall Street<br>Cambridge, MA 02142<br>USA<br><br>Genzyme Europe BV<br>Gooimeer 10<br>1411 DD Naarden<br>The Netherlands<br><br>**NAME OF FINISHED PRODUCT**<br>mipomersen sodium injection/ISIS 301012 Injection, 200 mg/mL, 1.0 mL<br>**NAME OF ACTIVE INGREDIENT**<br>mipomersen sodium/ISIS 301012 Drug Substance	**SUMMARY TABLE**<br>Referring to Part ……<br>of the Dossier:<br>Volume:<br>Page:<br>Reference:	**FOR NATIONAL AUTHORITY USE ONLY:**

comprised of all patients who receive at least 1 injection and have at least 1 post-Baseline LDL-C measurement. In addition, the primary efficacy analysis will be performed on the subset of patients with LDL-C of at least 200 mg/dL (5.1 mmol/L), for patients with CHD, or at least 300 mg/dL (7.8 mmol/L), for patients without CHD, representing a patient population in which LDL-C apheresis is indicated in the USA.

Percent change from Baseline to Week 28 in LDL-C, apo B, total cholesterol, HDL-C, non-HDL-C, TG, and Lp(a) will be compared between treatment groups using a 2-sample t-test (if data departs substantially from normality, a Wilcoxon rank sum test will be used). The analysis will take place after all patients have completed Week 28 and the database has been locked.

Safety Analyses:

The safety analyses will be performed on the Safety Set defined as all patients who receive at least 1 injection. Treatment duration and amount of study drug received will be summarized by treatment group, as will concomitant medications and treatments. Treatment-emergent AEs will be summarized by body system, preferred term, severity and relationship to the study procedures or treatments. Additionally listings of SAEs and AEs leading to discontinuation will be generated. Clinical laboratory evaluations, vital signs, electrocardiogram (ECG), and physical examination findings will be tabulated by treatment group. In addition, the number of patients who experience abnormalities in clinical laboratory evaluations will be summarized by treatment group.

Plasma concentrations and elimination half-life of mipomersen will be summarized over time by individual patient and by treatment group

Power and Sample Size

Based upon prior clinical trial experience with mipomersen, it is estimated that the standard deviation of the percent change in LDL-C is approximately 22%. With 45 randomized patients (15 patients in the control group and 30 patients in the mipomersen-treated group), there would be 80% power to detect a 20 percentage point difference between the 2 groups. Enrollment will be conducted such that at least 51 patients are randomized (to allow for potential exclusions from an analysis set) with LDL-C of at least 200 mg/dL (5.1 mmol/L), for patients with CHD, or at least 300 mg/dL (7.8 mmol/L), for patients without CHD, representing a patient population in which LDL-C apheresis is indicated in the USA. Additionally, approximately 24 patients with LDL-C of at least 190 mg/dL (4.9 mmol/L) will be randomized to provide additional exposure among a slightly broader patient population. Therefore, approximately 75 patients will be randomized.

2 TABLE OF CONTENTS

1 SYNOPSIS 3
2 TABLE OF CONTENTS 10
3 ABBREVIATIONS AND TERMS 14
4 INTRODUCTION 17
4.1 ApoB-100 as a Therapeutic Target for Hypercholesterolemia 18
4.2 Mipomersen 19
4.2.1 Mechanism of Action 19
4.2.2 Chemistry 19
4.3 Summary of Benefits and Risks 20
5 STUDY OBJECTIVES 21
6 INVESTIGATIONAL PLAN 22
6.1 Study Design 22
6.2 Endpoints 23
6.2.1 Efficacy Endpoints 23
6.2.1.1 Primary Efficacy Endpoint 23
6.2.1.2 Secondary Efficacy Endpoints 23
6.2.2 Safety Endpoints 23
6.3 Discussion of Study Design Including Choice of Control Group 23
7 PATIENT POPULATION AND SELECTION 25
7.1 Inclusion Criteria 25
7.2 Exclusion Criteria 26
7.3 Patient Withdrawal 27
8 TREATMENTS 29
8.1 Treatments Administered 29
8.2 Investigational Product 30
8.2.1 Packaging and Labeling 30
8.2.2 Study Drug Preparation 30
8.2.3 Drug Storage 30
8.3 Prior and Concomitant Medications 30
8.4 Method of Assigning Patients to Treatment 31
8.5 Dose Selection 31
8.6 Blinding and Randomization 31
8.6.1 Unblinding 32
9 EFFICACY AND SAFETY VARIABLES 33
9.1 Efficacy and Safety Measurements Assessed and Study Flowchart 33
9.1.1 Screening Assessments 38
9.1.1.1 Medical History, Physical Examinations, 12-Lead Electrocardiogram 38
9.1.1.2 Clinical Laboratory Evaluations and Therapeutic Lifestyle Changes 38
9.1.2 Treatment Period Assessments 39
9.1.2.1 Magnetic Resonance Imaging and Computed Tomography 40
9.1.2.2 Clinical Laboratory Evaluations 40

9.1.2.3 Demographics, Vital Signs, Body Weight, and Anthropometric Measurements 43
9.1.2.4 Pharmacokinetic Trough Levels 43
9.1.2.5 Electrocardiogram 43
9.1.2.6 Physical Examination 44
9.1.2.7 Concomitant Medications 44
9.1.2.8 Injection Site Reaction Assessment 44
9.1.3 Post-Treatment Evaluation Period Assessments 44
9.2 Adverse Events 45
9.2.1 Definition of Adverse Events 45
9.2.2 Definition of Serious Adverse Events 45
9.2.3 Evaluation of Adverse Events and Serious Adverse Events 46
9.2.3.1 Severity of Adverse Events 46
9.2.3.2 Action Taken/Medication or Therapy Given 47
9.2.3.3 Outcome of Adverse Events 47
9.2.3.4 Relationship to Study Treatment 48
9.2.3.5 Clinical Laboratory Tests 48
9.2.3.6 Dosing Errors 48
9.2.4 Adverse Experience and Serious Adverse Experience Reporting 49
9.2.4.1 Adverse Experience Reporting 49
9.2.4.2 Serious Adverse Event Reporting 49
9.2.5 Pregnancy Reporting 50
9.2.6 Stopping Rules and Safety Monitoring 51
9.2.6.1 Confirmation Guidance 51
9.2.6.2 Stopping Rule Guidance 52
9.2.6.3 Liver Chemistry Rules 52
9.2.6.4 Safety Monitoring Rules for Renal Function Test Results 54
9.2.6.5 Safety Monitoring Rule for Platelet Count Results 54
9.2.6.6 Safety Monitoring Rules for Coagulation Parameters 54
9.2.6.7 Safety Monitoring Rule for Constitutional Symptoms 54
9.2.6.8 Restriction on the Lifestyle of Patients 54
9.2.7 Summary of Risks 55
10 DATA COLLECTION, QUALITY ASSURANCE, AND MANAGEMENT 55
10.1 Recording of Data 55
10.2 Data Quality Assurance 55
10.3 Data Management 56
11 STATISTICAL METHODS AND PLANNED ANALYSES 57
11.1 Power and Determination of Sample Size 57
11.2 Demographics and Baseline Characteristics 58

11.3 Patient Accountability 58
11.4 Study Treatment Usage and Compliance 58
11.5 Efficacy 58
11.5.1 Primary Efficacy Endpoint 58
11.5.2 Secondary Efficacy Endpoints 58
11.5.3 Efficacy Analysis 58
11.6 Safety 59
11.6.1 Safety Endpoints 59
11.6.2 Safety Analysis 59
11.7 Statistical Analysis Plan 59
12 SPECIAL REQUIREMENTS AND PROCEDURES 60
12.1 Institutional and Ethical Review 60
12.2 Changes to the Conduct of the Study or Protocol 60
12.3 Investigator's Responsibilities 60
12.3.1 Patient Informed Consent 60
12.3.2 Electronic Case Report Forms 61
12.3.3 Record Retention 61
12.3.4 Patient Discontinuation 62
12.3.5 Study or Site Termination 62
12.3.6 Monitoring 62
12.3.7 Materials Control 62
12.3.7.1 Receipt of Clinical Supplies 62
12.3.7.2 Disposition of Unused Clinical Supplies 63
12.3.8 Warnings, Precautions, Contraindications 63
12.3.9 Clinical Study Report 63
12.3.10 Disclosure of Data 63
13 REFERENCES 64
14 APPENDICES 66
14.1 NCEP – ATP III Therapeutic Lifestyle Changes 67
14.2 Lipoprotein Testing 68
14.3 NCEP - ATP III Guidelines for Determining >20% 10-Year Risk for CHD 69
14.4 Pregnancy Notification Forms 74

LIST OF IN-TEXT TABLES

Table 9-1 Schedule of Study Events 34
Table 9-2 List of Screening Clinical Laboratory Evaluations 39
Table 9-3 List of Treatment Period Clinical Laboratory Evaluations 42
Table 9-4 Contact List for Serious Adverse Event Reporting 50
Table 14-1 Nutrient Composition of the TLC Diet 67
Table 14-2 Lipoprotein Testing 68

LIST OF IN-TEXT FIGURES

Figure 4-1 Design of Chimeric 2´-MOE Phosphorothioate Oligonucleotides (MOE-Gapmer) 20

Figure 6-1 Study Design 23

3 ABBREVIATIONS AND TERMS

AE	Adverse event
AICD	Automatic implantable cardioverter defibrillator
ALT	Alanine aminotransferase (SGPT)
ANA	Antinuclear antibody
apo	Apolipoprotein
aPTT	Activated Partial Thromboplastin Time
ASO	Antisense oligonucleotide
AST	Aspartate aminotransferase (SGOT)
ATP	Adult Treatment Panel
BMI	Body Mass Index
BP	Blood pressure
BUN	Blood urea nitrogen
C3	Complement
CABG	Coronary artery bypass graft
CAD	Coronary artery disease
CHD	Coronary heart disease
C_{min}	Minimum plasma concentration
CMV	Cytomegalovirus
CPK	Creatine phosphokinase
eCRF	Electronic Case Report Form
CS	Clinically significant
CT	Computed tomography
CTTC	Cholesterol Treatment Trialists' Collaboration
CVA	Cerebrovascular accident
DMC	Data Monitoring Committee
DNA	Deoxyribonucleic acid
EBV	Epstein-Barr Virus
ECG	Electrocardiogram
EDC	Electronic data capture
ESR	Erythrocyte sedimentation rate
ET	Early termination
GCP	Good Clinical Practice
HAV	hepatitis A virus
HBsAg	Hepatitis B surface antigen

hCG	Human chorionic gonadotropin
HCV	hepatitis C virus
HDL-C	High density lipoprotein cholesterol
HEENT	Head, ears, eyes, nose, throat
HIV	Human immunodeficiency virus
HbA_{1c}	Glycosylated hemoglobin A
HMG-CoA	3-hydroxy-3-methylglutaryl coenzyme A
hsCRP	High-sensitivity C-reactive protein
HSV	Herpes simplex virus
ICH	International Conference on Harmonisation
IEC	Independent Ethics Committee
IgG	Immunoglobulin G
IgM	Immunoglobulin M
INR	International normalized ratio (for anticoagulant monitoring)
IRB	Institutional Review Board
ISR	Injection site reaction
ITT	Intent-to-treat
IVRS	Interactive voice response system
LDL	Low density lipoprotein
LDL-C	Low density lipoprotein cholesterol
LFT	Liver function test
Lp(a)	Lipoprotein a
MCV	Mean corpuscular volume
MedDRA	Medical Dictionary for Regulatory Activities
MI	Myocardial Infarction
MOE	O-(2-Methoxyethyl)-D-Ribose
MRI	Magnetic resonance imaging
mRNA	Messenger Ribonucleic Acid
NCEP	National Cholesterol Education Program
NCS	Not Clinically Significant
NEPTUNE II	National Cholesterol Education (NCEP) Program Evaluation Project Utilizing Novel E-Technology II
NYHA	New York Heart Association
P/C	Urine protein to creatinine ratio
PK	Pharmacokinetic(s)
PT	Prothrombin Time

PCI	Percutaneous coronary intervention
PTT	Partial thromboplastin time
QA	Quality Assurance
QRS	Part of ECG wave representing ventricular depolarization
QS	A sufficient quantity to make
RR	Inter-beat interval
SAE	Serious Adverse Event
SC	Subcutaneous
SPECT	Single photon emission computed tomography
Study Drug	mipomersen or placebo
T_4	Thyroxine
TG	Triglycerides
TLC	Therapeutic Lifestyle Changes
TSH	Thyroid Stimulating Hormone
UAA	Translation stop codon
ULN	Upper Limit of Normal
USA	United States of America
VLDL	Very low density lipoprotein
VLDL-C	Very low density lipoprotein cholesterol
WBC	White blood cells
WHR	Waist-to-hip ratio

4 INTRODUCTION

Hypercholesterolemia is a common condition that in its untreated form is categorized by a broad range of elevated low density lipoprotein cholesterol (LDL-C) concentrations. Its etiology is mixed and although environmental contributors such as diet play a role, there is a strong genetic component especially in those patients who have LDL-C concentrations in the higher ranges. Monogenic forms of hypercholesterolemia include the different varieties of familial hypercholesterolemia, but there are also thought to be many polygenic contributors.

Elevated LDL-C is a major risk factor for coronary heart disease (CHD) and in the last years, results of observational and interventional studies in primary and secondary prevention indicate a continuous positive relationship between coronary artery disease (CAD) risk and blood cholesterol concentrations (National Cholesterol Education Program [NCEP] Adult Treatment Panel III [ATP III]) (Grundy, 2004, *Circulation*; NCEP, 2002, *Circulation*; see Appendix 14.3). The strong association between LDL-C concentrations and CHD risk is attributed to the central role of LDL-C in the development and progression of atherosclerotic lesions, the underlying cause of CHD. Low density lipoprotein is a major source of lesion cholesterol and stimulates inflammatory processes involved in lesion development (Davis, 2001, *Arterioscler Thromb Vasc Bio.*; Hulthe, 2002, *Arterioscler Thromb Vasc Biol*; Skalen, 2002, *Nature*). In addition reducing LDL-C is associated with improved outcomes in many studies. A meta-analysis of many randomized controlled outcome studies indicated that a 1 mmol/L reduction in LDL-C resulted in a 23% reduction in vascular events across a range of end of treatment LDL-C concentrations (Cholesterol Treatment Trialists' Collaboration [CTTC], 2005, *Lancet*). Because of the increased risk of elevated LDL-C plasma concentrations, LDL-C reduction has been the principal goal of CHD prevention strategies (Knopp, 1999, *N Engl J Med*; Gotto, 2002, *Am J Med*). The goals for lipid-lowering therapy were established by the NCEP and were updated most recently in 2004 (Grundy, 2004, *Circulation*). The NCEP guidelines define 3 major categories of risk for clinical events and provide risk-based LDL-C targets. Those at highest risk are patients with CHD or CHD-risk equivalent conditions and should be treated to LDL-C targets < 100 mg/dL (<2.6 mmol/L) or lower (< 70 mg/dL [< 1.8 mmol/L]). CHD-risk equivalent conditions include diabetes, peripheral vascular disease, abdominal aortic aneurysm, symptomatic carotid artery disease, metabolic syndrome, and multiple risk factors that confer a 10-year risk for CHD events > 20%.

There are currently 5 classes of approved therapeutic agents for hyperlipidemia, all of which are administered orally. These include the bile-acid sequestrants, fibrates, niacin, statins, and cholesterol absorption inhibitors. The choice of therapy is individualized to accommodate the needs of the patient although the statins are the overwhelming therapy of choice due to

the extent of LDL-C reductions achieved and the wealth of evidence of benefit. The statins are competitive inhibitors of 3-hydroxy-3-methylglutaryl coenzyme A (HMG-CoA) reductase, the rate-limiting step in cholesterol biosynthesis. Administration of these drugs also leads to increased hepatic low density lipoprotein (LDL) receptor expression and enhanced cholesterol clearance. In addition, the statins appear to have pleiotropic effects, including anti-inflammatory, anti thrombotic, and anti-proliferative properties that may prevent plaque growth and rupture. Despite the availability and clinical effectiveness of these agents, recent data (Sueta, 2003, *J Cardiac Failure*) indicate that a significant proportion of patients do not attain target LDL-C values and thus that cardiovascular mortality rates remain high, especially in high-risk patients. Studies such as the NCEP Program Evaluation Project Utilizing Novel E-Technology II (NEPTUNE II) show that a large number of high-risk patients are not sufficiently treated. Of patients classified as very high risk, only 18% were at the optional therapeutic LDL-C goal of < 70 mg/dL (< 1.8 mmol/L) with 60% achieving concentrations < 100 mg/dL (2.6 mmol/L) (Davidson, 2005, *Am J Cardiol*). In individuals with CHD or CHD equivalent who did not meet their targets, average LDL-C concentrations were 125 mg/dL (3.2 mmol/L). More stringent LDL-C lowering goals, coupled with a clearer understanding of the molecular basis of various types of dyslipidemia, underscore the need for the development of new therapeutics.

4.1 ApoB-100 as a Therapeutic Target for Hypercholesterolemia

Apolipoproteins (apo) are a family of structural proteins present on the surface of lipoproteins that are important for the regulation of lipid transport and metabolism. They were named in an arbitrary alphabetical order and include apoA, B, C and E. ApoB exists in the plasma in 2 main isoforms, apoB-48 and apoB-100. In man, the apoB-48 is synthesized exclusively by the gut while apoB-100 is synthesized by the liver (Powell, 1987, *Cell*). The intestinal (B-48) and hepatic (B-100) forms of apoB are produced from a single gene and a single messenger ribonucleic acid (mRNA) transcript. This results from an organ-specific apoB mRNA editing enzyme (apobec-1), which generates a stop codon within the mRNA, changing CAA at position 2153 base pairs to a translation stop codon (UAA) (Chen, 1987, *Science*). Translation of the edited transcript yields a truncated protein product similar in composition to the N-terminal half of apoB 100. From structural studies, it is thought that apoB-48 represents the amino terminal 47% of apoB 100.

ApoB-100 is the major apolipoprotein of very low density lipoprotein (VLDL), intermediate-density lipoprotein, and LDL, comprising approximately 30%, 60%, and 95% of the protein in these lipoproteins, respectively. ApoB-100 is essential for the assembly and secretion of VLDL from the liver. Lipids such as triglycerides (TG) and cholesterol are packaged with apoB-100 and other phospholipids into VLDL, which in turn is secreted into the plasma,

where additional apolipoproteins are added. Thus, inhibition of apoB-100 would be expected to impair VLDL synthesis and result in lower concentrations of LDL-C. Indeed, individuals who are unable to translate full length apoB-100 have plasma concentrations of apoB and LDL-C that are 25% to 50% of normal (Sankatsing, 2005, *Arterioscler Thromb Vasc Biol*).

4.2 Mipomersen

4.2.1 Mechanism of Action

Antisense oligonucleotides (ASO) are designed to be complementary to a segment of a target mRNA. ASO binding to mRNA is dependent on Watson and Crick base-pairing rules and is, therefore, highly specific for the target sequence. Binding of the ASO to its cognate mRNA results in RNase H-mediated degradation of the cognate mRNA thus inhibiting mRNA translation into protein. First generation oligonucleotides are nearly identical to endogenous deoxyribonucleic acid (DNA), differing only in the replacement of sulfur for 1 of the non-bridging oxygens in each of the phosphate linkages of DNA to form phosphorothioate linkages. Phosphorothioate linkages are more resistant than phosphodiester linkages to nuclease degradation and thus make the ASOs more resistant to metabolism by nucleases. Second generation ASOs have the same phosphorothioate linkages, but in addition, the nucleotides are modified at the 2' position of the ribose with 2'-O-(2-methoxyethyl)-D-ribose (2'-MOE). The MOE modification (1) increases affinity towards the cognate mRNA, (2) increases resistance to exonucleases and endonucleases (thereby increasing tissue half-life), and (3) ameliorates some of the high-dose toxicities associated with first generation ASOs. Increased affinity results in potency that is increased compared to first-generation ASOs.

4.2.2 Chemistry

Mipomersen is an antisense drug targeted to human apoB-100, the principal apolipoprotein of atherogenic LDL and its metabolic precursor, VLDL. Mipomersen is complementary to the coding region of the mRNA for apoB-100, binding by Watson and Crick base pairing. The hybridization (binding) of mipomersen to the cognate mRNA results in RNase H-mediated degradation of the cognate mRNA thus inhibiting translation of the apoB-100 protein.

Mipomersen is the nonadecasodium salt of a 20-base (20-mer) phosphorothioate oligonucleotide. Each of the 19 internucleotide linkages is a 3′-O to 5′-O phosphorothioate diester. Ten of the 20 sugar residues are 2-deoxy-D-ribose, the remaining 10 are 2'-MOE. The 2′-MOE modification improves binding affinity for the target mRNA while increasing stability against nuclease-mediated degradation relative to 2′-deoxyribonucleosides. However, since the 2′ MOE modification reduces RNase H activity, a chimeric oligonucleotide strategy was employed in which 2′-deoxyribonucleosides that support RNase H activity are flanked by nuclease resistant 2′-MOE ribonucleosides. These modifications are commonly known as second-generation ASO, and the chimeric design of mipomersen

improves its pharmacologic profile (relative to 2′ deoxyribonucleosides) while preserving RNase H degradation of the target mRNA. Mipomersen is also referred to as a 5-10-5 MOE gapmer where the term gap refers to the ten 2′ deoxyribonucleosides that are necessary to support enzymatic cleavage of the cognate mRNA.

The sequence of mipomersen is shown (note that all cytosine residues are methylated at the 5-position).

Figure 4-1 Design of Chimeric 2′-MOE Phosphorothioate Oligonucleotides (MOE-Gapmer)

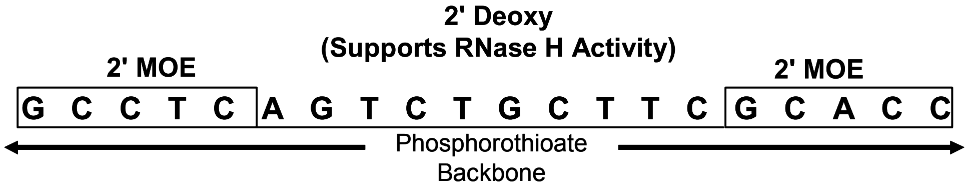

4.3 Summary of Benefits and Risks

Please refer to the Investigator's Brochure for additional information on clinical and nonclinical studies, and any known potential risks and benefits to humans.

5 STUDY OBJECTIVES

The objective of this study is to compare the safety and efficacy of 26 weekly, subcutaneous (SC) injections of mipomersen (200 mg) against placebo in treating severely hypercholesterolemic patients who are on a maximally tolerated lipid-lowering regimen and who are not on apheresis.

6 INVESTIGATIONAL PLAN

6.1 Study Design

This is a prospective, randomized, double-blind, placebo-controlled, parallel-group, multicenter Phase 3 study to investigate the safety and efficacy of mipomersen to treat severe hypercholesterolemia. Approximately 75 severely hypercholesterolemic adult patients who are on a maximally tolerated lipid lowering regimen with LDL-C ≥ 190 mg/dL (4.9 mmol/L), and who are not on apheresis will be randomized into this study. Patient enrollment will be conducted such that at least 51 patients are randomized with LDL-C of at least 200 mg/dL (5.1 mmol/L) for patients with CHD, or at least 300 mg/dL (7.8 mmol/L) for patients without CHD, representing a patient population in which LDL-C apheresis is indicated in the United States of America (USA). Additionally, approximately 24 patients with LDL-C of at least 190 mg/dL (4.9 mmol/L) will be randomized to provide additional exposure among a slightly broader patient population.

Patients will be randomized in a 2:1 ratio to receive mipomersen or placebo, respectively. Mipomersen or a matching volume of placebo will be administered weekly via SC injection for 26 doses.

The study will be conducted in compliance with International Conference on Harmonisation (ICH), Good Clinical Practice (GCP) guidelines as well as in accordance with all national, state and local laws of the appropriate regulatory authorities. The duration of the study, including completing patient enrollment, is expected to be approximately 18 months. Each patient will participate in a ≤ 4-week screening period and a 26-week treatment period. All patients, including those who discontinue prematurely and have received ≥ 1 dose of study drug, should be followed for safety for 24 weeks after their last dose of study drug as part of the post-treatment evaluation period (Figure 6-1).

An independent data monitoring committee (DMC) will provide an ongoing, expert, independent review of safety data to assure that the risks to study patients are minimized. This ongoing review will include pre-specified review of safety data during the conduct of the study as defined by the DMC Charter. Based on these data, the DMC may recommend changes in study conduct to Genzyme. As part of the conduct of this study, Genzyme will periodically evaluate the nature, frequency and severity of AEs that have been identified as potential risks associated with the use of mipomersen or other new observations.

The end of study is defined as the last patient's last visit.

Figure 6-1 Study Design

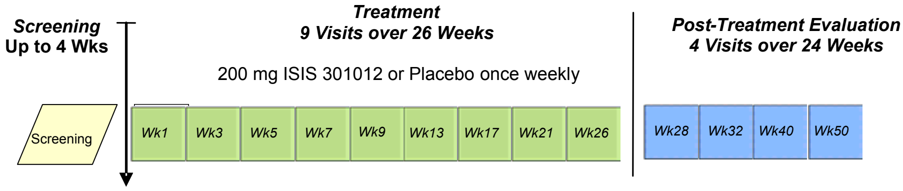

Randomization (2:1)
200 mg mipomersen (n=50) to Placebo (n=25)
Stratification: ≥ 51 patients with CHD and LDL-C ≥ 200 mg/dL (5.1 mmol/L) or without CHD but with LDL-C ≥ 300 mg/dL (7.8 mmol/L); 24 patients with LDL-C≥ 190 mg/dL (4.9 mmol/L)

6.2 Endpoints

6.2.1 Efficacy Endpoints

6.2.1.1 Primary Efficacy Endpoint

The primary efficacy endpoint is the percent change in LDL-C from Baseline to Week 28/Early Termination (ET represents the observation closest to 2 weeks after last dose among patients who terminate study medication dosing early).

6.2.1.2 Secondary Efficacy Endpoints

Secondary efficacy endpoints include the percent change in apoB, total cholesterol, high density lipoprotein cholesterol (HDL-C), non-HDL-C, TG, and lipoprotein a (Lp[a]) from Baseline to Week 28/ET.

6.2.2 Safety Endpoints

Safety will be determined using the incidence of treatment-emergent adverse events (AEs), clinical laboratory evaluations, vital signs, electrocardiograms (ECGs), and physical examination findings. Adverse events will be categorized using the Medical Dictionary for Regulatory Activities (MedDRA). Plasma concentrations of mipomersen will be evaluated.

6.3 Discussion of Study Design Including Choice of Control Group

This is a randomized, double-blind, placebo-controlled, parallel-group study with 24 weeks of treatment and 6 months of safety follow-up. This treatment duration and follow-up has been chosen as it is roughly 5 times the terminal elimination half-life of mipomersen, so that the efficacy endpoint can be evaluated when drug tissue levels are expected to be at > 90% of steady-state values and the safety assessments carried out when plasma trough levels are close to 0.

Patient randomization will be stratified based on LDL-C concentration at Screening, in order to represent a patient population in which LDL-C apheresis is indicated in the USA. Additionally, approximately 24 patients with LDL-C of at least 190 mg/dL (4.9 mmol/L) will be randomized to provide additional exposure among a slightly broader patient population.

It is considered appropriate to treat the control group with placebo as these patients will also be receiving a maximally tolerated lipid-lowering regimen including statin (the gold standard of care), diet, and a medication from a second class of hyperlipidemic agents.

Based on nonclinical and clinical findings discussed in the Investigator's Brochure, close monitoring of safety laboratory parameters has been stipulated within the protocol particularly focusing on liver function tests (LFTs; also including imaging of the liver and spleen by magnetic resonance imaging [MRI]) or computed tomography (CT), and clotting parameters. The protocol also specifies under what conditions the implementation of clear stopping rules are to be employed to protect patients from unnecessary exposure to study medication. The LFT stopping rules are adapted from the draft guidance for industry, "Drug-Induced Liver Injury: Premarketing Clinical Evaluation," issued by the U.S. Department of Health and Human Services, Food and Drug Administration, October 2007.

Injection site reactions (ISRs) will be closely monitored. Inflammatory markers will be measured regularly.

Finally, the overall safety of the study will be monitored by an independent DMC.

7 PATIENT POPULATION AND SELECTION

Approximately 50 study centers will enroll approximately 75 patients in this protocol.

7.1 Inclusion Criteria

Patients must fulfill all of the following criteria:

1. Age ≥ 18 years
2. Fasting LDL-C ≥ 190 mg/dL (4.9 mmol/L) and TG < 350 mg/dL (4.0 mmol/L) at Screening
3. Presence of at least 1 of the following criteria for coronary disease:
   a) Myocardial infarction (MI)
   b) Percutaneous coronary intervention (PCI) or coronary artery bypass graft (CABG)
   c) CAD documented by angiography or any other accepted imaging technique
   d) If 1 or more of criteria a) through c) are not met: a positive exercise test (≥ 1 mm ST-depression at maximal exercise or test terminated because of angina) or a perfusion defect, e.g., thallium or single photon emission computed tomography (SPECT)
   e) Or, if a) through d) are not met, LDL-C>300 mg/dL (7.8 mmol/L)
4. On a stable, maximally tolerated lipid-lowering regimen and expected to remain on it through Week 28 (must satisfy all criteria):
   a) A statin at a maximally tolerated dose per Investigator judgment, for at least 8 weeks prior to Screening
   b) A stable low-fat diet (e.g., NCEP-ATP III therapeutic lowering cholesterol [TLC] or equivalent) beginning at least 12 weeks prior to the first dose of study drug
   c) A medication from an additional class of hypolipidemic agents, per Investigator's judgment (e.g., bile acid sequestrants, niacin/nicotinic acid, fibrates) for at least 8 weeks prior to Screening
5. Body mass index (BMI) ≤ 40 kg/m^2 with weight stable (± 4 kg) for > 6 weeks prior to Screening per patient report.
6. Satisfy 1 of the following:
   a) Females: Non-pregnant and non-lactating; surgically sterile, postmenopausal, or patient or partner compliant with an acceptable contraceptive regimen for 4 weeks prior to Screening, during the treatment phase, and 24 weeks after the last study drug dose
   b) Males: Surgically sterile or patient or partner is utilizing an acceptable contraceptive method during the treatment phase and 24 weeks after the last study drug dose
7. Given informed consent

7.2 Exclusion Criteria

Patients meeting any of the following criteria will be excluded from the study:

1. Any of the following diagnoses, conditions, or prior treatments:
   a) MI, PCI, CABG, cerebrovascular accident (CVA), unstable angina or acute coronary syndrome within 24 weeks of Screening
   b) Presence of clinically significant arrhythmias, implantable pacemakers or automatic implantable cardioverter defibrillators (AICDs), or currently taking any medication for arrhythmias
   c) Type 1 diabetes mellitus
   d) New York Heart Association (NYHA) functional classification III or IV heart failure
   e) Hypertension, systolic blood pressure (BP) ≥ 160 mmHg, or diastolic BP ≥ 95 mmHg at Screening (despite antihypertensive medication/therapy)
   f) Active infection requiring systemic antimicrobial therapy
   g) Positive test for human immunodeficiency virus (HIV) or hepatitis B or C at Screening
   h) Any uncontrolled condition that may predispose the patient to secondary hyperlipidemia such as uncontrolled hypothyroidism.
   i) Malignancy within 5 years, except for basal or squamous cell carcinoma of the skin that has been adequately treated
   j) Clinically significant hepatic or renal disease or Gilbert’s syndrome
   k) Apheresis within 3 months prior to Screening or expected to start apheresis during the treatment phase
2. The following laboratory values at Screening:
   a) Serum creatine phosphokinase (CPK) ≥ 3 x upper limit of normal (ULN)
   b) Alanine aminotransferase (ALT) levels > 1.5 x ULN
   c) Serum creatinine > 0.1 mg/dL (> 8.8 µmol/L) above ULN for women, or > 0.2 mg/dL (> 17.7 µmol/L) above ULN for men
   d) Proteinuria (> 1+ on dipstick, confirmed on retest, with further confirmation by quantitative total urine protein > 1.0 g/24 hr)
   e) Total Bilirubin > 1.0 x ULN
   f) Glycosylated hemoglobin A (HbA_{1C}) > 8.0%
3. Use of the following medications within 12 weeks of Screening:
   a) Medications that may affect lipids except those allowed per the protocol, including but not limited to Cholestin™ (also known as red yeast rice, or monascus purpureus extract)
   b) Anti-obesity medications

   c) Chronic systemic corticosteroids or anabolic agents except for replacement therapy
   d) Oral contraceptives unless prospectively discussed with the Medical Monitor
   e) Fibrates unless prospectively discussed with the Medical Monitor
4. Use of the following medications unless a stable dose regimen was used for at least 12 weeks prior to Screening and the dose and regimen are expected to remain stable until Week 28:
   a) Oral anticoagulants (e.g., warfarin)
   b) Hormone replacement therapy
   c) Diabetes medications including but not limited to sulfonylureas, metformin and glitazones, with the exception of changes of ± 10 units of insulin.
   d) Antiviral therapy for herpes simplex virus (HSV)
5. Treatment with another investigational drug, biological agent, or device within 4 weeks of Screening or 5 half-lives of study agent, whichever is longer
6. Recent history of, or current, drug or alcohol abuse, or unwillingness to limit alcohol consumption for the entire duration of the study, including follow-up
7. Any disorders that would limit study participation or unwillingness to comply with study procedures, including follow-up, as specified by this protocol, or unwillingness to cooperate fully with the Investigator
8. Have any other medical conditions that, in the opinion of the Investigator, would make the patient unsuitable for enrollment, or could interfere with the patient participating in or completing the study

7.3 Patient Withdrawal

Patients have the right to withdraw from the study at any time and for any reason without prejudice to their ongoing or future medical care.

Any patient who withdraws consent to participate in the study will be removed from further treatment and/or study observation immediately upon the date of request.

Should a patient (or a legally acceptable representative) request or decide to withdraw from the study, all efforts will be made to complete and report the observations as thoroughly as possible up to the date of withdrawal, and an Early Termination visit should be performed. All information should be reported on the applicable electronic Case Report Forms (eCRFs).

Patients who request to stop study drug or have been withdrawn from study treatment at the request of the Investigator or Sponsor before completion of the protocol-specified treatment period, and who have received at least 1 dose of study drug and have not revoked their

consent to participate in the study, will be strongly encouraged to continue the post-treatment evaluation period beginning with the Week 28 visit assessments. This visit should be scheduled within 2 weeks of their last dose of study drug. For those patients who withdraw from the study prior to receiving any dose of study drug, no further follow up is necessary.

Patients who request to withdraw from the study during the post-treatment evaluation period should be encouraged to undergo clinical laboratory and safety evaluations including, but not limited to, hematology and chemistry panels, urinalysis, pregnancy test (for women of childbearing potential), vital signs collection and a physical examination prior to leaving the study (if it has been longer than 4 weeks since these procedures were last performed).

The Investigator must record the reason for withdrawal on the Study Termination eCRF.

If the patient withdraws due to an AE, the Investigator should arrange for the patient to have follow-up visits until the AE has resolved or stabilized.

Reasons for removal from study drug or observation may include:

- Withdrawal of consent
- Administrative decision by the Investigator or Sponsor
- Pregnancy (report on Pregnancy Notification Forms)
- Ineligibility
- Significant protocol deviation
- Patient non-compliance
- The patient experiences an AE that is considered intolerable by the patient or Investigator (includes disease progression; report on AE eCRF)

8 TREATMENTS

8.1 Treatments Administered

Patients will be randomized in a 2:1 ratio (i.e., mipomersen:placebo)

Mipomersen: 1-mL weekly injections of 200 mg of mipomersen will be administered for 26 weeks.

Placebo: 1-mL weekly injections of placebo (i.e., vehicle consisting of 9 mg of sodium chloride, 0.004 mg of riboflavin, filled to (QS) 1 mL with water for injection) for 26 weeks.

Injections will be administered as SC injections into the outer area of the upper arm, the medial thigh region, or the abdomen. The first 3 doses must be supervised by a health professional, including study center personnel or home health nurse. Thereafter, patients will be given the option to self-administer study drug injections unsupervised with the exception of doses on clinic visit days.

A specific administration schedule will be used in this study in order to gain some understanding about the patient's preference of injection site and the reason for the choice. For the first 3 injections, the injection site will be specified for all patients. The first injection will be in the outer area of the upper arm, the second injection will be in the medial thigh region, and the third injection will be in the abdomen. Based on the patient's experience from the first 3 doses, each will be allowed to choose the injection site for their remaining injections. The chosen site and the reason for the choice, (e.g., tolerability, convenience, physician recommendation, aesthetic, no reason, etc.) will be recorded on the eCRF. The patient may choose a different injection site at any time during the remainder of the treatment period. The new injection site and the reason for the change should be recorded on the eCRF. The patient's opinion on injection site location will also be collected at the conclusion of the treatment period.

The dose schedule may be adjusted in the event a patient is unable to come to the clinic due to vacations or holidays as follows:

- Patients can make up for a missed dose by receiving 2 doses of study drug in the week before or the week after the missed dose(s) provided that the 2 doses are given at least 3 days apart.
- Patients should be strongly discouraged from missing more than 2 scheduled doses of study drug in the first 13 weeks of dosing, more than 3 scheduled doses in the 26-week treatment period, and more than 1 of the last 4 scheduled doses of study drug.
- If the Investigator or the study coordinator is aware that a patient will be missing more than the recommended number of scheduled visits during the dosing period, the patient should be excluded from the study.

Dosing details must be recorded in the patient's source documents and the eCRF.

8.2 Investigational Product

Mipomersen is supplied as 200 mg/mL of mipomersen (ISIS 301012), with 1 mL of solution per vial.

Placebo is the vehicle, consisting of 9 mg of sodium chloride, 0.004 mg of riboflavin, QS to 1 mL with water for injection.

8.2.1 Packaging and Labeling

The study drug described in Section 8.2 is contained in 2-mL stoppered glass vials that will be provided to the study center by the Sponsor or designee. Mipomersen active pharmaceutical ingredient is manufactured by Isis Pharmaceuticals, Inc., Carlsbad, California, USA.

The Sponsor or designee will provide the Investigator with packaged study drug labeled in accordance with specific country regulatory requirements.

8.2.2 Study Drug Preparation

The study drug must be brought to room temperature prior to administration. Using aseptic technique, withdraw 1 mL from the vial of mipomersen (ISIS 301012) or placebo. Refer to the Dosing Instructions in the Investigational Product Handling Manual for additional details.

8.2.3 Drug Storage

The study drug must be stored securely at 2° to 8° Celsius and be protected from light in a limited access area.

8.3 Prior and Concomitant Medications

Reasonable efforts will be made to determine all medications and treatments (pharmacological and non-pharmacological) received by the patient. Prior medications or treatments are defined as any medication or therapy taken by the patient within 1 month prior to Screening. In addition, an attempt will be made to collect data on any lipid-lowering medications taken within 1 year prior to Screening. A medication or treatment is considered concomitant if it is taken at any time after the Screening visit up to and including the day of the final study evaluation. Data on medications will include: name, dose, route, regimen, start date, stop date, and indication. Data on treatments will include: treatment, start date, stop date, and indication. At each study visit, the patient will be asked about any additional medications or treatments or any changes in regimen or dosages since the last visit. Indications for any new medications or treatments during the study period will be recorded as an AE.

All concomitant lipid-lowering drugs are to remain constant from Screening through Week 28 of the study. Investigators may prescribe concomitant medications or treatments deemed necessary to provide adequate supportive care, with the exception of those listed in Section 7.2 as exclusions.

All concomitant medications taken during the study must be recorded in the source documentation and in the eCRF.

8.4 Method of Assigning Patients to Treatment

Using an Interactive Voice Response System (IVRS), patients will be randomized to mipomersen or placebo in a 2:1 ratio, prior to study drug administration. Patient enrollment will be conducted such that at least 51 patients are randomized with LDL-C of at least 200 mg/dL (5.1 mmol/L) for patients with CHD, or at least 300 mg/dL (7.8 mmol/L) for patients without CHD, representing a patient population in which LDL-C apheresis is indicated in the USA. Additionally, approximately 24 patients with LDL-C of at least 190 mg/dL (4.9 mmol/L) will be randomized to provide additional exposure among a slightly broader patient population. The Sponsor's Quality Assurance (QA) & Compliance department or designee will hold a copy of the randomization schedule, generated by the Sponsor (or designee). All patients, Investigators, study staff, and the Sponsor will be blinded to the treatment assignment.

8.5 Dose Selection

The 200 mg/week dose was selected for this study because results from Phase 1 and Phase 2 clinical studies showed a satisfactory safety profile with a significant pharmacodynamic effect at this dose (see Investigator's Brochure). Due to the long terminal elimination half-life of mipomersen, the treatment duration is 26 weeks so that the efficacy endpoint can be evaluated when drug tissue levels are expected to be at > 90% of steady-state values.

The safety and tolerability of the proposed dosing regimen is also supported by nonclinical chronic toxicology studies (see Investigator's Brochure).

8.6 Blinding and Randomization

Patients, monitors, study center personnel, and the Sponsor will remain blinded to the patient's treatment assignment throughout the study. In addition, in order to ensure maintenance of the study blind, lipid data will be not be available to the patients, Investigators, study staff, or the Sponsor until the study has been unblinded, as knowledge of these data could unblind these individuals to the treatment assignment and could influence patient assessment.

8.6.1 Unblinding

In the event of an emergency where the identity of the study drug must be known by the Investigator to provide appropriate medical treatment, the Investigator will be allowed to unblind using the IVRS. However, prior to this, the Investigator should make every effort to discuss the situation with the Medical Monitor.

In the event that an unblinding occurs, the Investigator will notify the Sponsor or designee of the unblinding within 24 hours of its occurrence. The treatment assignment should only be unblinded in the case of a serious adverse event (SAE; see Section 9.2.2), and when knowledge of the treatment assignment will impact the clinical management of the patient. Every reasonable attempt should be made to complete the End-of-Study (study termination) evaluation procedures prior to unblinding as knowledge of the treatment assignment could influence patient assessment.

9 EFFICACY AND SAFETY VARIABLES

9.1 Efficacy and Safety Measurements Assessed and Study Flowchart

The study for an individual patient will consist of the following periods:

- Up to a 4-week screening period
- A 26-week treatment period during which study drug will be administered (consisting of a SC dose once a week), and
- A 24-week post-treatment evaluation period

Table 9-1 summarizes the schedule of study events at each visit for patients enrolled.

Table 9-1 Schedule of Study Events

Study Period	Screen -ing	Treatment									Post-Treatment Evaluation			
Study Week	≤ 4 weeks	Wk 1	Wk 3	Wk 5	Wk 7	Wk 9	Wk 13	Wk 17	Wk 21	Wk 26	Wk 28	Wk 32	Wk 40	Wk 50
Study Day	-28 to -1	1	15	29	43	57	85	113	141	176	190	218	274	344
Visit[h]	-1	Visit 1	Visit 2	Visit 3	Visit 4	Visit 5	Visit 6	Visit 7	Visit 8	Visit 9	PTx Visit 1	PTx Visit 2	PTx Visit 3	PTx Visit 4
Visit Window		± 2 days									± 7 days			
Written Informed Consent	X													
Medical History	X													
Demographics and Baseline Characteristics	X	X												
Inclusion / Exclusion Criteria	X													
Physical Examination[a]	X			X		X	X				X			X
Abdominal & Hip Circumference	X										X			
Liver & Spleen MRI or CT		X[b]												
Vital Signs (+ body weight and height at Screening)	X	X	X	X	X	X	X	X	X	X	X	X	X	X
Urinalysis[c]	X[g]	X[d]	X	X	X	X	X[d]	X	X	X	X[d]	X	X	X
Pregnancy Test[e]	X	X		X		X	X	X	X	X		X	X	X
ISR Evaluation		X	X	X	X	X	X	X	X	X	X			
Weekly Study Drug[f] Administration		X	X	X	X	X	X	X	X	X				

Table 9-1 Schedule of Study Events (Continued)

Study Period	Screen-ing	Treatment									Post-Treatment Evaluation			
Study Week	≤ 4 weeks	Wk 1	Wk 3	Wk 5	Wk 7	Wk 9	Wk 13	Wk 17	Wk 21	Wk 26	Wk 28	Wk 32	Wk 40	Wk 50
Study Day	-28 to -1	1	15	29	43	57	85	113	141	176	190	218	274	344
Visit[h]	-1	Visit 1	Visit 2	Visit 3	Visit 4	Visit 5	Visit 6	Visit 7	Visit 8	Visit 9	PTx Visit 1	PTx Visit 2	PTx Visit 3	PTx Visit 4
Visit Window		± 2 days									± 7 days			
12-Lead ECG	X						X				X			X
AE Assessment	X	X	X	X	X	X	X	X	X	X	X	X	X	X
Concomitant Medications/Therapies	X	X	X	X	X	X	X	X	X	X	X	X	X	X

[a] Full physical examination at Screening must include height measurement. Physical examinations, including general appearance, skin, head, ears, eyes, nose, throat (HEENT), lymph nodes, heart, lungs, abdomen, extremities/joints, neurological, and mental status will be performed at Screening and during the treatment period. Additionally, the patients will be monitored for lymphadenopathy and splenomegaly.

[b] Baseline magnetic resonance imaging (MRI) or computed tomography (CT) should be done prior to administering the first dose of the study drug. Follow-up MRI or CT will be obtained if clinically indicated (i.e., for alanine aminotransferase [ALT] elevations ≥ 3 x upper limit of normal [ULN]) during the course of the study. Please refer to the Site Reference Binder for MRI and CT procedures and settings.

[c] Sample collected prior to study drug administration during treatment period

[d] Urinalysis on these days includes total protein, microalbumin, creatinine, urine protein to creatinine ratio (P/C ratio), and β2-microglobulin

[e] For females of childbearing potential only.

[f] The first 3 doses must be supervised by a health professional, including study center personnel or home health nurse. Thereafter, patients will be given the option to self-administer study drug injections unsupervised.

[g] At Screening, if urine dipstick is positive (> 1+) for microalbuminuria, then a 24-hour sample will be obtained to assess protein excretion. If total protein > 1g/24 hr then the patient will be excluded.

ISR = injection site reaction
ECG = electrocardiogram
AE=adverse event

Table 9-1 Schedule of Study Events (Continued)

Study Period	Screen-ing	Treatment									Post-Treatment Evaluation			
Study Week	≤ 4 weeks	Wk 1	Wk 3	Wk 5	Wk 7	Wk 9	Wk 13	Wk 17	Wk 21	Wk 26	Wk 28	Wk 32	Wk 40	Wk 50
Study Day	-28 to -1	1	15	29	43	57	85	113	141	176	190	218	274	344
Visit[h]	-1	Visit 1	Visit 2	Visit 3	Visit 4	Visit 5	Visit 6	Visit |7	Visit 8	Visit 9	PTx Visit 1	PTx Visit 2	PTx Visit 3	PTx Visit 4
Visit Window		± 2 days									± 7 days			
Chemistry Panel (+ CPK)	X	X	X	X	X	X	X	X	X	X	X	X	X	X
Full Serum Lipid Panel	X	X		X		X	X	X	X	X	X	X	X	X
HbA_{1C}[k]	X						X				X			X
Hematology (Complete Blood Count + Diff.)	X	X	X	X	X	X	X	X	X	X	X	X	X	X
Coagulation (aPTT, PT)		X					X				X			
Hepatitis B & C, HIV	X													
Thyroid function (T_4, TSH)	X													
Trough PK - ISIS 301012[j]		X	X	X	X	X	X	X	X	X	X	X	X	X
ISIS 301012 Antibodies[i]		X									X			X
IgG		X					X				X			
Cardiovascular Risk Markers (hsCRP and LDL subclass concentrations)	X	X					X			X	X			
Complement (C3)		X					X				X			

Table 9-1 Schedule of Study Events (Continued)

Study Period	Screen-ing	Treatment									Post-Treatment Evaluation			
Study Week	≤ 4 weeks	Wk 1	Wk 3	Wk 5	Wk 7	Wk 9	Wk 13	Wk 17	Wk 21	Wk 26	Wk 28	Wk 32	Wk 40	Wk 50
Study Day	-28 to -1	1	15	29	43	57	85	113	141	176	190	218	274	344
Visit[h]	-1	Visit 1	Visit 2	Visit 3	Visit 4	Visit 5	Visit 6	Visit 7	Visit 8	Visit 9	PTx Visit 1	PTx Visit 2	PTx Visit 3	PTx Visit 4
Visit Window		± 2 days[c]									± 7 days			
Erythrocyte Sedimentation Rate (ESR)		X					X				X			
Inflammatory Markers[l]		X					X				X			

h All visits are conducted after an overnight fast for > 10 hours (only water permitted). Note: all labs must be performed prior to injection of study drug.

i Sample collected prior to study drug administration during treatment period

j The outpatient visits at Week 13 and Week 26 must be scheduled on a dosing day, and must occur 7 days after the previous dose. Blood samples for pharmacokinetic (PK) trough levels MUST be drawn prior to mipomersen administration at all outpatient visits during the treatment period.

k At Screening for all patients and diabetic patients only thereafter.

l In addition to the pre-specified inflammatory markers (i.e., immunoglobulin G [IgG], white blood cell [WBC] differentials, complement [C3], erythrocyte sedimentation rate [ESR], and high-sensitivity C-reactive protein [hsCRP]), a serum sample specifically designated for the measurement of inflammatory parameters will be collected pretreatment, during treatment (Week 13) and at the end of treatment (Week 28) and appropriately stored for subsequent analysis, as needed. This will allow for the measurement of other inflammatory markers should significant changes in the primary markers be noted or for further characterization of inflammation based on emerging markers.

Note: The laboratory test, hsCRP, is a marker of inflammation and, as such, may be elevated due to any condition that causes general inflammation including but not limited to recent or ongoing illness, viral, bacterial or fungal infection, tissue injury, trauma, bone fractures or exacerbation of chronic inflammatory conditions. Since this test is sensitive to these other conditions, samples for hsCRP evaluations should not be drawn from patients with acute inflammatory conditions or with chronic inflammatory conditions that have been acutely exacerbated until such conditions are clinically stable. In any event, tests should not be postponed for more than 4 weeks. Reasons for such postponing tests should be documented.

Note: All items on this page of the study schedule require blood draws (collected prior to study drug administration during the treatment period). CPK=Creatine phosphokinase, aPTT=activated partial thromboplastin time, PT=prothrombin time, T_4=thyroxine, TSH=thyroid stimulating hormone, HbA_{1c}=Glycosylated hemoglobin A, HIV=human immunodeficiency virus, LDL=low density lipoprotein.

9.1.1 Screening Assessments

The initial Screening visit will take place within a maximum of 4 weeks before the first dose of study drug is administered (on Day 1). Once written informed consent is obtained, a patient's eligibility for entry into the study will be assessed according to the inclusion and exclusion criteria.

9.1.1.1 Medical History, Physical Examinations, 12-Lead Electrocardiogram

Patients will give a full medical history, and undergo a full physical examination, including anthropometric and vital signs measurements and monitoring for lymphadenopathy and splenomegaly (Refer to Section 9.1.2.6). A 12-lead ECG tracing will be obtained.

9.1.1.2 Clinical Laboratory Evaluations and Therapeutic Lifestyle Changes

Blood and urine samples will be collected for clinical laboratory evaluations according to the schedule in Table 9-1 (see Table 9-2 for a complete list of analytes). The patient will also be counseled on the TLC approach (Appendix 14.1); this will include advice on diet that should be maintained throughout the treatment period. Lipoprotein testing information is in Appendix 14.2.

Table 9-2 List of Screening Clinical Laboratory Evaluations

<table>
<tr><th colspan="4">Screening Assessments</th></tr>
<tr><th>Clinical Chemistry</th><th>Hematology</th><th>Urinalysis</th><th>Other</th></tr>
<tr>
<td rowspan="3">Sodium
Potassium
Chloride
Bicarbonate
Total protein
Albumin
Total globulin
Calcium
Phosphorus
Glucose
Blood urea nitrogen (BUN)
Creatinine
Uric Acid
LDH
Total, Direct, and Indirect Bilirubin
Alkaline phosphatase
AST (SGOT)
ALT (SGPT)
CPK</td>
<td rowspan="3">Red blood cells
Hemoglobin
Hematocrit
MCV
Platelets
White blood cells (WBC)
WBC Differential (% and absolute)
• Neutrophils
• Eosinophils
• Basophils
• Lymphocytes
• Monocytes</td>
<td>Specific gravity
pH
Protein
Glucose
Ketones
Bilirubin
Blood
Red blood cells
WBC
Epithelial cells
Bacteria
Casts
Crystals</td>
<td><u>Thyroid Function</u>
T_4
TSH

<u>Hepatitis</u>
Hepatitis B surface antigen
Hepatitis C antibody

<u>HIV</u>
HIV antibody

<u>Pregnancy</u>
hCG

<u>Cardiovascular Risk Markers</u>
hsCRP
LDL-subclass concentrations</td>
</tr>
<tr><th>Full Lipid Panel</th><th>Metabolic</th></tr>
<tr><td>ApoB
ApoA-1
Total cholesterol
LDL-C
HDL-C
VLDL-C
TG
Lp(a)</td><td>HbA_{1c}</td></tr>
</table>

BUN=blood urea nitrogen, LDH=low density lipoprotein, AST (SGOT)=aspartate aminotransferase, ALT (SGPT)=alanine aminotransferase, CPK=creatine phosphokinase, MCV=mean corpuscular volume, WBC=white blood cells, apo=apoliprotein, LDL-C=low density lipoprotein cholesterol, HDL-C=high density lipoprotein cholesterol, VLDL-C= very high density lipoprotein cholesterol, TG=triglycerides, Lp(a)=lipoprotein (a), T_4=thyroxine, TSH=thyroid-stimulating hormone, HIV=human immunodeficiency virus, hCG=human chorionic gonadotropin, hsCRP=high-sensitivity C-reactive protein, HbA_{1c}=glycosylated hemoglobin A.

9.1.2 Treatment Period Assessments

The study for an individual patient will consist of the following periods:

- Up to a 4-week screening period
- A 26-week treatment period during which study drug will be administered (consisting of a SC dose once a week), and

- A 24-week post-treatment evaluation period

There is a visit window of ± 2 days through Week 26. In the event that a visit does not occur on the scheduled date, all subsequent visits should be calculated based on the time elapsed since Day 1, rather than from the date of the last visit. The outpatient visits at Week 13 and Week 26 must be scheduled on a dosing day, and must occur 7 days after the previous dose.

9.1.2.1 Magnetic Resonance Imaging and Computed Tomography

Baseline MRI or CT of the liver and spleen will be collected for all patients prior to administering the first dose of study drug. A CT scan may be performed in lieu of both the baseline and follow-up MRI for patients in whom an MRI is contraindicated. The images will be stored for future analysis if required. Follow-up MRI (or CT) will be obtained if clinically indicated (i.e., for ALT elevations ≥ 3 x ULN) during the course of the study. Please refer to the Site Reference Binder for MRI (or CT) procedures and settings.

9.1.2.2 Clinical Laboratory Evaluations

The following clinical laboratory tests will be assessed by a central laboratory at the time points specified in Table 9-1.

A blood sample for baseline full serum lipid panel, hematology with differential, chemistry, cardiovascular risk markers (high-sensitivity C-reactive protein [hsCRP] and LDL subclass concentrations), immunoglobulin G (IgG), ISIS 301012 antibodies, erythrocyte sedimentation rate (ESR), complement (C3), and coagulation parameters (activated partial thromboplastin time [aPTT] and prothrombin time [PT]) will be drawn prior to study drug administration according to the schedule in Table 9-1. In addition to the pre-specified inflammatory markers (i.e., IgG, white blood cell (WBC) differentials, C3, ESR, and hsCRP), a serum sample specifically designated for the measurement of inflammatory parameters will be collected pretreatment, during treatment (Week 13) and at the end of treatment (Week 28) and appropriately stored for subsequent analysis, as needed. This will allow for the measurement of other inflammatory markers should significant changes in the primary markers be noted or for further characterization of inflammation based on emerging markers. Urinalysis will be performed at each visit during the treatment period. Pregnancy tests for women of childbearing potential will be performed monthly during study participation. Table 9-3 details clinical laboratory evaluations for the treatment period; Table 9-1 lists the visits at which samples will be drawn.

A central laboratory will provide collection supplies, arrange collection, and perform analysis of clinical laboratory evaluations indicated in Table 9-2 and Table 9-3. Using the central laboratory for unscheduled clinical laboratory evaluations and redraws is

recommended. Procedures for the handling and shipment of all central laboratory samples will be included in the laboratory manual. Specimens will be appropriately processed by the central laboratory facility and laboratory reports will be made available to the Investigator in a timely manner to ensure appropriate clinical review.

The Investigator is responsible for reviewing and signing all laboratory reports. The clinical significance of each value outside of reference range will be assessed and documented as either not clinically significant (NCS) or clinically significant (CS). All CS values require a comment and the out-of-range value or the overlying diagnosis (or value if the diagnosis is unknown) must be captured as an AE.

Clinical significance is defined as any finding that results in an alteration in medical care (for definition of clinical significance, please refer to Section 9.2.3.5). The Investigator will continue to monitor the patient until the parameter returns to its baseline status or until agreement is reached between the Investigator and Sponsor.

Clinical laboratory reports will serve as source documents.

Table 9-3 List of Treatment Period Clinical Laboratory Evaluations

Clinical Safety Assessments (Minimum Requirements)		Efficacy Assessments	Other Assessments
**Clinical Chemistry**	**Urinalysis**	**Full Lipid Panel**	**Metabolic**
Sodium<br>Potassium<br>Chloride<br>Bicarbonate<br>Total protein<br>Albumin<br>Total globulin<br>Calcium<br>Phosphorus<br>Glucose<br>BUN<br>Creatinine<br>Uric Acid<br>LDH<br>Total, Direct, and Indirect Bilirubin<br>Alkaline phosphatase<br>AST (SGOT)<br>ALT (SGPT)<br>CPK	Specific gravity<br>pH<br>Protein<br>Glucose<br>Ketones<br>Bilirubin<br>Blood<br>Red blood cells<br>WBC<br>Epithelial cells<br>Bacteria<br>Casts<br>Crystals<br>Creatinine*<br>Total Protein*<br>Protein to creatinine (P/C) Ratio*<br>Microalbumin*<br>β2-microglobulin*	ApoB<br>ApoA-1<br>Total cholesterol<br>LDL-C<br>HDL-C<br>VLDL-C<br>TG<br>Lp(a)	HbA_{1c}<br>**Inflammatory Markers†**<br>To be determined
**Coagulation**	**Hematology**	**Cardiovascular Risk Markers**	**Immune Function**
aPTT<br>PT<br>INR<br>**Complement**<br>C3	Red blood cells<br>Hemoglobin<br>Hematocrit<br>MCV<br>Platelets<br>White blood cells<br>WBC Differential (% and absolute)<br>• Neutrophils<br>• Eosinophils<br>• Basophils<br>• Lymphocytes<br>• Monocytes	hsCRP<br>LDL subclass concentrations	IgG<br>ISIS 301012 antibodies<br>ESR<br>**Pharmacokinetic**<br>Trough ISIS 301012 level<br>**Pregnancy**<br>hCG[a]

*At select outpatient visits (Weeks 1, 13, and 28)
[a] For women of childbearing potential; see Table 9-1 for schedule of visits at which a pregnancy test is performed.
†In addition to the pre-specified inflammatory markers (i.e., IgG, WBC Differential, C3, ESR, and hsCRP), a serum sample specifically designated for the measurement of inflammatory parameters will be collected pretreatment, during treatment (Week 13) and at the end of treatment (Week 28) and appropriately stored for subsequent analysis, as needed.

9.1.2.3 Demographics, Vital Signs, Body Weight, and Anthropometric Measurements

Demographic data, height, and weight will be recorded at the Screening visit.

Vital signs (BP, heart rate, respiratory rate, and temperature) will be measured at the time points specified in Table 9-1. Semi-supine systolic and diastolic BP should always be measured on the same arm (preferentially on the left arm) with the patient being in a semi-supine position for at least 5 minutes. Any clinically significant abnormal findings will be recorded on the AE page of the eCRF.

If a CS deterioration is noted, the changes will be documented as AEs on the eCRF. Clinical significance is defined as any finding that results in an alteration in medical care. The Investigator will continue to monitor the patient until the parameter returns to its baseline status or until agreement is reached between the Investigator and Sponsor.

Height and body weight should be measured without shoes. Body weight should be measured with the patient fully dressed in everyday clothes, but overcoats should be removed before weighing.

Waist circumference should be measured with a stretchless tape and patient in a standing position after normal expiration, midway between the caudal part of the lateral costal arch and the iliac crest. Hip circumference should be measured at the symphysis trochanter level. Calculations to be performed are BMI and waist-to-hip ratio (WHR).

9.1.2.4 Pharmacokinetic Trough Levels

At each outpatient visit to the study center, including Day 1, a blood sample will be drawn prior to study drug administration for plasma PK trough levels. Blood samples for PK trough levels MUST be drawn prior to study drug administration at all outpatient visits during the treatment period.

9.1.2.5 Electrocardiogram

The ECG will be recorded after the patient has been resting in a supine position for at least 5 minutes. Six limb leads, as specified by Einthoven (I, II and III) and Goldberger (aVR, aVL and aVF) and 6 precordial leads (V1-V6) according to Wilson will be used. Printouts of the recordings will be done with 25 mm/s time resolution at an amplification of 10 mm/mV. Electrocardiograms will be performed at Screening, and at Weeks 13, 28, and 50.

The ECG tracing should contain at least 4 QRS complexes (in order to obtain 3 RR intervals). Each ECG tracing should include the following information: identification of each lead, study number, study day number, patient number, paper speed, voltage

calibration, and date and time of recording. After identification of each lead, the Investigator should evaluate, sign, and date the printout. The Investigator should state whether the ECG is normal or abnormal; if abnormal, whether the ECG tracing is clinically significant.

9.1.2.6 Physical Examination

A complete physical examination will be conducted at the time points specified in Table 9-1. The examination will include an assessment of the patient general appearance; skin; head, ears, eyes, nose throat (HEENT); examinations of lymph nodes; heart; lungs; abdomen; extremities/joints; and neurological and mental status. Additionally, the patients will be specifically monitored for lymphadenopathy and splenomegaly and any findings will be recorded. Whenever possible, the same physician should perform the examination at each study visit.

If a clinically significant deterioration is noted, the change will be documented as an AE on the eCRF. Clinical significance is defined as any finding that results in an alteration in medical care. The Investigator will continue to monitor the patient until the parameter returns to its baseline status or until agreement is reached between the Investigator and Sponsor.

9.1.2.7 Concomitant Medications

Concomitant medications are to be assessed at all visits per Table 9-1. Reasonable efforts will be made to determine all treatments (pharmacological and non-pharmacological) received by the patient. A therapy is considered concomitant if it is taken at any time after the Screening visit up to and including the day of the final study evaluation. Data on pharmacological and non-pharmacological treatments will be captured on separate eCRF pages, but both to include: name, dose, route, regimen, start date, stop date, and indication. At each study visit, the patient will be asked about any additional treatments or any changes in regimen or dosages since the last visit. Indications for any new medications or therapies during the study period will be recorded as an AE.

9.1.2.8 Injection Site Reaction Assessment

At each outpatient visit during the treatment period, an ISR assessment will be made for the current and previous injection sites. ISR assessments are recorded starting after the first injection is given.

9.1.3 Post-Treatment Evaluation Period Assessments

There is a visit window of ± 7 days throughout the post-treatment evaluation period. During this period, MRI or CT (if clinically indicated), clinical laboratory evaluations, vital signs, body weight, anthropometric measurements, plasma PK trough levels, ECG, and a full

physical examination will be performed according to the schedule in Table 9-1, and as described in Section 9.1.2. Adverse events and changes in concomitant medication use will continue to be reported at each visit.

9.2 Adverse Events

9.2.1 Definition of Adverse Events

An AE is defined as any undesirable physical, psychological or behavioral effect experienced by a patient during their participation in an investigational study, in conjunction with the use of the drug, whether or not product-related. This includes any untoward signs or symptoms experienced by the patient from the time of signing the informed consent until completion of the study.

Adverse events may include, but are not limited to:

- Subjective or objective symptoms spontaneously offered by the patient and/or observed by the Investigator or medical staff
- Laboratory abnormalities of clinical significance

Disease signs, and symptoms and/or laboratory abnormalities already existing prior to the use of the product are not considered AEs after treatment unless they recur after the patient has recovered from the pre-existing condition or, in the opinion of the Investigator, they represent a clinically significant exacerbation in intensity or frequency.

9.2.2 Definition of Serious Adverse Events

A SAE is any AE that results in any of the following outcomes:

- Death
- Life-threatening experience
- Required or prolonged inpatient hospitalization
- Persistent or significant disability/incapacity
- Congenital Anomaly
- Important medical events that may jeopardize the patient or subject and may require medical or surgical intervention to prevent one of the outcomes listed above.

Death: The patient died following an AE.

Life-threatening experience: Any AE that places the patient, in the view of the reporter, at immediate risk of death from the AE as it occurred, i.e., does not include an AE that had it occurred in a more severe form, might have caused death.

Requires inpatient hospitalization, or prolongation of existing hospitalization: The AE resulted in an initial inpatient hospitalization or prolonged an existing hospitalization of the patient. For this protocol, hospitalization is defined as an admission to a medical facility of greater than 24 hours.

Persistent or significant disability/incapacity: An AE that results in a substantial disruption of a person's ability to conduct normal life functions.

Congenital anomaly: The exposure of the patient to the drug during pregnancy that is judged to have resulted in the congenital anomaly/birth defect.

Important medical events: AEs that may not result in death, be life-threatening, or require hospitalization may be considered SAEs when, based upon appropriate medical judgment, they may jeopardize the patient and may require medical or surgical intervention to prevent one of the outcomes listed above.

Planned hospital admissions or surgical procedures for an illness or disease that existed before the administration of the study treatment or before the patient was enrolled in the study, will not be captured as SAEs unless they occur at a time other than the planned date.

Serious adverse events will be followed until resolution or until such time agreed upon by the Sponsor or designee and the Investigator. Adverse events and concomitant medications will be collected from the time of informed consent through study completion (Week 50 or Early Termination).

9.2.3 Evaluation of Adverse Events and Serious Adverse Events

9.2.3.1 Severity of Adverse Events

The Investigator will assess the severity of the (S)AE using the following categories: Mild, Moderate, and Severe. This assessment is subjective, and the Investigator should use medical judgment to compare the reported (S)AE to similar events observed in clinical practice.

Guidelines for severity assessment are listed below:

Mild: Symptom(s) barely noticeable to the patient or do(es) not make the patient uncomfortable. The (S)AE does not influence performance or functioning. Prescription drugs are not ordinarily needed for relief of symptom(s).

Moderate: Symptom(s) of a sufficient severity to make the patient uncomfortable. Performance of daily activities is influenced. Treatment of symptom(s) may be needed.

Severe: Symptom(s) of a sufficient severity to cause the patient severe discomfort. Severity may cause cessation of treatment with the study drug. Treatment for symptom(s) may be given.

Severity is not equivalent to seriousness.

9.2.3.2 Action Taken/Medication or Therapy Given

The Investigator will be required to provide any action taken with regards to an (S)AE. The action taken is determined as follows:

No change: No change in product dosing was implemented. This includes cases in which the product was discontinued prior to the occurrence of the (S)AE.

Temporarily Discontinued: The drug was discontinued temporarily and will probably be restarted at a later time point. The Investigator will provide a stop and restart date.

Permanently Discontinued: The drug was discontinued permanently. The Investigator will provide a stop date.

9.2.3.3 Outcome of Adverse Events

The Investigator will be required to provide information regarding the patient outcome of an (S)AE to the study drug.

The patient outcome is determined as follows:

Recovered: The (S)AE has resolved and the patient does not have any residual symptoms.

Recovered With Sequelae: The (S)AE has resolved, however the patient has some residual symptoms. The Investigator will provide information to specify the sequelae.

Not Yet Recovered: The patient has not yet recovered from the (S)AE. These cases will be followed-up until the report is classified in one of the other categories or follow-up is no longer required as determined by the Investigator. The Investigator must follow patients with (S)AEs until their condition resolves or stabilizes. Certain (S)AEs may not resolve. Serious adverse events will be followed until resolution or until such time agreed upon by the Sponsor or designee and the Investigator.

Ongoing at the time of death: The (S)AE was ongoing at the time of death of the patient and was not directly related to the patient's death.

Fatal: The patient died from the (S)AE. The patient's date of death is the date of the (S)AE cessation.

9.2.3.4 Relationship to Study Treatment

The Investigator will assess the relationship between the (S)AE and the study treatment according to the following definitions:

Unrelated: There was no relationship of the (S)AE to the use of the study drug. This may include, but is not limited, to the (S)AE being an expected outcome of a previously existing or concurrent disease, concomitant medication or procedure the patient experienced during their treatment period. For reporting purposes, Unrelated will be considered Not Product Related.

Remote/Unlikely: (Serious) Adverse experiences which are judged probably not related to the drug. For reporting purposes, Remote/Unlikely will be considered Not Product Related.

Possible: There was no clear relationship of the (S)AE to the use of the study drug; however, one cannot definitively conclude that there was no relationship. For reporting purposes, Possible will be considered Product Related.

Probable: While a clear relationship to the drug cannot be established, the experience is associated with an expected (S)AE or there is no other medical condition or intervention which would explain the occurrence of such an experience. For reporting purposes, Probable will be considered Product Related.

Definite: The relationship of the use of the drug to the experience is considered definitively established. For reporting purposes, Definite will be considered Product Related.

9.2.3.5 Clinical Laboratory Tests

The Investigator will assess all laboratory results outside the normal range as CS or NCS. Clinical significance is defined as any variation that has medical relevance resulting in an alteration in medical care. Whenever possible, the underlying diagnosis should be listed in lieu of the abnormal laboratory values. Laboratory abnormalities deemed NCS by the Investigator should not be reported as (S)AEs. Similarly, laboratory abnormalities reported as (S)AEs by the Investigator should not be deemed NCS on the laboratory sheet.

9.2.3.6 Dosing Errors

Dosing details should be captured on the Dosing eCRF. In addition, if the patient takes a dose of study drug that significantly exceeds protocol specifications, the “Dose/dosing rate exceeded protocol specifications” box should also be checked. If the patient is symptomatic, the “Associated with AE(s)? - Yes” box should be checked. If the event does not meet serious criteria, the symptom(s) should be documented on the non-serious AE eCRF and the corresponding AE numbers listed on the Dosing eCRF; if the event meets serious criteria, an SAE form should be completed and the event reported as a SAE instead. The site should

also document the SAE on the AE eCRF and enter the corresponding AE numbers on the Dosing eCRF. Do not capture the event on the non-serious AE eCRF or SAE form if the patient is not symptomatic.

9.2.4 Adverse Experience and Serious Adverse Experience Reporting

9.2.4.1 Adverse Experience Reporting

Adverse events, including SAEs, will be reported from the time the patient signs the informed consent form until study completion. All AEs, including SAEs, experienced by the patient will be noted on the eCRF. A full description including the nature, date, time of onset and resolution, determination of seriousness, intensity, corrective treatment, outcome, and relationship to study drug will be recorded. Changes in vital signs, laboratory results and other safety assessments from Baseline will be recorded if they are deemed clinically significant. When possible, a diagnosis should be recorded as an AE, rather than the symptoms related to that diagnosis. A medical or surgical procedure is not an AE; rather the condition leading to the procedure should be recorded as the AE. All SAEs experienced by the patient will be recorded on a SAE report form and reported to Genzyme.

The Investigator must follow patients with AEs until their condition resolves or stabilizes. Certain AEs may not resolve. Serious adverse events will be followed until resolution or until such time agreed upon by Genzyme or its designee and the Investigator. Adverse events and concomitant medications will be collected from the time of informed consent through study completion (Week 50 or Early Termination).

9.2.4.2 Serious Adverse Event Reporting

9.2.4.2.1 Reporting to Genzyme

All SAEs must be reported by to the Genzyme Pharmacovigilance Department within 24 hours of the Investigator's first knowledge of the event (Table 9-4). SAE communication is directed to:

Table 9-4 Contact List for Serious Adverse Event Reporting

United States and Rest of World (excluding Europe)	Europe
**Genzyme US Pharmacovigilance**<br><br>FAX: 1 617 761 8506<br><br>Email:<br>pharmacovigilancesafety@genzyme.com<br><br>Phone:<br>Night (6 pm EST- 8 am EST), Weekends and Holidays<br>1 800 745 4447 (US toll free number)<br>1 617 768 9000 (outside US)	**Genzyme Europe BV Pharmacovigilance**<br><br>FAX: +31 (0) 35 694 87 56<br><br>Email:<br>EUPharmacovigilance@genzyme.com<br><br>Phone:<br>7days/24hours<br>+31 (0) 35 699 12 99

For all SAEs, a SAE Report Form that includes a detailed written description, copies of relevant anonymized patient records, and other documents will be sent to Genzyme Pharmacovigilance within 24 hours. Follow-up SAE reports will be forwarded as soon as the information is available.

Additionally, the Institutional Review Board (IRB)/Independent Ethics Committee (IEC) must also be notified in writing of any SAEs according to the applicable regulations. Genzyme will report SAEs to appropriate regulatory agencies, as required.

After the study is completed, if the Investigator or study staff becomes aware of a related SAE that occurs within 30 days of study completion, this event and any known details must be reported to Genzyme Pharmacovigilance.

9.2.5 Pregnancy Reporting

If a patient becomes pregnant at anytime during the conduct of this study, she must not receive any additional study drug and must be discontinued from the study.

The patient must be followed until the outcome of the pregnancy is known (i.e., delivery, elective termination, or spontaneous abortion). If the pregnancy results in birth of a child, additional follow-up information may be requested.

In case of paternal exposure anytime after receiving study treatment, the patient may continue the study. However, the patient's pregnant partner must be followed until the outcome of the pregnancy is known (i.e., delivery, elective termination, or spontaneous

abortion). If the pregnancy results in the birth of a child, additional follow-up information may be requested.

The Investigator must notify Genzyme Pharmacovigilance within 24 hours of first learning of the occurrence of pregnancy, using the appropriate Pregnancy Notification Form(s) (Appendix 14.4, Forms A and/or B), providing as much information as possible. For reporting initial notification of a female study patient please provide Pregnancy Notification Form A. If study patient is male, please complete and provide both Pregnancy Notification Forms A and B. The Investigator must notify Genzyme about reported complications within 24 hours using the same procedure. Outcome of pregnancy, once known by the Investigator, must also be reported to Genzyme within 24 hours using the Pregnancy Outcome Form C and faxing it to Genzyme. For reporting additional information about the pregnancy use Pregnancy Notification Form(s) A and/or B and indicate "follow-up" on the form.

Communications regarding pregnancies should be directed to the contacts listed in Table 9-4.

Please note that pregnancy in and of itself is neither an AE nor a SAE. Pregnancy should not be entered into the eCRF as an AE unless the Investigator suspects an interaction between the study treatment and contraceptive method used. For female patients, pregnancy will be documented as the reason for study discontinuation.

9.2.6 Stopping Rules and Safety Monitoring

In addition to the standard monitoring of clinical safety parameters, the following guidelines are provided for the monitoring of selected parameters chosen based on preclinical and clinical observations (see Section 8, "Guidance for the Investigator," in the Investigator's Brochure).

9.2.6.1 Confirmation Guidance

At any time during the study (treatment or post-treatment periods), initial clinical laboratory results meeting the stopping rules or safety monitoring criteria presented below must be confirmed by performing measurements (ideally in the same laboratory that performed the initial measurement) on new specimens. All new specimen collections should take place as soon as possible (ideally within 3 days of the initial collection) and, in any event, no longer than 5 days from the collection that yielded the initial observation. If the initial laboratory result is observed during the treatment period, the results from the retest must be available prior to the next scheduled dose of study drug (mipomersen or placebo).

If a new specimen is not, or cannot be, collected ≤ 7 days of the specimen collection that produced the initial laboratory observation and/or the result from the test of the new

specimen is not available prior to the scheduled administration of the next dose, the initial laboratory result is presumed confirmed.

9.2.6.2 Stopping Rule Guidance

If any of the stopping criteria described below are met and are confirmed, the patient will be permanently discontinued from further treatment with study drug (mipomersen or placebo), evaluated fully as outlined below and in consultation with the Medical Monitor, and will be entered into the post-treatment evaluation portion of the study. In general, patients who do not meet the stopping rules based upon retest may continue dosing. However, the Investigator and the Medical Monitor should confer as to whether additional close monitoring of the patient is appropriate.

9.2.6.3 Liver Chemistry Rules

The following rules are adapted from the draft guidance for industry, “Drug-Induced Liver Injury: Premarketing Clinical Evaluation,” issued by the U.S. Department of Health and Human Services, Food and Drug Administration, October 2007.

9.2.6.3.1 Stopping Rules for Liver Chemistry Elevations

In the event of confirmed or presumed confirmed laboratory results meeting the following criteria, and the event is without an alternative explanation (e.g., concomitant therapy with anticoagulants), dosing of a patient with study drug (mipomersen or placebo) will be stopped permanently:

- ALT or aspartate aminotransferase (AST) ≥ 8 x ULN
- ALT or AST ≥ 5 x ULN at 2 consecutive weekly measurements (not less than 7 days) at least 1 of which is confirmed by retest. (Weekly measurement of liver chemistry tests will be instituted following the first ALT or AST ≥ 5 x ULN finding. Treatment with study drug [mipomersen or placebo] will continue weekly until the second consecutive weekly ALT or AST measurement is confirmed or presumed confirmed to be ≥ 5 x ULN).
- ALT or AST ≥ 3 x ULN and total bilirubin > 1.5 x ULN or international normalized ratio (INR) > 1.5

In the event of confirmed or presumed confirmed laboratory results for either of the following criteria, continued dosing of a patient with study drug (mipomersen or placebo) will be discussed with the Medical Monitor:

- ALT or AST ≥ 3 x ULN with the appearance or worsening of symptoms felt by the Investigator to be potentially related to hepatic inflammation, such as fatigue, nausea, vomiting, right upper quadrant pain or tenderness, fever, rash or eosinophilia

9.2.6.3.2 Safety Monitoring Rules for Liver Chemistry Tests

In the event of an ALT or AST measurement that is ≥ 3 x ULN at any time during the study (treatment or post-treatment period), the initial measurement(s) should be confirmed as described above. Similarly, confirmatory measurements should also be performed if ALT or AST levels increase to ≥ 5 x ULN and 8 x ULN (following the initial meeting of each of those criteria).

Frequency of Repeat Measurements: Patients with confirmed or presumed confirmed ALT or AST levels ≥ 3 x ULN should have their liver chemistry tests (ALT, AST, alkaline phosphatase, and total bilirubin at a minimum) retested at least once weekly until levels stabilize or begin to recover. The performance of additional laboratory tests should be discussed with the Medical Monitor. Thereafter, liver chemistry tests for these patients should be performed at least every 2 weeks until ALT and AST are both < 3 x ULN. Subsequently, liver chemistry tests for these patients should be performed at least once a month until their ALT and AST levels become ≤ 1.2 x ULN (or ≤ 1.2 x Screening value for patients who enter the study with ALT and/or AST > ULN).

Further Investigation into Liver Chemistry Elevations: For patients with confirmed or presumed confirmed ALT or AST levels ≥ 3 x ULN, the following evaluations should be performed:

- A detailed history of symptoms and prior and concurrent diseases.
- A history for concomitant drug use (including nonprescription medications, herbal and dietary supplement preparations), alcohol use, recreational drug use, and special diets.
- A history for exposure to environmental chemical agents and travel.
- Serology for viral hepatitis (hepatitis A virus [HAV] immunoglobulin M [IgM], hepatitis B surface antigen [HBsAg], hepatitis C virus [HCV] antibody, cytomegalovirus (CMV) IgM, and Epstein-Barr Virus [EBV] antibody panel)
- Serum albumin, PT or INR, and partial thromboplastin time (PTT)
- Serology for autoimmune hepatitis (e.g., antinuclear antibody [ANA]).
- Liver MRI (chemical shift imaging) or CT

Additional liver evaluations, including gastroenterology/hepatology consults, may be performed at the discretion of the Investigator, in consultation with the Medical Monitor. Repetition of the above evaluations should be considered if a patient's ALT and/or AST levels reach ≥ 5 x ULN.

9.2.6.4 Safety Monitoring Rules for Renal Function Test Results

In the event of confirmed or presumed confirmed laboratory results for either of the following criteria, continued dosing of a patient with study drug (mipomersen or placebo) will be discussed with the Medical Monitor:

- Serum creatinine increase ≥ 0.2 or 0.3 mg/dL (≥ 17.7 or 26.5 µmol/L) above Baseline, for women and men, respectively
- Proteinuria, dipstick ≥ 2 + (confirmed by dipstick re-measurement and then further confirmed by a quantitative total urine protein measurement of > 1.0 g/24 hr)

The follow-up schedule for any events meeting either of these criteria will be determined by the Investigator in consultation with the Medical Monitor.

9.2.6.5 Safety Monitoring Rule for Platelet Count Results

In the event of a confirmed or presumed confirmed platelet count less than 75,000/mm^3, continued dosing of a patient with study drug (mipomersen or placebo) will be discussed with the Medical Monitor. The follow-up schedule for any events meeting this criterion will be determined by the Investigator in consultation with the Medical Monitor.

9.2.6.6 Safety Monitoring Rules for Coagulation Parameters

In the event that a patient has a confirmed or presumed confirmed finding for either of the following 2 criteria and the event is without an alternative explanation (e.g., concomitant therapy with anticoagulants), the Investigator should discuss the follow-up of the event with the Medical Monitor.

- aPTT > 75 seconds
- PT > 20 seconds or INR > 1.5

9.2.6.7 Safety Monitoring Rule for Constitutional Symptoms

Patients will be instructed to report any signs or symptoms of fever or flu-like symptoms that may arise within the first 24 hours after an injection is given and the Investigator should closely evaluate all potential causes of the fever, including concomitant illness.

9.2.6.8 Restriction on the Lifestyle of Patients

Patients will be counseled to follow the TLC approach (see Appendix 14.1), or similar approach depending on local guidelines, throughout the treatment period.

Patients will be required to fast overnight for > 10 hours (only water permitted) before a blood sample is taken for a full serum lipid panel on all visit days.

Patients should be willing to limit alcohol consumption for the entire duration of the study: male patients to a maximum of 2 drinks (20 g) per day, and 8 drinks (80 g) per week; female patients to a maximum of 1 drink (10 g) per day, and 4 drinks (40 g) per week.

Male patients and female patients of childbearing potential must continue to use appropriate contraception with their partners, or refrain from sexual activity, from the time of Screening and throughout study participation until 24 weeks after the last dose of mipomersen. Acceptable methods of contraception are condoms with contraceptive foam, oral contraceptives that have been prospectively discussed with the Medical Monitor, implantable or injectable contraceptives, contraceptive patch, intrauterine device, diaphragm with spermicidal gel, or sexual partner who is surgically sterilized or postmenopausal.

9.2.7 Summary of Risks

Please refer to the Investigator's Brochure for information regarding the risks and benefits of mipomersen.

10 DATA COLLECTION, QUALITY ASSURANCE, AND MANAGEMENT

10.1 Recording of Data

Original documents, data, and records being: hospital records, clinical and office charts, laboratory notes/reports, memoranda, patients' evaluation checklists, pharmacy dispensing records, recorded data from automated instruments, copies of transcriptions certified after verification as being accurate copies, source document worksheets (i.e., for study visits and IVRS), and X-rays are considered source documents. Medical histories and narrative statements relating to the patient's progress (i.e., source documents) will be maintained during the trial and for a period of 15 years after completion of the study. The Investigator must provide direct access to the source documents to the Sponsor or designee.

All data captured for this study are to be recorded in the patient's notes first and then entered in the eCRF.

Clinical data will be entered using electronic data capture (EDC) technology from a reputable vendor. All data captured electronically will be provided on a CD-ROM at the end of the study in PDF format.

All required data will be recorded in the eCRF. All missing data will be explained. Any changes made to the data after initial entry will be captured in an electronic audit trail.

10.2 Data Quality Assurance

The eCRFs will be reviewed manually at the study site for completeness by a Clinical Monitor from the Sponsor or designee, and returned to the Sponsor or designee, for data

management and analysis. If necessary, the study site will be contacted for corrections and/or clarifications. All data will be entered into a study database for analysis and reporting. Any data captured electronically will be electronically transferred to the database. Upon completion of data entry, the database will receive a QA check to ensure acceptable accuracy and completeness.

10.3 Data Management

The Sponsor or designee will be responsible for:

- Database creation and validation
- eCRF review and data validation

Prior to finalizing and locking the database, all decisions concerning the inclusion or exclusion of data from the analysis for each patient will be determined by appropriate clinical and statistical personnel. Any and all exclusions related to either safety or efficacy will be documented in patient listings.

11 STATISTICAL METHODS AND PLANNED ANALYSES

The Sponsor or designee will be responsible for the production of the following items:

1. Data listings and summary tables
2. Statistical analysis
3. Combined clinical and statistical study report

All eCRF data, as well as any outcomes derived from the data, will be summarized in detailed data listings. Patient data listings will be presented for all patients enrolled into the study.

Descriptive summary statistics including n, mean, median, standard deviation, interquartile range (25th percentile, 75th percentile), and range (minimum, maximum) for continuous variables, and counts and percentages for categorical variables will be used to summarize most data. Where appropriate, p-values will be reported. All statistical tests will be conducted using 2-sided tests with 5% Type I error rates unless otherwise stated.

Prior to locking the database and unblinding the study, all decisions concerning exclusion of patients from analysis sets will be made by appropriate clinical and statistical personnel. The Safety Set will include all randomized patients who received at least 1 injection. The Full Analysis Set, which represents the practically-feasible intent-to-treat (ITT) population as delineated in ICH Guideline E9, will include the subset of the Safety Set with at least 1 post-Baseline LDL-C measure. The Per-Protocol Set will include the subset of the Full Analysis Set with no significant protocol deviations that would be expected to effect efficacy assessments. All efficacy parameters will be assessed on the Per-Protocol Set and Full Analysis Set (primary analysis population). In addition, the primary efficacy analysis will be performed on the subset of patients with LDL-C of at least 200 mg/dL (5.1 mmol/L), for patients with CHD, or at least 300 mg/dL (7.8 mmol/L), for patients without CHD, representing a patient population in which LDL-C apheresis is indicated in the USA. All safety assessments will be performed on the Safety Set.

11.1 Power and Determination of Sample Size

Based upon prior clinical trial experience with mipomersen, it is estimated that the standard deviation of the percent change in LDL-C is approximately 22%. With 45 randomized patients (15 in the control group and 30 patients in the mipomersen-treated group), there would be 80% power to detect a 20 percentage point difference between the 2 groups. Enrollment will be conducted such that at least 51 patients are randomized (to allow for potential exclusions from an analysis set) with LDL-C of at least 200 mg/dL (5.1 mmol/L), for patients with CHD, or at least 300 mg/dL (7.8 mmol/L), for patients without CHD,

representing a patient population in which LDL-C apheresis is indicated in the USA. Additionally, approximately 24 patients with LDL-C of at least 190 mg/dL (4.9 mmol/L) will be randomized to provide additional exposure among a slightly broader patient population. Therefore, approximately 75 patients will be randomized.

11.2 Demographics and Baseline Characteristics

Demographic and baseline characteristics will be summarized using descriptive statistics by treatment group.

11.3 Patient Accountability

Patient randomization will be summarized by study site and treatment group. The patient disposition will be summarized.

All patients enrolled will be included in a summary of patient accountability for this study. The frequency and percentages of patients enrolled, presenting at each visit, discontinuing before study completion (including reason for discontinuation), and completing the study will be presented.

11.4 Study Treatment Usage and Compliance

Treatment duration and amount of study drug received will be summarized by treatment group.

11.5 Efficacy

11.5.1 Primary Efficacy Endpoint

The primary efficacy endpoint is the percent change in LDL-C from Baseline to Week 28/ET (ET represents the observation closest to 2 weeks after last dose among patients who early terminate study medication dosing).

11.5.2 Secondary Efficacy Endpoints

Secondary efficacy endpoints include the percent change in apoB, total cholesterol, non-HDL-C, TG, HDL-C, and Lp(a) from Baseline to Week 28/ET.

11.5.3 Efficacy Analysis

Percent change from Baseline to Week 28/ET in LDL-C, apo B, total cholesterol, HDL-C, non-HDL-C, TG, and Lp(a) will be compared between treatment groups using a 2-sample t-test (if data departs substantially from normality; a Wilcoxon rank sum test will be used). The analysis will take place after all patients have completed Week 28 and this database has been locked.

The Baseline is the value on Day 1, prior to the first dose of study drug. For patients without a Day 1 value (e.g., missing or inadequate sample), the last observation prior to the first dose of study drug will be used as Baseline.

The incremental effects of mipomersen on VLDL-C, apo A-1, and lipoprotein subclasses may also be explored.

11.6 Safety

11.6.1 Safety Endpoints

Safety will be determined using the incidence of treatment-emergent AEs, clinical laboratory evaluations, vital signs, ECGs, and physical examination findings. AEs will be categorized using MedDRA. Plasma concentrations of mipomersen will be evaluated.

11.6.2 Safety Analysis

The safety analyses will be performed on the Safety Set defined as all patients who receive at least 1 injection. Treatment duration and amount of study drug received will be summarized by treatment group, as will concomitant medications and treatments.

Treatment-emergent AEs will be summarized by body system, preferred term, severity, and relationship to the study procedures/treatments. If a patient has more than 1 occurrence of the same AE, he/she will be counted only once within that preferred term in the summary tables. The most severe occurrence of an AE, as well as the most extreme relationship of the AE to the study procedures or treatment, will be indicated in cases of multiple occurrences of the same AE. All AEs will be presented in a listing. Additionally listings of SAEs and AEs leading to discontinuation will be generated.

Clinical laboratory evaluations, vital signs, ECGs, and physical examination findings will be tabulated by treatment group. In addition, the number of patients who experience abnormalities in clinical laboratory evaluations will be summarized by treatment group.

Trough plasma concentrations of mipomersen (C_{min}) will be summarized over time by individual patient and by treatment group. Elimination half-life of mipomersen will be estimated for patients who enter the post-treatment follow-up period. Exploratory analyses will include but may not be limited to comparisons of trough plasma concentration of mipomersen over time within individual patients and within each treatment group.

11.7 Statistical Analysis Plan

A formal statistical analysis plan will be developed and finalized prior to database lock for the primary efficacy endpoint after all patients have completed Week 28.

12 SPECIAL REQUIREMENTS AND PROCEDURES

12.1 Institutional and Ethical Review

This protocol was designed and will be conducted, recorded, and reported in compliance with the principles of GCP guidelines as well as in accordance with all national, state and local laws of the appropriate regulatory authorities. These guidelines are stated in "Guidance for Good Clinical Practice," ICH Technical Requirements for Registration of Pharmaceuticals for Human Use.

A copy of the protocol, proposed informed consent form, other written patient information, and any proposed advertising material must be submitted to the IRB/IEC for written approval. A copy of the written approval of the protocol and informed consent form must be received by the Sponsor or designee before recruitment of patients into the study and shipment of study drug.

The Investigator must submit and, where necessary, obtain approval from the IRB/IEC for all subsequent protocol amendments and changes to the informed consent document. The Investigator should notify the IRB/IEC of deviations from the protocol or SAEs occurring at the study center and other AE reports received from the Sponsor or designee in accordance with local procedures.

The Investigator will be responsible for obtaining annual IRB/IEC approval/renewal throughout the duration of the study. Copies of the Investigator's reports and the IRB's/IEC's continuance of approval must be sent to the Sponsor or designee.

12.2 Changes to the Conduct of the Study or Protocol

No change in the study procedures shall be effected without the mutual agreement of the Investigator and Sponsor. All changes must be documented by signed protocol amendments. If changes to the design of the study are made, the amendment must be submitted to and approved by the IRB/IEC, signed by the Investigator, and returned to the Sponsor for submission to the appropriate regulatory authorities (e.g., US Food and Drug Administration [FDA], European national regulatory agencies, etc.).

12.3 Investigator's Responsibilities

12.3.1 Patient Informed Consent

The written informed consent document should be prepared in the language(s) of the potential patient population, based on an English version provided by the Sponsor or designee.

Before a patient's participation in the trial, the Investigator is responsible for obtaining written informed consent from the patient after adequate explanation of the aims, methods,

anticipated benefits, and potential hazards of the study and before any protocol-specific screening procedures or any study drugs are administered.

The acquisition of informed consent and the patient's agreement or refusal of his/her notification of the primary care physician should be documented in the patient's medical records, and the informed consent form should be signed and personally dated by the patient and by the person who conducted the informed consent discussion (not necessarily an Investigator). The original signed informed consent form should be retained in accordance with institutional policy, and a copy of the signed consent form should be provided to the patient or legally acceptable representative.

12.3.2 Electronic Case Report Forms

All data will be obtained using EDC. Refer to Section 10.1 for details regarding recording of data.

Copies of pertinent records in connection with the study, including patient charts, laboratory data, etc., will be made available to Genzyme on request with due precaution towards protecting the privacy of the patient.

12.3.3 Record Retention

Essential documents should be retained until at least 2 years after the last approval of a marketing application in an ICH region and until there are no pending or contemplated marketing applications in an ICH region or at least 2 years have elapsed since the formal discontinuation of clinical development of the investigational product. Patient files and other source data must be kept for the maximum period of time permitted by your institution, but not less than 15 years. Should Investigators be unable to continue maintenance of patient files for the full 15 years, Genzyme will assist in this regard.

These documents should be retained for a longer period, however, if required by the applicable regulatory requirements or by an agreement with the Sponsor. It is the responsibility of the Sponsor to inform the Investigator/institution as to when these documents no longer need to be retained.

Essential documents are those documents, which individually and collectively, permit evaluation of the conduct of a trial and the quality of the data produced. These documents serve to demonstrate the compliance of the Investigator, Sponsor or designee, and monitor with the standards of GCP and with all applicable regulatory requirements.

Any or all of the documents should be available for audit by the Sponsor's or designee's auditor and inspection by the regulatory authority(-ies).

12.3.4 Patient Discontinuation

If a patient decides to discontinue participation in the study, he or she should be contacted in order to obtain information about the reason(s) for discontinuation and collection of any potential AEs. Whenever possible the patient should return to the clinic for the Week 28 assessments. The Investigator will document the eCRF describing the reason for discontinuation.

12.3.5 Study or Site Termination

The Sponsor reserves the right to terminate the study, according to the study contract. For example, the study may be terminated and dosing stopped at any time if the Medical Monitor is of the opinion that proceeding with the study will compromise the safety of the patients in the study. The Investigator should notify the regulatory authority and IRB/IEC in writing of the trial's completion or early termination and provide a copy of the notification to the Sponsor or designee.

12.3.6 Monitoring

The Sponsor representative or designee and regulatory authority inspectors are responsible for contacting and visiting the Investigator for the purpose of inspecting the facilities and, upon request, inspecting the various records of the trial (for example, eCRFs and other pertinent data) provided that patient confidentiality is respected.

The Sponsor representative or designee is responsible for inspecting the eCRFs at regular intervals throughout the study to verify adherence to the protocol; completeness, accuracy, and consistency of the data; and adherence to local regulations on the conduct of clinical research. The monitor should have access to patient medical records and other study-related records needed to verify the entries on the eCRFs.

The Investigator agrees to cooperate with the monitor to ensure that any problems detected in the course of these monitoring visits, including delays in completing eCRFs, are resolved.

In accordance with ICH GCP and the Sponsor's audit plans, this study may be selected for audit by representatives from the Sponsor's Clinical and QA Department (or designees). Inspection of study center facilities (e.g., pharmacy, drug storage areas, laboratories) and review of study-related records will occur to evaluate the trial conduct and compliance with the protocol, ICH GCP, and applicable regulatory requirements.

12.3.7 Materials Control

12.3.7.1 Receipt of Clinical Supplies

The study staff is required to document the receipt of study drug supplies.

12.3.7.2 Disposition of Unused Clinical Supplies

The study staff is required to document the dispensing, accountability, and return of study drug supplies. The study center must return all used and unused study drug as per Sponsor or designee instructions. Note that unused study drug should be maintained in refrigerated storage (2° to 8°C) until returned. Refer to the Investigational Product Handling Manual for additional details.

12.3.8 Warnings, Precautions, Contraindications

For specific information concerning warnings, precautions, and contraindications, the Investigator is asked to refer to the appropriate section of the Investigator’s Brochure. Because of the possibility of AEs, a fully equipped emergency cart, or equivalent supplies and equipment, and personnel competent in recognizing and treating adverse reactions of all types should be immediately available.

Dose-dependent, transient, and reversible prolongations of aPTT have been observed with phosphorothioate oligonucleotides. Therefore, mipomersen should be used with caution in patients receiving warfarin, heparin or fractionated heparin products.

12.3.9 Clinical Study Report

If deemed appropriate by the Sponsor, the Coordinating Investigator shall be designated to sign the completed clinical study report at the end of this study.

The signatory Coordinating Investigator shall be identified by the Sponsor upon the completion of the study, based upon factors including, but not limited to, prior clinical research experience and publications, patient enrollment and level of involvement in the study.

12.3.10 Disclosure of Data

All information obtained during the conduct of this study will be regarded as confidential, and written permission from the Sponsor is required prior to disclosing any information relative to this study. Manuscripts prepared for publication will be in accordance with the policy established and presented to the Investigator previously by the Sponsor. Submission to the Sponsor for review and comment prior to submission to the publisher will be required. This requirement should not be construed as a means of restricting publication, but is intended solely to assure concurrence regarding data, evaluations, and conclusions and to provide an opportunity to share with the Investigator any new and/or unpublished information of which he/she may be unaware.

13 REFERENCES

Chen SH, Habib G, Yang CY, et al. Apolipoprotein B-48 is the product of a messenger RNA with organ-specific in-frame stop codon. *Science*. 1987;238(4825):363–366.

Cholesterol Treatment Trialists' Collaborators [CTTC]. Efficacy and safety of cholesterol-lowering treatment: prospective meta-analysis of data from 90,056 participants in 14 randomised trials of statins. *Lancet.* 2005;366:1267-1278.

Davidson MH, Maki KC, Pearson TA, et al. Results of the National Cholesterol Education Program (NCEP) Evaluation Project Utilizing Novel E-Technology (NEPTUNE) II survey and implications for treatment under the recent NCEP Writing Group Recommendations. *Am J Cardiol.* 2005;96(4):556-563.

Davis RA, Hui TY. 2000 George Lyman Duff Memorial Lecture. Atherosclerosis is a liver disease of the heart. *Arterioscler Thromb Vasc Biol*. 2001;21:887-898.

Gotto AM Jr. Management of dyslipidemia. *Am J Med*. 2002;112(8A):10S-18S.

Grundy SM, Cleeman JI, Merz CN, et al. Implications of recent clinical trials for the National Cholesterol Education Program Adult Treatment Panel III guidelines. *Circulation*. 2004;110:227-239.

Hulthe J, Fagerberg B. Circulating oxidized LDL is associated with subclinical atherosclerosis development and inflammatory cytokines (AIR study). *Arterioscler Thromb Vasc Biol*. 2002;22(7):1162-1167.

Knopp RH. Drug treatment of lipid disorders. *N Engl J Med*. 1999;341(7):498-511.

Third report of the National Cholesterol Education Program (NCEP) expert panel on detection, evaluation, and treatment of high blood cholesterol in adults (Adult Treatment Panel III) Final Report. *Circulation*. 2002;106(25):3143-3421.

Powell LM, Wallis SC, Pease RJ, Edwards YH, Knott TJ, Scott J. A novel form of tissue-specific RNA processing produces apolipoprotein-B48 in intestine. *Cell*. 1987;50(6):831–840.

Sankatsing RR, Fouchier SW, de Haan S, et al. Hepatic and cardiovascular consequences of familial hypobetalipoproteinemia. *Arterioscler Thromb Vasc Biol.* 2005;25:1979-1984.

Skalen K, Gustafsson M, Rydberg EK, et al. Subendothelial retention of atherogenic lipoproteins in early atherosclerosis. *Nature*. 2002;417(6890):750-754.

Sueta CA, Massing MW, Chowdhury M, Biggs DP, Simpson RJ Jr. Undertreatment of hyperlipidemia in patients with coronary artery disease and heart failure. *J Card Failure*. 2003;9(1):36-41.

14 APPENDICES

14.1 NCEP – ATP III Therapeutic Lifestyle Changes

- Reduced intakes of saturated fats (< 7% of total calories) and cholesterol (< 200 mg per day)
- Therapeutic options for enhancing LDL lowering such as plant stanols/sterols (2 g/day) and increased viscous (soluble) fiber (10 to 25 g/day)
- Weight reduction
- Increased physical activity

Table 14-1 Nutrient Composition of the TLC Diet

Nutrient	Recommended Intake
Saturated fat*	Less than 7% of total calories
Polyunsaturated fat	Up to 10% of total calories
Monounsaturated fat	Up to 20% of total calories
Carbohydrate†	50% to 60% of total calories
Fiber	20 to 30 g/day
Protein	Approximately 15% of total calories
Cholesterol	Less than 200 mg/day
Total calories (energy)‡	Balance energy intake and expenditure to maintain desirable body weight/prevent weight gain

*Trans fatty acids are another LDL-raising fat that should be kept at a low intake.
†Carbohydrate should be derived predominantly from foods rich in complex carbohydrates including grains, especially whole grains, fruits, and vegetables.
‡Daily energy expenditure should include at least moderate physical activity (contributing approximately 200 kcal per day)

14.2 Lipoprotein Testing

Lipoprotein testing will be performed in a clinical laboratory which holds a current certification of traceability to the national reference system for total cholesterol. In addition, the analytical systems and reagents used to measure total cholesterol and HDL-C must be certified by the Cholesterol Reference Method Laboratory Network (CRMLN) as having documented traceability to the national reference system for total cholesterol, and the designated comparison method for HDL-C, respectively.

Table 14-2 Lipoprotein Testing

Lipoprotein	Analysis Method
Total Cholesterol	Enzymatic colorimetry
LDL-C	Friedewald Calculation; ultracentrifugation for TG > 400
HDL-C	Dextran sulfate precipitation followed by enzymatic colorimetry
Triglycerides	Enzymatic colorimetry
Apo B-100	Rate Nephelometry
Apo A-1	Rate Nephelometry
Lp(a)	Isoform Independent assay standardized to Northwest Lipid Research Clinic

14.3 NCEP - ATP III Guidelines for Determining >20% 10-Year Risk for CHD

Risk Factors (one point for each risk factor present):

- Cigarette smoking
- Hypertension (BP ≥ 140/90 mm Hg or on antihypertensive medication)
- Low HDL-C (< 40 mg/dL)*
- Family history of premature CHD (CHD in male first degree relative < 55 years; CHD in female first degree relative < 65 years)
- Age (men ≥ 45 years; women ≥ 55 years)

***HDL-C ≥ 60 mg/dL counts as a 'negative' risk factor; its presence removes one risk factor from the total count.**

Note: If patient has 2 or more risk factors, please calculate the Framingham 10-Year CHD Risk using the following pages or using the online calculator at the website:

http://hp2010.nhlbihin.net/atpiii/calculator.asp?usertype=pub

Framingham Risk Scale

For each Patient (as appropriate), please circle the appropriate point value.

Men: Age (years)	Men: Points	Women: Age (years)	Women: Points
20-34	-9	20-34	-7
35-39	-4	35-39	-3
40-44	0	40-44	0
45-49	3	45-49	3
50-54	6	50-54	6
55-59	8	55-59	8
60-64	10	60-64	10
65-69	11	65-69	12
70-74	12	70-74	14
75-79	13	75-79	16

Points

Men: Total Cholesterol (mg/dL)	Age (years) 20-39	40-49	50-59	60-69	70-79	Women: Total Cholesterol (mg/dL)	Age (years) 20-39	40-49	50-59	60-69	70-79
< 160	0	0	0	0	0	< 160	0	0	0	0	0
160-199	4	3	2	1	0	160-199	4	3	2	1	1
200-239	7	5	3	1	0	200-239	8	6	4	2	1
240-279	9	6	4	2	1	240-279	11	8	5	3	2
≥ 280	11	8	5	3	1	≥ 280	13	10	7	4	2

	Points				
	Age (years)				
	20-39	40-49	50-59	60-69	70-79
**Non-Smoker**	0	0	0	0	0
**Smoker**	8	5	3	1	1

HDL-C (mg/dL)	Points
≥ 60	-1
50-59	0
40-49	1
< 40	2

Systolic BP (mmHg)	If Untreated	If Treated
< 120	0	0
120-129	0	1
130-139	1	2
140-159	1	2
≥ 160	2	3

	Points				
	Age (years)				
	20-39	40-49	50-59	60-69	70-79
**Non-Smoker**	0	0	0	0	0
**Smoker**	9	7	4	2	1

HDL-C (mg/dL)	Points
≥ 60	-1
50-59	0
40-49	1
< 40	2

Systolic BP (mmHg)	If Untreated	If Treated
< 120	0	0
120-129	1	3
130-139	2	4
140-159	3	5
≥ 160	4	6

*Framingham Risk Score Points Totals**

Age Score	
Total Cholesterol Score	
Smoking Status Score	
HDL-C Score	
Systolic Blood Pressure Score	
Total Score	
**Framingham 10-Year CHD Risk %:**	

Framingham Risk Scale

**Men**		**Women**	
Point Total	10-Year Risk (%)	Point Total	10-Year Risk (%)
<0	<1	<9	<1
0	1	9	1
1	1	10	1
2	1	11	1
3	1	12	1
4	1	13	2
5	2	14	2
6	2	15	3
7	3	16	4
8	4	17	5
9	5	18	6
10	6	19	8
11	8	20	11
12	10	21	14
13	12	22	17
14	16	23	22
15	20	24	27
16	25	≥25	≥30
≥17	≥30		

Please circle total points above.

14.4 Pregnancy Notification Forms
